# Plant diversity modifies multi-trophic interactions in croplands, grasslands and forests

**DOI:** 10.1126/sciadv.aeb8680

**Published:** 2026-07-23

**Authors:** Nian-Feng Wan, Muyao Li, Siyuan Shen, Jun-Xiang Zhou, Yu-Tong Zhu, Ben A. Woodcock, Yue-Qing Hu, Shinichi Nakagawa, David Tilman, Andy Hector, Michel Loreau, Peter B. Reich, Matteo Dainese, Forest Isbell, Bernhard Schmid, Kris A. G. Wyckhuys, Geoff M. Gurr, Linsheng Wan, Nico Eisenhauer, Yann Hautier, Anu Eskelinen, Richard D. Bardgett, Paul Kardol, Lauchlan H. Fraser, Qi Su, Shiyun Qiu, Yavanna Aartsma, Ramon Albajes, Matthias Albrecht, Md Panna Ali, Hugo Alejandro Alvarez, Corinne Anagonou, Jan Christoph Axmacher, Dan Bahauddin, Yongfei Bai, Elisabeth S. Bakker, Péter Batáry, Felix J. J. A. Bianchi, Fabian A. Boetzl, Jana Brandmeier, Helge Bruelheide, Buntika Areekul Butcher, Abida Butt, Zhiping Cai, Alistair John Campbell, Fengqin Cao, Simone Cesarz, Lourdes Chamorro-Lorenzo, Fajun Chen, Jing-Ting Chen, Julian Chen, Li-Lin Chen, Yiying Chen, Gemma Clemente-Orta, Luuk Croijmans, David Crowder, Jéssica Karina da Silva Pachú, Anicet Dassou, Francisco de Sousa Ramalho, Thomas F. Döring, Anne Ebeling, Francisco Sales Fernandes, Stefan Geisen, Tesfay Gidey, Wesley Augusto Conde Godoy, Jingwei Guo, Séverin Hatt, George Heimpel, Johannes Heinze, Chris Helzer, Finbarr G. Horgan, Xiaoli Hu, Zixuan Huang, Stine Kramer Jacobsen, Hervé Jactel, Anthony Joern, Mattias Jonsson, Agnes Waringa Kiriga, Margaret Kosmala, Luhui Kuang, Sarwan Kumar, Minoo Heidari Latiberi, Chaoxu Li, Feng-Rui Li, Frank Yonghong Li, Jinhua Li, Likun Li, Qi Li, Xiaofei Li, Yi Li, Yingbin Li, Ji-Liang Liu, Jushan Liu, Wei-Jia Liu, Yinzhan Liu, Zhanfeng Liu, Gianalberto Losapio, Xiaoming Lu, Ola Lundin, Quanhui Ma, Filipe Madeira, José Bruno Malaquias, Mostafa Ghafouri Moghaddam, Ana Carolina Monmany-Garzia, Xoaquín Moreira, Marina Morente, Bretor Katuku Mutua, Daniel Munyao Mutyambai, Yuta Nakano, Vojtech Novotny, Lawrence O. Ochieng, Silvia Pappagallo, Daniel Paredes, Jana S. Petermann, Erik H. Poelman, Orelvis Portal, Ming-Jing Qu, Yordanys Ramos, Syed Rizvi, Francisca Ruano, Weiguo Sang, F. Xavier Sans-Serra, Cornelia Sattler, Christian Schöb, Julian Schrader, Andreas Schuldt, Josef Settele, Jing Shang, Lene Sigsgaard, Daniel Ricardo Sosa-Gómez, Michael Staab, Ingolf Steffan-Dewenter, Philip C. Stevenson, Cory Straub, Edison Ryoiti Sujii, Louis Sutter, Piotr Szefer, Hafiz Muhammad Tahir, Xiaoling Tan, Aimi Tanada, Pedro Henrique Brum Togni, Edina Török, Alba Tous-Fandos, Silvère Tovignan, Matthias Tschumi, Alina Twerski, Shunsuke Utsumi, Dirk F. van Apeldoorn, Cassandra Vogel, Felix L. Wäckers, Deli Wang, Lianjie Wang, Ling Wang, Ming-Qiang Wang, Su Wang, Xiaodong Wang, Xinyu Wang, Yadong Wang, Yong-Zhen Wang, Wolfgang W. Weisser, Ellen A. R. Welti, Anna Wenda-Piesik, Wenjia Wu, Xinqiang Xi, Qingxuan Xu, Zhenzhu Xu, Huimin Yi, Lixia Zhang, Xiaoke Zhang, Xiaoming Zhang, Zhengqun Zhang, Zhiwei Zhong, Chao-Dong Zhu, Hui Zhu, Pingyang Zhu, Yu Zhu, Yi Zou, Christoph Scherber

**Affiliations:** ^1^ Shanghai Key Laboratory of Chemical Biology, State Key Laboratory of Bioreactor Engineering, School of Pharmacy, East China University of Science and Technology, Shanghai 200237, China.; ^2^ State Key Laboratory of Submarine Geoscience, School of Automation and Intelligent Sensing, Shanghai Jiao Tong University, Shanghai 200240, China.; ^3^ State Key Laboratory of Genetic Engineering, Institute of Biostatistics, School of Life Sciences, Fudan University, Shanghai 200438, China.; ^4^ UK Centre for Ecology and Hydrology, Wallingford OX10 8BB, UK.; ^5^ Department of Biological Sciences, University of Alberta, CW 405, Biological Sciences Building, Edmonton, Alberta T6G 2E9, Canada.; ^6^ Department of Ecology, Evolution and Behavior, University of Minnesota, Saint Paul, MN 55108, USA.; ^7^ Bren School of Environmental Science and Management, University of California, Santa Barbara, CA 93106-5131, USA.; ^8^ Department of Biology, University of Oxford, Oxford OX1 3EL, UK.; ^9^ Theoretical and Experimental Ecology Station, CNRS, 2 route du CNRS, Moulis, 09200 France.; ^10^ Institute of Ecology, College of Urban and Environmental Sciences, Peking University, Beijing 100871, China.; ^11^ Department of Forest Resources, University of Minnesota, St. Paul, MN 55108, USA.; ^12^ Institute for Global Change Biology, School for Environment and Sustainability, University of Michigan, Ann Arbor, MI 48109, USA.; ^13^ Hawkesbury Institute for the Environment, Western Sydney University, Penrith, New South Wales 2751, Australia.; ^14^ Department of Biotechnology, University of Verona, Strada Le Grazie 15, 37134 Verona, Italy.; ^15^ Remote Sensing Laboratories, Department of Geography, University of Zurich, Winterthurerstrasse 190, CH-8057 Zurich, Switzerland.; ^16^ Chrysalis Consulting, Danang 550000, Vietnam.; ^17^ State Key Laboratory for Biology of Plant Diseases and Insect Pests, Institute of Plant Protection, Chinese Academy of Agricultural Sciences, Beijing 100193, China.; ^18^ School of the Environment, University of Queensland, Saint Lucia, QLD 4072, Australia.; ^19^ Gulbali Institute, Charles Sturt University, Orange, New South Wales 2800, Australia.; ^20^ Jiangsu Coastal Area Institute of Agricultural Sciences, Yancheng 224002, China.; ^21^ German Centre for Integrative Biodiversity Research (iDiv) Halle-Jena-Leipzig, Puschstraße 4, Leipzig, 04103, Germany.; ^22^ Institute of Biology, Leipzig University, Puschstraße 4, Leipzig, 04103, Germany.; ^23^ Ecology and Biodiversity Group, Department of Biology, Utrecht University, Padualaan 8, 3584 CH, Utrecht, Netherlands.; ^24^ Department of Ecology and Genetics, University of Oulu, Oulu 90014, Finland.; ^25^ Lancaster Environment Centre, Lancaster University, Lancaster LA1 4YQ, UK.; ^26^ Department of Forest Mycology and Plant Pathology, Swedish University of Agricultural Sciences, 750 07 Uppsala, Sweden.; ^27^ Department of Natural Resource Science, Thompson Rivers University, Kamloops, BC V2C 0C8, Canada.; ^28^ Wetland Research Department, Shanghai Wildlife and Protected Natural Areas Research Center, Shanghai 202150, China.; ^29^ Laboratory of Entomology, Wageningen University & Research, P.O. Box 8031, Wageningen 6700 EA, Wageningen, Netherlands.; ^30^ Department of Crop and Forest Sciences, Universitat de Lleida, Agrotecnio Center, Rovira Roure 191, 25198 Lleida, Spain.; ^31^ Agroscope, Agroecology and Environment, Reckenholzstrasse 191 8046 Zurich, Switzerland.; ^32^ Crops Division, Bangladesh Agricultural Research Council, Farmgate, Dhaka 1215, Bangladesh.; ^33^ Department of Zoology, Faculty of Sciences, University of Granada, Av. Fuente Nueva s/n, 18071 Granada, Spain.; ^34^ Department of Biogeography and Climate Change, National Museum of Natural Sciences (CSIC), C/ Serrano 115 bis, E-28006 Madrid, Spain.; ^35^ National School of Biosciences and Applied Biotechnologies (ENSBBA), National University of Sciences, Technologies, Engineering and Mathematics (UNSTIM), Dassa, 999105, Benin.; ^36^ Department of Geography, University College London, London WC1E 6BT, UK.; ^37^ Key Laboratory of Vegetation and Environmental Change, Institute of Botany, Chinese Academy of Sciences, Beijing 100093, China.; ^38^ Department of Aquatic Ecology, Netherlands Institute of Ecology (NIOO-KNAW), Wageningen 6708 PB Netherlands.; ^39^ Wildlife Ecology and Conservation Group, Wageningen University & Research, Droevendaalsesteeg 3a, 6708 PB, Wageningen, Netherlands.; ^40^ “Lendület” Landscape and Conservation Ecology, Institute of Ecology and Botany, MTA–HUN-REN Centre for Ecological Research, 2163, Vácrátót, Hungary.; ^41^ Farming Systems Ecology group, Wageningen University and Research, P.O. Box 338, Wageningen 6700 AH, Netherlands.; ^42^ Department of Ecology, Swedish University of Agricultural Sciences, Box 7044, 750 07 Uppsala, Sweden.; ^43^ Institute of Landscape Ecology, University of Muenster, Heisenbergstraße 2, 48149 Muenster, Germany.; ^44^ Leibniz Institute for the Analysis of Biodiversity Change, Museum Koenig Bonn, Adenauerallee 127, 53113 Bonn, Germany.; ^45^ Institute of Biology/Geobotany and Botanical Garden, Martin-Luther-University Halle-Wittenberg, Am Kirchtor 1, D3206108, 06108 Halle, Germany.; ^46^ Integrative Insect Ecology Research Unit, Department of Biology, Faculty of Science, Chulalongkorn University, Bangkok 10330, Thailand.; ^47^ Department of Zoology, University of Punjab, Lahore 54590, Punjab, Pakistan.; ^48^ College of Agriculture, Shihezi University, Shihezi 832003, China.; ^49^ Laboratório de Entomologia, Embrapa Amazônia Oriental, CEP, 66095-903 Belém, Pará, Brasil.; ^50^ School of Tropical Agriculture and Forestry, Hainan University, Danzhou, Hainan 571737, China.; ^51^ Departament de Biologia Evolutiva, Ecologia i Ciències Ambientals (Botànica i Micologia), Facultat de Biologia, Universitat de Barcelona (UB), Barcelona 08028, Spain.; ^52^ Institut de Recerca de la Biodiversitat (IRBio), Universitat de Barcelona (UB), Barcelona 08028, Spain.; ^53^ Department of Entomology, Nanjing Agricultural University, Nanjing 210095, China.; ^54^ State Key Laboratory of Animal Biodiversity Conservation and Integrated Pest Management, Institute of Zoology, Chinese Academy of Sciences, Beijing 100101, China.; ^55^ State Key Laboratory of Agricultural and Forestry Biosecurity, College of Plant Protection, Fujian Agriculture and Forestry University, Fuzhou, Fujian 350002, China.; ^56^ Centre for cropping systems Analysis, Wageningen University & Research, P.O. Box 430, 6700 AK Wageningen, Netherlands.; ^57^ Department of Entomology, Washington State University, Pullman, WA 99164, USA.; ^58^ Department of Entomology, Luiz de Queiroz College of Agriculture (ESALQ), Piracicaba, SP 13418-900‌, Brazil.; ^59^ Biological Control Unit, Embrapa Cotton, Campina Grande, PB 58428-095, Brazil.; ^60^ Centre for Biological Diversity, School of Biology, St. Andrews University, Fife KY16 9TH, UK.; ^61^ Agroecology and Organic Farming group, Institute of Crop Science and Resource Conservation, University of Bonn, Auf dem Hügel 6, 53121 Bonn, Germany.; ^62^ Institute of Biodiversity, Ecology and Evolution, University Jena, Dornburger Strasse 159, 07743 Jena, Germany.; ^63^ Laboratory of Nematology, Plant Science Group, Wageningen University & Research (WU), 6700 AJ Wageningen, Netherlands.; ^64^ Department of Forest Botany, Dendrology and Geobiocoenology, Faculty of Forestry and Wood Technology, Mendel University in Brno, Zemědělská 1613 00 Brno, Czech Republic.; ^65^ Department of Plant Science, College of Agriculture and Environmental Sciences, Adigrat University, P.O. Box 50, Adigrat, Ethiopia.; ^66^ International Joint Research Laboratory for Global Change Ecology, Laboratory of Biodiversity Conservation and Ecological Restoration, School of Life Sciences, Henan University, Kaifeng 475004, Henan, China.; ^67^ Natagriwal asbl, Gembloux site, Passage des Déportés 2, 5030 Gembloux, Belgium.; ^68^ Department of Entomology, University of Minnesota, St. Paul, MN, USA.; ^69^ Heinz Sielmann Foundation, Department of Biodiversity, Zur Döberitzer Heide 9, 14641 Wustermark / Elstal, Germany.; ^70^ Institute of Biochemistry and Biology, University of Potsdam, Maulbeerallee 1, 14469 Potsdam, Germany.; ^71^ The Nature Conservancy, Nebraska Chapter, P.O. Box 438, Aurora, NE 68818 USA.; ^72^ EcoLaVerna Integral Restoration Ecology, Bridestown, Kildinan, T56 P499 County Cork, Ireland.; ^73^ Faculty of Agrarian and Forest Sciences, School of Agronomy, Catholic University of Maule, Casilla 7-D, Curicó 3349001, Chile.; ^74^ Department of Ecology, School of Life Sciences, Nanjing University, Nanjing, 210093, China.; ^75^ Department of Plant and Environmental Sciences, University of Copenhagen, Thorvaldsensvej 40, 1871 Frederiksberg C, Denmark.; ^76^ INRAE, University of Bordeaux, Biogeco, F-33610 Cestas, France.; ^77^ Division of Biology, Kansas State University, 116 Ackert Hall, Manhattan, KS 66506 USA.; ^78^ International Centre of Insect Physiology and Ecology, P.O Box 30772-0010, Nairobi, Kenya.; ^79^ South China Botanical Garden, Chinese Academy of Sciences, Guangzhou 510650, China.; ^80^ Department of Plant Breeding and Genetics, Punjab Agricultural University, Ludhiana 141 004, Punjab, India.; ^81^ Coconut Research Institute, Chinese Academy of Tropical Agricultural Sciences, Wenchang, Hainan 571339, China.; ^82^ Northwest Institute of Eco-Environment and Resources, Chinese Academy of Sciences, Lanzhou 730000, China.; ^83^ Ministry of Education Key Laboratory of Ecology and Resource Use of the Mongolian Plateau, Inner Mongolia Key Laboratory of Grassland Ecology & Observation and Research Station for the Typical Steppe Ecosystem of the Ministry of Education, School of Ecology and Environment, Inner Mongolia University, Hohhot 010021, China.; ^84^ Institute of Applied Ecology, Chinese Academy of Sciences, Shenyang 110016, China.; ^85^ College of Resources and Environmental Sciences, Jilin Agricultural University, Changchun, Jilin 130118, China.; ^86^ Key Laboratory of Vegetation Ecology of the Ministry of Education, Jilin Songnen Grassland Ecosystem National Observation and Research Station, Institute of Grassland Science, Northeast Normal University, Changchun 130024, China.; ^87^ Pest Bio-control Laboratory, Shandong Peanut Research Institute, Qingdao 266041, China.; ^88^ International Joint Research Laboratory for Global Change Ecology, Laboratory of Biodiversity Conservation and Ecological Restoration, School of Life Sciences, Henan University, Kaifeng 475005, Henan, China.; ^89^ Department of Biosciences, University of Milan, 20133 Milan, Italy.; ^90^ Research Center in Natural Resources, Environment and Society (CERNAS), Santarém Polytechnic University, Quinta do Galinheiro - S. Pedro, 2001-904 Santarém, Portugal.; ^91^ School of Agriculture, Santarém Polytechnic University, Quinta do Galinheiro - S. Pedro, 2001-904 Santarém, Portugal.; ^92^ Entomology Laboratory, Federal University of Paraiba, 12 Rodovia, PB-079, Areia 58397-000, PB, Brazil.; ^93^ Centre for Insect Systematics, Department of Biological Sciences and Biotechnology, Faculty of Science and Technology, Universiti Kebangsaan Malaysia, 43600 Bangi, Selangor, Malaysia.; ^94^ Instituto de Ecología Regional (Universidad Nacional de Tucumán-CONICET), Tucumán 4107, Argentina.; ^95^ Misión Biológica de Galicia (MBG-CSIC), Apartado de Correos 28, 36080 Pontevedra, Galicia, Spain.; ^96^ South Eastern Kenya University, P.O Box 170-90200, Kitui, Kenya.; ^97^ Graduate School of Environmental Science, Hokkaido University, Sapporo, Hokkaido 0600810, Japan.; ^98^ Biology Centre, Institute of Entomology, Czech Academy of Sciences, Branišovská 1160/31, 370 05 České Budějovice, Czech Republic.; ^99^ Faculty of Science, University of South Bohemia, Branišovská 1645/31a, 370 05 České Budějovice, Czech Republic.; ^100^ Department of Crops, Horticulture and Soils, Egerton University, Njoro 20115, Kenya.; ^101^ Department of Vegetal Biology, Ecology and Earth Science, University of Extremadura, Avd. de Elvas s/n, 06006 Badajoz, Spain.; ^102^ Department of Environment and Biodiversity, University of Salzburg, 5020 Salzburg, Austria.; ^103^ Centro de Investigaciones Agropecuarias, Universidad Central “Marta Abreu” de Las Villas, Santa Clara 54830, Villa Clara, Cuba.; ^104^ Instituto de Investigaciones Agropecuarias y Forestales, Universidad Michoacana de San Nicolás de Hidalgo, Tarímbaro 58880, Michoacán, Mexico.; ^105^ Applied Biosciences, Macquarie University, North Ryde, New South Wales 2109, Australia.; ^106^ College of Life and Environmental Sciences, Minzu University of China, Beijing 100081, China.; ^107^ School of Natural Sciences, Macquarie University, Sydney, New South Wales 2109, Australia.; ^108^ Departamento de Biología y Geología, Física y Química Inorgánica & Instituto de Investigación en Cambio Global (IICG-URJC), Universidad Rey Juan Carlos, c/ Tulipan s/n, 28933 Móstoles, Spain.; ^109^ Forest Nature Conservation, University Göttingen, Büsgenweg 3, 37077 Göttingen, Germany.; ^110^ Department of Conservation Biology & Social-Ecological Systems Helmholtz-Centre for Environmental Research (UFZ), Permoserstraße 15, 04318 Leipzig, Germany.; ^111^ Institute of Biological Sciences, College of Arts and Sciences, University of the Philippines, Los Baños, College, Los Baños 4031, Philippines.; ^112^ College of Agronomy, Sichuan Agricultural University/Sichuan Engineering Research Center for Crop Strip Intercropping System, Chengdu 611130, China.; ^113^ Department of Plant Sciences, Norwegian University of Life Sciences, Postboks 5003, 1432 Ås, Norway.; ^114^ Embrapa Soybean, Londrina, 86001-970 Paraná, Brazil.; ^115^ Institute of Ecology, Leuphana University Lüneburg, Universitätsallee 1, 21335 Lüneburg, Germany.; ^116^ Department of Animal Ecology and Tropical Biology, University of Würzburg, Würzburg 97074, Germany.; ^117^ Natural Resources Institute, University of Greenwich, Central Avenue, Chatham Maritime, Kent ME4 4TB. UK.; ^118^ Royal Botanic Gardens, Kew, Kew Green, Richmond, Surrey TW9 3AE UK.; ^119^ Department of Biology, Ursinus College, Collegeville, PA 19426-1000, USA.; ^120^ Embrapa Recursos Genéticos e Biotecnologia-EMBRAPA CENARGEN, 70770917 Brasília, Brazil.; ^121^ Agroscope, Plant-Production Systems, Route des Eterpys 18, 1964 Conthey, Switzerland.; ^122^ Department of Zoology, Government College University Lahore, 54000, Pakistan.; ^123^ Departamento de Ecologia, Instituto de Ciências Biológicas, Universidade de Brasília (UnB), Asa Norte, 70910900, Brasília, DF, Brazil.; ^124^ Laboratory of Economic and Social Dynamics Analysis (LARDES), Faculty of Agronomy (FA), University of Parakou (UP), Parakou 999105, Benin.; ^125^ Swiss Ornithological Institute, Seerose 1, Sempach CH-6204, Switzerland.; ^126^ Department of Nature Conservation, Anhalt University of Applied Sciences, Bernburg 06406, Germany.; ^127^ Faculty of Environmental Earth Science, Hokkaido University, Sapporo, Hokkaido 0600810, Japan.; ^128^ Field Crops, Wageningen University & Research, 6708 PB, Wageningen, the Netherlands.; ^129^ Ecology of Tropical Agricultural Systems, University of Hohenheim, Stuttgart 70559, Germany.; ^130^ Lancaster Environment Centre, Lancaster University, Lancaster LA1 4YO, UK.; ^131^ Biobest Group, Westerlo 2260, Belgium.; ^132^ Haikou Fuman Sanjiang Agricultural Development Co. LTD., Hainan 571133, China.; ^133^ Mountain Ecological Restoration and Biodiversity Conservation Key Laboratory of Sichuan Province, Chengdu Institute of Biology, Chinese Academy of Sciences, 610213 Chengdu, China.; ^134^ Institute of Plant Protection, Beijing Academy of Agriculture and Forestry Sciences, Beijing 100097, China.; ^135^ Institute of Industrial Crops, Jiangsu Academy of Agricultural Sciences, Key Laboratory of Cotton and Rapeseed, Ministry of Agriculture and Rural Affairs, Nanjing 210014, China.; ^136^ Terrestrial Ecology Research Group, Department of Life Science Systems, School of Life Sciences, Technical University of Munich, 85354 Freising, Germany.; ^137^ Great Plains Science Program, Smithsonian’s National Zoo and Conservation Biology Institute, Bozeman, MT 59718, USA.; ^138^ Department of Agronomy and Food Processing, Faculty of Agriculture and Biotechnology, Bydgoszcz University of Science and Technology, Al. Kaliskiego 7, 85-796 Bydgoszcz, Poland.; ^139^ College of Horticulture Science and Engineering, Shandong Agricultural University, Tai’an 271018, China.; ^140^ State Key Laboratory for Conservation and Utilization of Bio-Resources in Yunnan, College of Plant Protection, Yunnan Agricultural University, Kunming 650201, China.; ^141^ Heilongjiang Xingkai Lake Wetland Ecosystem National Observation and Research Station, Northeast Institute of Geography and Agroecology, Chinese Academy of Sciences, Changchun, Jilin 130102, China.; ^142^ State Key Laboratory of Black Soils Conservation and Utilization, Northeast Institute of Geography and Agroecology, Chinese Academy of Sciences, Changchun, Jilin 130102, China.; ^143^ College of Life Science, Zhejiang Normal University, Jinhua 321004, China.; ^144^ Department of Health and Environmental Sciences, School of Science, Xi’an Jiaotong-Liverpool University, Suzhou 215123, China.; ^145^ Bonn Institute for Organismic Biology, University of Bonn, Endenicher Allee 1, 53113 Bonn, Germany.

## Abstract

Multitrophic interactions can strongly influence the structure and functioning of ecosystems, but how plant diversity influences the direction and predictability of multitrophic interactions across agricultural and natural ecosystems remains unclear. Using 149 field studies across five continents, we found that, on average, increasing plant diversity tended to exert differential top-down and bottom-up effects in croplands versus grasslands and forests. Organic and nonorganic croplands exhibited 846 and 148% higher invertebrate natural enemy-to-herbivore abundance ratios under increased plant diversity, consistent with top-down control patterns where predator gains cause herbivore declines, enhancing crop outcomes. In grasslands and forests, increasing plant diversity was associated with bottom-up effects where enhanced productivity increased both herbivore and predator populations, with the enemy-to-herbivore ratio increasing 4.73% for grasslands and 21.2% for forests. Our findings suggest that biodiversity effects on productivity are not solely explained by direct plant-plant interactions and the resulting biodiversity-productivity relationship. Rather, they reveal patterns consistent with the framework of top-down and bottom-up effects, the relative balance of which may vary depending on ecosystem and management type. The magnitude of the effects of diversified farming on crop pests suggests that crop diversification may be an important avenue for managing crop pests preventatively and thereby enhancing agricultural sustainability.

## Teaser

Plant diversity differentially affects trophic interactions across ecosystems.

## INTRODUCTION

Multitrophic interactions are universal in ecology and play a fundamental role in structuring biological communities (*1*–*7*). These interactions (e.g., trophic cascades) occur when population or behavior changes at one trophic level propagate through other levels in a food web. They can be mediated by both top-down and bottom-up processes (*8*, *9*). In top-down trophic interactions, predators indirectly promote plant productivity by suppressing herbivores that would otherwise consume plants. Such effects have been widely observed across terrestrial (*5*, *10*) and aquatic systems (*3*, *11*). Experimental studies have demonstrated that top-down control may be driven by increases in predator populations (*12*), as well as by more complex ecological factors such as habitat overlap among predators (*13*), the availability of alternative prey (*14*), and climate-mediated shifts in species interactions (*8*). In contrast, bottom-up interactions occur when changes in plant productivity affect herbivores, which in turn affect their predators. These interactions are often initiated by increased plant biodiversity and biomass (*6*, *15*, *16*) and are shaped by factors, such as soil fertility (*3*, *17*) or the presence of plant secondary metabolites that attract herbivores (*18*). However, top-down and bottom-up effects can act simultaneously and involve different resource channels (*8*). These may include plant-herbivore and decomposer pathways, which can have important implications for sustaining predator populations and driving top-down cascading effects, especially in agricultural systems.

Understanding biodiversity-driven multitrophic interactions is not only important for advancing ecological theory but also has major implications for ecosystem management (*4*, *6*, *10*, *13*). Croplands, grasslands, and forests can exist as either managed monocultures or species-diverse ecosystems. Whether multitrophic interactions become more top-down (predator-driven) or more bottom-up (plant-driven) in response to increased plant diversity, or respond to both drivers (*6*, *12*, *19*, *20*), is therefore a critical question that directly affects the capacity of ecosystems to sustainably produce biomass (e.g., crops, timber, and fodder) and various ecosystem services. To assess the generality and applied relevance of such relationships, it is necessary to examine how they vary across plant life forms (herbaceous versus woody), climatic regions (temperate versus tropical), and study types (managed versus observational).

Plant diversity can influence multitrophic interactions through two main pathways. First, diverse plant communities may enhance predator populations by increasing habitat complexity, offering chemical cues that signal herbivore presence, and providing alternative food sources such as pollen, which can enhance predator reproduction (*21*, *22*). According to the classic enemies hypothesis (EH), increased plant diversity enhances natural enemy abundance and effectiveness, thereby strengthening top-down control of herbivores, including agricultural pests (*23*, *24*). While this hypothesis has gained empirical support in simplified cropping systems, typically with just one to three crop species (*21*), its relevance in more complex and diverse ecosystems such as grasslands or forests remains poorly understood. Second, plant diversity can increase primary productivity through mechanisms predicted by the niche complementarity hypothesis. Niche differences among species in diverse communities enhance overall resource capture and use efficiency, thereby boosting plant biomass (*24*–*31*). In turn, greater plant abundances and structural heterogeneity support larger and more diverse herbivore communities (*6*, *32*, *33*). This herbivore response can trigger a bottom-up trophic interactions, whereby growing herbivore populations support increased abundance or activity of their predators. Such multitrophic interactions have been documented in both grasslands (*10*, *28*, *32*–*34*) and forests (*35*–*37*), where they shape consumer dynamics and ecosystem functioning.

In reality, multiple bottom-up and top-down interactions often operate simultaneously. However, the net direction of their effects may depend on the underlying diversity of primary producers. Croplands typically exhibit lower baseline plant diversity, where conventional pest management practices prioritize yield security. However, strategic crop diversification can activate the EH mechanisms (*22*, *38*), boosting natural enemy recruitment and consequently strengthening top-down control. In floristically diverse grasslands and forests, niche complementarity among plant species increases productivity and the diversity of biomass, with evidence suggesting that this will first benefit herbivores and then predators potentially suppressing herbivore populations as plant productivity and predator abundances increase (*6*, *10*, *35*). While plant diversity-mediated bottom-up and top-down effects often operate simultaneously, which mechanism dominates has important implications for natural enemy-mediated biological control. For example, top-down control can suppress herbivores preemptively, reducing the likelihood of pest outbreaks and economic losses. In contrast, bottom-up control depends on herbivore population growth to trigger predator responses, which may come too late to prevent damage, particularly in managed agroecosystems.

**Fig. 1.**
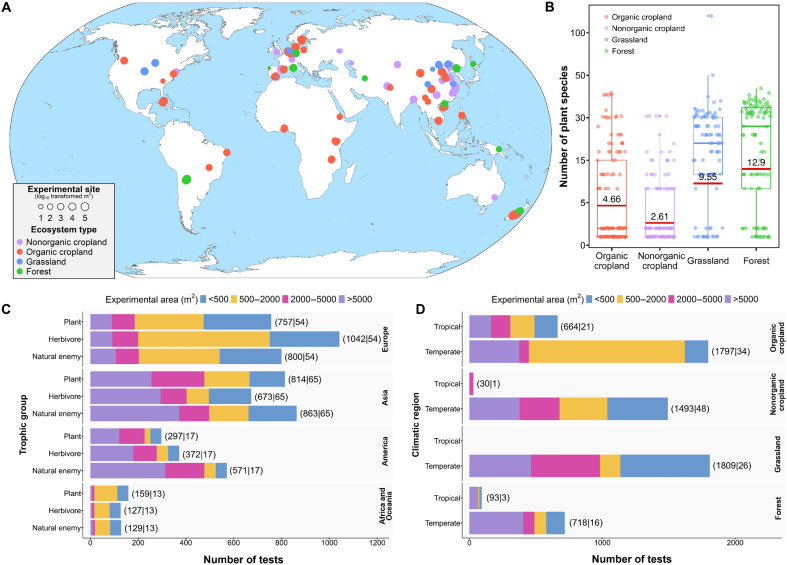
Global 149 field experiments from 27 countries across 5 continents to test plant diversity effects on the tritrophic interactions of plants, invertebrate herbivores, and their natural enemies. (**A**) Study locations [104 studies from 136 locations for organic and nonorganic croplands, 26 studies from 17 locations for grasslands including the largest grassland biodiversity experiments in Cedar Creek of USA and Jena of Germany, and 19 studies from 61 locations for forests including the largest forest biodiversity experiments in China; 23 experiments included more than one study location (range 2 to 25)]. (**B**) Number of plant species in organic and nonorganic croplands, grasslands, and forests (lines within each box denote the median; outer borders of the boxes show the upper and lower quartile; lines outside of the boxes depict the maximum and minimum number of individuals; and red lines in each box represent the average value of number of plant species richness). (**C**) Number of tests for the tritrophic groups in five continents (Asia, Africa, Europe, America, and Oceania) with different experimental areas (sample sizes are shown as number of tests | number of experiments to the right of each bar). (**D**) Number of tests for the tritrophic groups in different terrestrial ecosystems (organic and nonorganic croplands, grasslands, and forests) and climatic regions (tropical and temperate) with different experimental areas (sample sizes are shown as number of tests | number of experiments to the right of each bar).

To systematically decode what may be highly context-dependent mechanisms of plant diversity, we compiled a database comprising 149 field studies that assessed the responses of organisms across trophic groups (i.e., plants, invertebrate herbivores, and natural enemies) to changes in plant diversity (Fig. 1A). This dataset includes 55 organic and 49 nonorganic cropland studies (all implementing pest management through pesticides and/or pesticide alternative technologies), 13 managed and 13 observational grassland studies (including the long-term biodiversity experiments at Cedar Creek, USA and Jena, Germany), and 11 managed and 8 observational forest studies (including China’s large-scale forest diversity experiments) (data S1 and S2). Crop-feeding herbivores are managed in different ways in organic and nonorganic croplands. Whereas agroecological preventative measures such as cultural (e.g., crop rotation and sanitary practices) or physical control (e.g., trapping and mechanical removal) are extensively adopted in organic systems, conventional farmers often rely upon chemical control, i.e., in which synthetic pesticides are used in a curative but also occasionally in a prophylactic manner (see Supplementary Text). Invertebrate herbivore management in the 45 studies from both grasslands and forests was absent. The managed experiments (e.g., the Cedar Creek and Jena grassland biodiversity experiments) isolate causality via controlled diversity gradients, while observational studies monitor natural covariation between diversity and ecosystem processes under real-world conditions. Both study types consistently show positive diversity-productivity relationships (*24*, *26*, *29*–*31*, *39*–*41*) and so were combined here to improve our interpretation of the generality of trophic multitrophic interactions across spatial scales and ecosystem types.

As keystone mediators of multitrophic interactions, invertebrate herbivores structurally link top-down and bottom-up cascades that can be fundamentally shaped also by plant diversity (*6*, *21*, *22*, *37*). Beyond the plant-herbivore (green) resource channel, we also recognize the importance of the decomposer (brown) resource channel. Here, detritivores and microbial decomposers drive nutrient cycling and energy flow, which can indirectly support predator populations and modulate trophic cascades, particularly in systems with high organic matter input such as organic croplands and forest floors (*19*, *20*). While the use of insecticides is widespread in most conventional crop systems and may result in potential nontarget effects on beneficial natural enemies, the contribution of natural pest control remains an important contributor to controlling herbivorous insects (*21*, *22*). In pest-managed croplands, plant diversity may be expected to increase the natural enemy-to-herbivore abundance ratio independent of whether invertebrate herbivores are controlled using nonpesticide techniques (e.g., cultural control), conventional chemical control, suppression by natural enemies, or more commonly all three. We therefore test an overall hypothesis that (i) in both organic and nonorganic croplands with active pest management, increasing plant diversity enhances top-down control by benefiting natural enemies more than herbivores, resulting in herbivore suppression and finally increasing crop production; and (ii) in grasslands and forests where invertebrate herbivores are rarely directly managed, increasing plant diversity will promote bottom-up effects by enhancing plant productivity, which may primarily although not exclusively benefit herbivore populations and, in turn, their natural enemies. This net effect will occur although both bottom-up and top-down interactions will operate simultaneously to some extent.

To test these hypotheses, we first calculated the ratio of enemy-to-herbivore abundances as a higher ratio was predicted to correlate with stronger top-down control (*42*). We calculated this ratio using data from the 120 studies that had paired observations of natural enemy abundance versus herbivore abundance (i.e., predator abundance versus herbivore abundance, and parasitoid abundance versus herbivore abundance) (data S2). Next, we compiled data from all the 149 studies and used Bayesian zero-or-one inflated beta regression models to examine correlations between *z*-score standardized plant diversity and the performances of plants, herbivores, and natural enemies (see Materials and Methods). We further applied piecewise structural equation models to test for direct and indirect biodiversity-mediated effects of plant diversity on plant performance (i.e., growth, reproduction, and quality), herbivore performance (i.e., abundance, herbivory damage, and diversity), and natural enemy performance (i.e., predator and parasitoid abundance, diversity, predation rate, and parasitism) (*21*, *22*, *38*). To evaluate the ecological causes and applied implications of these patterns, we analyzed variations across ecosystem types, plant life form, climatic region, and study types. Our findings show that trophic interactions are an additional way that plant diversity shapes ecosystem functioning. The direction of these interactions vary across ecosystems, driven by distinct top-down or bottom-up pathways. These results suggest that biodiversity contributes to ecosystem services via biocontrol of crop pests in croplands and via simultaneous gains in plant productivity and consumer biodiversity in grasslands and forests.

## RESULTS AND DISCUSSION

### Effect of plant diversity on abundances of herbivores and natural enemies

**Fig. 2.**
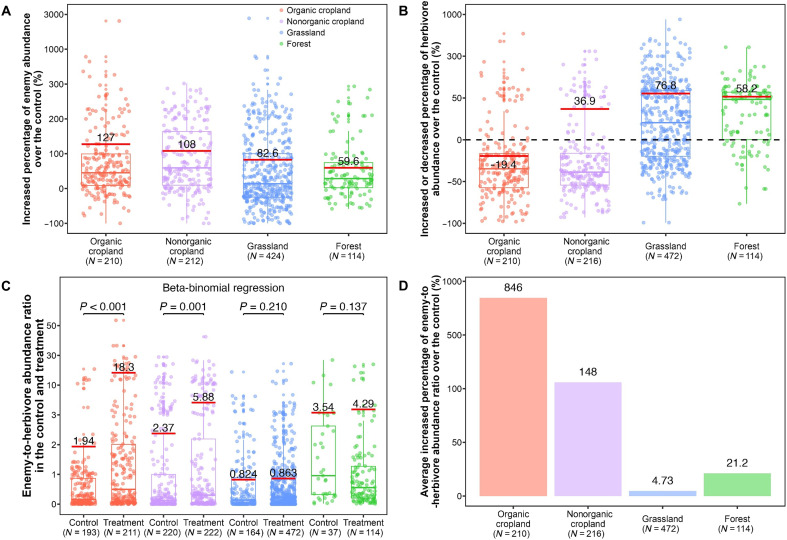
Effects of an increase in plant diversity on the abundances of invertebrate herbivores and their natural enemies and on enemy-to-herbivore abundance ratio. (**A**) Increased percentage of enemy abundance over the control (%). (**B**) Increased or decreased percentage of herbivore abundance over the control (%). (**C**) Enemy-to-herbivore abundance ratio in the control and treatment. (**D**) Average increased percentage of enemy-to-herbivore abundance ratio over the control (%). Natural enemy abundance includes the predator abundance and parasitoid abundance. The “Control” represents single, pure, or lowest plant species; and the “Treatment” represents the higher plant species richness (≥2). Enemy-to-herbivore abundance ratio = Enemy abundance ÷ herbivore abundance. Average increased percentage of enemy-to-herbivore abundance ratio over the control = (enemy-to-herbivore abundance ratio in treatment-enemy-to-herbivore abundance ratio in control) ÷ enemy-to-herbivore abundance ratio in control × 100%. There were 120 studies with paired 1641 observations of natural enemy abundance versus herbivore abundance (see data S2). In (A) to (C), lines within each box denote the median, outer borders of the boxes show the upper and lower quartile, lines outside of the boxes depict the maximum and minimum number of individuals, and red lines in each box represent the average value. In (A), (B), and (D), “*N*” denotes the number of observations in treatment group, whereas in (C), *N* is the number of observations in control group and treatment group across different ecosystems, respectively. In (C), we removed the eight observations with zero herbivore abundance because the enemy-to-herbivore abundance ratio cannot be calculated in such cases, and used the remaining 1633 observations from the total 1641 to calculate the ratio. NS, *P* > 0.05; *P < 0.05, **P < 0.01, ***P < 0.001.

The 120 studies with paired observations of natural enemy abundance versus herbivore abundance provided 1641 comparisons (data S2). Using these, we found that an increase in plant diversity was generally associated with greater enemy abundance (Fig. 2A) but differentially affected herbivores in organic and nonorganic croplands and in grasslands and forests (Fig. 2B). The ratio of enemy-to-herbivore abundance was significantly higher when plant species richness was ≥2 in both organic and nonorganic croplands (Fig. 2C). Crucially, the increase in plant diversity was associated with a strong and significant enhancement of the enemy-to-herbivore abundance ratio in both organic (+846%) and nonorganic croplands (+148%). The magnitude of this increase was markedly greater in croplands than the relatively modest and nonsignificant changes observed in grasslands (+4.73%) and forests (+21.2%) (Fig. 2D). This stark contrast indicates that plant diversity is linked to a much stronger relative shift toward higher natural enemy pressure in croplands. This pattern is consistent with a stronger increase in natural enemy relative to herbivore abundance in croplands, which could facilitate stronger top-down control, whereas in grasslands and forests, the increases in both groups were more proportional, suggesting a dynamic more consistent with bottom-up processes.

### Direct effect of plant diversity on different trophic groups

The data from all the 149 studies with 6604 observations showed that plant species richness was higher in grasslands and forests than in croplands [Fig. 1B; grasslands, mean = 9.55, IQR (interquartile range) = 4.00 to 10.8; forests, mean = 13.0, IQR = 3.00 to 22.0; organic croplands, mean = 4.66, IQR = 1.00 to 5.00; nonorganic croplands, mean = 2.61, IQR = 1.00 to 3.00]. Within these three systems, we assessed the direct and indirect effects of increasing plant species richness on overall measures of (i) plant performance, composed of plant growth, reproduction and quality (e.g., phenolics as chemical anti-herbivore defensive traits of plants, and sugars); (ii) herbivore performance, composed of invertebrate herbivore abundance, herbivory damage, and herbivore diversity; (iii) natural enemy performance, composed of invertebrate predator abundance, predation, predator diversity, parasitoid abundance, parasitoid diversity, and parasitism; and (vi) aggregate performance indicators for each of these three categories (*21*).

**Fig. 3.**
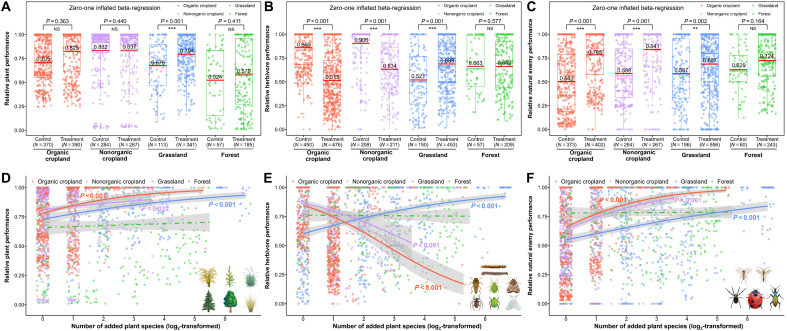
The effects of plant diversity on the tritrophic groups among global 149 field experiments. (**A**) Comparison of relative plant performance between the control and the treatment. (**B**) Comparison of relative invertebrate herbivore performance between the control and the treatment. (**C**) Comparison of relative natural enemy performance between the control and the treatment. (**D**) Relationships between the number of added plant species and relative plant performance. (**E**) Relationships between the number of added plant species and relative herbivore performance. (**F**) Relationships between the number of added plant species and relative natural enemy performance. Plant performance includes the growth, reproduction and quality of plants, herbivore performance includes the abundance, damage and diversity of herbivores, and natural enemy performance includes the predator abundance, predation, predator diversity, parasitoid abundance, parasitism, and parasitoid diversity. In (A) to (C) (see table S1), the Control represents single, pure or lowest plant species, the Treatment represents the higher plant species richness (≥2), the zero-or-one inflated beta regression was used to test whether the sets of values in two groups are significantly different (NS, *P* > 0.05; **P < 0.01, ***P < 0.001), lines within each box denote the median, outer borders of the boxes show the upper and lower quartile, lines outside of the boxes depict the maximum and minimum number of individuals, and red lines in each box represent the average value of relative performance of plants, herbivores or natural enemies in the control (i.e., single, pure or lowest plant species richness) and treatments (i.e., higher plant species richness, ≥2). In (D) to (F) with beta regressions (see table S5), solid lines show model predictions for significant (*P* < 0.05) effects, dashed lines show model predictions for nonsignificant (*P* > 0.05) relationships, the gray-shaded zone indicates the 95% CI. Photoshop8.0 was used to design the images.

Using zero-or-one inflated beta regression, we found that an increase in plant diversity (a binary variable that denotes any increase in the number of plant species in a plant community) enhances average relative plant performance by 17.1, 0.570, 17.6, and 10.5% across organic croplands, nonorganic croplands, grasslands, and forests, respectively, but only in grasslands this positive effect was significant [grasslands: confidence interval (CI) = 0.264 to 0.694, df = 452, *P* < 0.001; organic croplands: CI = −0.0910 to 0.248, df = 758, *P* = 0.363; nonorganic croplands: CI = −0.314 to 0.134, df = 569, *P* = 0.440; and forests: CI = −0.508 to 0.214, df = 240, *P* = 0.411] (Fig.3A and table S1). Likewise, an increase in plant diversity increased relative plant performance by 6.11 and 12.4% across temperate and tropical regions, by 11.3% in herbaceous plant–dominated systems (CI = 0.0100 to 0.258, df = 1302, *P* = 0.0320) (table S3), and by 8.10% in managed experiments (CI = −0.162 to 0.0710, df = 1771, *P* = 0.438) (table S4). However, only in herbaceous plant–dominated systems, this positive effect was significant (herbaceous: CI = 0.0100 to 0.258, df = 1302, *P* = 0.0320; temperate: CI = −0.170 to 0.0610, df = 1757, *P* = 0.385; tropical: CI = −0.157 to 0.490, df = 266, *P* = 0.305; managed experiments: CI = −0.162 to 0.0710, df = 1771, *P* = 0.438). Oppositely, we found that plant diversity significantly decreased relative plant performance by 1.46% in woody plants and nonsignificantly decreased relative plant performance by 2.92% in observational studies (woody: CI = −0.473 to −0.0609, df = 721, *P* = 0.0108; observational: CI = −0.396 to 0.277, df = 252, *P* = 0.755) (tables S2 to S4).

We found both positive and negative responses of plant performance to plant diversity across all ecosystems, climatic regions, plant types, and study types. When splitting the aggregate indicator of plant performance into plant growth, reproduction, and quality, we also found the variable responses to plant diversity across all ecosystems, climatic regions, plant types, and study types (Detailed analysis was seen in Supplementary Text, tables S1 to S4, and figs. S1 to S3). The fitted regression lines using beta regression showed that plant performance increased with the increase in the number of added plant species (a continuous variable with log_2_-transformation) across different ecosystems (Fig. 3D), in temperate regions (fig. S4A), for herbaceous plants (fig. S5A), and in managed experiments (fig. S6A). This positive biodiversity-productivity relationship is consistent with previous meta-analyses (*21*, *25*, *29*, *31*) or biodiversity experiments (*39*, *43*). There was a significantly positive response of herbivore performance to plant diversity in grasslands, but such response was not positively significant in forests (Fig. 3B; grasslands: +32.2%, CI = 0.342 to 0.737, df = 601, *P* < 0.001; forests: +0.280%, CI = −0.233 to 0.411, df = 266, *P* = 0.577). The positive herbivore response was likely due to high plant species richness increasing food availability (biomass, quality, and diversity) for herbivores. This has been previously reported for invertebrate herbivores in grassland (*6*, *10*, *32*) and forest biodiversity experiments (*33*, *35*–*37*). Overall, the responses of herbivores were more pronounced in grasslands than in forests (Fig. 3, B and E).

An increase in plant diversity increased the relative performance of natural enemies across ecosystems (organic: +56.0%, CI = 0.328 to 0.708, df = 773, *P* < 0.001; nonorganic: +42.9%, CI = 0.510 to 0.941, df = 531, *P* < 0.001; grasslands: +17.2%, CI = 0.119 to 0.509, df = 752, *P* = 0.002; forests: +15.0%, CI = −0.0840 to 0.464, df = 301, *P* = 0.164) (Fig. 3C and table S1). Likewise, the response of natural enemy performance to plant diversity was positive across different climatic regions (temperate: +34.6%, CI = 0.383 to 0.598, df = 2077, *P* < 0.001; tropical: +38.7%, CI = 0.275 to 0.877, df = 284, *P* < 0.001; table S2), plant types (herbaceous: +32.4%, CI = 0.331 to 0.579, df = 1668, *P* < 0.001; woody: +40.3%, CI = 0.386 to 0.701, df = 695, *P* < 0.001; table S3), and study types (managed: +38.7%, CI = 0.422 to 0.647, df = 2027, *P* < 0.001; observational: +8.95%, CI = −0.216 to 0.268, df = 336, *P* = 0.811; table S4). The positive response of natural enemies to increased plant species may be a product of both the mechanisms described under the EH (*22*, *23*) or by bottom-up effects of prey availability as they respond to increased productivity of the plant community (*6*, *10*, *21*). When we split the aggregate indicator of natural enemy performance into predator and parasitoid performances, we found variable responses to plant diversity across different ecosystems (fig. S7, A and B), climatic regions (fig. S1, D, E, I, and J), plant types (fig. S2, D, E, I, and J), and study types (fig. S3, D, E, I, and J). Similar responses were observed when natural enemy performance was classified into predator abundance, predation, predator diversity, parasitoid abundance, parasitism, and parasitoid diversity (tables S1 to S4). The positive relationships between natural enemy (Fig. 3F), predator (fig. S7C), or parasitoid (fig. S7D) performance and the number of added plant species were similar across different ecosystems, although relationships differed across climatic regions, plant types, and study types (table S5).

Of importance for enhancing biological control of crop pests via habitat manipulation or mixed cropping systems, higher plant diversity substantially decreased herbivore performance in croplands (organic: −40.1%, CI = −0.908 to −0.599, df = 924, *P* < 0.001; nonorganic: −30.0%, CI = −0.739 to −0.176, df = 417, *P* < 0.001) (Fig. 3B and table S1). These negative responses were variable across climatic regions, plant types, and study types (tables S1 to S4). This herbivore-suppressing effect in croplands may be explained by the EH, which predicts that natural enemy diversity is positively correlated with plant species diversity, resulting in lower herbivore levels in crop fields with higher plant species richness (*21*–*23*). The effects may also be a product of a greater chemical diversity in plant secondary metabolites suppressing herbivores or acting as cues for predators (*23*). It is also possible that the apparent decrease in herbivore numbers with plant diversity may be a product of monocultures supporting disproportionately high population of pest herbivores. This is predicted under the resource concentration hypothesis, where undiluted host visual or chemical cues combined with high local host density promote specialist pest herbivores (*22*, *23*).

### Effects of plant diversity on tritrophic interactions

**Fig. 4.**
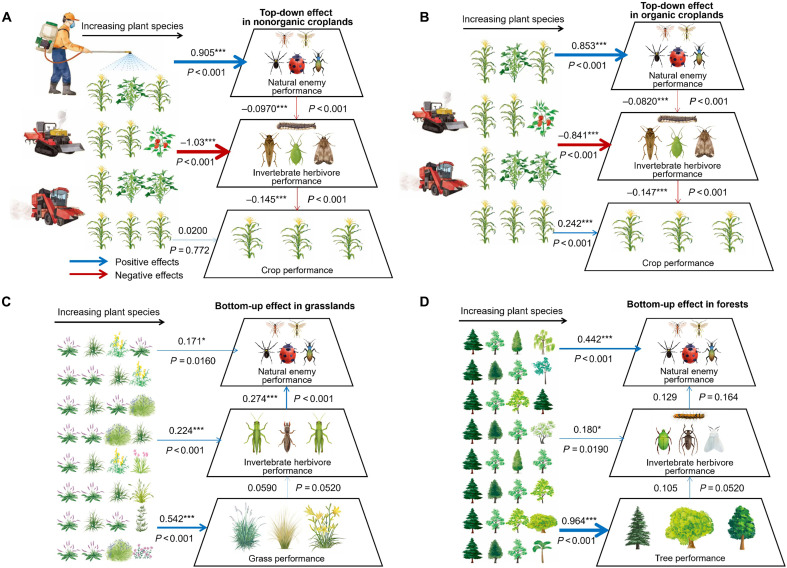
Structural equation model to test for an association between an increase in plant diversity and the top-down and bottom-up effects among plants, invertebrate herbivores, and their natural enemies across different ecosystems. (**A**) Top-down effect in nonorganic croplands (*N* = 887). (**B**) Top-down effect in organic croplands (*N* = 1189). (**C**) Bottom-up effect in grasslands (*N* = 832). (**D**) Bottom-up effect in forests (*N* = 333). Crop, grass, or forest performance includes the growth, reproduction, and quality of plants; herbivore performance includes the abundance, damage, and diversity of herbivores; and natural enemy performance includes the predator abundance, predation, predator diversity, parasitoid abundance, parasitism, and parasitoid diversity. *P < 0.05, ***P < 0.001. Blue and red arrows denote positive and negative relationships, respectively. Numbers next to each arrow are the estimated coefficients from structural equation models, and line width is proportional to the magnitude of the coefficients. Piecewise structural equation model (restricted maximum likelihood) was used to test the effects of plant diversity (measured as the number of plant species generalize to binary variable) on the tritrophic interactions of plants, invertebrate herbivores, and their natural enemies. There were 55, 49, 26, and 19 studies for organic croplands, nonorganic croplands, grasslands and forests, respectively (data S1 and S2). An increase in plant diversity was not associated with bottom-up effects in nonorganic croplands (*N* = 887) or in organic croplands (*N* = 1189), and was not associated with top-down effects in grasslands (*N* = 832) or in forests (*N* = 333) (see table S6). Photoshop8.0 was used to design the images.

To determine the directionality of the patterns observed above, we used structural equation models to test the direct and indirect effects of an increase in plant diversity (i.e., a binary variable as 0 and 1) on trophic groups and on both top-down and bottom-up effects. First, we analyzed the tritrophic interactions based on top-down effects, and we found that an increase in plant diversity in organic croplands (*N* = 1189) was positively associated with natural enemy performance (estimate = 0.85, CI = 0.760 to 0.945, *P* < 0.001) and crop performance (estimate = 0.242, CI = 0.145 to 0.340, *P* < 0.001), while it was negatively associated with herbivore performance (estimate = −0.841, CI = −0.200 to −0.0940, *P* < 0.001). We also detected a statistical pathway that is consistent with a top-down effect, mediated via a negative association between natural enemy and herbivore performance in nonorganic croplands (i.e., natural enemies versus herbivores: estimate = −0.0974, CI = −0.156 to −0.0389, *P* < 0.001; herbivores versus crops: estimate = −0.145, CI = −1.13 to −0.919, *P* < 0.001) (Fig. 4A). Similarly, an increase in plant diversity was associated with a pattern consistent with a top-down effect in organic croplands (i.e., natural enemies versus herbivores: estimate = −0.0822, CI = −0.135 to −0.0290, *P* < 0.001; herbivores versus crops: estimate = −0.147, CI = −0.939 to −0.743, *P* < 0.001) (Fig. 4B). However, in grasslands (*N* = 832) and forests (*N* = 333), an increase in plant diversity was not significantly associated with a top-down pattern (table S6). When natural enemies were divided into predators and parasitoids, respectively, we also found top-down effects in organic croplands for predators (fig. S8C) and in both organic and nonorganic croplands for parasitoids (fig. S9, A and C), but not in grasslands or forests (tables S16 and S17). The top-down cascading effects remained largely similar when data on the tritrophic interactions of natural enemies, herbivores, and crops in croplands were split across climatic regions, plant types, and study types (fig. S10 to S12 and table S7). Lower plant species richness in croplands may facilitate stronger top-down control, where small changes in diversity result in substantial changes in resources for predators, relative to what is seen in higher diversity grassland and forest systems where the base line occurrence of such resources may be relatively high (*22*, *23*, *38*). It is also likely that as soil nutrients due to intensified fertilization are typically high in croplands, niche complementarity (triggering bottom-up effects) effects common to grasslands and forests may have low importance (*44*).

Next, we tested bottom-up effects of an increase in plant diversity on tritrophic interactions. We found direct effects of increased plant richness on plant performance (estimate = 0.542, CI = 0.400 to 0.683, *P* < 0.001), herbivore performance (estimate = 0.224, CI = 0.2061 to 0.342, *P* < 0.001), and natural enemy performance (estimate = 0.171, CI = 0.0315 to 0.310, *P* = 0.0163) in grasslands (Fig. 4C), and in forests (plant performance: estimate = 0.964, CI = 0.734 to 1.19, *P* < 0.001; herbivore performance: estimate = 0.180, CI = 0.0215 to 0.237, *P* = 0.0188; natural enemy performance: estimate = 0.442, CI = 0.205 to 0.678, *P* < 0.001) (Fig. 4D). In addition, we detected statistical pathways consistent with a bottom-up effects in grasslands (i.e., plants versus herbivores: estimate = 0.0590, CI = −0.000500 to 0.119, *P* = 0.0519; herbivores versus natural enemies: estimate = 0.274, CI = 0.0958 to 0.352, *P* < 0.001) (Fig. 4C), and in forests (i.e., plants versus herbivores: estimate = 0.1051, CI = −0.000800 to 0.211, *P* = 0.0518; herbivores versus natural enemies: estimate = 0.129, CI = −0.0741 to 0.434, *P* = 0.164) (Fig. 4D). However, such plant diversity–mediated bottom-up effects were not observed in organic and nonorganic croplands (table S6). When natural enemies were split into predators and parasitoids, we also found bottom-up effects in grasslands (figs. S8F and S9F) and forests (figs. S8H and S9H), but not in croplands (tables S16 and S17). When all the data on the tritrophic interactions of natural enemies, herbivores, and plants in grasslands or forests were split across different climatic regions, different plant types, and different study types, the observed patterns were largely similar being characterized by bottom-up cascading effects (figs. S13 to S18 and tables S8 and S9). An increase in plant diversity triggered bottom-up effects in both managed and observational studies in grasslands (fig. S15). Likewise, focusing on the data in Cedar Creek and Jena grassland experiments, increased plant species richness was also shown to drive bottom-up effects (tables S6). As considered above, plant diversity–mediated bottom-up effects are likely linked to increased availability of floral resources in grasslands (*6*, *27*) (on average 9.55 grass species) and forests (*30*) (on average 12.95 tree species) in response to complementarity providing more resources for herbivores. The effects of plant species gradients on trophic interactions were presented in Supplementary Text, figs. S19 to S21, and data S4 to S6.

**Fig. 5.**
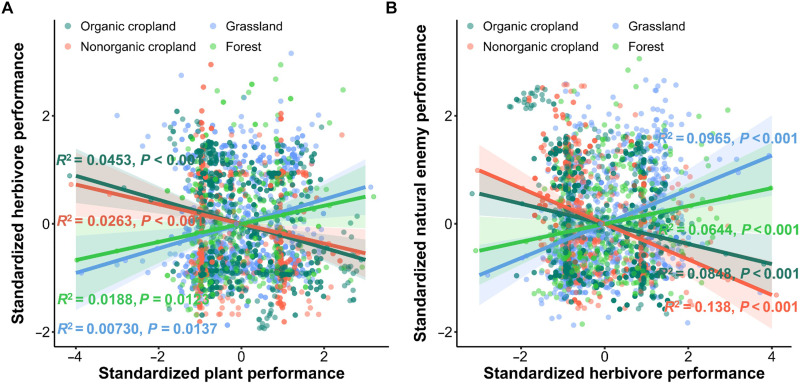
Predictions from linear mixed-effects models on the relationships in the interactions of plants, invertebrate herbivores, and their natural enemies. (**A**) Relationship between standardized plant performance and standardized herbivore performance in interaction with ecosystem categories (organic cropland versus nonorganic cropland versus grassland versus forest). (**B**) Relationship between standardized natural enemy performance and standardized herbivore performance in interaction with ecosystem categories (organic cropland versus nonorganic cropland versus grassland versus forest). The solid lines show fixed-effect predictions from mixed-effects models, the gray-shaded zone shows 95% CIs. The number of samples was *N* = 3241 from 149 experiments (see data S6 and S7).

To further test the directions of trophic interactions, we conducted linear regressions to investigate the relationships between standardized plant performance and standardized herbivore performance as well as between standardized herbivore performance and standardized natural enemy performance (data S6 and S7). Using mixed linear regressions to account for the random effect of experiment identity, we found that ecosystem type had a significant interaction with these correlations (Fig. 5 and data S6 and S7). This provides further evidence for our hypothesis of top-down control in organic and nonorganic croplands but bottom-up effects in both grasslands and forests. Specifically, the increase of plant performance boosted the increase of herbivore performance in both grasslands and forests while led to the decline of herbivore performance in croplands (Fig. 5A and data S6). To test whether interactive effects existed between each ecosystem type and the remaining other ecosystems, we made a contrast for each ecosystem type versus the remaining other ecosystems (i.e., nonorganic croplands versus organic croplands and grasslands and forests, organic croplands versus nonorganic croplands and grasslands and forests, grasslands versus nonorganic croplands and organic croplands and forests, and forests versus nonorganic croplands and organic croplands and grasslands). In terms of each tested ecosystem type, we found vivid interactive relationships between plant performance and herbivore performance (see data S6). Similar results were observed on the relationship between herbivore performance and natural enemy performance (Fig. 5B and data S7).

Across the 104 cropland studies, 49 used synthetic pesticides and 55 used organic technologies to suppress herbivores (data S1 and S2). Top-down control of herbivores is important in conventional agricultural systems, although it may play an even more prominent role in organic croplands with nonpesticide technologies (e.g., light, pheromone, or sticky traps). In conventional agricultural systems, integrated pest management strategies based on economic threshold levels can still provide benefits, as pesticide applications are more likely to be reduced when natural pest control is effective (*45*). Cultural approaches (e.g., optimized sowing schedules and adjusted harvesting periods), combined with mechanized tillage operations, were also used to suppress herbivores by damaging their habitats and refuges or creating phenological mismatch between the crop and their lifecycle (*22*). As invertebrate herbivores mediate between top-down and bottom-up effects, their abundance and diversity as affected by these practices may also determine the direction of cascading effects. This may explain why, for grasslands and forests that were included in this study, the absence of herbivore-suppressing technologies or management practices led to a prevalence of bottom-up effects when compared to croplands. Here, higher herbivore abundances in the absence of anthropogenic control measures may simply overwhelm top-down control by predators. In contrast, the use of herbivore-suppressing technologies in croplands may make top-down predatory effects more likely as herbivore abundances remain low enough for these to happen.

An important consideration when interpreting our results is the nature of the underlying data. As noted earlier, our global synthesis relies on a variety of metrics (e.g., abundance, damage, and diversity measures for herbivore performance). These metrics provide robust evidence for systematic correlations and biomass redistribution patterns along plant diversity gradients. While these patterns are strongly consistent with the theoretical frameworks of top-down and bottom-up effects, they represent correlations rather than direct, experimental quantification of trophic interactions. Future experiments that combine plant diversity manipulations with direct measurements of predation, parasitism, and herbivory rates will be needed to move from correlation to causal demonstration of these mechanisms. While empirical work involving predator/natural enemy exclusion would be ideal for definitively testing this hypothesis, such data are rare in current research. Given the incompleteness of existing data, we used structural equation modeling to examine correlational patterns between plant diversity, herbivore performance, and predator performance. Our findings therefore support but do not conclusively prove causal trophic cascades.

A key consideration in interpreting our reported effect sizes (e.g., percentage changes) is our operational use of a binary plant diversity factor. These estimates represent the average relative change when transitioning from low- to higher-diversity states across diverse studies. While this approach validly captures the overarching direction and ecosystem-specific contrasts, the precise numerical values should be interpreted as indicative of strong, systematic trends rather than as universal constants. The consistency of these trends is reinforced by supplementary analyses using continuous richness measures, which support the same qualitative conclusions (see Figs. 3, D to F and 4; figs. S4 to S6, S7, C and D, and S19 to S24; and tables S5, S10 to S13, S15, S18, and S19). We also reported the analysis with standardized richness gradients to quantify the exact shape of these relationships (see Supplementary Text).

Overall, our multiecosystem analysis reveals that an increase in plant diversity generally enhances plant productivity and benefits natural enemies of invertebrate herbivores in the four main terrestrial ecosystems (i.e., organic and nonorganic croplands, grasslands, and forests), with statistically significant effects on plant performance only in grasslands, and with significant effects on natural enemy performance in organic and nonorganic croplands, and grasslands. Our findings indicate that the effects of plant diversity on plant productivity are not solely explained by direct plant-plant interactions or the classic biodiversity-productivity relationship. Instead, they reveal correlation patterns additionally consistent with top-down and bottom-up effects, the prevalence of which may vary depending on ecosystem context and management goals. However, the observed top-down process in croplands suggests that greater plant diversity may support predator populations through mechanisms beyond simply providing more herbivore prey. In contrast, for grasslands and forests, the primary effect of plant diversity appears to be an increase in plant availability, leading to greater herbivore population size. Predators then respond to this increased herbivore abundance but potentially after significant herbivory. For conservation-focused management of grasslands and forests, these bottom-up effects may contribute to increasing plant productivity and consumer diversity. Conversely, in intensively managed croplands with chronic pest pressure, strategic plant diversification may reinforce top-down control via natural enemy augmentation, thereby preemptively suppressing herbivore populations and helping to protect crop yield. The role of diversifying crop system plant diversity is therefore an important pathway to explore when trying to better achieve sustainable food production.

## MATERIALS AND METHODS

### Data compilation

This study relied on the collection of data from studies where plant-invertebrate herbivore-invertebrate natural enemy (hereafter plant-herbivore-enemy) interactions had been quantified in response to an increase in plant diversity, either directly manipulated under experimental conditions or part of existing natural variation in study sites. We obtained the raw data from two compilation efforts. First, we identified studies by searching the reference lists of recent meta-analyses that reported on plant diversity–mediated effects on different trophic groups (*21*, *36*, *46*–*49*). Second, we launched a data request to researchers within the field of multitrophic interactions, starting in June 2020 and running until May 2025. In both cases, we requested that raw data be provided within a designed datasheet (see data S1 and description below). This datasheet allowed for standardized documentation of plant species diversity effects on the tritrophic interactions among plants, invertebrate herbivores, and their natural enemies (i.e., predators and/or parasitoids). We did not consider bitrophic interactions (plant-herbivore, plant-enemy, or herbivore-enemy interactions). Of ∼3800 researchers initially contacted, authors of 149 of the above experiments meeting our criteria provided data. In collecting data, we found that some authors offered data from multiple experiments and that some experiments belonged to multiple authors (see data S2). A subset of the experiments associated with croplands included the use of pesticides, although for most studies (including all data from grasslands and forests), no pesticides were used. The use of pesticides and/or other pest control methods was the same for the control (single/lowest plant species) and the treatment (diverse plant species) to exclude the effects of pest control methods on trophic interactions. Nonpesticide control methods (e.g., cultural control methods) are also used. In croplands, nonpesticide-based herbivore control methods included pest-resistant crop varieties, removing crop stubble or ploughing to destroy pest overwintering sites, clipping buds at flowering to physically remove aphids, spreading ash, crop rotation and even using irrigation to suppress pests in rice. Nonsynthetic pesticides were also used included sulphur as well as physically catching pests using sticky traps and other attractants (light and pheromone) and direct manipulation of natural pest control (e.g. releasing parasitic *Trichogramma* spp. wasps). In all cases, these no-pesticide control methods were consistently applied across all relevant treatments originating from a single study within our final analysis. Overall, we analyzed data from 149 experiments comprising 214 sites in 27 countries i 5 continents (data S2).

As stated above, we standardized data collected by providing all data providers with four database sheets (data S1). These were (i) data for plant diversity effects on trophic groups; (ii) structural equation modeling data of plant diversity effects on tritrophic interactions of plants, herbivores, and natural enemies; (iii) structural equation modeling data of plant diversity effects on tritrophic interactions of plants, herbivores, and parasitoids; and (iv) structural equation modeling data of plant diversity effects on tritrophic interactions of plants, herbivores, and predators. In each database sheet, plant performance was measured as plant growth, plant reproduction and plant quality, invertebrate herbivore performance consisted of invertebrate herbivore growth, herbivory damage, and herbivore diversity in plants, natural enemy performance composed of predator abundance, predation, predator diversity, parasitoid abundance, parasitoid diversity, and parasitism. Natural enemy performance was split into invertebrate predator performance and invertebrate parasitoid performance, in which the former performance measure was composed of predator abundance, predation and predator diversity, and the latter performance composed of parasitoid abundance, parasitism, and parasitoid diversity (*21*).

### Description of predictor variables

Trophic groups: Trophic groups included plant performance, invertebrate herbivore performance, and natural enemy performance (i.e., predator performance and parasitoid performance).

#### Plant performance

This represents the primary producer metric used in the assessment of tritrophic interactions. Measures of plant performance included plant growth, reproduction, and quality. Plant growth was assessed as growth rate and size characteristics (e.g., weight, height, biomass, dry matter, stem length, wood volume, width, and density). Plant reproduction included the yield of grains, fruits, flowers, pods, reproductive plant parts and seeds per unit, and reproductive traits measured on individual plants (e.g., flower production, pod production, and grain number per spike). Plant quality included content of chemical antiherbivore defensive traits of plants (e.g., total phenolics and polyphenols), sugars, and amino acids. The synthesis combined these diverse metrics to estimate the overall effect size of tritrophic interactions on plant performance, with the understanding that different aspects of plant performance may respond differently to ecological interactions.

#### Invertebrate herbivore performance

This integrated performance metric synthesizes three ecologically interdependent dimensions: herbivore abundance (population density, e.g., individuals/unit area) reflects colonization pressure, herbivory damage (e.g., percentage or number of herbivore damage to plants, or as loss of plant yield or biomass due to herbivores) quantifies plant response, and herbivore diversity (e.g., herbivore species richness and Shannon’s diversity index) captures community structure. This combination addresses three key ecological mechanisms (i) abundance-driven immediate impacts, (ii) diversity-mediated functional redundancy, and (iii) damage thresholds that trigger plant defenses. While synthesis analysis requires standardized metrics, their integration provides a robust proxy for ecosystem function—navigating the balance between necessary simplification (which enables cross-system comparisons) and the preservation of ecological context (e.g., accounting for differences between tropical and temperate systems).

#### Invertebrate predator performance

This integrated performance metric combines predator abundance (population density, e.g., individuals/unit area), predation (prey consumption rate, e.g., % prey killed), and predator diversity (e.g., predator species richness and Shannon’s diversity index) into a single indicator to capture trophic interactions holistically. This integration is scientifically justified because (i) abundance drives immediate prey suppression, (ii) diversity enhances functional redundancy (e.g., niche partitioning), and (iii) predation rates link predator activity to ecosystem stability. While synthesis analysis requires standardized metrics, their aggregation provides a robust measure of top-down control—balancing the need for comparability with ecological complexity (e.g., habitat-specific variability).

#### Invertebrate parasitoid performance

This integrated performance metric combines parasitoid abundance (population density, e.g., individuals/unit area), parasitism (e.g., percentage or number of hosts that are parasitized by parasitoids), and parasitoid diversity (e.g., parasitoid species richness and Shannon’s diversity index) into a single indicator to comprehensively assess biological control efficacy. This integration is ecologically meaningful because (i) abundance determines immediate host suppression capacity, (ii) parasitism directly measures trophic efficiency, and (iii) diversity enhances ecosystem stability through niche complementarity. While synthesis analysis requires standardized metrics, this aggregation provides a robust framework for evaluating parasitoid-mediated population regulation—effectively balancing the need for comparative synthesis with the preservation of ecosystem-specific dynamics (e.g., agricultural versus natural habitats).

#### Natural enemy performance

This integrated assessment combines invertebrate predator performance (measuring predator abundance, predation, and predator diversity) with invertebrate parasitoid performance (quantifying parasitoid abundance, parasitism, and parasitoid diversity), creating a comprehensive metric for evaluating top-down population regulation and bottom-up effects. To enhance transparency, we listed all original performance metrics (e.g., species-level abundance per plot, Shannon diversity indices) for each study in Figshare (https://doi.org/10.6084/m9.figshare.31947189), which enables cross-referencing each integrated performance index back to the specific raw data from each of the 149 included studies. Herbivore, predator, and parasitoid abundance metrics were synthesized by standardizing heterogeneous measurements across studies [e.g., counts per unit area (individuals per square meter), per plant (individuals per 20 plants), and per soil sample (individuals per gram dry weight)]. In forests, predator biomass data (grams per square meter) were available from two studies, while one grassland study reported parasitoid biomass per plot (gram per plot). These invertebrate biomass metrics were incorporated into the composite abundance index after confirming their strong correlation with count-based measures [as it has been proved that insect abundance was positively correlated with insect biomass (*50*)]. This methodological approach addressed the limitation of insufficient sample size for separate invertebrate biomass analyses.

Ecosystem type: We categorized the ecosystems into croplands, grasslands, and forests. However, we define croplands to include harvestable food crop associated with annual and perennial crop systems under arable or horticultural management, with this including ornamental plant farming systems, as well as longer term perennial systems such as orchards or biomass crops. We applied categorizations for grasslands as defined by authors of individual studies. Forest systems were in general dominated by woody plants under the minimum of long-term rotational systems. Other ecosystems such as wetlands, shrublands, and arid ecosystems were not found and thus were excluded in this study.

Plant life form: To distinguish this from ecosystem types, we used a categorical variable reflecting whether the terrestrial plants are herbaceous or woody (*21*). Cotton, fruits (e.g., apple and pear) and tea are crops in croplands but were assigned to woody plants. Some herbaceous plants (e.g., wheat, mustard, tomato, and pepper) are found in croplands, while some herbaceous plants (e.g., grasses, legumes, and small and tall herbs) are in grasslands.

Type of experimental study: Here, we defined “managed experiments” that the authors manage the plant species richness in field experiments directly, while “observational experiment” denotes that the authors do not manage plant species richness in field experiments directly and only use readily available land (e.g., cultivating plant species from natural vegetation or farmers’ planting) for survey and sampling. For example, the authors used the farmers’ fields in which plant species richness in cropping fields was managed by farmers themselves instead of the authors; we considered this as observational experiment. In general, there was only one replicate for each observational experiment with multiple locations/sites in natural grasslands and forests. We defined and confirmed type of experimental study by contacting and discussing with the authors who provided us data directly.

Climatic region type is a categorical variable reflecting whether a certain study was performed in the temperate or tropical zones. Temperate zones ranged from 23.5° to 66.5°N and from 23.5° to 66.5°S, and the tropical zones ranged from 0° to 23.5°N and from 0° to 23.5°S. Data from greenhouse and indoor experiments were removed from models considering climatic predictors (*21*).

Number of added plant species is a continuous variable describing the increase in plant species richness between the control (monocultures of the lowest experimental species richness of plants) and the treatment containing an increased number of plant species richness relative to the control. When we compared the plant species richness of the control (i.e., pure, mono, or lowest plant species) with that of the treatment (i.e., higher plant species richness, ≥2 plant species richness), we confirmed that both the control and treatment were compared on the same trophic groups.

### Analysis for proposed hypothesis

To test our hypothesis, we derived a new dataset including paired natural enemy abundance and invertebrate herbivore abundance in each experiment. In total, among all the 149 studies, we extracted the data of 120 studies with paired 1641 observations of natural enemy abundance versus herbivore abundance (data S2). First, we tested the increased percentage of natural enemy abundance and herbivore abundance in four ecosystem types, respectively (Fig. 2, A and B). For example, the increased percentage of enemy abundance follows (enemy abundance in treatment − enemy abundance in control) ÷ enemy abundance in control × 100%; the treatment group indicates the higher number of plant species, whereas the control group indicates the lowest number of plant species. Furthermore, we tested whether the ratio of natural enemy abundance to herbivore abundance differs in diverse plant communities (i.e., high plant species richness, set as treatment “Treatment”) and simple plant communities or monocultures (i.e., low species richness, set as control “Control”) [not significant (NS) *P* > 0.05; *P < 0.05; **P < 0.01; ***P < 0.001], we conducted analysis using beta-binomial regression (*51*), which can accommodate ratio data. The “glmmTMB()” function with parameter “family = betabinomial()” in the glmmTMB (version 4.3.1) library in R statistical environment was used to fit a beta-binomial regression model using maximal likelihood estimation. We removed the eight observations with zero abundance of herbivores as the enemy-to-herbivore abundance ratio was invalid when the herbivore abundance is zero. Namely, we used 1633 of the 1641 observations to calculate the enemy-to-herbivore abundance ratio. Meanwhile, we analyzed the enemy-to-herbivore abundance ratio in the control and treatment (Fig. 2C) and then and calculated the average increased percentage of enemy-to-herbivore abundance ratio over the control (%) (Fig. 2D) as follows: enemy-to-herbivore abundance ratio = enemy abundance ÷ herbivore abundance. Increased percentage of enemy-to-herbivore abundance ratio = (enemy-to-herbivore abundance ratio in treatment-enemy-to-herbivore abundance ratio in control) ÷ enemy-to-herbivore abundance ratio in control × 100%.

### Definition of an increase in plant diversity and number of added plant species

To assess the general effects of plant diversity across a globally heterogeneous dataset, we first treated plant diversity as a binary variable (zero or one), comparing control plots (monocultures or the lowest richness level in a given study) to treatment plots with increased plant diversity (≥2 species) (*49*). This approach standardizes comparisons across studies with vastly different maximum richness levels and experimental designs, allowing us to test the fundamental question of whether introducing plant diversity yields consistent directional effects. A binary plant diversity was used in the context of the analysis as a descriptor of within-study relative differences in plant diversity that allows standardization across the diversity of studies where there are differences in experimental design, plot sizes, management regimes, and functional characteristics of the community, e.g., between croplands, grasslands, and woodlands. We acknowledge that this simplification does not capture potential nonlinear effects across a full richness gradient. Therefore, we complemented this primary analysis with models using plant species richness as a continuous variable (log_2_-transformed) where sample size permitted. Results for “an increase in plant diversity” are shown in Fig. 3, A to C; figs. S7, A and B, S8 and S9, S1 to S3 and S7 to S15; and tables S1 to S4, S6 to S9, S14, S16, and S17. Results for “number of added plant species” are shown in Figs. 3, D to F, and 4; figs. S4 to S6, S7, C and D, and S19 to S24; and tables S5, S10 to S13, S15, S18, and S19.

### Definition of relative performance and data standardization

We assessed the effects of binary plant diversity effects (low versus high) as well as the number of added plant species on plant performance, herbivore performance, and natural enemy performance. Because of differences in the units of indicators used to measure different trophic groups, we had to standardize plant performance (i.e., plant growth, plant reproduction, and plant quality), herbivore performance (herbivore abundance, herbivory damage, and herbivore diversity), natural enemy performance (i.e., predator abundance, predation, predator diversity, parasitoid abundance, parasitoid diversity, and parasitism), predator performance (predator abundance, predation, and predator diversity), and parasitoid performance (i.e., parasitoid abundance, parasitoid diversity, and parasitism). This was done by using the standardized performance (i.e., relative performance) index

$$\text{Relative performance}=\frac{{\text{Performance}}_{i}}{\text{max}\left(\text{Performance}\right)}$$ (1)

where Performance is a vector consisting of all performance values in each replication in each experiment from each unit of each indicator in each experimental year, ${\text{Performance}}_{i}$ is the actual value in the ith element of Performance, and max(Performance) is the max value in each experiment from each unit of each indicator in each experimental year. Namely, ${\text{Performance}}_{i}$ and max(Performance) should be matched in the same unit, in the same experiment, and in the same year. Thus, the value of relative performance ranges from 0 to 1.

As data were extracted from experiments that used different units of measurements, we also standardized the response value (plant, herbivore, and natural enemy performances) using *z* scores. This was done within each experiment and among different experimental years and replicate orders (*48*)

$$\text{Standardized performance}=\frac{\text{Performance}-\mu }{\sigma }$$ (2)

where μ,σ are the mean value and SD of performances within each experiment among different experimental years and replicate orders, respectively. This standardization allows for comparisons between studies with differences in methodology and plot range (*52*). The *z-*score standardization reduces the risk of misinterpreting results due to studies differing in methodology and number of plots (*53*). To test our hypothesis, we conducted simple linear regressions and mixed linear regressions to investigate the relationships between standardized plant performance and standardized herbivore performance as well as between standardized herbivore performance and standardized natural enemy performance (see data S6 and S7).

### Differential analysis of binary plant diversity effects

To examine whether the relative performance of plants, herbivores, and natural enemies differed between diverse plant communities (i.e., high plant species richness, set as treatment “TR”) and simple plant communities or monocultures (i.e., low species richness, set as control “CK”) (NS, *P* > 0.05; *P < 0.05; **P < 0.01; ***P < 0.001), we ran differential analyses using zero-or-one inflated beta regressions (*54*), which can accommodate boundary inflations at zero-or-one of the response variable (i.e., relative performance). We then analyzed the relationship between various binary plant diversity groups (CK is considered as zero and TR as one), as shown in Fig. 3 (A to C) and fig. S7 (A and B). Since the relative performance is proportional data (i.e., zero-one), the response is often one in our data, which leads to the one-inflation. To improve the precision of estimation, the “brm()” function in the brms library (*55*) in R statistical environment was used to construct zero-or-one inflated beta regressions. Only the case of one-inflation was allowed, as implemented using setting “family = zero_one_inflated_beta,” “zoi ∼ 1,” “coi ∼ Plant.diversity” in the call to brm. Then, we can obtain statistic values, 95% CI and *P* values from summary results of model.

### Beta regression analysis of the number of added plant species

To examine whether the number of added plant species had additional explanatory variance on the relative performance of each trophic group, we ran further analyses using the logarithm of the number of plant species added [e.g., log_2_ (1, 2, 3, 4, 5, …)] in the plant diversity treatment over the control as an explanatory variable and using the relative value of performance (i.e., plant performance, herbivore performance, predator performance, parasitoid performance, and natural enemy performance, that is, all of combined predator and parasitoid performance) as the response variable. Then, we analyzed the relationships between the number of added plant species and the different relative performances of trophic levels using beta-regression (Figs. 3, D to F, and 4; figs. S7, C and D, and S4 to S6; and tables S5, S10 to 13, 15, 18, and 19). In these regression analyses, we took the number of added plant species as a predictor and relative performance as response following the equation

$$\text{Relative performance}=\frac{e^{\beta_{0}+\beta_{1}\times {\text{log}}_{2}\left(\text{The number of added plant species}\right)}}{1+e^{\beta_{0}+\beta_{1}\times {\text{log}}_{2}\left(\text{The number of added plant species}\right)}}$$ (3)

where $\beta_{0}$ is the intercept, $\beta_{1}$ is the slop between the number of added plant species and relative performance, and ε is the random error. The glmmTMB() function in the glmmTMB (version 4.3.1) library in R statistical environment was used to fit a beta regression model using maximal likelihood estimation (*56*). We used the R function “confint()” in the “stats” library to calculate 95% CI of the parameters estimated.

### Structural equation modeling for binary plant diversity and number of added plant species

To explore the bottom-up and top-down effects of binary plant diversity on the tritrophic interactions between plants, invertebrate herbivores and their natural enemies, we used subsets of our data. These were (i) paired tritrophic observations (i.e., for all natural enemies, for predators and for parasitoids) (see tables S6, S16, and S17); (ii) different ecosystem types (croplands, grasslands, and forests) (Fig. 4 and table S6), being divided by plant performance versus herbivore performance versus predator performance, and plant performance versus herbivore performance versus parasitoid performance, separately for croplands, grasslands and forests (figs. S8 and S9 and tables S16 and S17); (iii) different climatic regions, plant types, and study types (figs. S10 to S18 and table S14); (iv) and comprising different climatic regions, plant types, and study types separated by each ecosystem (tables S7 to S9). Meanwhile, to investigate the effects of varying levels of plant species richness, we carried out structural equation modeling to test the direct and indirect effects of the number of added plant species on the above tritrophic interactions across different ecosystems, climatic regions, plant types, and study types, respectively (tables S10 to S13, S15, S18, and S19). Specifically, we used structural equation modeling to understand the relative effect of (i) increasing plant species richness on the performance of the overall plant community, (ii) the indirect effect of plant performance resulting from the response by the herbivore performance to increasing plant species richness (top-down effect), (iii) the indirect effect of herbivore performance resulting from the response by the natural enemy performance to increasing plant species richness (top-down effect), (iv) the indirect effect of natural enemy performance resulting from the response by the herbivore performance to increasing plant species richness (bottom-up effect), and (v) the indirect effect of herbivore performance resulting from the response by the plant performance to increasing plant species richness (bottom-up effect). Likewise, we considered the number of added plant species as predictor and conducted the same analyses as the above binary plant diversity. Plant performance, herbivore performance, and natural enemy performance were derived from these pairwise datasets.

To analyze the direct and indirect effect of binary plant diversity and the number of added plant species, we fitted piecewise structural equation models to each of the data subsets. In this analysis, we used the standardized values of varying performance as the response variable. Specifically, the direct effects of binary plant diversity or the number of added plant species on varying performance was estimated using linear mixed-effects models

1) Top-down effect [directional effects describing: plant diversity → secondary consumers (i.e., natural enemy performance, predator performance, and parasitoid performance) → primary consumers (i.e., invertebrate herbivore performance) → primary producers (i.e., plant performance)]

$$\begin{array}{c} \text{Natural enemy performance in tritrophic interaction}=\beta_{0}+\beta_{1}\\ \times {\text{log}}_{2}\left(T\text{he number of added plant species}\right)+r_{i}+w_{\mathrm{ij}}+\varepsilon_{\mathrm{ij}} \end{array}$$ (4)

$$\begin{array}{c} \text{Herbivore performance in tritrophic interaction}=\beta_{0}+\beta_{2}\\ \times N\text{atural enemy performance in tritrophic interaction}+\beta_{3}\\ \times {\text{log}}_{2}\left(T\text{he number of added plant species}\right)+r_{i}+w_{\mathrm{ij}}+\varepsilon_{\mathrm{ij}} \end{array}$$ (5)

$$\begin{array}{c} \text{Plan}t\;\text{performance in tritrophic interaction}=\beta_{0}+\beta_{4}\\ \times \;H\text{erbivore performance in tritrophic interaction}+\beta_{5}\\ \times {\text{log}}_{2}\left(T\text{he number of added plant species}\right)+r_{i}+w_{\mathrm{ij}}+\varepsilon_{\mathrm{ij}} \end{array}$$ (6)

2) Bottom-up effect [directional effects describing: plant diversity → primary producers (i.e., plant performance) → primary consumer (i.e., invertebrate herbivore performance) → secondary consumers (i.e., natural enemy performance, predator performance, and parasitoid performance]

$$\begin{array}{c} \text{Plant performance in tritrophic interaction}=\beta_{0}+\beta_{1}\\ \times {\text{log}}_{2}\left(T\text{he number of added plant species}\right)+r_{i}+w_{\mathrm{ij}}+\varepsilon_{\mathrm{ij}} \end{array}$$ (7)

$$\begin{array}{c} \text{Herbivore performance in tritrophic interaction}=\beta_{0}\;+\beta_{2}\;\\ \times \;P\text{lant performance in tritrophic interaction}+\beta_{3}\\ \times \;{\text{log}}_{2}\left(T\text{he number of added plant species}\right)+r_{i}+w_{\mathrm{ij}}+\varepsilon_{\mathrm{ij}} \end{array}$$ (8)

$$\begin{array}{c} \text{Natural enemy performance in tritrophic interaction}=\beta_{0}\;+\beta_{4}\;\\ \times \;H\text{erbivore performance in tritrophic interaction}+\beta_{5}\\ \times \;{\text{log}}_{2}\left(T\text{he number of added plant species}\right)+r_{i}+w_{\mathrm{ij}}+\varepsilon_{\mathrm{ij}} \end{array}$$ (9)

where $\beta_{0}$ is the intercept of each linear mixed-effects model; $\beta_{1},\beta_{3},\;\text{and}\;\beta_{5}$ are the estimated effect size of the logarithm of the number of added plant species on relative performance (i.e., plant performance, herbivore performance, and natural enemy performance); $\beta_{2}\;\text{and}\;\beta_{4}$ are the effect of plant/natural enemy performance on herbivore performance and herbivore performance on plant/natural enemy performance, respectively; $r_{i}$ represents the random intercept in ith study; $w_{\mathrm{ij}}$ is the random intercept of the jth observation in ith study; and $\varepsilon_{\mathrm{ij}}$ is the random error term. In top-down effect modeling, Eq. 4 models the relationship between the number of added plant species and natural enemy performance, Eq. 5 models the relationship between the number of added plant species and herbivore and natural enemy performance, and Eq. 6 models the relationship between the number of added plant species and plant and herbivore performance. In bottom-up effect modeling, Eq. 7 models the relationship between the number of added plant species and plant performance, Eq. 8 models the relationship between the number of added plant species and herbivore and plant performance, Eq. 9 models the relationship between the number of added plant species and natural enemy and herbivore performance. The detailed models follows below

i) Binary plant diversity/number of added plant species − Natural enemy performance − Herbivore performance in tritrophic interactions (top-down effect) (tables S6 and S10)

Natural enemy performance in tritrophic interactions=

$$\begin{array}{c} \beta_{0}+\beta_{1}\times {\text{log}}_{2}\left(\text{The number of added plant species}\right)+r_{i}+w_{\mathrm{ij}}+\varepsilon_{\mathrm{ij}} \end{array}$$

Herbivore performance in tritrophic interactions=

$$\begin{array}{c} \beta_{0}+\beta_{2}\times \text{Natural enemy performance in tritrophic interactions}+\beta_{3}\\ \times {\text{log}}_{2}\left(\text{The number of added plant species}\right)+r_{i}+w_{\mathrm{ij}}+\varepsilon_{\mathrm{ij}} \end{array}$$

ii) Binary plant diversity/number of added plant species – Herbivore performance – Plant performance in tri-trophic interactions (top-down effect) (tables S6 and S10)

Herbivore performance in tritrophic interactions=

$$\begin{array}{c} \beta_{0}+\beta_{1}\times {\text{log}}_{2}\left(\text{The number of added plant species}\right)+r_{i}+w_{\mathrm{ij}}+\varepsilon_{\mathrm{ij}} \end{array}$$

$\beta_{0}+\beta_{2}\times \text{Herbivore performance in tritrophic interactions}+$

iii) Binary plant diversity/number of added plant species – Natural enemy performance − Herbivore performance – Plant performance in tri-trophic interactions (top-down effect) (tables S6 and S10)

Natural enemy performance in tritrophic interactions=

$$\begin{array}{c} \beta_{0}+\beta_{1}\times {\text{log}}_{2}\left(\text{The number of added plant species}\right)+r_{i}+w_{\mathrm{ij}}+\varepsilon_{\mathrm{ij}} \end{array}$$

Herbivore performance in tritrophic interactions=

$$\begin{array}{c} \beta_{0}+\beta_{2}\times \text{Natural enemy performance in tritrophic interactions}+\\ \beta_{3}\times {\text{log}}_{2}\left(\text{The number of added plant species}\right)+r_{i}+w_{\mathrm{ij}}+\varepsilon_{\mathrm{ij}} \end{array}$$

Plant performance in tri−trophic interactions=

$$\begin{array}{c} \beta_{0}+\beta_{4}\times \text{Herbivore performance in tritrophic interactions}+\beta_{5}\times \\ {\text{log}}_{2}\left(\text{The number of added plant species}\right)+r_{i}+w_{\mathrm{ij}}+\varepsilon_{\mathrm{ij}} \end{array}$$

iv) Binary plant diversity/number of added plant species – Plant performance – Herbivore performance in tritrophic interactions (bottom-up effect) (tables S6 and S10)

Plant performance in tritrophic interactions=

$$\begin{array}{c} \beta_{0}+\beta_{1}\times {\text{log}}_{2}\left(\text{The number of added plant species}\right)+r_{i}+w_{\mathrm{ij}}+\varepsilon_{\mathrm{ij}} \end{array}$$

Herbivore performance lnRR=

$$\begin{array}{c} \beta_{0}+\beta_{2}\times \text{Plant performance in tritrophic interactions}+\beta_{3}\times \\ {\text{log}}_{2}\left(\text{The number of added plant species}\right)+r_{i}+w_{\mathrm{ij}}+\varepsilon_{\mathrm{ij}} \end{array}$$

v) Binary plant diversity/number of added plant species – herbivore performance – Natural enemy performance in tritrophic interactions (bottom-up effect) (tables S6 and S10)

Herbivore performance in tritrophic interactions=

$$\begin{array}{c} \beta_{0}+\beta_{1}\times {\text{log}}_{2}\left(\text{The number of added plant species}\right)+r_{i}+w_{\mathrm{ij}}+\varepsilon_{\mathrm{ij}} \end{array}$$

Natural enemy performance in tritrophic interactions=

$$\begin{array}{c} \beta_{0}+\beta_{2}\times \text{Herbivore performance in tritrophic interactions}+\beta_{3}\times \\ {\text{log}}_{2}\left(\text{The number of added plant species}\right)+r_{i}+w_{\mathrm{ij}}+\varepsilon_{\mathrm{ij}} \end{array}$$

vi) Binary plant diversity/number of added plant species – Plant performance − Herbivore performance – Natural enemy performance in tritrophic interactions (bottom-up effect) (tables S6 and S10)

Plant performance in tritrophic interactions=

$$\begin{array}{c} \beta_{0}+\beta_{1}\times {\text{log}}_{2}\;\left(\text{The number of added plant species}\right)+r_{i}+w_{\mathrm{ij}}+\varepsilon_{\mathrm{ij}} \end{array}$$

Herbivore performance in tritrophic interactions=

$$\begin{array}{c} \beta_{0}+\beta_{2}\times \text{Plant performance in tritrophic interactions}+\beta_{3}\times \\ {\text{log}}_{2}\left(\text{The number of added plant species}\right)+r_{i}+w_{\mathrm{ij}}+\varepsilon_{\mathrm{ij}} \end{array}$$

Natural enemy performance in tritrophic interactions=

$$\begin{array}{c} \beta_{0}+\beta_{4}\times \text{Herbivore performance in tritrophic interactions}+\beta_{5}\times \\ {\text{log}}_{2}\left(\text{The number of added plant species}\right)+r_{i}+w_{\mathrm{ij}}+\varepsilon_{\mathrm{ij}} \end{array}$$

vii) Binary plant diversity/number of added plant species – Predator performance − Herbivore performance – Plant performance in tritrophic interactions (top-down effect) (tables S16 and S18)

Predator performance in tritrophic interactions=

$$\begin{array}{c} \beta_{0}+\beta_{1}\times {\text{log}}_{2}\left(\text{The number of added plant species}\right)+r_{i}+w_{\mathrm{ij}}+\varepsilon_{\mathrm{ij}} \end{array}$$

Herbivore performance in tritrophic interactions=

$$\begin{array}{c} \beta_{0}+\beta_{2}\times \text{Predator performance in tritrophic interactions}+\beta_{3}\times \\ {\text{log}}_{2}\left(\text{The number of added plant species}\right)+r_{i}+w_{\mathrm{ij}}+\varepsilon_{\mathrm{ij}} \end{array}$$

Plant performance in tritrophic interactions=

$$\begin{array}{c} \beta_{0}+\beta_{4}\times \text{Herbivore performance in tritrophic interactions}+\beta_{5}\times \\ {\text{log}}_{2}\left(\text{The number of added plant species}\right)+r_{i}+w_{\mathrm{ij}}+\varepsilon_{\mathrm{ij}} \end{array}$$

viii) Binary plant diversity/number of added plant species – Parasitoid performance − Herbivore performance – Plant performance in tritrophic interactions (top-down effect) (tables S17 and S19)

Parasitoid performance in tritrophic interactions=

$$\begin{array}{c} \beta_{0}+\beta_{1}\times {\text{log}}_{2}\left(\text{The number of added plant species}\right)+r_{i}+w_{\mathrm{ij}}+\varepsilon_{\mathrm{ij}} \end{array}$$

Herbivore performance in tritrophic interactions=

$$\begin{array}{c} \beta_{0}+\beta_{2}\times \text{Parasitoid performance in tritrophic interactions}+\beta_{3}\times \\ {\text{log}}_{2}\left(\text{The number of added plant species}\right)+r_{i}+w_{\mathrm{ij}}+\varepsilon_{\mathrm{ij}} \end{array}$$

Plant performance in tri−trophic interactions=

$$\begin{array}{c} \beta_{0}+\beta_{4}\times \text{Herbivore performance in tritrophic interactions}+\beta_{5}\times \\ {\text{log}}_{2}\left(\text{The number of added plant species}\right)+r_{i}+w_{\mathrm{ij}}+\varepsilon_{\mathrm{ij}} \end{array}$$

ix) Binary plant diversity/number of added plant species – Natural enemy performance – Herbivore performance in croplands, grasslands, forests respectively in tritrophic interactions (top-down effect) (Fig. 4 and tables S6 and S10)

Natural enemy performance in different ecosystems=

$$\begin{array}{c} \beta_{0}+\beta_{1}\times {\text{log}}_{2}\left(\text{The number of added plant species}\right)+r_{i}+w_{\mathrm{ij}}+\varepsilon_{\mathrm{ij}} \end{array}$$

Herbivore performance in different ecosystems=

$$\begin{array}{c} \beta_{0}+\beta_{2}\times \text{Natural enemy performance in different ecosystems}+\\ \beta_{3}\times {\text{log}}_{2}\left(\text{The number of added plant species}\right)+r_{i}+w_{\mathrm{ij}}+\varepsilon_{\mathrm{ij}} \end{array}$$

x) Binary plant diversity/number of added plant species – Herbivore performance – Plant performance in croplands, grasslands, forests, respectively, in tritrophic interactions (top-down effect) (Fig. 4 and tables S6 and S10)

Herbivore performance in different ecosystems=

$$\begin{array}{c} \beta_{0}+\beta_{1}\times {\text{log}}_{2}\left(\text{The number of added plant species}\right)+r_{i}+w_{\mathrm{ij}}+\varepsilon_{\mathrm{ij}} \end{array}$$

Plant performance in different ecosystems=

$$\begin{array}{c} \beta_{0}+\beta_{2}\times \text{Herbivore performance in different ecosystems}+\beta_{3}\times \\ {\text{log}}_{2}\left(\text{The number of added plant species}\right)+r_{i}+w_{\mathrm{ij}}+\varepsilon_{\mathrm{ij}} \end{array}$$

xi) Binary plant diversity/number of added plant species – Natural enemy performance – Herbivore performance – Plant performance in croplands, grasslands, forests, respectively in tritrophic interactions (top-down effect) (Fig. 4 and tables S6 and S10)

Natural enemy performance in different ecosystems=

$$\begin{array}{c} \beta_{0}+\beta_{1}\times {\text{log}}_{2}\left(\text{The number of added plant species}\right)+r_{i}+w_{\mathrm{ij}}+\varepsilon_{\mathrm{ij}} \end{array}$$

Herbivore performance in different ecosystems=

$$\begin{array}{c} \begin{array}{c} \beta_{0}+\beta_{2}\times \text{Natural enemy performance in different ecosystems}+\beta_{3}\times \\ {\text{log}}_{2}\left(\text{The number of added plant species}\right)+r_{i}+w_{\mathrm{ij}}+\varepsilon_{\mathrm{ij}} \end{array} \end{array}$$

Plant performance in different ecosystems=

$$\begin{array}{c} \beta_{0}+\beta_{4}\times \text{Herbivore performance in different ecosystems}+\beta_{5}\times \\ {\text{log}}_{2}\left(\text{The number of added plant species}\right)+r_{i}+w_{\mathrm{ij}}+\varepsilon_{\mathrm{ij}} \end{array}$$

xii) Binary plant diversity/number of added plant species – Natural enemy performance – Herbivore performance in herbaceous plants, woody plants, managed studies, observational studies, temperate climatic zones, and tropical climatic zones among different ecosystem types, respectively, in tritrophic interactions (top-down effect) (figs. S10 to S18 and tables S7 to S9 and S11 to S15)

Natural enemy performance in different subgroups=

$$\begin{array}{c} \beta_{0}+\beta_{1}\times {\text{log}}_{2}\left(\text{The number of added plant species}\right)+r_{i}+w_{\mathrm{ij}}+\varepsilon_{\mathrm{ij}} \end{array}$$

Herbivore performance in different subgroups=

$$\begin{array}{c} \beta_{0}+\beta_{2}\times \text{Natural enemy performance in different subgroups}+\beta_{3}\times \\ {\text{log}}_{2}\left(\text{The number of added plant species}\right)+r_{i}+w_{\mathrm{ij}}+\varepsilon_{\mathrm{ij}} \end{array}$$

xiii) Binary plant diversity/number of added plant species – Herbivore performance – Plant performance in herbaceous plants, woody plants, managed studies, observational studies, temperate climatic zones, and tropical climatic zones among different ecosystem types, respectively, in tritrophic interactions (top-down effect) (figs. S10 to S18 and tables S7 to S9 and S11 to S15)

Herbivore performance in different subgroups=

$$\begin{array}{c} \beta_{0}+\beta_{1}\times {\text{log}}_{2}\left(\text{The number of added plant species}\right)+r_{i}+w_{\mathrm{ij}}+\varepsilon_{\mathrm{ij}} \end{array}$$

Plant performance in different subgroups=

$$\begin{array}{c} \beta_{0}+\beta_{2}\times \text{Herbivore performance in different subgroups}+\beta_{3}\times \\ {\text{log}}_{2}\left(\text{The number of added plant species}\right)+r_{i}+w_{\mathrm{ij}}+\varepsilon_{\mathrm{ij}} \end{array}$$

xiv) Binary plant diversity/number of added plant species – Natural enemy performance – Herbivore performance in herbaceous plants, woody plants, managed studies, observational studies, temperate climatic zones, and tropical climatic zones among different ecosystem types respectively in tri-trophic interactions (top-down effect) (figs. S10 to 18 and tables S7 to S9 and S11 to S15)

Natural enemy performance in different subgroups=

$$\begin{array}{c} \beta_{0}+\beta_{1}\times {\text{log}}_{2}\left(\text{The number of added plant species}\right)+r_{i}+w_{\mathrm{ij}}+\varepsilon_{\mathrm{ij}} \end{array}$$

Herbivore performance in different subgroups=

$$\begin{array}{c} \beta_{0}+\beta_{2}\times \text{Natural enemy performance in different subgroups}+\beta_{3}\times \\ {\text{log}}_{2}\left(\text{The number of added plant species}\right)+r_{i}+w_{\mathrm{ij}}+\varepsilon_{\mathrm{ij}} \end{array}$$

Plant performance in different subgroups=

$$\begin{array}{c} \beta_{0}+\beta_{4}\times \text{Herbivore performance in different subgroups}+\beta_{5}\times \\ {\text{log}}_{2}\left(\text{The number of added plant species}\right)+r_{i}+w_{\mathrm{ij}}+\varepsilon_{\mathrm{ij}} \end{array}$$

In the analysis of top-down effects, we used mediation analysis to test whether the effect of an independent variable D (i.e., plant diversity) on a dependent variable Y (i.e., herbivore performance) (D → Y) is at least partly explained via the inclusion of a mediator variable X (i.e., secondary consumers including natural enemy performance, predator performance, and parasitoid performance) (D → X → Y). Meanwhile, we also test whether the effect of an independent variable D on a dependent variable Z (i.e., plant performance) is at least partly explained via the inclusion of a mediator variable Y (D → Y → Z). Similarly, in analysis of bottom-up effects, the effect of an independent variable D′ (i.e., plant diversity) on a dependent variable X′ (i.e., secondary consumers including natural enemy performance, predator performance, and parasitoid performance) (D′ → X′) is at least partly explained via the inclusion of a mediator variable Y′ (i.e., herbivore performance) (D′ → Y′ → X′), and the effect of an independent variable D′ on a dependent variable Y′ is at least partly explained via the inclusion of a mediator variable Z′ (i.e., plant performance) (D′ → Z′ → Y′). Likewise, mediation analysis of both top-down and bottom-up effects were adopted to analyze the effects of the number of added plant species (i.e., a continuous variable describing the increase in plant species richness) on trophic groups and their interactions (see fig. S25). In our top-down effect, the five causal paths e1, e2, e3, e4, and e5 correspond to the effect of plant diversity on secondary consumer performance, the effect of secondary consumer performance on herbivore performance, the effect of plant diversity on herbivore performance accounting for secondary consumer performance, the effect of herbivore performance on plant performance, and the effect of plant diversity on plant performance accounting for herbivore performance, respectively. The five causal paths correspond to parameters from three regression models, one in which secondary consumer performance is the outcome and plant diversity is the predictor, one in which herbivore performance is the outcome and plant diversity and secondary consumer performance are the simultaneous predictors, one in which plant performance is the outcome and herbivore performance is the predictors and one in which plant performance is the outcome and plant diversity and herbivore performance are the simultaneous predictors. From these parameters, we can calculate the mediation effect (the product e2 × e3 and e3 × e4) and total effect (e2 × e3 + e1) of plant diversity on herbivore performance and total effect (e3 × e4 + e5) of plant performance on plant performance. In our bottom-up effect, the five causal paths e1′, e2′, e3′, e4′, and e5′ correspond to the effect of plant diversity on secondary consumer performance, the effect of herbivore performance on secondary consumer performance, the effect of plant diversity on secondary consumer performance accounting for herbivore performance, the effect of plant performance on herbivore performance, and the effect of plant diversity on herbivore performance accounting for plant performance respectively. The five causal paths correspond to parameters from three regression models, one in which secondary consumer performance is the outcome and herbivore performance is the predictor, one in which secondary consumer performance is the outcome and plant diversity and herbivore performance are the simultaneous predictors, one in which plant performance is the outcome and plant diversity is the predictors, and one in which herbivore performance is the outcome and plant diversity and plant performance are the simultaneous predictors. From these parameters, we can calculate the mediation effect (the product e1′ × e2′ and e4′ × e5′) and total effect (e1′ × e2′ + e1′) of plant diversity on secondary consumer performance and total effect (e4′ × e5′ + e3′) of plant diversity on herbivore performance. Likewise, mediation analysis of both top-down and bottom-up effects were adopted to analyze the effects of the number of added plant species (i.e., a continuous variable describing the increase in plant species richness) on trophic groups and their interactions. It is possible that the action of predators can influence soil nutrient cycling via nutrient redistribution from excretion or carcasses (*19*, *20*, *57*). However, the original studies that were included in our analysis were not designed to explicitly test this process, and so this potential effect has not been considered in the structural equation models (SEMs). In addition, it is well established that plant structure can directly affect predator abundance by providing architectural complexity leading to habitat or niche diversification (*19*, *20*, *57*). Yet, as few of the original studies in our analysis directly assessed plant structural components, we are unable to analyze this further. However, plant diversity is linked to, and to an extent inseparable from, overall plant community architectural complexity. Its impacts on predator abundance and ecosystem service delivery are thus captured in our analysis, albeit not necessarily explicitly.

Our SEM path analyses were fitted with “psem” function in library “piecewiseSEM” (*58*), using series of linear mixed-effects models with random intercepts for (i) experimental order of studies, area of experimental site, experimental year, units of target species or trophic group performance indicators, and replicate order for target species or trophic group performance in both control and treatment for grasslands and forests; and (ii) experimental order of studies, area of experimental site, experimental year, units of target species or trophic group performance indicators, replicate order for target species or trophic group performance in both control and treatment and plant diversity practices (i.e., intercropping, cover cropping, and sown field margins) for croplands. Here, experimental order of studies refers to the 149 experiments from order 1 to order 149 (the sequence of the experiments was determined on the basis of the time when we got the data from the authors who provided us data). Replicate order for target species or trophic group performance in both control and treatment refers to the number of replicates in a certain experiment. Specifically, we extracted the *t* values and its corresponding coefficient to test the effects of binary plant diversity on the tritrophic interactions of plant, herbivore, and natural enemy performances (table S6). Furthermore, we tested the effects of binary plant diversity on the tritrophic interactions of plants, herbivore, and natural enemy (i.e., predators and parasitoids) performances (figs. S8 and S9 and tables S16 and S17). Likewise, we tested the effects of plant species gradients on top-down and bottom-up effects in croplands, grasslands, and forests (figs. S19 to S21 and data S3 to S5). The estimations and test statistics were extracted using the “summary()” function in R on piecewise equation SEM models.
