# Supplementary material for: Plant diversity modifies multi-trophic interactions in croplands, grasslands and forests
Source: Sci Adv. 2026 Jul 23;12(30):eaeb8680. doi: 10.1126/sciadv.aeb8680 (PMC13394405; doi:10.1126/sciadv.aeb8680)
Supplement: Supplementary file 1 — Supplementary Text Figs. S1 to S25 Tables S1 to S19 Legends for data S1 to S7 [file sciadv.aeb8680_sm.pdf]

Science Advances
AAAS

Supplementary Materials for

Plant diversity modifies multi-trophic interactions in croplands, grasslands and forests

Nian-Feng Wan *et al.*

Corresponding author: Nian-Feng Wan, nfwan@ecust.edu.cn, fnianwan_2004@163.com

Sci. Adv. **12**, eaeb8680 (2026)
DOI: 10.1126/sciadv.aeb8680

The PDF file includes:

Supplementary Text
Figs. S1 to S25
Tables S1 to S19
Legends for data S1 to S7

Other Supplementary Material for this manuscript includes the following:

Data S1 to S7

Supplementary Text

Pest management or control methods

Chemical Control: This primarily refers to the use of synthetic pesticides (e.g., insecticides) to directly kill or suppress invertebrate herbivores (pests) and other target organisms. In the context of non-organic croplands within our study, this is a key management practice.

Cultural Control: This involves modifying agricultural practices or the crop environment to make it less suitable for pests, thereby suppressing populations indirectly. Examples from the analyzed studies included optimizing sowing schedules, adjusting harvesting periods, employing pest-resistant crop varieties, and crop rotation. The goal is often to disrupt the pest's life cycle, habitat, or the pest-host plant synchrony, creating a phenological mismatch. The manuscript states: "Cultural approaches (e.g., optimized sowing schedules, and adjusted harvesting periods), combined with mechanized tillage operations, were also used to suppress herbivores by damaging their habitats, refuges or creating phenological mismatch between the crop and their lifecycle".

Physical Control: This encompasses non-chemical, direct mechanical or physical interventions to remove, kill, or exclude pests. Examples documented in the data provided by the contributing authors included manual or mechanical removal of pests (e.g., clipping buds to physically remove aphids), the use of traps (e.g., sticky traps, light traps, pheromone traps for mass trapping or monitoring), spreading substances like ash, and using irrigation specifically to suppress pests (e.g., in rice fields). These methods provide an alternative to synthetic pesticides, especially in organic farming systems.

The key distinction is: chemical control acts directly on the pest's physiology, whereas cultural and physical controls manipulate the agroecosystem or use physical means to achieve suppression. In our dataset and analysis, both organic (often relying on cultural, physical, and biological controls) and non-organic (often involving synthetic pesticides alongside other tactics) cropping systems implement "pest management through pesticides and/or pesticide alternative technologies." Our finding that top-down trophic cascades are strengthened by plant diversity in both organic and non-organic croplands suggests that diverse plant communities can enhance biological control effectiveness across this spectrum of management practices.

Effects of plant diversity on trophic groups

Across all studies, we found that the responses of natural enemy performance, predator performance and parasitoid performance to plant diversity were significantly positive (enemy: CI=0.385 to 0.583, df=2361, $P<0.001$; predator: CI=0.308 to 0.555, df=1427, $P<0.001$; parasitoid: CI=0.459 to 0.763, df=932, $P<0.001$), the response of invertebrate herbivore performance to plant diversity was significantly negative (CI=−0.279 to −0.062, df=2212, P=0.002), and the response of plant performance was not significant (CI=−0.164 to 0.058, df=2025, P=0.359) (table S1).

The response of plant performance to plant diversity was not significant in organic croplands (CI=−0.091 to 0.248, df=758, P=0.363), non-organic croplands (CI=−0.314 to 0.134, df=569, P=0.440), or in forests (CI=−0.508 to 0.214, df=240, P=0.411), while such response was significantly positive in grasslands (CI=0.264 to 0.694, df=452, $P<0.001$) (table S1). In both temperate and tropical regions, and both managed and observational studies, the responses of plant performance to plant diversity were not

significant (temperate: CI=−0.170 to 0.061, df=1757, P=0.385; tropical: CI=−0.157 to 0.490, df=266, P=0.305; managed: CI=−0.162 to 0.071, df=1771, P=0.438; observational: CI=−0.396 to 0.277, df=252, P=0.755). Across plant life forms, we found that the response of plant performance was significantly positive for herbaceous plants (CI=0.010 to 0.258, df=1302, P=0.032) but significantly negative for woody plants (CI=−0.473 to −0.061, df=721, P<0.001). When plant performance was divided into plant growth, reproduction and quality, plant diversity only significantly affected plant quality in organic croplands (CI=0.441 to 1.412, df=16, P<0.001), plant growth in grasslands (CI=0.288 to 0.735, df=384, P<0.001), plant growth for herbaceous plants (CI=0.153 to 0.542, df=548, P<0.001), and plant growth for woody plants (CI=−0.622 to −0.140, df=397, P=0.002) (tables S1–S4).

In both organic and non-organic croplands, plant diversity significantly decreased herbivore performance (organic croplands: CI=−9.579 to −0.599, df=924, P<0.001; non-organic croplands: CI=−0.739 to −0.176, df=417, P<0.001), while plant diversity significantly increased herbivore performance in grasslands (CI=0.343 to 0.737, df=601, P<0.001) but not in forests (CI=−0.233 to 0.411, df=264, P=0.577). In temperate regions and managed studies, there were significantly negative effects (temperate: CI=−0.283 to −0.064, df=1977, P=0.002; managed: CI=−0.306 to −0.071, df=1914, P=0.001), and such significantly negative effect also occurred for herbaceous plants (CI=−0.298 to −0.057, df=1677, P=0.004). However, such effect was not significant in tropical regions (CI=−1.192 to 0.097, df=233, P=0.109), for woody plants (CI=−0.477 to 0.026, df=533, P=0.075), or in observational studies (CI=−0.461 to 0.178, df=296, P=0.392). When herbivore performance was divided into herbivore abundance, herbivore damage and herbivore diversity, we found that: i) across ecosystems, plant diversity had significantly negative effects on herbivore abundance and damage in organic croplands (abundance: CI=−0.754 to −0.366, df=589, P<0.001; damage: CI=−1.386 to −0.924, df=333, P<0.001), and herbivore abundance in non-organic croplands (CI=−0.824 to −0.170, df=359, P<0.001), while had significantly positive effects on herbivore abundance, damage and diversity in grasslands (abundance: CI=0.222 to 0.677, df=456, P<0.001; damage: CI=0.580 to 1.759, df=78, P<0.001; diversity: CI=0.341 to 1.320, df=63, P=0.001); ii) across climatic regions, plant diversity had significantly negative effects on herbivore damage (CI=−0.798 to −0.345, df=419, P<0.001) but significantly positive effects on herbivore diversity (CI=0.091 to 0.729, df=169, P=0.012) in organic croplands; and iii) across plant life forms, plant diversity had significantly negative effects on herbivore damage (CI=−0.790 to −0.308, df=418, P<0.001) for herbaceous plants, and on herbivore abundance for woody plants, but had significantly positive effects on herbivore diversity (CI=0.061 to 0.939, df=87, P=0.031) for herbaceous plants; and iv) in managed studies, the responses of herbivore abundance and damage to plant diversity was significantly negative (abundance: CI=−0.277 to −0.006, df=1352, P=0.048; damage: CI=−0.745 to −0.271, df=19, P<0.001), while the response of herbivore diversity was significantly positive (CI=0.178 to 0.862, df=126, P=0.004) (tables S1–S4).

Across ecosystems, plant diversity had significantly positive effects on natural enemy, predator and parasitoid performances in both croplands and non-croplands (enemy in croplands: CI=0.328 to 0.703, df=773, P<0.001; predator in croplands: CI=0.277 to 0.834, df=374, P<0.001; parasitoid in croplands: CI=0.521 to 0.960, df=397, P<0.001; enemy in non-croplands: CI=0.510 to 0.941, df=531, P<0.001; predator in non-croplands:

CI=0.271 to 0.801, df=371, P<0.001; parasitoid in non-croplands: CI=0.760 to 1.584, df=158, P<0.001). Meanwhile, plant diversity had significantly positive effects on natural enemy performance (CI=0.119 to 0.509, df=750, P=0.002), and predator performance (CI=0.128 to 0.611, df=496, P=0.002), but not on parasitoids performance (CI=−0.125 to 0.527, df=252, P=0.212) in grasslands. Among the natural enemy, predator, and parasitoids performances in forests, only the response of predator performance was significantly positive (predator: CI=0.172 to 0.779, df=180, P=0.002; enemy: CI=−0.084 to 0.464, df=301, P=0.164; parasitoid: CI=−0.413 to 0.504, df=119, P=0.818). In two regions, plant diversity had significantly positive effects on natural enemy and predator performances in both temperate and tropical regions (enemy in temperate: CI=0.383 to 0.578, df=2077, P<0.001; enemy in tropical: CI=0.275 to 0.877, df=531, P<0.001; predator in temperate: CI=0.251 to 0.507, df=1272, P<0.001; predator in tropical: CI=0.453 to 1.425, df=153, P<0.001), while the response of parasitoid performance was significantly positive in temperate regions (CI=0.494 to 0.824, df=803, P<0.001), but was not significant in tropical regions (CI=−0.151 to 0.621, df=11, P=0.243). For both herbaceous and woody plants, these responses were also significantly positive (enemy for herbaceous: CI=0.331 to 0.579, df=1666, P<0.001; predator for herbaceous: CI=0.131 to 0.439, df=982, P<0.001; parasitoid for herbaceous: CI=0.475 to 0.838, df=682, P<0.001; enemy for woody: CI=0.386 to 0.701, df=693, P<0.001; predator for woody: CI=0.543 to 0.933, df=443, P<0.001; parasitoid for woody: CI=0.119 to 0.608, df=248, P=0.004). In managed studies, these responses were significantly positive (enemy: CI=0.422 to 0.647, df=2025, P<0.001; predator: CI=0.347 to 0.618, df=1213, P<0.001; parasitoid: CI=0.478 to 0.812, df=810, P<0.001), but not significant in observational studies (enemy: CI=−0.216 to 0.268, df=334, P=0.811; predator: CI=−0.176 to 0.417, df=212, P=0.406; parasitoid: CI=−0.479 to 0.366, df=120, P=0.794) (tables S1–S4).

When splitting the aggregate indicator of predator performance into predator abundance, predation and predator diversity, and parasitoid performance into parasitoid abundance, parasitism and parasitoid diversity, we found the variable responses. In organic croplands, only the responses of predator abundance and parasitism were significantly positive (predator abundance: CI=0.272 to 0.870, df=342, P<0.001; parasitism: CI=0.894 to 1.340, df=262, P<0.001), and in non-organic croplands, only the responses of predator abundance and parasitoid abundance were significantly positive (predator abundance: CI=0.356 to 0.911, df=312, P<0.001; parasitoid abundance: CI=0.816 to 1.701, df=124, P<0.001). In grasslands, the responses of predator abundance and parasitoid diversity were significantly positive (predator abundance: CI=0.103 to 0.611, df=431, P=0.008; parasitoid diversity: CI=0.272 to 1.482, df=48, P=0.007), while in forests, only the response of predator abundance was significantly positive (CI=0.007 to 0.904, df=96, P=0.049). Among the six metrics, we found that: i) the responses of four metrics were significantly positive in temperate regions (predator abundance: CI=0.243 to 0.521, df=100, P=0.851; predation: CI=−0.731 to 0.880, df=14, P<0.001; predator diversity: CI=−0.041 to 0.549, df=17, P=0.077; parasitoid abundance: CI=0.039 to 0.503, df=31, P=0.024; parasitoid diversity: CI=0.295 to 1.228, df=72, P=0.002; parasitism: CI=0.719 to 1.155, df=308, P<0.001), while only the response of predator abundance was significantly positive in tropical regions (CI=0.423 to 1.417, df=20, P<0.001); ii) the responses of three metrics were significantly positive for herbaceous plants (predator abundance: CI=0.132 to 0.476, df=834, P<0.001; predation: CI=−1.135 to 0.865, df=35,

P=0.806; predator diversity: CI=−0.211 to 0.594, df=109, P=0.322; parasitoid abundance: CI=−0.226 to 0.366, df=334, P=0.627; parasitoid diversity: CI=0.262 to 1.489, df=48, P=0.007; parasitism: CI=0.754 to 1.227, df=296, P<0.001), and the responses of two metrics were significantly positive for woody plants (predator abundance: CI=0.500 to 0.954, df=351, P<0.001; predation: CI=−0.456 to 1.536, df=10, P=0.427; predator diversity: CI=−0.279 to 0.598, df=78, P=0.472; parasitoid abundance: CI=0.231 to 0.831, df=172, P<0.001; parasitoid diversity: CI=−0.414 to 1.085, df=33, P=0.363; parasitism: CI=−1.701 to 0.292, df=39, P=0.179); and iii) the responses of four metrics were significantly positive in managed studies (predator abundance: CI=0.355 to 0.649, df=1039, P<0.001; predation: CI=−1.389 to 1.148, df=24, P=0.795; predator diversity: CI=0.036 to 0.675, df=146, P=0.027; parasitoid abundance: CI=−0.010 to 0.452, df=452, P=0.059; parasitoid diversity: CI=0.309 to 1.269, df=72, P=0.002; parasitism: CI=0.722 to 1.193, df=282, P<0.001), while the responses of all metrics were not significant in observational studies (predator abundance: CI=−0.348 to 0.349, df=33, P=0.993; predation: CI=−1.678 to 1.335, df=21, P=0.848; predator diversity: CI=−0.382 to 1.458, df=41, P=0.249; parasitoid abundance: CI=−0.389 to 0.796, df=54, P=0.495; parasitoid diversity: CI=−1.776 to 2.542, df=9, P=0.776; parasitism: CI=−1.031 to 0.467, df=53, P=0.479) (tables S1–S4).

Effects of plant species gradients on trophic interactions

Then we tested the effects of plant species gradients (i.e., each plant species gradient in each experiment=higher plant species－lowest plant species) on top-down and bottom-up interactions. For example, in Jena biodiversity experiments, there are five number of plant species (i.e., 1, 4, 8, 16 and 60), and the plant species gradients are 3, 7, 15 and 59. The results indicated that i) five levels of plant species gradients (i.e., 1, 4, 5, 10 and 36) trigger top-down interactions in croplands (fig. S19), but all the levels of plant species gradients did not trigger bottom-up interactions in croplands (Data S3); and ii) three levels of plant species gradients (i.e., 2, 3 and 15) result in bottom-up interactions in grasslands (fig. S20), four gradients (i.e., 1, 3, 5 and 6) result in bottom-up interactions in forests (fig. S21), but all the levels of plant species gradients did not trigger top-down interactions in grasslands (Data S4) or forests (Data S5). When croplands were classified into organic and non-organic croplands, we found that three levels of plant species gradients (i.e., 1, 10 and 36) trigger top-down interactions in organic croplands (fig. S19), and two levels of plant species gradients (i.e., 1 and 5) trigger top-down interactions in non-organic croplands (Data S3).

Detailed Acknowledgements

We would like to thank Guadalupe Peralta, Carol M. Frost, David A. Wedin, Amanda L. Buchanan, Cerruti R. R. Hooks, Carmen K. Blubaugh, Lionel Hertzog and Xiaoyu Ning for providing us valuable data.

Anne Ebeling was supported by German Science Foundation (DFG) Research Unit FOR 1451; Nico Eisenhauer and Nico Eisenhauer acknowledge funding by the German Research Foundation (DFG, FZT 118, 202548816; FOR 1451; FOR 5000; Ei 862/29-1).

Hervé Jactel was supported by European Union Project "PHOCUS" (grant agreement CT97-3440).

Xiaofei Li was supported by the National Natural Science Foundation of China (No. 32471624).
Zhiwei Zhong was supported by the National Natural Science Foundation of China (No. 32371587).
Andreas Schuldt was supported by German Science Foundation (DFG) Research Unit for 891.
Margaret Kosmala was supported by a US National Science Foundation Graduate Research Fellowship (00006595) and a Carol H. and Wayne A. Pletcher Graduate Fellowship; research was made possible by the US National Science Foundation Long-Term Ecological Research Program (DEB-0620652; DEB-1234162).
Ji-Liang Liu was funded by the National Natural Science Foundation of China (Grand No. 41771290), Gansu Province Top-notch Leading Talents Project (E339040101), and the Key Research and Development Program of Gansu Province (25YFNA053).
This work was supported by grants from the US National Science Foundation Long-Term Ecological Research Program (LTER) including DEB-0620652, DEB-1234162 and DEB-1831944. Further support was provided by the Cedar Creek Ecosystem Science Reserve and the University of Minnesota.
Nico Eisenhauer and Simone Cesarz were support by the German Research Foundation (DFG, FZT 118, 202548816; FOR 1451; FOR 5000; Ei 862/29-1).
Peter B. Reich was supported by the Department of Energy (DOE/DE-FG02-96ER62291) and the National Science Foundation (NSF Biocomplexity 0322057, NSF LTER DEB 9411972 (1994-2000), DEB 0080382 (2000-2006), and DEB 0620652 (2006-2012), and NSF LTREB 0716587).
Stine Kramer Jacobsen was supported by Project PROTECFRUIT (project No grant informationber 34009-13-0686), and the Ministry of Environment and Food of Denmark (GUDP), as a part of the Organic RDD-2 program, coordinated by ICROFS Denmark.
Xoaquín Moreira was supported was supported by grants from the Spanish Ministry of Science and Innovation (PID2022-141761OB-I00 and EUR2023-143463 projects).
Xiaoming Zhang was supported by the Young Talents of "Yunnan Xingdian Talent Support Program" (YNWRQNBJ2020291), the Reserve Talent Project of Yunnan's Young and Middle-aged Academic and Technical Leaders (202105AC160071).
Frank Yonghong Li was supported by the Ministry of Science and Technology of China (Grant No. 2015BAC02B04; 2016YFC0500508) and the Department of Science and Technology of Inner Mongolia Autonomous Region of China (Grant for Key Basic Research on Grassland Ecosystem on the Mongolian Plateau).
Matthias Tschumi and Matthias Albrecht were supported by the Hauser and Sur-La-Croix foundations.
Pedro Henrique Brum Togni and Edison Ryoiti Sujii were supported by research grants and fellowships from Conselho Nacional de Desenvolvimento Científico e Tecnológico (CNPq), Fundação de Apoio a Pesquisa do Distrito Federal (FAPDF) (grant No grant informationber 00193-00000934/2019-11).
José Bruno Malaquias received a grant from FAPESP (FAPESP process: 2018/20435-5, 2017/05953-7, 2015/20380-8).
Francisco de Sousa Ramalho received a grant from the CNPq provided the initial support for the Project. Additional funds were received by Francisco de Sousa Ramalho from the Brazilian agency Financiadora de Estudos e Projetos FINEP.

Mattias Jonsson and Steve Wratten were supported by the Tertiary Education Commission through the Bio-Protection Research Centre at Lincoln University.
Alistair John Campbell was funded by the Biotechnology and Biological Sciences Research Council (BBSRC) and Syngenta UK as part of a case award PhD (grant no. 1518739).
Su Wang was funded by the National Key R&D Program of China (ref. 2017YFD0201000), the Technical Innovation Program of Beijing Academy of Agriculture and Forestry Sciences (ref. 20200110).
Filipe Madeira was funded by the EU Horizon 2020 projects EMPHASIS (Grant Agreement No 634179) and EUCLID (Grant Agreement No 633999). The authors thank the Portuguese Foundation for Science and Technology (FCT) for the financial support to the Research Centre for Natural Resources, Environment and Society-CERNAS (UIDB/00681/2025).
Gemma Clemente-Orta was funded by the grant BES-2015-072378 from the Ministry of Science, Innovation and Universities. Projects AGL2014-53970-C2-1-R. and AGL2017-84127-R funded by the Spanish Ministry of Economy, Industry and Competitiveness.
Jana Brandmeier, Christoph Scherber and Silvia Pappagallo received funding from the Euopean Union's Horizon 2020 reseach and innovation programme under Grant Agreement No grant informationber 727284.
Fengqin Cao was supported by Shanghai Science and Technology Innovation Action Plan from Shanghai Municipal Science and Technology Commission of China (22015821000).
Daniel Paredes was granted by Junta de Andalucía (PO7-AGR-2747) & Ministry of Education of Spain (D. P. FPU grant AP-2007-03970).
Ming-Qiang Wang was supported by the National Natural Science Foundation, China (32100343).
Chao-Dong Zhu was supported by the Strategic Priority Research Program of the Chinese Academy of Sciences (XDB310304).
Deli Wang and Hui Zhu were supported by the National Key Research and Development Program of China (2016YFC0500602), National Natural Science Foundation of China (31230012, 31772652, and 31770520), and the Program for Introducing Talents to Universities (B16011).
Fajun Chen was supported by National Key Research and Development Program (2017YFD0200408).
Ana Carolina Monmany-Garzia received grants from the University of Puerto Rico, ProYungas Foundation (Argentina), and a fellowship from the National Scientific and Technical Research Council of Argentina. MA received a grant from NASA-IRA.
Gianalberto Losapio was funded by the Italian Ministry of University and Research (PRIN 2022 PNRR MITEX P2022N5KYJ); Christian Schöb was funded by the Swiss National Science Foundation (PP00P3_170645 and PZ00P3_148261).
Mercedes Campos and Francisca Ruano were financed by the Excellence Project of the Andalusian Regional Government (AGR 1419).
Linsheng Wan and Xiaodong Wang were supported by Biological Breeding-Vational Science and Technology Major Project (2022ZD04010).
Abida Butt was supported by Pakistan Science Foundation , project No grant informationber PU-Bio (410).

Carmen K. Blubaugh received a grant from United States Department of Agriculture: Southern Sustainable Agricultural Research and Education grant LS20-337.
Li-Lin Chen was supported by Nanping City Science and Technology Commissioner Special Fund (no. N2023T011).
Louis Sutter received funding from the European Union's Seventh Framework Programme for research, technological development and demonstration (Contract No grant informationber 311879).
Cornelia Sattler and Josef Settele were funded by the German Federal Ministry for Economic Cooperation and Development (BMZ) commissioned and administered through the Deutsche Gesellschaft für Internationale Zusammenarbeit (GIZ) Fund for International Agricultural Research (FIA) under the Project VERDE (81219435).
Zhiping Cai was supported by the National Natural Science Foundation of China (32160664).
Jinhua Li was supported by the National Natural Science Fund of China (NSFC) (32260699).
This project was funded by the Marsden Fund (UOC-0802) to Jason Tylianakis.
Jing Shang received a grant from the national Modern Agricultural Industrial Technology System Sichuan Soy Grains Innovation Team (Project No. Sccxtd-2020-20); Sichuan Province Breeding Project (Project No. 2021YFYZ0021).
Zhengqun Zhang was supported by Natural Science Foundation of Shandong Province (ZR2020MC122) and Major Project for Agricultural Application Technology Innovation of Shandong Province (20182130106).
Anna Wenda-Piesik was supported by the Grant of Polish Ministry of Science and Higher Education: 0665/P06/98/15.
Xiaoling Tan received a grant from National Science Foundation of China (32172415) and China Agriculture Research System of MOF and MARA (CARS-22).
Vojtech Novotny received the Czech Science Foundation (GACR 23-07776S) and Praemium Academiae grants.
Yu Zhu received a grant from the National Natural Science Foundation of China (No. 42471134).
Jushan Liu was supported by the National Natural Science Foundation of China (32171540, 31971744).
Zhanfeng Liu was supported by the National Natural Science Foundation of China (32301565; 42177289); Wenjia Wu was supported by Science and Technology Plan Project of Guangdong of China (2022A1515110990).
Xiaoke Zhang was supported by National Science and Technology Fundamental Resources Investigation Program of China (2024FY100400).
Qingxuan Xu was supported by the earmarked fund for CARS (CARS-30).
Frank Yonghong Li and Yadong Wang were supported by the Department of Science and Technology of Inner Mongolia of China (Grant No. 2019ZD007, 2021ZD0011).
Quanhui Ma and Zhenzhu Xu were supported by the National Natural Science Foundation of China (31661143028, 31170456, 32471774), and China Special Fund for Meteorological Research in the Public Interest (Major projects) (GYHY201506001-3).
Qi Li were supported by Nantional Science Fundation of China (Grant No. 32271718).
Ming-Jing Qu was Supported by the earmarked fund for CARS-13.

Yinzhan Liu were supported by the National Natural Science Foundation of China (31670477).
Xinqiang Xi were supported by Nantional Science Fundation of China (Grant No. 32022409).
Pingyang Zhu was suported by 'Shuanglong Scholar' Research Start-up Fund of Zhejiang Normal University, China (YS304021920).
Severin Hatt received a grant from the European Union's Horizon 2020 programme "Nurturing excellence by means of cross-border and cross-sector mobility" (Marie Sklodowska-Curie Individual Fellowship, Grant agreement ID: 891566).
Anicet Dassou was supported by SDC-2.
Philip C. Stevenson was supported by Grand Challenges Research Fund (BBSRC) (Grant No grant informationber: BB/R020361/1).
McKnight Foundation (Grant No grant informationber 20-034) and Darwin Initiative grant (DI22-012).
Yi Li was supported by the National Natural Science Foundation of China (32301337).
Tesfay Gidey received a grant from the Adigrat University, Ethiopia (grant Id: AGU/CAES/071/11).
Xiaoming Lu was supported by the National Key Research and Development Program for Young Scientists of China (2024YFF1308700).
Yordanys Ramos was supported by Universidad Central "Marta Abreu" de Las Villas, Santa Clara, Cuba.
Ola Lundin recieved a grant from Formas (grant No 2019- 01294) for this research.
Péter Batáry was supported by the grants of "Biodiversity under agricultural and urbanisation constraints" (NKFIH KKP 133839).
Shunsuke Utsumi received a grant from JSPS (19H02974).
This work was done under the framework of the Gottfried Wilhelm Leibniz Prize (Ei 862/29-1) (Nico Eisenhauer was awarded the prize in 2021. Huimin Yi has been paid by the prize money as a PhD student, with the supervision of Nico Eisenhauer and Olga Ferlian, to finish the work).
F. Xavier Sans-Serra received funding from the European Union's Horizon 2020 research and innovation program under Grant Agreement no. 773554. Duration of the Project: September 2018-March 2024. www.ecostack-h2020.eu, info@ecostack-h2020.eu. This research was partially supported by Proyecto PID2021-127575OB-I00 financed by MICIU/AEI /10.13039/501100011033 and by FEDER, UE.
Finbarr G. Horgan received funding from the Philippines Department of Agriculture, Bureau of Agricultural Research (Project: Developing ecological engineering approaches to restore and conserve ecosystem services for pest management for sustainable rice production in the Philippines).
Geoff Gurr received a grant from Horticulture Innovation (VG16062).
Alina Twerski was supported by The German Federal Environmental Foundation (DBU) (grant No grant informationber AZ 34.029/01).
Ingolf Steffan-Dewenter was funded from 2017-2018 Belmont Forum and BiodivERsA joint call for research proposals, under the BiodivScen ERA-NetCOFUND program, and from German Federal Ministry of Education and Research (BMBF #01LC11804A).
Minoo Heidari Latiberi was financed by the Second Century Fund (C2F), Chulalongkorn University, Thailand. Mostafa Ghafouri Moghaddam was supported by Rachadaphisek

Somphot Fund for postdoctoral fellowship, Graduate School, Chulalongkorn University, Thailand. Buntika Areekul Butcher was financed by Thailand Science Research and Innovation Fund, Chulalongkorn University (BCG_FF_68_178_2300_039).
Md Panna Ali received a grant U.S. National Academy of Sciences (NAS) via USAID and NAS Prime Agreement No. AID-OAA-A-11 -00012. DAL (US partner on the PEER grant) acknowledges support from the NSF Long-term Ecological Research Program (DEB 1637653) at the Kellogg Biological Station, Michigan State University South Asia Partnership and MSU AgBioResearch.
Daniel Munyao Mutyambai received a grant from Biovision Foundation (DPP-020/2022-2024) and IKEA Foundation (Grant no. DN00151).
The Jena Experiment is funded by the German Research Foundation (FOR 456) and supported by the Friedrich-Schiller-University of Jena and the Max-Planck-Institute for Biogeochemistry, Jena. Additional support was provided by the Swiss National Science Foundation (grant no. 31-65224-01 to Bernhard Schmid).
Minoo Heidari Latiberi was financed by the Second Century Fund (C2F), Chulalongkorn University, Thailand.
Séverin Hatt was financed by the European Commission - grant number 891566.
Mostafa Ghafouri Moghaddam was supported by Rachadaphisek Somphot Fund, Graduate School, Chulalongkorn University, Thailand and also was supported by Modal Insan University Fund (RIA2) for postdoctoral fellowship, Universiti Kebangsaan Malaysia, Malaysia.

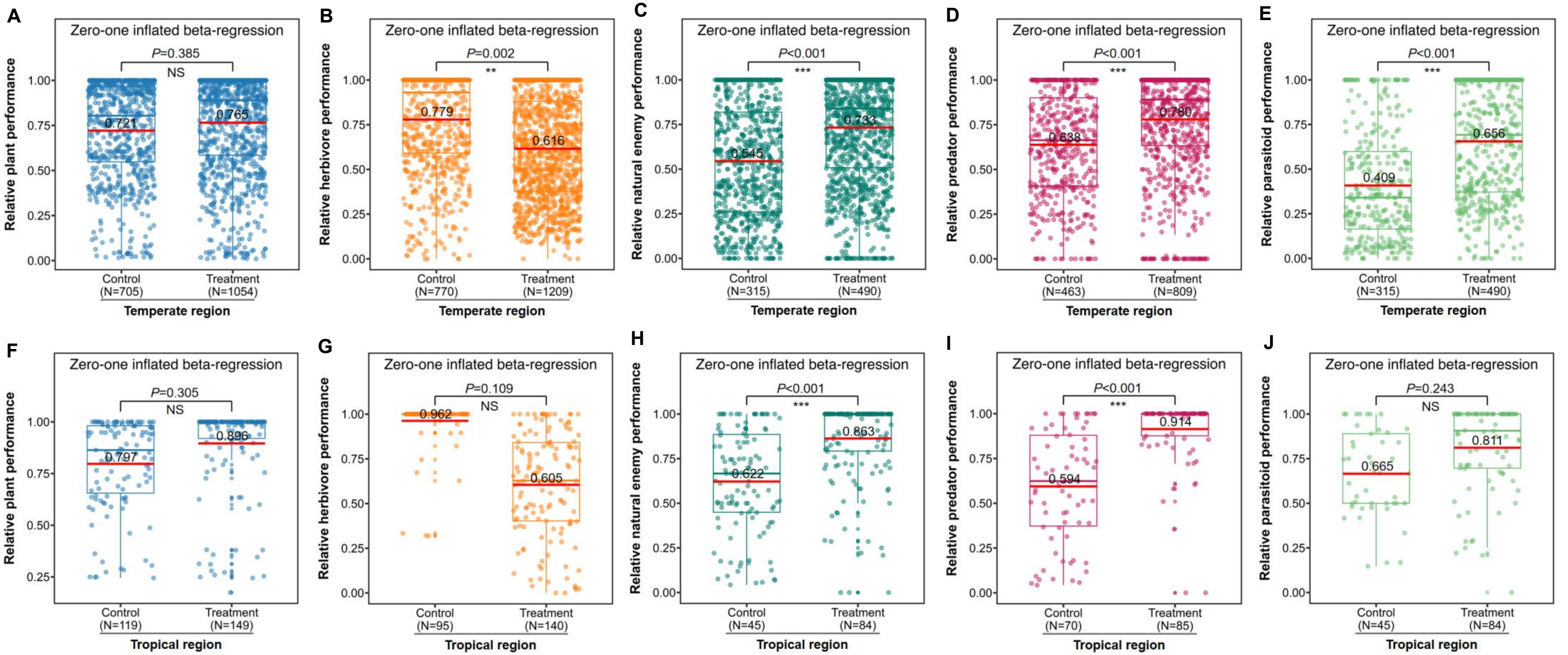

Fig. S1. The effects of binary plant diversity on the trophic groups in global tri-trophic field experiments across different climatic regions. **A**, Comparison of relative plant performance between the control and the treatment in temperate regions. **B**, Comparison of relative invertebrate herbivore performance between the control and the treatment in temperate regions. **C**, Comparison of relative natural enemy performance between the control and the treatment in temperate regions. **D**, Comparison of relative predator performance between the control and the treatment in temerate regions. **E**, Comparison of relative parasitoid performance between the control and the treatment in temperate regions. **F**, Comparison of relative plant performance between the control and the treatment in tropical regions. **G**, Comparison of relative herbivore performance between the control and the treatment in tropical regions. **H**, Comparison of relative natural enemy performance between the control and the treatment in tropical regions. **I**, Comparison of relative predator performance between the control and the treatment in tropical regions. **J**, Comparison of relative parasitoid performance between the control and the treatment in tropical regions. Plant performance includes the growth, reproduction and quality of plants, herbivore performance includes the abundance, damage and diversity of herbivores, and natural enemy performance includes the predator abundance, predation, predator diversity, parasitoid abundance, parasitism and parasitoid diversity. Relative value of each performance is the actual value divided by the maximun value in each replication in each experimnt. The "Control" represents single, pure or lowest plant species and "Treatment" represents the higher plant species richness (≥2). The zero-or-one inflated beta regression was used to test whether the sets of values in two groups are significantly different (NS, P>0.05; *P<0.05, **P<0.01, ***P<0.001; see table S2), lines within each box denote the median, outer borders of the boxes show the upper and lower quartile, lines outside of the boxes depict the maximum and minimum number of individuals, and red lines in each box represent the average value of relative performance of plants, herbivores or natural enemies in the control (i.e., single, pure or lowest plant species richness) and treatments (i.e., higher plant species richness, ≥2).

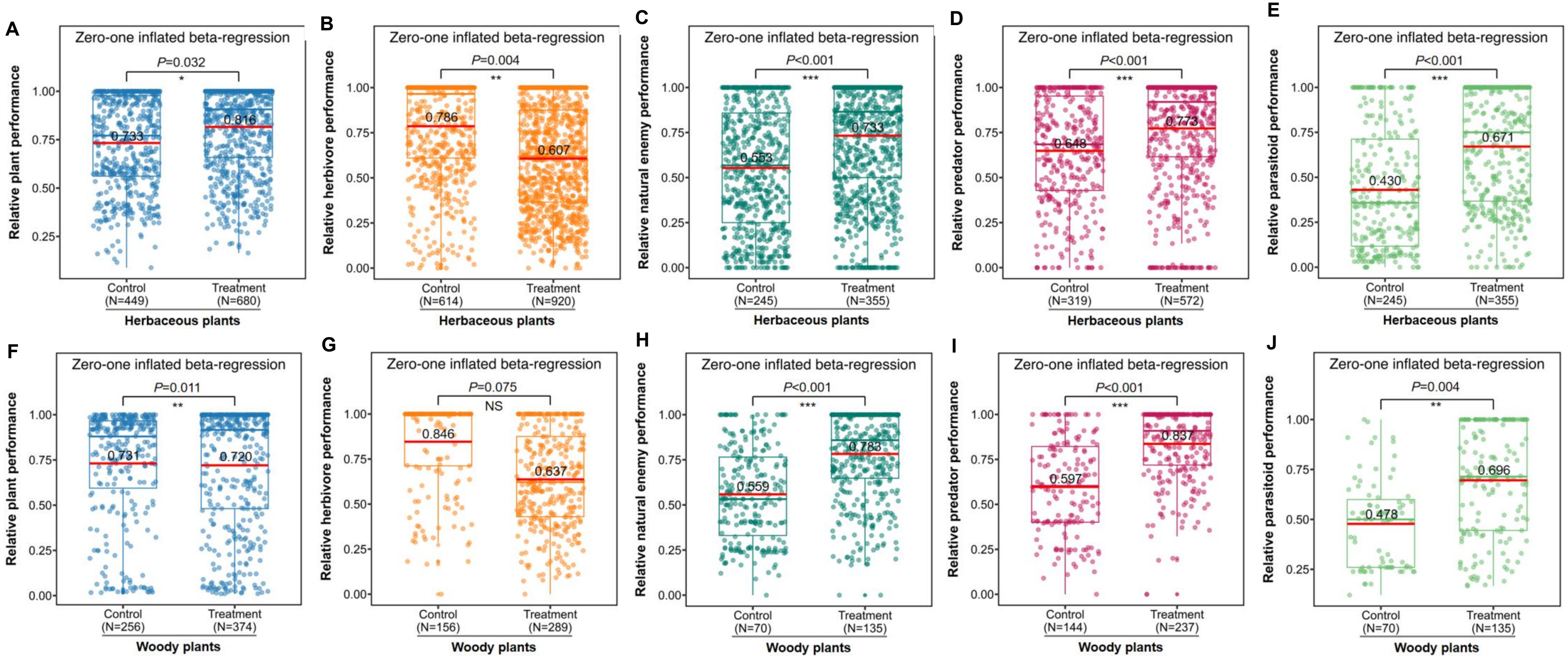

Fig. S2. The effects of binary plant diversity on the trophic groups in global tri-trophic field experiments across different plant types. **A**, Comparison of relative plant performance between the control and the treatment for herbaceous plants. **B**, Comparison of relative invertebrate herbivore performance between the control and the treatment for herbaceous plants. **C**, Comparison of relative natural enemy performance between the control and the treatment for herbaceous plants. **D**, Comparison of relative predator performance between the control and the treatment for herbaceous plants. **E**, Comparison of relative parasitoid performance between the control and the treatment for herbaceous plants. **F**, Comparison of relative plant performance between the control and the treatment for woody plants. **G**, Comparison of relative herbivore performance between the control and the treatment for woody plants. **H**, Comparison of relative natural enemy performance between the control and the treatment for woody plants. **I**, Comparison of relative predator performance between the control and the treatment for woody plants. **J**, Comparison of relative parasitoid performance between the control and the treatment for woody plants. Plant performance includes the growth, reproduction and quality of plants, herbivore performance includes the abundance, damage and diversity of herbivores, and natural enemy performance includes the predator abundance, predation, predator diversity, parasitoid abundance, parasitism and parasitoid diversity. Relative value of each performance is the actual value divided by the maximun value in each replication in each experimnt. The “Control” represents single, pure or lowest plant species and “Treatment” represents the higher plant species richness (≥2). The zero-or-one inflated beta regression was used to test whether the sets of values in two groups are significantly different (NS, $P>0.05$; $^{*}P<0.05$, $^{**}P<0.01$, $^{***}P<0.001$; see table S3), lines within each box denote the median, outer borders of the boxes show the upper and lower quartile, lines outside of the boxes depict the maximum and minimum number of individuals, and red lines in each box represent the average value of relative performance of plants, herbivores or natural enemies in the control (i.e., single, pure or lowest plant species richness) and treatments (i.e., higher plant species richness, ≥2).

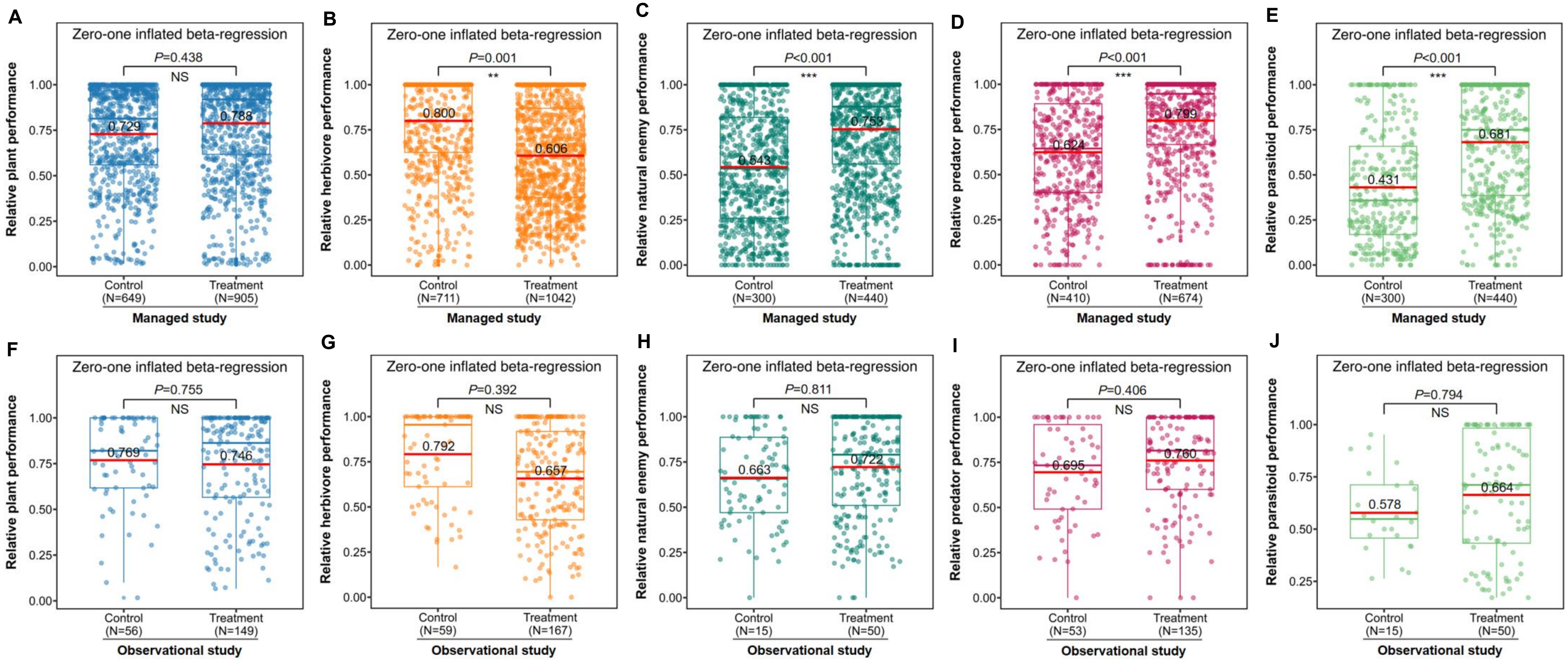

Fig. S3. The effects of binary plant diversity on the trophic groups in global tri-trophic field experiments across different study types. **A**, Comparison of relative plant performance between the control and the treatment in managed study. **B**, Comparison of relative invertebrate herbivore performance between the control and the treatment in managed study. **C**, Comparison of relative natural enemy performance between the control and the treatment in managed study. **D**, Comparison of relative predator performance between the control and the treatment in managed study. **E**, Comparison of relative parasitoid performance between the control and the treatment in managed study. **F**, Comparison of relative plant performance between the control and the treatment in observational study. **G**, Comparison of relative herbivore performance between the control and the treatment in observational study. **H**, Comparison of relative natural enemy performance between the control and the treatment in observational study. **I**, Comparison of relative predator performance between the control and the treatment in observational study. **J**, Comparison of relative parasitoid performance between the control and the treatment in observational study. Plant performance includes the growth, reproduction and quality of plants, herbivore performance includes the abundance, damage and diversity of herbivores, and natural enemy performance includes the predator abundance, predation, predator diversity, parasitoid abundance, parasitism and parasitoid diversity. Relative value of each performance is the actual value divided by the maximun value in each replication in each experimnt. The "Control" represents single, pure or lowest plant species and "Treatment" represents the higher plant species richness (≥2). The zero-or-one inflated beta regression was used to test whether the sets of values in two groups are significantly different (NS, $P>0.05$; *$P<0.05$, **$P<0.01$, ***$P<0.001$; see table S4), lines within each box denote the median, outer borders of the boxes show the upper and lower quartile, lines outside of the boxes depict the maximum and minimum number of individuals, and red lines in each box represent the average value of relative performance of plants, herbivores or natural enemies in the control (i.e., single, pure or lowest plant species richness) and treatments (i.e., higher plant species richness, ≥2).

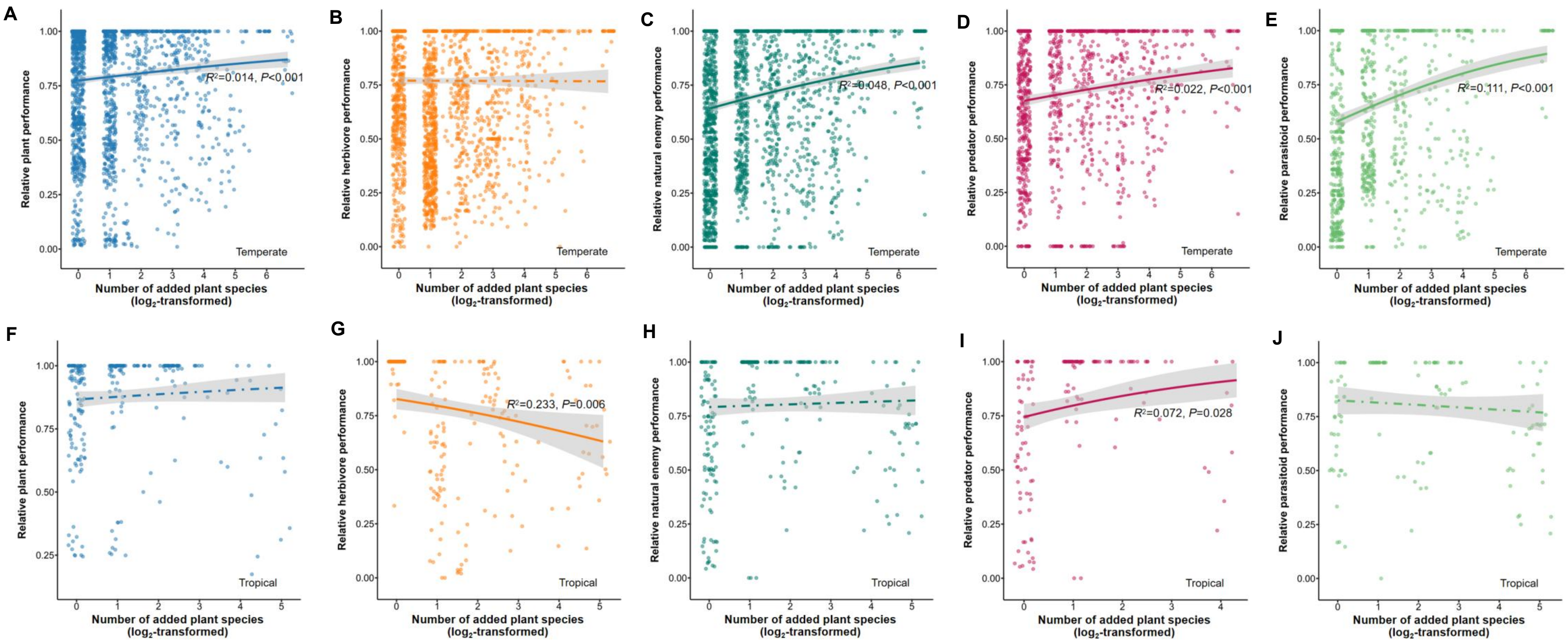

Fig. S4. The effects of the number of added plant species on the trophic groups in global tri-trophic field experiments across different climatic regions. A, Relationships between number of added plant species and relative plant performance in temperate regions. **B**, Relationships between number of added plant species and relative herbivore performance in temperate regions. **C**, Relationships between number of added plant species and relative natural enemy performance in temperate regions. **D**, Relationships between number of added plant species and relative predator performance in temperate regions. **E**, Relationships between number of added plant species and relative parasitoid performance in temperate regions. **F**, Relationships between number of added plant species and relative plant performance in tropical regions. **G**, Relationships between number of added plant species and relative herbivore performance in tropical regions. **H**, Relationships between number of added plant species and relative natural enemy performance in tropical regions. **I**, Relationships between number of added plant species and relative predator performance in tropical regions. **J**, Relationships between number of added plant species and relative parasitoid performance in tropical regions. Solid lines show significant ($P<0.05$) effects, dashed lines show non-significant ($P>0.05$) relationships, the grey-shaded zone covers the 95% confidence interval (see table S5).

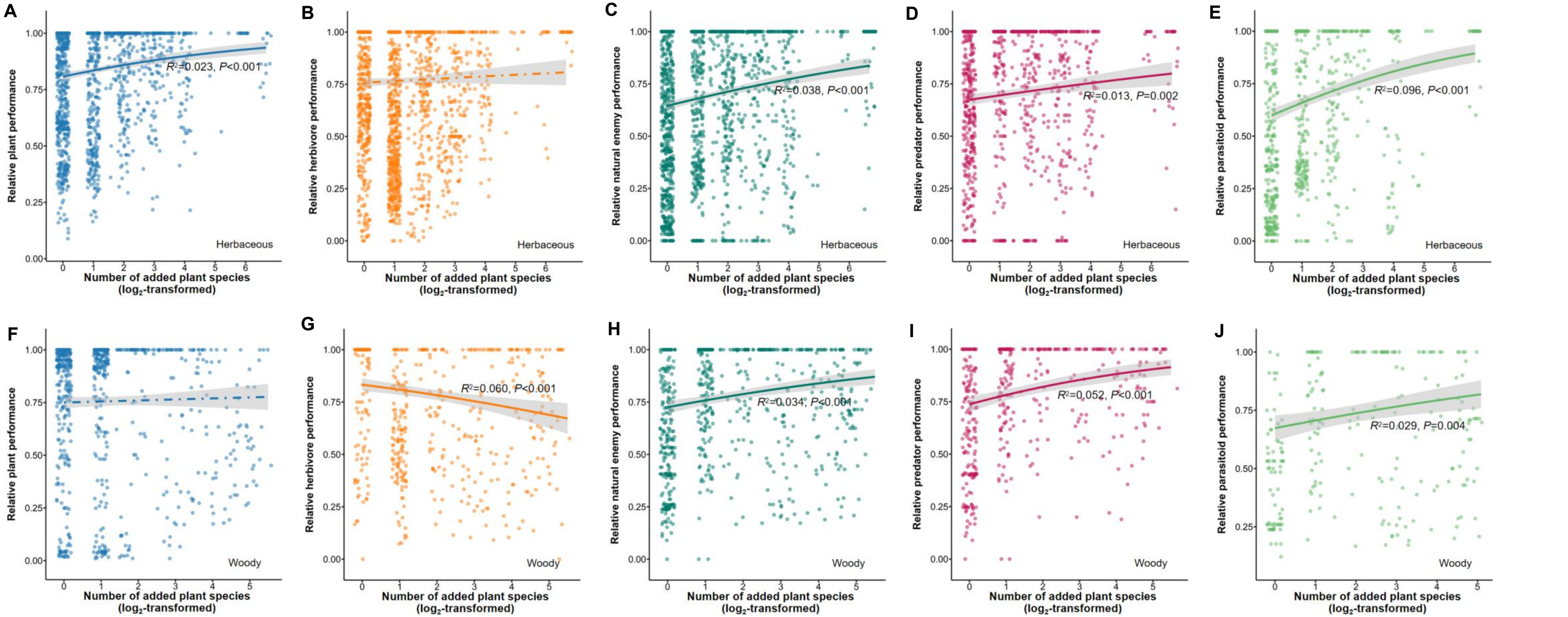

Fig. S5. The effects of the number of added plant species on the trophic groups in global tri-trophic field experiments across different plant types. A, Relationships between number of added plant species and relative plant performance for herbaceous plants. **B**, Relationships between number of added plant species and relative herbivore performance for herbaceous plants. **C**, Relationships between number of added plant species and relative natural enemy performance for herbaceous plants. **D**, Relationships between number of added plant species and relative predator performance for herbaceous plants. **E**, Relationships between number of added plant species and relative parasitoid performance for herbaceous plants. **F**, Relationships between number of added plant species and relative plant performance for woody plants. **G**, Relationships between number of added plant species and relative herbivore performance for woody plants. **H**, Relationships between number of added plant species and relative natural enemy performance for woody plants. **I**, Relationships between number of added plant species and relative predator performance for woody plants. **J**, Relationships between number of added plant species and relative parasitoid performance for woody plants. Solid lines show significant ($P<0.05$) effects, dashed lines show non-significant ($P>0.05$) relationships, the grey-shaded zone covers the 95% confidence interval (see table S5).

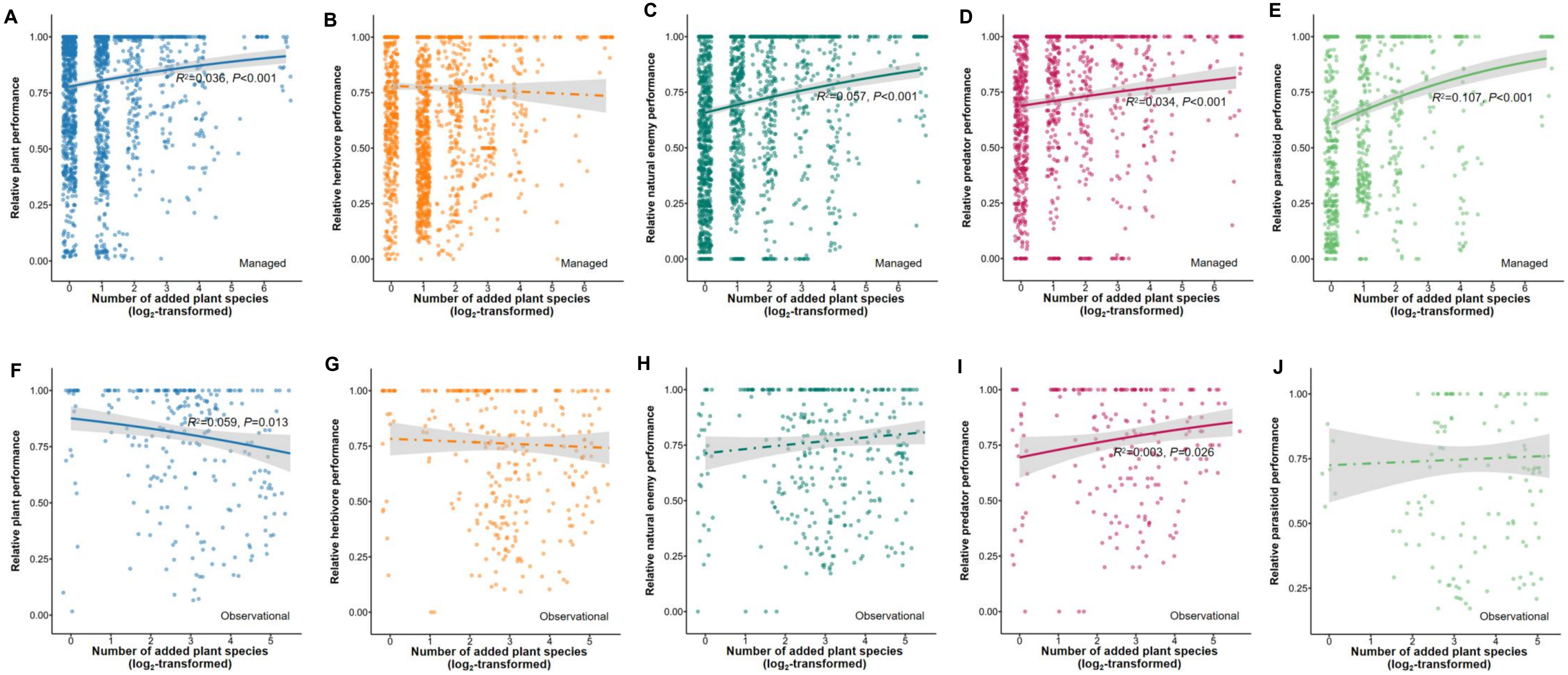

Fig. S6. The effects of the number of added plant species on the trophic groups in global tri-trophic field experiments across different study types. A, Relationships between number of added plant species and relative plant performance in managed study. **B**, Relationships between number of added plant species and relative herbivore performance in managed study. **C**, Relationships between number of added plant species and relative natural enemy performance in managed study. **D**, Relationships between number of added plant species and relative predator performance in managed study. **E**, Relationships between number of added plant species and relative parasitoid performance in managed study. **F**, Relationships between number of added plant species and relative plant performance in observational study. **G**, Relationships between number of added plant species and relative herbivore performance in observational study. **H**, Relationships between number of added plant species and relative natural enemy performance in observational study. **I**, Relationships between number of added plant species and relative predator performance in observational study. **J**, Relationships between number of added plant species and relative parasitoid performance in observational study. Solid lines show significant (P<0.05) effects, dashed lines show non-significant (P>0.05) relationships, the grey-shaded zone covers the 95% confidence interval (see table S5).

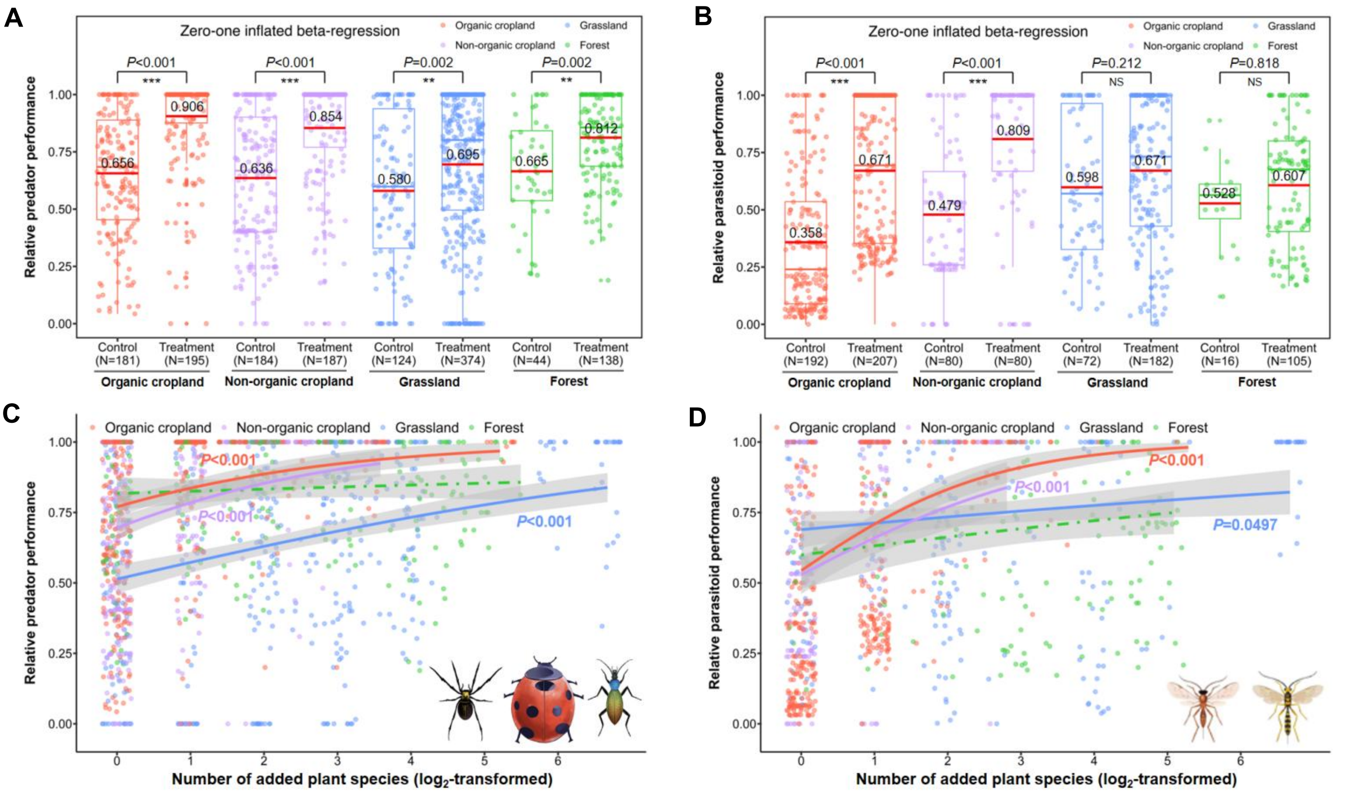

Fig. S7. The effects of plant diversity on the invertebrate predators and parasitoids in global field experiments of tri-trophic interactions. A, Comparison of relative invertebrate predator performance between the control and the treatment. **B**, Comparison of relative invertebrate parasitoid performance between the control and the treatment. **C**, Relationships between number of added plant species and relative predator performance. **D**, Relationships between number of added plant species and relative parasitoid performance. Invertebrate predator performance includes the predator abundance, predation and predator diversity. Invertebrate parasitoid performance includes the parasitoid abundance, parasitism and parasitoid diversity. Relative value of each performance is the actual value divided by the maximun value in each replication in each experimnt. In figs. S7A, B (see table S1), the "Control" represents single, pure or lowest plant species and "Treatment" represents the higher plant species richness (≥2), the zero-or-one inflated beta regression was used to test whether the sets of values in two groups are significantly different (NS, P>0.05; *P<0.05, **P<0.01, ***P<0.001), lines within each box denote the median, outer borders of the boxes show the upper and lower quartile, lines outside of the boxes depict the maximum and minimum number of individuals, and red lines in each box represent the average value of relative performance of predators or parastoids in the control (i.e., single, pure or lowest plant species richness) and treatments (i.e., higher plant species richness, ≥2). In figs. S7C, D with beta regressions (see table S5), solid lines show significant (P<0.05) effects, dashed lines show non-significant (P>0.05) relationships, the grey-shaded zone covers the 95% confidence interval. Photoshop8.0 was used to design the images.

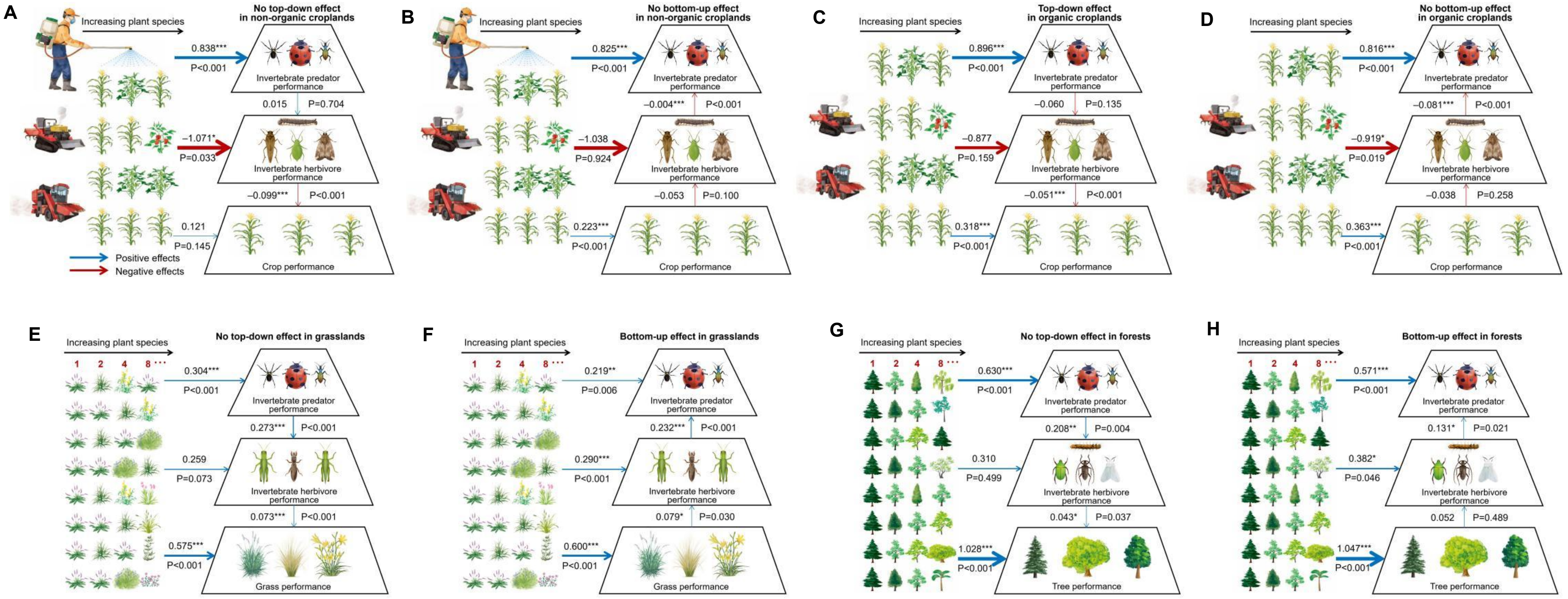

Fig. S8. Structural equation model to test for an association between an increase in plant diversity and the top-down and bottom-up effects among plants, invertebrate herbivores and their invertebrate predators across different ecosystems. **A**, No top-down effects in non-organic croplands (N=621). **B**, No bottom-up effects in non-organic croplands (N=621). **C**, Top-down effects in organic croplands (N=596). **D**, No bottom-up effects in organic croplands (N=596). **E**, No top-down effects in grasslands (N=578). **F**, Bottom-up effects in grasslands (N=578). **G**, No top-down effects in forests (N=202). **H**, Bottom-up effects in forests (N=202). Crop, grass or forest performance includes the growth, reproduction and quality of plants, herbivore performance includes the abundance, damage and diversity of herbivores, and predator performance includes the predator abundance, predation, and predator diversity. *P<0.05, **P<0.01, ***P<0.001. Blue and red arrows denote positive and negative relationships, respectively. Numbers next to each arrow are the estimated coefficients from structural equation models, and line width is proportional to the magnitude of the coefficients (table S16). Piecewise structural equation model (restricted maximum likelihood, REML) was used to test the effects of plant diversity (measured as the number of plant species generalize to binary variable) on the tri-trophic interactions of plants, invertebrate herbivores and their invertebrate predators. Photoshop8.0 was used to design the images.

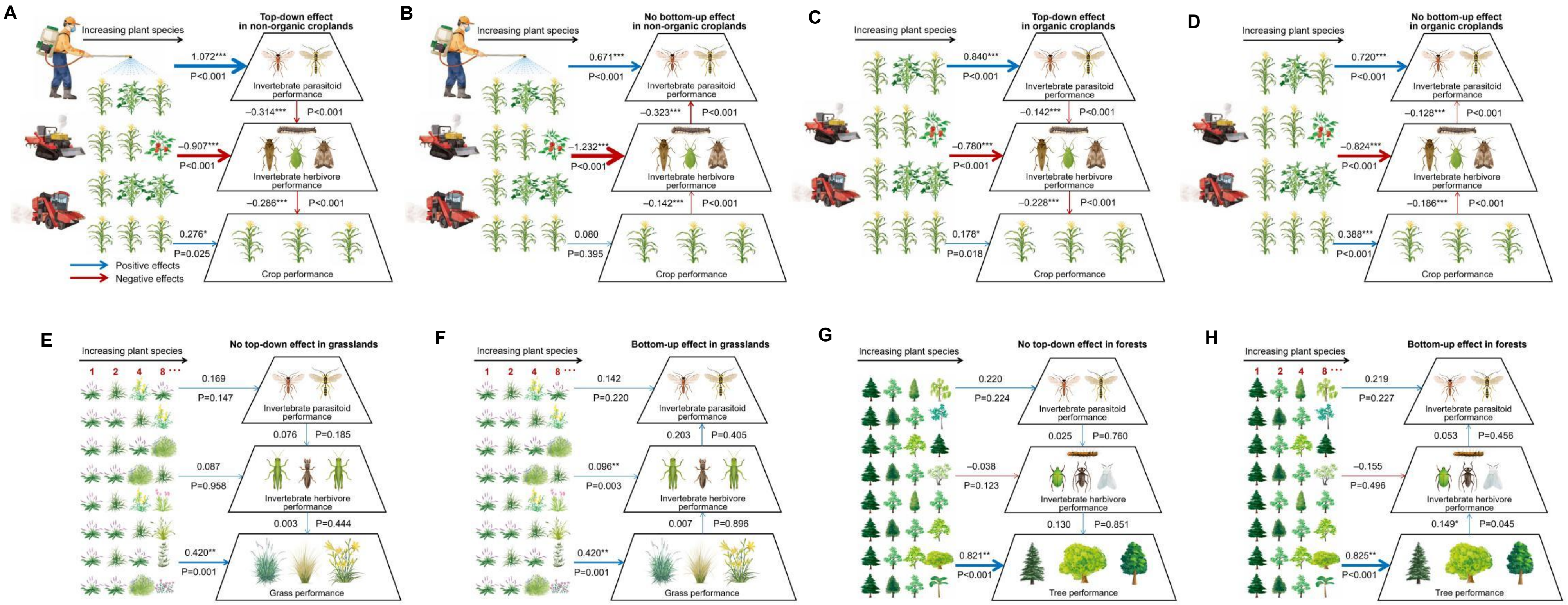

Fig. S9. Structural equation model to test for an association between an increase in plant diversity and the top-down and bottom-up effects among plants, invertebrate herbivores and their invertebrate parasitoids across different ecosystems. **A**, Top-down effect in non-organic croplands (N=266). **B**, No bottom-up effect in non-organic croplands (N=266). **C**, Top-down effect in organic croplands (N=593). **D**, No bottom-up effect in organic croplands (N=593). **E**, No top-down effect in grasslands (N=254). **F**, Bottom-up effect in grasslands (N=254). **G**, No top-down effect in forests (N=131). **H**, Bottom-up effect in forests (N=131). Crop, grass or forest performance includes the growth, reproduction and quality of plants, herbivore performance includes the abundance, damage and diversity of herbivores, and parasitoid performance includes the parasitoid abundance, parasitism and parasitoid diversity. *P<0.05, **P<0.01, ***P<0.001. Blue and red arrows denote positive and negative relationships, respectively. Numbers next to each arrow are the estimated coefficients from structural equation models, and line width is proportional to the magnitude of the coefficients (table S17). Piecewise structural equation model (restricted maximum likelihood, REML) was used to test the effects of plant diversity (measured as the number of plant species generalize to binary variable) on the tri-trophic interactions of plants, invertebrate herbivores and their incertebrate parasitoids. Photoshop8.0 was used to design the images.

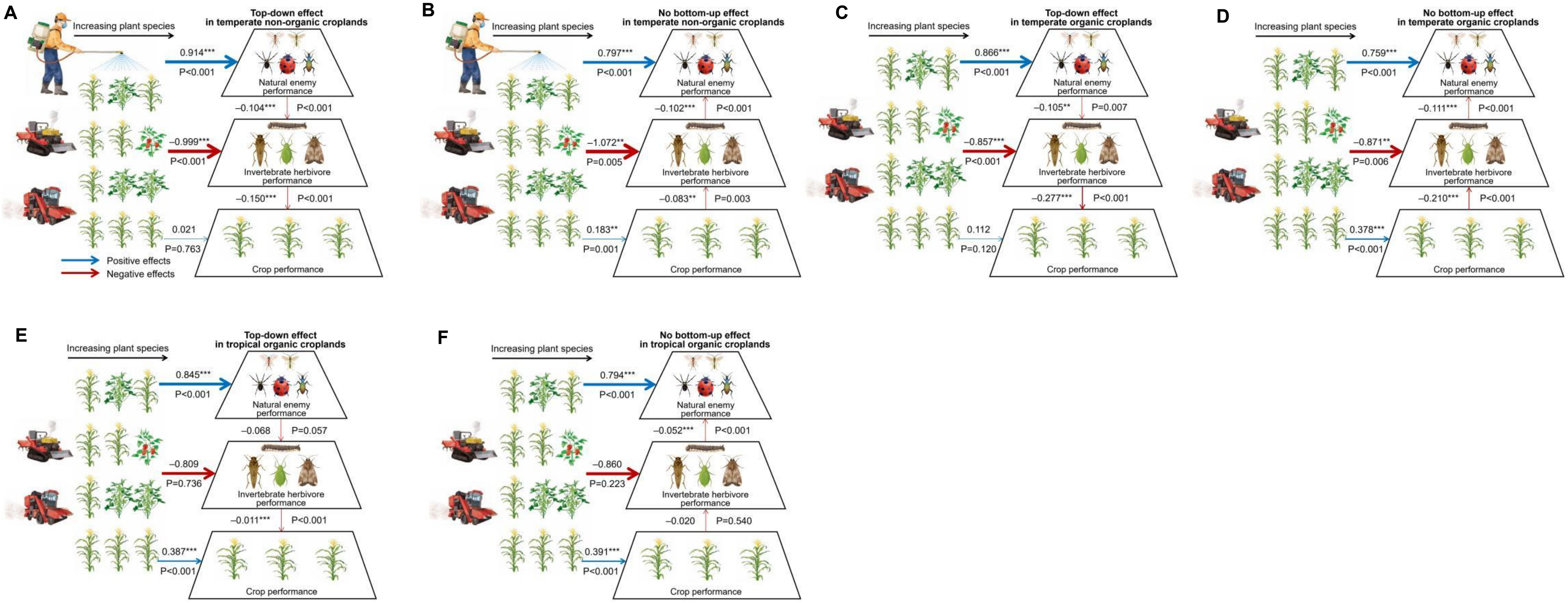

Fig. S10. Structural equation model to test for an association between an increase in plant diversity and the tri-trophic interactions of crops, invertebrate herbivores and their natural enemies in croplands across different climatic regions. **A**, Top-down effect in temperate non-organic croplands (N=863). **B**, No bottom-up effect in temperate non-organic croplands (N=863). **C**, Top-down effect in temperate organic croplands (N=635). **D**, No bottom-up effect in temperate organic croplands (N=635). **E**, Top-down effect in tropical organic croplands (N=554). **H**, No bottom-up effect in tropical organic croplands (N=554). There is no sufficient data to analyze the top-down or bottom-up effect in tropical non-organic croplands (N=24). Crop performance includes the growth, reproduction and quality of crops, herbivore performance includes the abundance, damage and diversity of herbivores, and natural enemy performance includes the predator abundance, predation, predator diversity, parasitoid abundance, parasitism and parasitoid diversity. *P<0.05, **P<0.01, ***P<0.001. Blue and red arrows denote positive and negative relationships, respectively. Numbers next to each arrow are the estimated coefficients from structural equation models, and line width is proportional to the magnitude of the coefficients (table S7). Piecewise structural equation model (restricted maximum likelihood, REML) was used to test the effects of plant diversity (measured as the number of plant species generalize to binary variable) on the tri-trophic interactions of crops, invertebrate herbivores and their natural enemies. Photoshop8.0 was used to design the images.

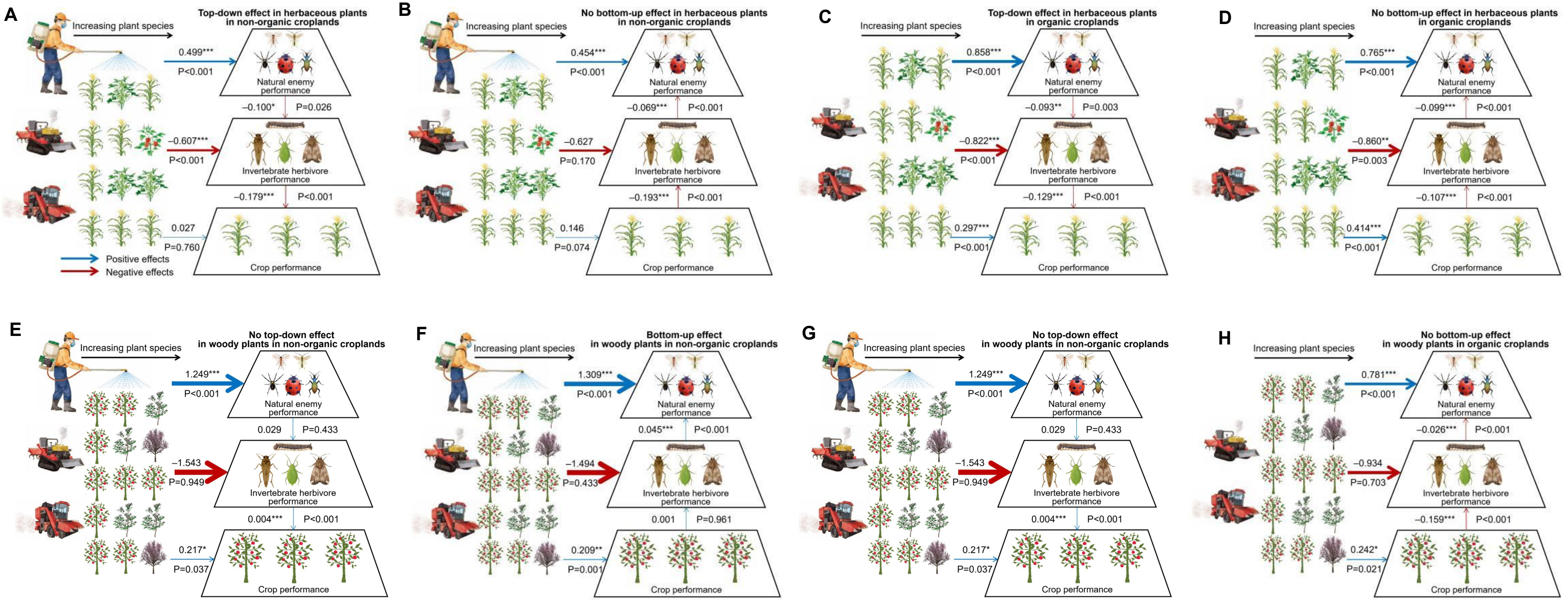

Fig. S11. Structural equation model to test for an association between an increase in plant diversity and the tri-trophic interactions of crops, invertebrate herbivores and their natural enemies in croplands across different plant types. **A**, Top-down effect in herbaceous plants in non-organic croplands (N=405). **B**, No bottom-up effect in herbaceous plants in non-organic croplands (N=405). **C**, Top-down effect in herbaceous plants in organic croplands (N=945). **D**, No bottom-up effect in herbaceous plants in organic croplands (N=945). **E**, No top-down effect in woody plants in non-organic croplands (N=482). **F**, Bottom-up effect in woody plants in non-organic croplands (N=482). **G**, Top-down effect in woody plants in organic croplands (N=244). **H**, No bottom-up effect in woody plants in organic croplands (N=244). Crop performance includes the growth, reproduction and quality of crops, herbivore performance includes the abundance, damage and diversity of herbivores, and natural enemy performance includes the predator abundance, predation, predator diversity, parasitoid abundance, parasitism and parasitoid diversity. *P<0.05, **P<0.01, ***P<0.001. Blue and red arrows denote positive and negative relationships, respectively. Numbers next to each arrow are the estimated coefficients from structural equation models, and line width is proportional to the magnitude of the coefficients (table S7). Piecewise structural equation model (restricted maximum likelihood, REML) was used to test the effects of plant diversity (measured as the number of plant species generalize to binary variable) on the tri-trophic interactions of crops, invertebrate herbivores and their natural enemies. Photoshop8.0 was used to design the images.

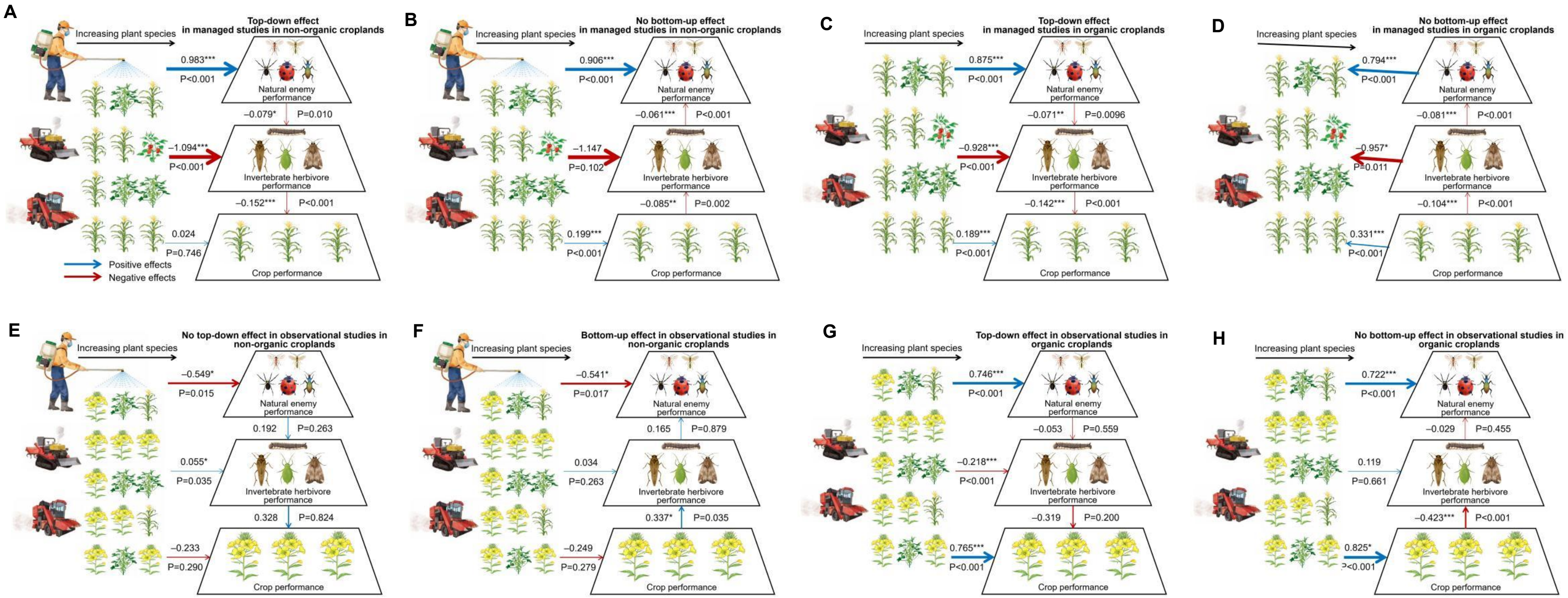

Fig. S12. Structural equation model to test for an association between an increase in plant diversity and the tri-trophic interactions of crops, invertebrate herbivores and their natural enemies in croplands across different study types. **A**, Top-down effect in managed studies in non-organic croplands (N=844). **B**, No bottom-up effect in managed studies in non-organic croplands (N=844). **C**, Top-down effect in managed studies in organic croplands (N=1079). **D**, No bottom-up effect in managed studies in organic croplands (N=1079). **E**, No top-down effect in observational studies in non-organic croplands (N=43). **F**, Bottom-up effect in observational studies in non-organic croplands (N=43). **G**, Top-down effect in observational studies in organic croplands (N=110). **H**, No bottom-up effect in observational studies in organic croplands (N=110). Crop performance includes the growth, reproduction and quality of crops, herbivore performance includes the abundance, damage and diversity of herbivores, and natural enemy performance includes the predator abundance, predation, predator diversity, parasitoid abundance, parasitism and parasitoid diversity. *P<0.05, **P<0.01, ***P<0.001. Blue and red arrows denote positive and negative relationships, respectively. Numbers next to each arrow are the estimated coefficients from structural equation models, and line width is proportional to the magnitude of the coefficients (table S7). Piecewise structural equation model (restricted maximum likelihood, REML) was used to test the effects of plant diversity (measured as the number of plant species generalize to binary variable) on the tri-trophic interactions of crops, invertebrate herbivores and their natural enemies. Photoshop8.0 was used to design the images.

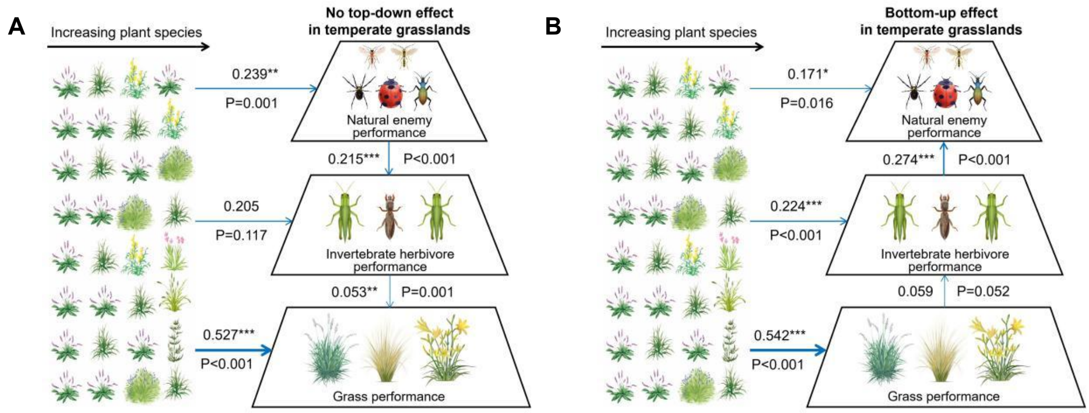

Fig. S13. Structural equation model to test for an association between an increase in plant diversity and the tri-trophic interactions of grasses, invertebrate herbivores and their natural enemies in grasslands in temperate regions. **A**, No top-down effect in temperate grasslands (N=832). **B**, Bottom-up effect in temperate grasslands (N=832). There is no data to test the effects of plant diversity on bottom-up effect in tropical grasslands (N=0). Grass performance includes the growth, reproduction and quality of grasses, herbivore performance includes the abundance, damage and diversity of herbivores, and natural enemy performance includes the predator abundance, predation, predator diversity, parasitoid abundance, parasitism and parasitoid diversity. *P<0.05, **P<0.01, ***P<0.001. Blue and red arrows denote positive and negative relationships, respectively. Numbers next to each arrow are the estimated coefficients from structural equation models, and line width is proportional to the magnitude of the coefficients (table S8). Piecewise structural equation model (restricted maximum likelihood, REML) was used to test the effects of plant diversity (measured as the number of plant species generalize to binary variable) on the tri-trophic interactions of grasses, invertebrate herbivores and their natural enemies. Photoshop8.0 was used to design the images.

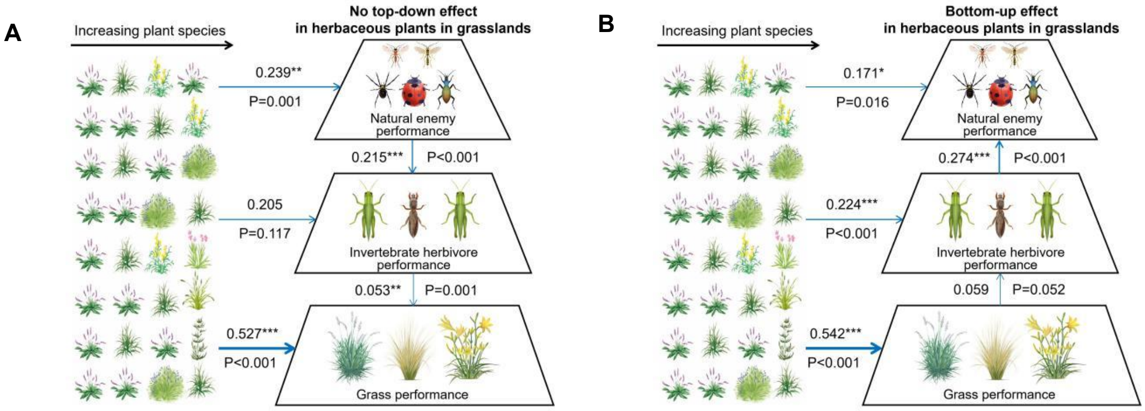

Fig. S14. Structural equation model to test for an association between an increase in plant diversity and the tri-trophic interactions of grasses, invertebrate herbivores and their natural enemies in herbaceous plants in grasslands. **A**, Top-down effect in herbaceous plants in grasslands (N=832). **B**, No bottom-up effect in herbaceous plants in grasslands (N=832). There is no data to test the effects of plant diversity on top-down or bottom-up effect in woody grasslands (N=0). Grass performance includes the growth, reproduction and quality of grasses, herbivore performance includes the abundance, damage and diversity of herbivores, and natural enemy performance includes the predator abundance, predation, predator diversity, parasitoid abundance, parasitism and parasitoid diversity. *P<0.05, **P<0.01, ***P<0.001. Blue and red arrows denote positive and negative relationships, respectively. Numbers next to each arrow are the estimated coefficients from structural equation models, and line width is proportional to the magnitude of the coefficients (table S8). Piecewise structural equation model (restricted maximum likelihood, REML) was used to test the effects of plant diversity (measured as the number of plant species generalize to binary variable) on the tri-trophic interactions of grasses, invertebrate herbivores and their natural enemies. Photoshop8.0 was used to design the images.

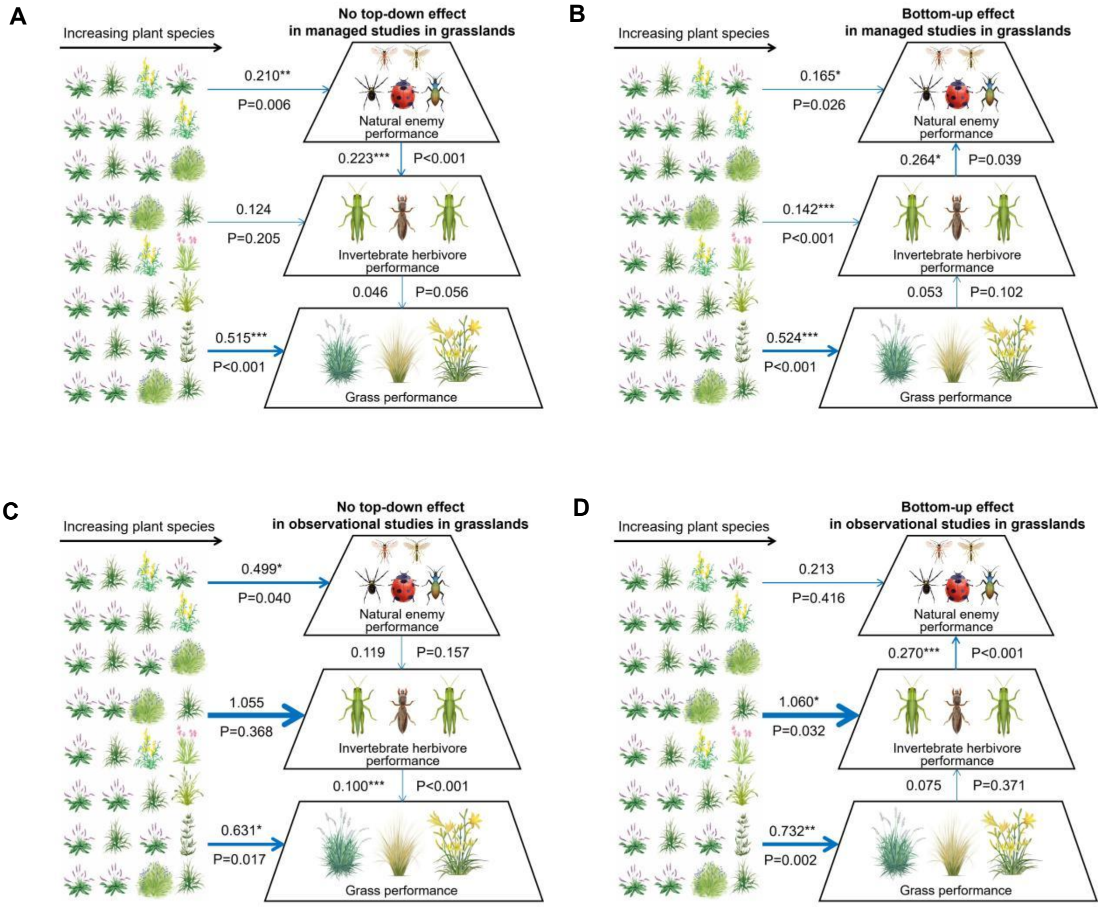

Fig. S15. Structural equation model to test for an association between an increase in plant diversity and the tri-trophic interactions of grasses, invertebrate herbivores and their natural enemies in grasslands across different study types. **A**, No top-down effect in managed studies in grasslands (N=738). **B**, Bottom-up effect in managed studies in grasslands (N=738). **C**, No top-down effect in observational studies in grasslands (N=94). **D**, Bottom-up effect in observational studies in grasslands (N=94). Grass performance includes the growth, reproduction and quality of grasses, herbivore performance includes the abundance, damage and diversity of herbivores, and natural enemy performance includes the predator abundance, predation, predator diversity, parasitoid abundance, parasitism and parasitoid diversity. *P<0.05, **P<0.01, ***P<0.001. Blue and red arrows denote positive and negative relationships, respectively. Numbers next to each arrow are the estimated coefficients from structural equation models, and line width is proportional to the magnitude of the coefficients (table S8). Piecewise structural equation model (restricted maximum likelihood, REML) was used to test the effects of plant diversity (measured as the number of plant species generalize to binary variable) on the tri-trophic interactions of grasses, invertebrate herbivores and their natural enemies. Photoshop8.0 was used to design the images.

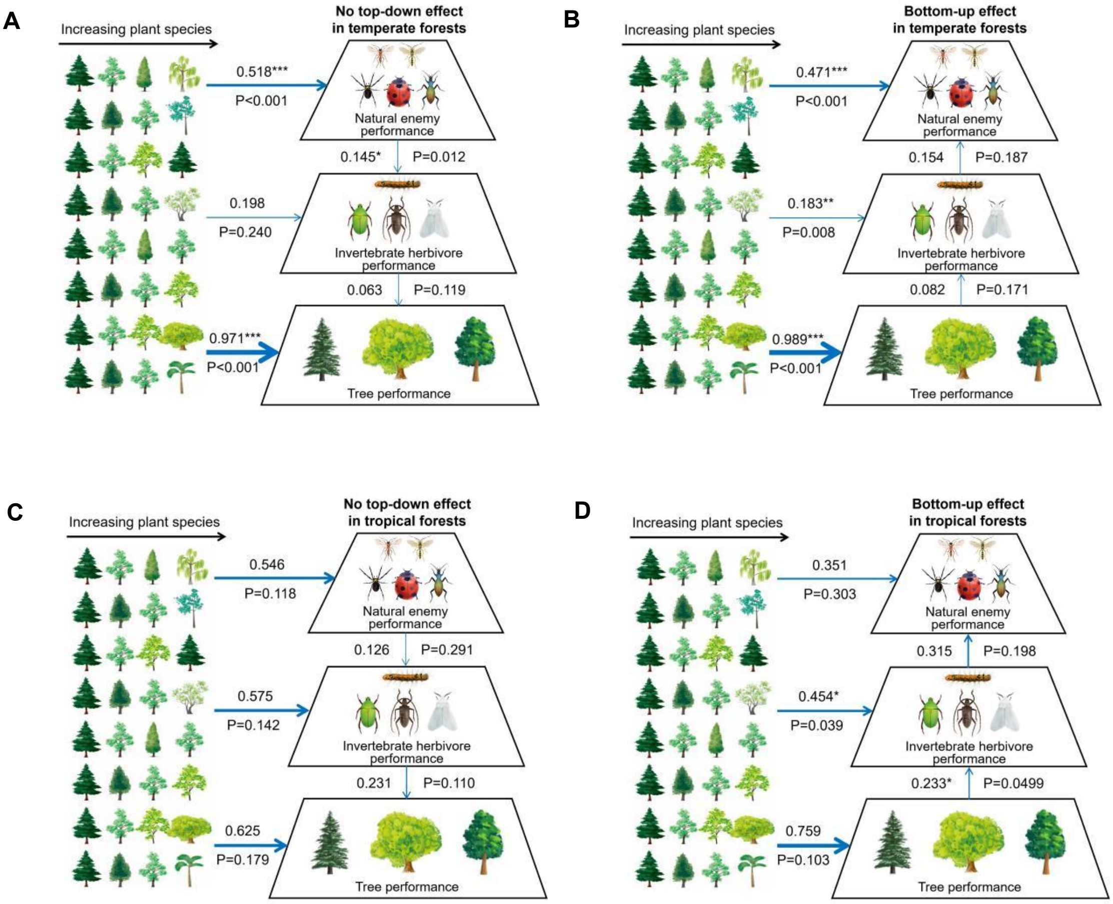

Fig. S16. Structural equation model to test for an association between an increase in plant diversity and the tri-trophic interactions of trees, invertebrate herbivores and their natural enemies in forests across different climatic regions. **A**, No top-down effect in temperate forests (N=291). **B**, Bottom-up effect in temperate forests (N=291). **C**, No top-down effect in tropical forests (N=42). **D**, Bottom-up effect in tropical forests (N=42). Tree performance includes the growth, reproduction and quality of trees, herbivore performance includes the abundance, damage and diversity of herbivores, and natural enemy performance includes the predator abundance, predation, predator diversity, parasitoid abundance, parasitism and parasitoid diversity. *P<0.05, **P<0.01, ***P<0.001. Blue and red arrows denote positive and negative relationships, respectively. Numbers next to each arrow are the estimated coefficients from structural equation models, and line width is proportional to the magnitude of the coefficients (table S9). Piecewise structural equation model (restricted maximum likelihood, REML) was used to test the effects of plant diversity (measured as the number of plant species generalize to binary variable) on the tri-trophic interactions of forests, invertebrate herbivores and their natural enemies. Photoshop8.0 was used to design the images.

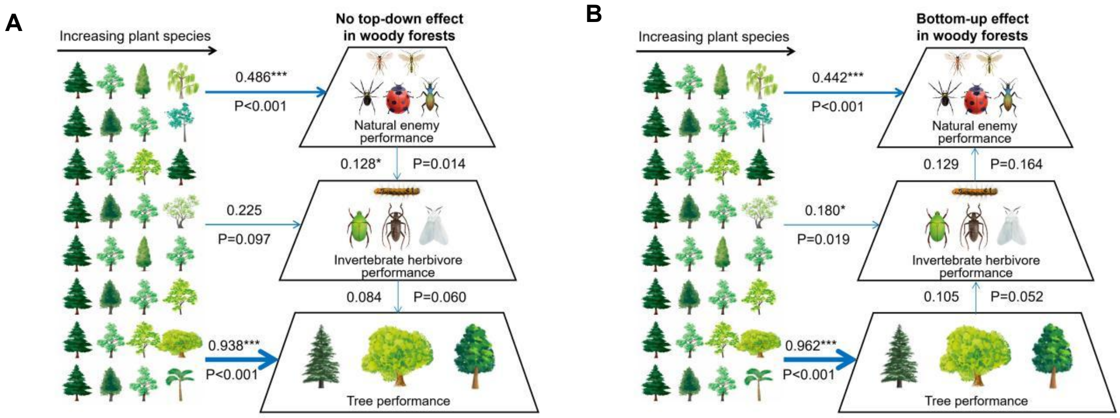

Fig. S17. Structural equation model to test for an association between an increase in plant diversity and the tri-trophic interactions of trees, invertebrate herbivores and their natural enemies in forests across different plant types. A, No top-down effect in woody plants in forests (N=333). **B**, Bottom-up effect in woody plants in forests (N=333). There is no data to test the effects of plant diversity on bottom-up effect in herbaceous plants in forests (N=0). Tree performance includes the growth, reproduction and quality of trees, herbivore performance includes the abundance, damage and diversity of herbivores, and natural enemy performance includes the predator abundance, predation, predator diversity, parasitoid abundance, parasitism and parasitoid diversity. *P<0.05, **P<0.01, ***P<0.001. Blue and red arrows denote positive and negative relationships, respectively. Numbers next to each arrow are the estimated coefficients from structural equation models, and line width is proportional to the magnitude of the coefficients (table S9). Piecewise structural equation model (restricted maximum likelihood, REML) was used to test the effects of plant diversity (measured as the number of plant species generalize to binary variable) on the tri-trophic interactions of forests, invertebrate herbivores and their natural enemies. Photoshop8.0 was used to design the images.

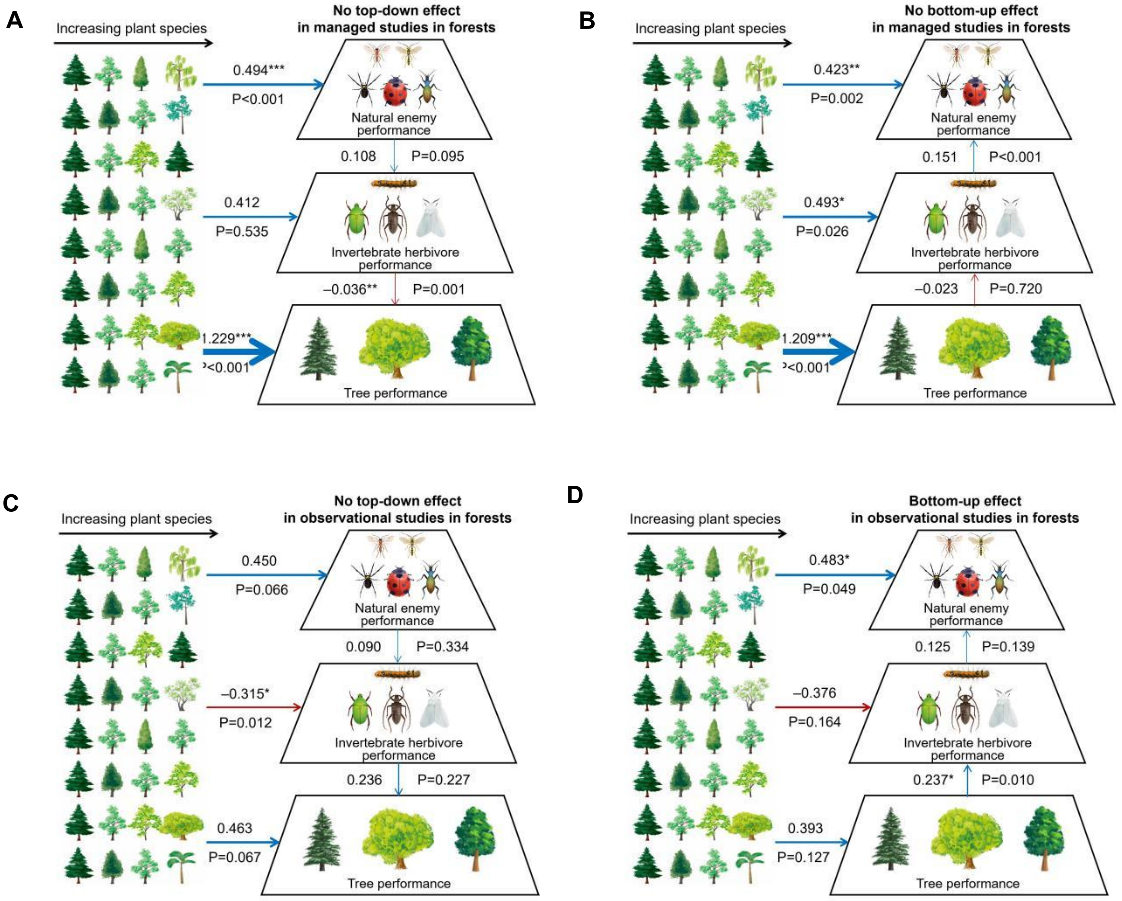

Fig. S18. Structural equation model to test for an association between an increase in plant diversity and the tri-trophic interactions of trees, invertebrate herbivores and their natural enemies in forests across different study types. **A**, No top-down effect in managed studies in forests (N=220). **B**, No bottom-up effect in managed studies in forests (N=220). **C**, No top-down effect in observational studies in forests (N=113). **D**, Bottom-up effect in observational studies in forests (N=113). Tree performance includes the growth, reproduction and quality of trees, herbivore performance includes the abundance, damage and diversity of herbivores, and natural enemy performance includes the predator abundance, predation, predator diversity, parasitoid abundance, parasitism and parasitoid diversity. *P<0.05, **P<0.01, ***P<0.001. Blue and red arrows denote positive and negative relationships, respectively. Numbers next to each arrow are the estimated coefficients from structural equation models, and line width is proportional to the magnitude of the coefficients (table S9). Piecewise structural equation model (restricted maximum likelihood, REML) was used to test the effects of plant diversity (measured as the number of plant species generalize to binary variable) on the tri-trophic interactions of forests, invertebrate herbivores and their natural enemies. Photoshop8.0 was used to design the images.

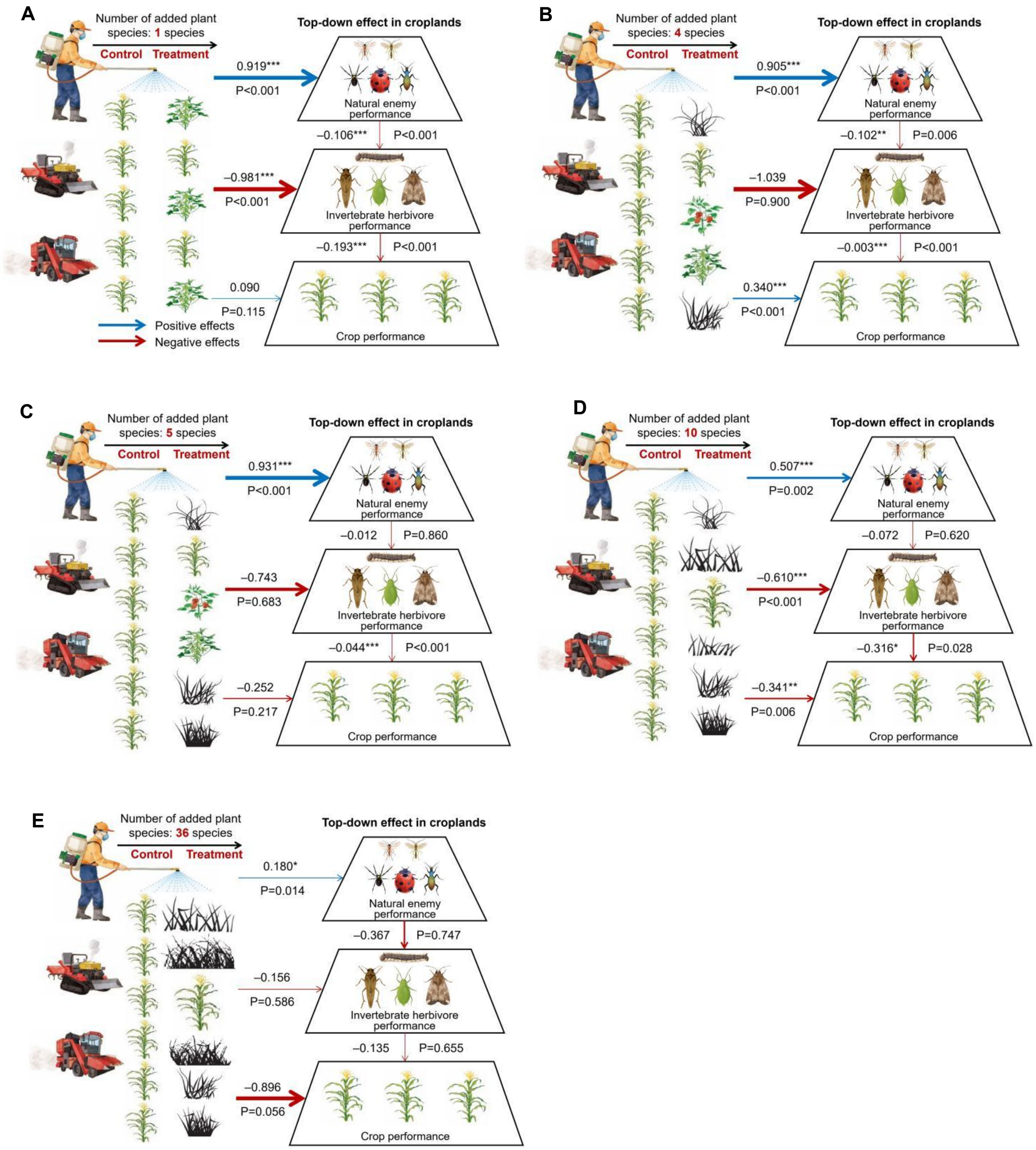

Fig. S19. Structural equation model shows that five gradients of number of added plant richness (i.e., 1, 4, 5, 10 and 36) result in top-down effect among plants, invertebrate herbivores and their natural enemies in croplands, respectively. A, One plant species gradient (N=1347). **B**, Four plant species gradients (N=292). **C**, Five plant species gradients (N=102). **D**, Ten plant species gradients (N=48). **E**, Thirty-six plant species gradients (N=16). All the plant species gradients did not trigger bottom-up effects in croplands. Crop performance includes the growth, reproduction and quality of crops, herbivore performance includes the abundance, damage and diversity of herbivores, and natural enemy performance includes the predator abundance, predation, predator diversity, parasitoid abundance, parasitism and parasitoid diversity. **P*<0.05, ***P*<0.01, ****P*<0.001. Blue and red arrows denote positive and negative relationships, respectively. Numbers next to each arrow are the estimated coefficients from structural equation models, and line width is proportional to the magnitude of the coefficients. The effects of each plant species gradient in organic and non-organic croplands on trophic effects were presented in Data S3. Photoshop8.0 was used to design the images.

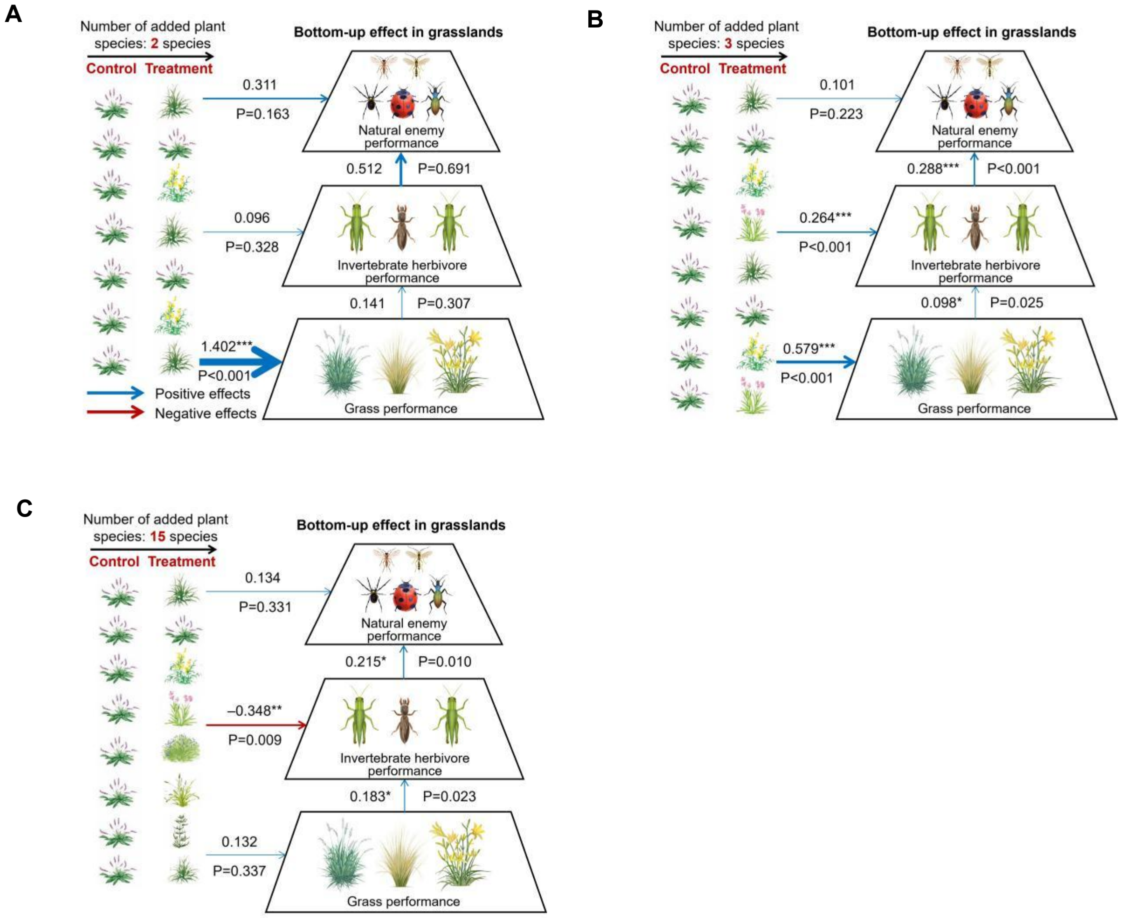

Fig. S20. Structural equation model shows that three gradients of number of added plant richness (i.e., 2, 3 and 15) result in bottom-up effect among plants, invertebrate herbivores and their natural enemies in grasslands, respectively. **A**, Two plant species gradient (N=48). **B**, Three plant species gradients (N=414). **C**, Fifteen plant species gradients (N=152). All the plant species gradients did not trigger top-down effect in grasslands. Grass performance includes the growth, reproduction and quality of grasses, herbivore performance includes the abundance, damage and diversity of herbivores, and natural enemy performance includes the predator abundance, predation, predator diversity, parasitoid abundance, parasitism and parasitoid diversity. *P<0.05, **P<0.01, ***P<0.001. Blue and red arrows denote positive and negative relationships, respectively. Numbers next to each arrow are the estimated coefficients from structural equation models, and line width is proportional to the magnitude of the coefficients (Data S4). Photoshop8.0 was used to design the images.

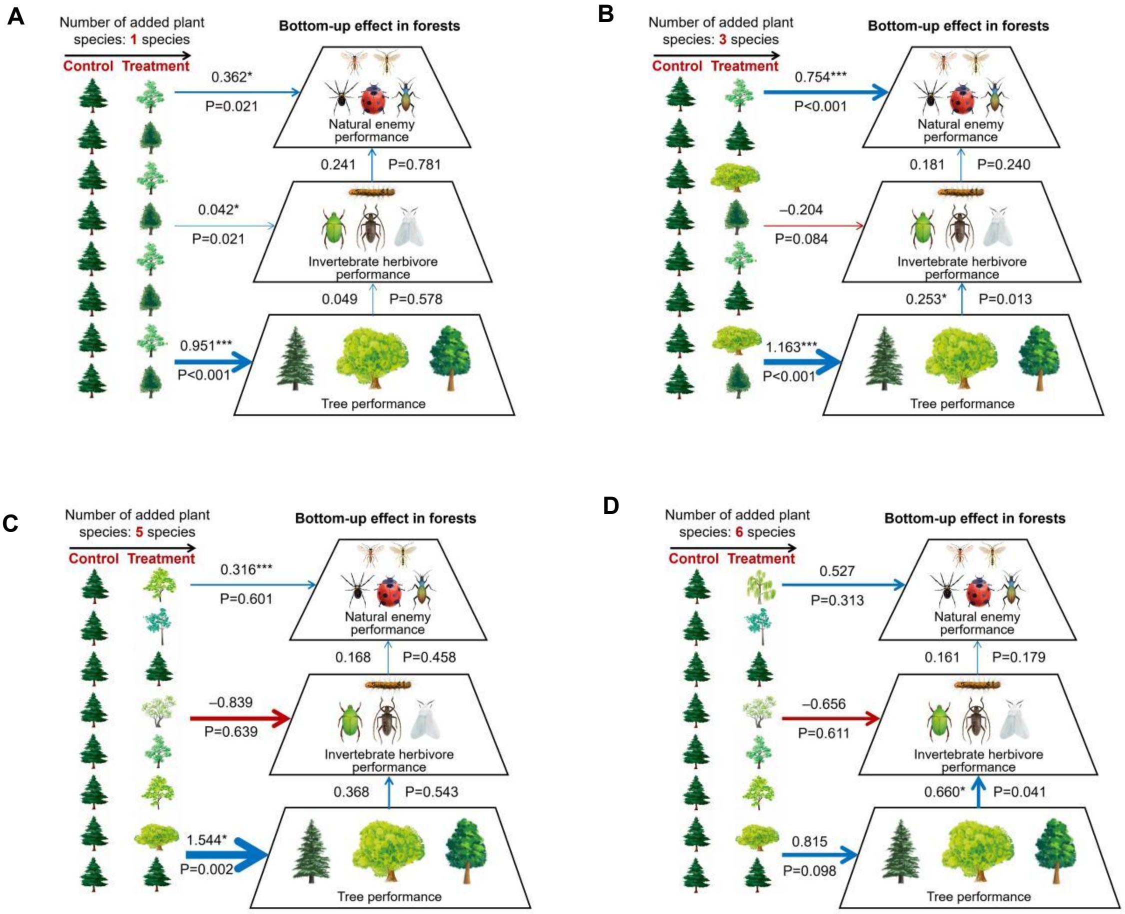

Fig. S21. Structural equation model shows that four gradients of number of added plant richness (i.e., 1, 3, 5 and 6) result in bottom-up effects among plants, invertebrate herbivores and their natural enemies in forests, respectively. A, One plant species gradient (N=108). **B**, Three plant species gradients (N=90). **C**, Five plant species gradients (N=10). **D**, Six plant species gradients (N=13). All the plant species gradients did not trigger top-down effects in forests. Tree performance includes the growth, reproduction and quality of trees, herbivore performance includes the abundance, damage and diversity of herbivores, and natural enemy performance includes the predator abundance, predation, predator diversity, parasitoid abundance, parasitism and parasitoid diversity. **P*<0.05, ***P*<0.01, ****P*<0.001. Blue and red arrows denote positive and negative relationships, respectively. Numbers next to each arrow are the estimated coefficients from structural equation models, and line width is proportional to the magnitude of the coefficients (Data S5). Photoshop8.0 was used to design the images.

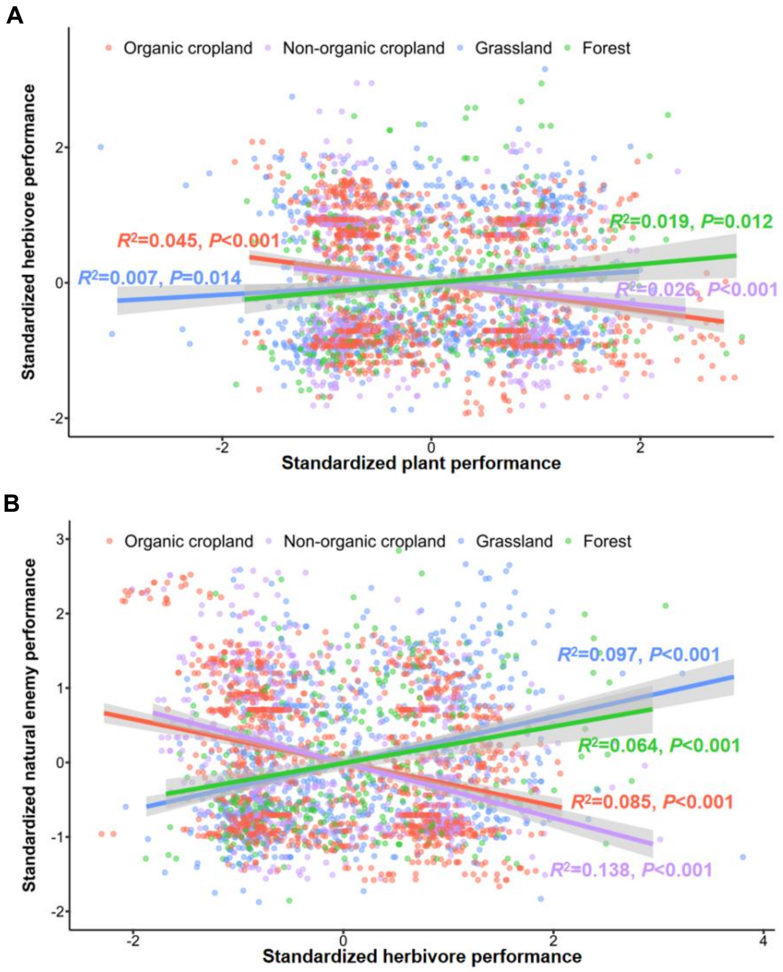

Fig. S22. Predictions from simple linear regressions on the relationships among standardized plant performance, standardized herbivore performance and standardized natural enemy performance. A, Relationship between standardized plant performance and standardized herbivore performance. **B**, Relationship between standardized herbivore performance and standardized natural enemy performance. The solid lines showed the fitted curve, the grey-shaded zone covered the 95% confidence interval, and the value of R^2 indicates the goodness-of-fit of linear regressions. The number of samples are N=887 from 49 experiments in non-organic croplands, N=1189 from 55 experiments in organic croplands, N=832 from 26 experiments in grasslands and N=333 from19 experiments in forests.

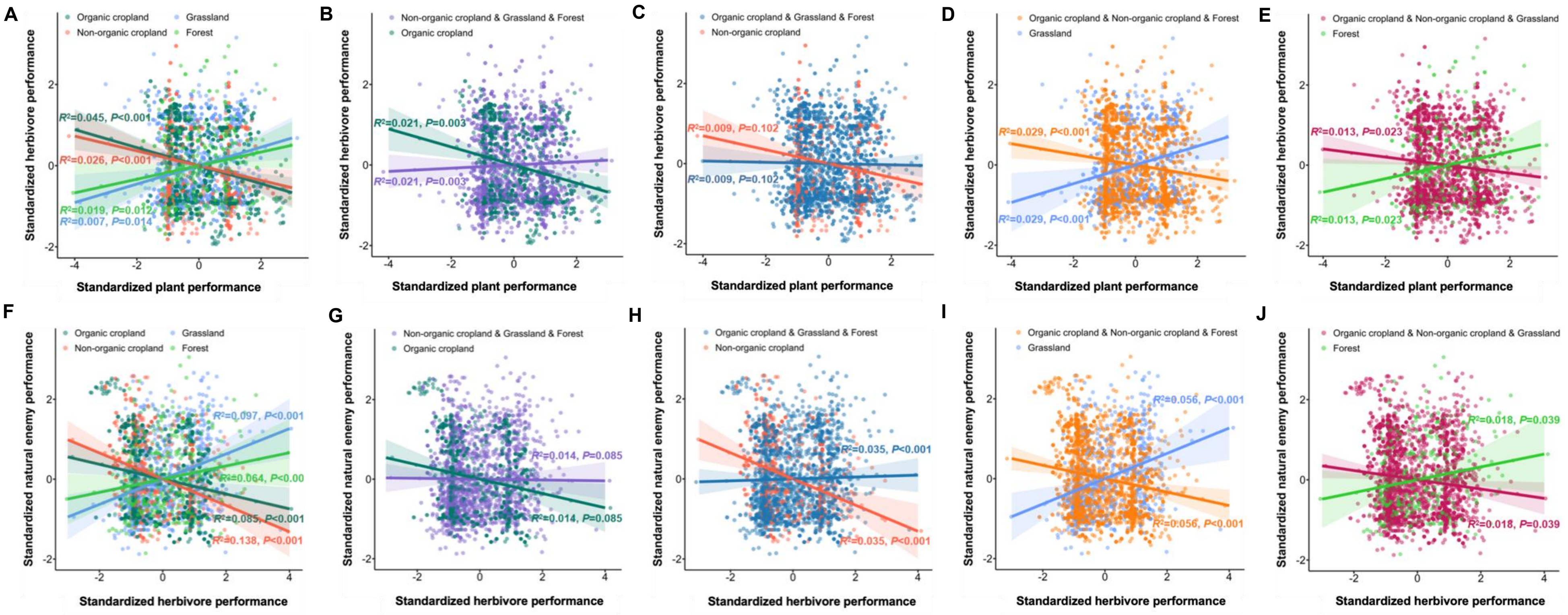

Fig. S23. Predictions from linear mixed-effects models on the relationships between invertebrate herbivore performance and plant performance as well as between natural enemy performance and invertebrate herbivore performance with interaction terms and a random effect for experiment identity. A, Relationship between standardized plant performance and standardized herbivore performance in interaction with ecosystem categories (organic cropland vs. non-organic cropland vs. grassland vs. forest). **B**, Relationship between standardized plant performance and standardized herbivore performance with two categories (organic cropland vs. non-organic cropland & grassland & forest). **C**, Relationship between standardized plant performance and standardized herbivore performance with two categories (non-organic cropland vs. organic cropland & grassland & forest). **D**, Relationship between standardized plant performance and standardized herbivore performance with two categories (grassland vs. organic cropland & non-organic cropland & forest). **E**, Relationship between standardized plant performance and standardized herbivore performance with two categories (forest vs. organic cropland & non-organic cropland & grassland). **F**, Relationship between standardized natural enemy performance and standardized herbivore performance in interaction with ecosystem categories (organic cropland vs. non-organic cropland vs. grassland vs. forest). **G**, Relationship between standardized natural enemy performance and standardized herbivore performance with two categories (organic cropland vs. non-organic cropland & grassland & forest). **H**, Relationship between standardized natural enemy performance and standardized herbivore performance with two categories (non-organic cropland vs. organic cropland & grassland & forest). **I**, Relationship between standardized natural enemy performance and standardized herbivore performance with two categories (grassland vs. organic cropland & non-organic cropland & forest). **J**, Relationship between standardized natural enemy performance and standardized herbivore performance with two categories (forest vs. organic cropland & non-organic cropland & grassland). The solid lines show fixed-effect predictions from mixed models, the grey-shaded zone shows the 95% confidence interval. The number of samples was N=3241 from 149 experiments (see Data S6, 7).

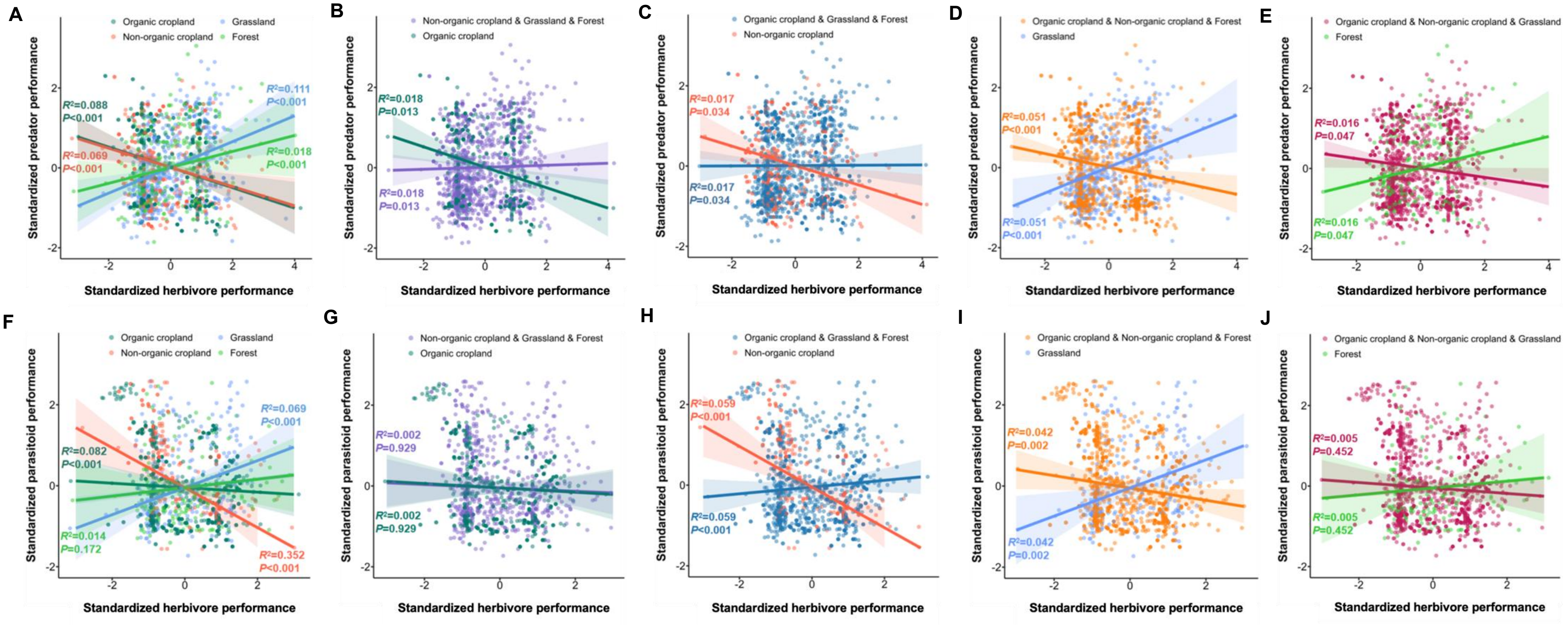

Fig. S24. Predictions from linear mixed-effects models on the relationships between herbivore performance and predator/parasitoid performance with interaction terms and a random effect for experiment identity. A, Relationship between standardized predator performance and standardized herbivore performance with four ecosystem categories (non-organic cropland vs. organic cropland vs. grassland vs. forest). **B**, Relationship between standardized predator performance and standardized herbivore performance with two categories (cropland vs. non-organic cropland & grassland & forest). **C**, Relationship between standardized predator performance and standardized herbivore performance with two categories (non-organic cropland vs. organic cropland & grassland & forest). **D**, Relationship between standardized predator performance and standardized herbivore performance with two categories (grassland vs. organic cropland & non-organic cropland & forest). **E**, Relationship between standardized predator performance and standardized herbivore performance with two categories (forest vs. organic cropland & non-organic cropland & grassland). **F**, Relationship between standardized parasitoid performance and standardized herbivore performance with four ecosystem categories (non-organic cropland vs. organic cropland vs. grassland vs. forest). **G**, Relationship between standardized parasitoid performance and standardized herbivore performance with two categories (organic cropland vs. non-organic cropland & grassland & forest). **H**, Relationship between standardized parasitoid performance and standardized herbivore performance with two categories (non-organic cropland vs. Organic cropland & grassland & forest). **I**, Relationship between standardized parasitoid performance and standardized herbivore performance with two categories (grassland vs. organic cropland & non-organic cropland & forest). **J**, Relationship between standardized parasitoid performance and standardized herbivore performance with two categories (forest vs. organic cropland & non-organic cropland & grassland). The solid lines showed the fitted curve, the grey-shaded zone covered the 95% confidence interval. The number of samples are N=1997 from 135 experiments for predators, and N=1244 from 49 experiments for parasitoids (see Data S7).

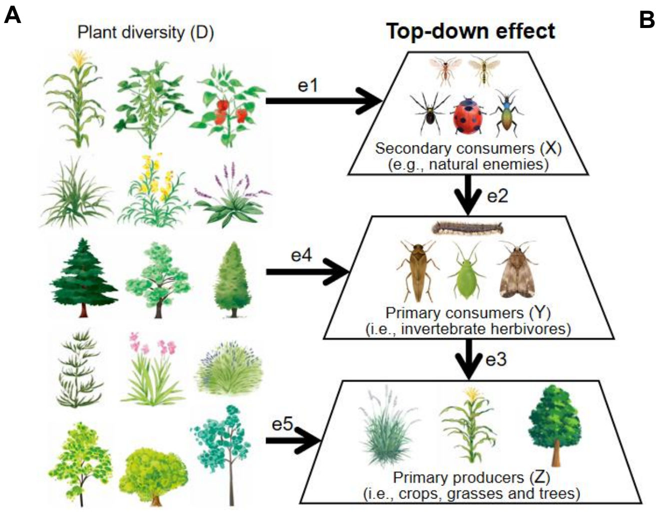

Fig. S25. Mediation analysis among tri-trophic groups. In the analysis of top-down effects, derived by mediation analysis, A statistical procedure to test whether the effect of an independent variable D (i.e., plant diversity) on A dependent variable Y (i.e., herbivore performance) (D→Y) is at least partly explained via the inclusion of A mediator variable X (i.e., secondary consumers including natural enemy performance, predator performance and parasitoid performance) (D→X→Y). The effect of an independent variable D on A dependent variable Z (i.e., plant performance) is at least partly explained via the inclusion of A mediator variable Y (D→Y→Z). Similarly, in analyses of bottom-up effects, the effect of an independent variable D' (i.e., plant diversity) on A dependent variable X' (i.e., secondary consumers including natural enemy performance, predator performance and parasitoid performance) (D'→X') is at least partly explained via the inclusion of A mediator variable Y' (i.e., herbivore performance) (D'→Y'→X') and the effect of an independent variable D' on A dependent variable Y' is at least partly explained via the inclusion of A mediator variable Z' (i.e., plant performance) (D'→Z'→Y'). **A**, In our top-down analysis, the five causal paths e1, e2, e3, e4, and e5 correspond to the effect of plant diversity on secondary consumers (i.e., natural enemy, predator or parasitoid performance), the effect of secondary consumer performance on herbivore performance, the effect of plant diversity on herbivore performance accounting for secondary consumer performance, the effect of herbivore performance on plant performance and the effect of plant diversity on plant performance accounting for herbivore performance respectively. The five causal paths correspond to parameters from three regression models, one in which secondary consumer performance is the outcome and plant diversity is the predictor, on in which herbivore performance is the outcome and plant diversity and secondary consumer performance are the simultaneous predictors, one in which plant performance is the outcome and herbivore performance is the predictors and one in which plant performance is the outcome and plant diversity and herbivore performance are the simultaneous predictors. From these parameters, we can calculate the mediation effect (the product e2×e3 and e3×e4) and total effect (e2×e3+ e1) of plant diversity on herbivore performance and total effect (e3×e4+ e5) of plant diversity on plant performance. **B**, In our bottom-up analysis, the five causal paths e1', e2', e3', e4', and e5' correspond to the effect of plant diversity on secondary consumer performance, the effect of herbivore performance on secondary consumer performance, the effect of plant diversity on secondary consumer performance accounting for herbivore performance, the effect of plant performance on herbivore performance and the effect of plant diversity on herbivore performance accounting for plant performance respectively. The five causal paths correspond to parameters from three regression models, one in which secondary consumer performance is the outcome and herbivore performance is the predictor, one in which secondary consumer performance is the outcome and plant diversity and herbivore performance are the simultaneous predictors, on in which plant performance is the outcome and plant diversity is the predictors and one in which herbivore performance is the outcome and plant diversity and plant performance are the simultaneous predictors. From these parameters, we can calculate the mediation effect (the product e1'×e2' and e4'×e5') and total effect (e1'×e2'+ e1') of plant diversity on secondary consumer performance and total effect (e4'×e5'+ e3') of plant diversity on herbivore performance. Photoshop8.0 was used to design the images.

Table S1. The effects of binary plant diversity on the five trophic groups of plants, invertebrate herbivores, invertebrate natural enemies, invertebrate predators and invertebrate parasitoids in different ecosystems. Plant performance includes the growth, reproduction and quality of plants, herbivore performance includes the abundance, damage and diversity of herbivores, and natural enemy performance includes the predator abundance, predation, predator diversity, parasitoid abundance, parasitism and parasitoid diversity. Predator performance includes predator abundance, predation and predator diversity. Parasitoid performance includes parasitoid abundance, parasitism and parasitoid diversity. Zero-or-one inflated beta regression was used to test whether the sets of values in two groups are significantly different ($P<0.05$). Each test is two-sided and the original P value is reported with no multiple comparisons. Increased percentage of relative performance = (data of relative performance in treatment－data of relative performance in control) / data of relative performance in control×100%. Degree of freedom, 95% confidence interval and the number of studies and observations available for each predictor category are also presented.

Ecosystem type	Category	Number of observations	Number of studies	Increased percentage of relative performance	df	t-value	CI_{lb}	CI_{ub}	P-value
Global	Total relative response of plant performance	2027	149	6.7281%	2025	-0.9166	-0.1640	0.0579	0.3594
	Total relative response of invertebrate herbivore performance	2214	149	-23.0285%	2212	-3.0890	-0.2794	-0.0623	0.0020
	Total relative response of natural enemy performance	2363	149	34.8446%	2361	9.4929	0.3846	0.5827	5.2910×10^{-21}
	Total relative response of predator performance	1429	135	25.3922%	1427	6.7693	0.3075	0.5546	1.8830×10^{-11}
	Total relative response of parasitoid performance	934	49	54.1190%	932	7.8711	0.4587	0.7634	9.7070×10^{-15}
	Plant growth response	949	71	5.8315%	947	-0.4239	-0.1795	0.1174	0.6718
	Plant reproduction response	940	90	8.4838%	938	-0.0620	-0.1680	0.1540	0.9506
	Plant quality response	138	3	-0.7994%	136	-1.2336	-0.7246	0.1580	0.2195
	Herbivore abundance	1559	136	-20.1761%	1557	-2.2766	-0.2781	-0.0215	0.0230
	Herbivore damage	473	30	-45.7442%	471	-4.1221	-0.7149	-0.2569	4.4330×10^{-5}
	Herbivore diversity	182	12	20.2203%	180	2.8952	0.1497	0.7559	0.0043
	Predator abundance	1189	120	28.0808%	1187	6.1285	0.2952	0.5716	1.2040×10^{-9}
	Predation	49	14	0.6861%	47	0.1695	-0.7658	0.9217	0.8661
	Predator diversity	191	17	13.6147%	189	1.7865	-0.0233	0.5482	0.0756
	Parasitoid abundance	510	42	28.6013%	508	2.0547	0.0185	0.4543	0.0404
	Parasitoid diversity	85	5	30.9998%	83	2.8386	0.2136	1.1010	0.0057
	Parasitism	339	13	113.1257%	337	8.2989	0.7055	1.1420	2.5220×10^{-15}
Organic croplands	Total relative response of plant performance	760	55	17.1057%	758	0.9096	-0.0905	0.2476	0.3633
	Total relative response of invertebrate herbivore performance	926	55	-40.0640%	924	-9.5787	-0.9077	-0.5986	8.6910×10^{-21}
	Total relative response of natural enemy performance	775	55	56.0050%	773	5.3814	0.3279	0.7029	9.8010×10^{-8}
	Total relative response of predator performance	376	47	37.9803%	374	3.8530	0.2765	0.8340	1.3710×10^{-4}
	Total relative response of parasitoid performance	399	23	87.1545%	397	6.5778	0.5210	0.9599	1.5010×10^{-10}
	Plant growth response	162	16	12.8326%	160	0.7871	-0.2524	0.5867	0.4324
	Plant reproduction response	580	45	18.8590%	578	0.7600	-0.1214	0.2641	0.4475
	Plant quality response	18	1	18.9357%	16	3.7212	0.4408	1.4120	1.5630×10^{-3}
	Herbivore abundance	591	45	-30.8538%	589	-5.8000	-0.7543	-0.3663	1.0810×10^{-8}
	Herbivore damage	335	22	-56.4296%	333	-9.6715	-1.3860	-0.9243	1.1270×10^{-19}
	Herbivore diversity	0	0	NA	NA	NA	NA	NA	NA
	Predator abundance	344	45	42.0401%	342	3.8157	0.2719	0.8698	1.6100×10^{-4}
	Predation	14	2	3.5425%	12	-0.4827	-2.4410	1.6630	0.6368
	Predator diversity	18	5	9.7122%	16	0.6834	-0.9978	1.9170	0.5030
	Parasitoid abundance	135	19	34.7944%	133	1.3023	-0.1560	0.7455	0.1950
	Parasitoid diversity	0	0	NA	NA	NA	NA	NA	NA
	Parasitism	264	8	159.0850%	262	9.7666	0.8935	1.3400	1.9590×10^{-19}
	Total relative response of plant performance	571	49	0.5733%	569	-0.7725	-0.3143	0.1342	0.4402
	Total relative response of invertebrate herbivore performance	419	49	-30.0111%	417	-3.1408	-0.7388	-0.1757	1.8050×10^{-3}
	Total relative response of natural enemy performance	533	49	42.9074%	531	6.5263	0.5099	0.9413	1.5710×10^{-10}
	Total relative response of predator performance	373	48	34.3814%	371	3.9843	0.2711	0.8011	8.1410×10^{-5}

	Total relative response of parasitoid performance	160	11	68.8213%	158	5.6236	0.7597	1.5840	8.1500×10^{-8}
	Plant growth response	218	13	1.4436%	216	0.4114	-0.2390	0.3524	0.6812
Non-organic croplands	Plant reproduction response	281	42	-0.8628%	279	-1.2105	-0.4779	0.1168	0.2271
	Plant quality response	72	1	5.5776%	70	-0.5742	-0.8179	0.4223	0.5676
	Herbivore abundance	361	49	-31.9788%	359	-2.9816	-0.8244	-0.1698	3.0620×10^{-3}
	Herbivore damage	34	5	-29.5984%	32	0.4207	-0.8819	1.2810	0.6766
	Herbivore diversity	24	1	-0.1575%	22	0.2705	-0.4958	0.6890	0.7891
	Predator abundance	314	38	43.9096%	312	4.4380	0.3563	0.9108	1.2580×10^{-5}
	Predation	23	10	-21.3380%	21	-0.2030	-1.5870	1.2470	0.8409
	Predator diversity	36	3	8.4340%	34	-1.2086	-0.7606	0.1944	0.2347
	Parasitoid abundance	126	9	76.9780%	124	5.6315	0.8162	1.7010	1.1070×10^{-7}
	Parasitoid diversity	0	0	NA	NA	NA	NA	NA	NA
	Parasitism	34	3	46.6527%	32	1.7613	-0.0327	2.0140	0.0872
	Total relative response of plant performance	454	26	17.5929%	452	4.4128	0.2637	0.6939	1.2760×10^{-5}
	Total relative response of invertebrate herbivore performance	603	26	32.1724%	601	5.4622	0.3428	0.7374	6.8850×10^{-8}
	Total relative response of natural enemy performance	752	26	17.1554%	750	3.1011	0.1189	0.5093	0.0020
	Total relative response of predator performance	498	24	19.9006%	496	3.0863	0.1275	0.6114	0.0021
	Total relative response of parasitoid performance	254	10	12.1321%	252	1.2520	-0.1247	0.5274	0.2117
	Plant growth response	386	24	29.4568%	384	4.3976	0.2876	0.7349	1.4170×10^{-5}
Grasslands	Plant reproduction response	68	2	-14.1332%	66	0.0429	-0.6741	0.6585	0.9659
	Plant quality response	0	0	NA	NA	NA	NA	NA	NA
	Herbivore abundance	458	26	29.7738%	456	3.8495	0.2223	0.6766	0.0001
	Herbivore damage	80	1	12.4125%	78	3.9077	0.5797	1.7590	0.0002
	Herbivore diversity	65	3	77.6982%	63	3.3719	0.3406	1.3200	0.0013
	Predator abundance	433	24	16.6354%	431	2.6780	0.1028	0.6109	0.0077
	Predation	0	0	NA	NA	NA	NA	NA	NA
	Predator diversity	65	3	41.5379%	63	1.9478	-0.0069	1.0690	0.0558
	Parasitoid abundance	204	10	5.8785%	202	0.5449	-0.2794	0.5086	0.5864
	Parasitoid diversity	50	2	38.7495%	48	2.8266	0.2718	1.4820	0.0067
	Parasitism	0	0	NA	NA	NA	NA	NA	NA
	Total relative response of plant performance	242	19	10.5085%	240	-0.8242	-0.5079	0.2142	0.4107
	Total relative response of invertebrate herbivore performance	266	19	0.2787%	264	0.5591	-0.2327	0.4105	0.5765
	Total relative response of natural enemy performance	303	19	15.0359%	301	1.3961	-0.0843	0.4640	0.1637
	Total relative response of predator performance	182	16	22.2258%	180	3.0852	0.1723	0.7788	0.0024
	Total relative response of parasitoid performance	121	5	13.1340%	119	0.2304	-0.4132	0.5036	0.8182
	Plant growth response	183	18	1.9080%	181	-1.6436	-0.7203	0.0664	0.1020
	Plant reproduction response	11	1	38.3621%	9	0.5378	-1.7630	2.9930	0.6014
Forests	Plant quality response	48	1	4.7662%	46	-0.7205	-0.9481	0.4607	0.4747
	Herbivore abundance	149	16	-6.3674%	147	-0.1881	-0.4785	0.4093	0.8510
	Herbivore damage	24	2	-0.1172%	22	0.0338	-0.9347	0.8545	0.9733
	Herbivore diversity	93	8	15.9012%	91	1.8285	-0.0445	0.9226	0.0707
	Predator abundance	98	13	23.1376%	96	1.9943	0.0074	0.9036	0.0489
	Predation	12	2	59.3625%	10	0.7961	-0.4976	1.5860	0.4414
	Predator diversity	72	6	10.5882%	70	1.2096	-0.1736	0.6802	0.2304
	Parasitoid abundance	45	4	43.2013%	43	1.3189	-0.1968	1.0980	0.1939
	Parasitoid diversity	35	3	18.2988%	33	0.8927	-0.4393	1.0870	0.3781
	Parasitism	41	2	-19.9054%	39	-1.3519	-1.7570	0.2883	0.1838

Table S2. The effects of binary plant diversity on the five trophic groups of plants, invertebrate herbivores, invertebrate natural enemies, invertebrate predators and invertebrate parasitoids in temperate or tropical regions. Plant performance includes the growth, reproduction and quality of plants, herbivore performance includes the abundance, damage and diversity of herbivores, and natural enemy performance includes the predator abundance, predation, predator diversity, parasitoid abundance, parasitism and parasitoid diversity. Predator performance includes predator abundance, predation and predator diversity. Parasitoid performance includes parasitoid abundance, parasitism and parasitoid diversity. Zero-or-one inflated beta regression was used to test whether the sets of values in two groups are significantly different ($P<0.05$). Each test is two-sided and the original P value is reported with no multiple comparisons. Increased percentage of relative performance = (data of relative performance in treatment－data of relative performance in control) / data of relative performance in control×100%. Degree of freedom, 95% confidence interval and the number of studies and observations available for each predictor category are also presented.

Climatic region type	Category	Number of observations	Number of studies	Increased percentage of relative performance	df	t-value	CI_{lb}	CI_{ub}	P-value
Temperate regions	Total relative response of plant performance	1759	124	6.1053%	1757	-0.8688	-0.1700	0.0614	0.3851
	Total relative response of invertebrate herbivore performance	1979	124	-20.8809%	1977	-3.0704	-0.2830	-0.0639	0.0022
	Total relative response of natural enemy performance	2079	124	34.5582%	2077	9.0401	0.3825	0.5979	3.4910×10^{-19}
	Total relative response of predator performance	1274	115	22.2618%	1272	5.8496	0.2511	0.5071	6.2570×10^{-9}
	Total relative response of parasitoid performance	805	38	60.6776%	803	7.9582	0.4941	0.8236	5.9220×10^{-15}
	Plant growth response	856	63	5.5045%	854	0.2494	-0.1415	0.1760	0.8031
	Plant reproduction response	765	70	7.2131%	763	-0.9498	-0.2612	0.0903	0.3425
	Plant quality response	138	3	-0.7994%	136	-1.2044	-0.7270	0.1762	0.2305
	Herbivore abundance	1387	113	-16.9211%	1385	-1.7317	-0.2438	0.0102	0.0835
	Herbivore damage	421	22	-48.1229%	419	-4.9382	-0.7983	-0.3446	1.1390×10^{-6}
	Herbivore diversity	171	11	21.6090%	169	2.5508	0.0913	0.7288	0.0116
	Predator abundance	1034	100	24.6165%	1032	5.3331	0.2429	0.5207	1.1850×10^{-7}
	Predation	49	14	0.6861%	47	0.1891	-0.7306	0.8795	0.8508
	Predator diversity	191	17	13.6147%	189	1.7773	-0.0406	0.5490	0.0771
	Parasitoid abundance	421	31	30.2262%	419	2.2603	0.0388	0.5026	0.0243
	Parasitoid diversity	74	4	34.8035%	72	3.1878	0.2954	1.2280	0.0021
	Parasitism	310	8	118.0593%	308	8.4712	0.7194	1.1550	9.9190×10^{-16}
Tropical regions	Total relative response of plant performance	268	25	12.4002%	266	1.0275	-0.1573	0.4904	0.3051
	Total relative response of invertebrate herbivore performance	235	25	-37.1802%	233	-1.6099	-1.1920	0.0972	0.1088
	Total relative response of natural enemy performance	284	25	38.7433%	282	3.7594	0.2749	0.8774	0.0002
	Total relative response of predator performance	155	20	53.8580%	153	3.6366	0.4525	1.4250	0.0004
	Total relative response of parasitoid performance	129	11	21.9280%	127	1.1736	-0.1510	0.6210	0.2427
	Plant growth response	93	8	12.6896%	91	-0.7788	-0.8521	0.4134	0.4381
	Plant reproduction response	175	20	11.8683%	173	1.8681	-0.0252	0.7558	0.0634
	Plant quality response	0	0	NA	NA	NA	NA	NA	NA
	Herbivore abundance	172	23	-41.1485%	170	-1.7990	-1.4620	0.0690	0.0738
	Herbivore damage	52	8	-29.5821%	50	-0.3275	-1.4640	0.9239	0.7446
	Herbivore diversity	11	1	-21.8519%	9	0.2568	-81.9500	108.0000	0.8021
	Predator abundance	155	20	53.8580%	153	3.6762	0.4229	1.4170	0.0003
	Predation	0	0	NA	NA	NA	NA	NA	NA
	Predator diversity	0	0	NA	NA	NA	NA	NA	NA
	Parasitoid abundance	89	11	26.7709%	87	0.8597	-0.2677	0.7093	0.3923
	Parasitoid diversity	11	1	24.2857%	9	0.2634	-2.0190	2.5230	0.7971
	Parasitism	29	5	17.2962%	27	0.2379	-0.6983	0.8549	0.8136

Table S3. The effects of binary plant diversity on the five trophic groups of plants, invertebrate herbivores, invertebrate natural enemies, invertebrate predators and invertebrate parasitoids for herbaceous and woody plants. Plant performance includes the growth, reproduction and quality of plants, herbivore performance includes the abundance, damage and diversity of herbivores, and natural enemy performance includes the predator abundance, predation, predator diversity, parasitoid abundance, parasitism and parasitoid diversity. Predator performance includes predator abundance, predation and predator diversity. Parasitoid performance includes parasitoid abundance, parasitism and parasitoid diversity. Zero-or-one inflated beta regression was used to test whether the sets of values in two groups are significantly different ($P<0.05$). Each test is two-sided and the original P value is reported with no multiple comparisons. Increased percentage of relative performance = (data of relative performance in treatment—data of relative performance in control) / data of relative performance in control×100%. Degree of freedom, 95% confidence interval and the number of studies and observations available for each predictor category are also presented.

Plant type	Category	Number of observations	Number of studies	Increased percentage of relative performance	df	t-value	CI_{lb}	CI_{ub}	P-value
Herbaceous plants	Total relative response of plant performance	1304	98	11.3052%	1302	2.1480	0.0098	0.2580	0.0319
	Total relative response of invertebrate herbivore performance	1679	98	-22.807%	1677	-2.8680	-0.2976	-0.0571	0.0042
	Total relative response of natural enemy performance	1668	98	32.3628%	1666	7.1098	0.3310	0.5787	1.7170×10^{-12}
	Total relative response of predator performance	984	87	19.1883%	982	3.5985	0.1307	0.4391	0.0003
	Total relative response of parasitoid performance	684	29	55.9378%	682	7.0173	0.4747	0.8376	5.4540×10^{-12}
	Plant growth response	550	42	12.6908%	548	3.5167	0.1528	0.5418	0.0005
	Plant reproduction response	754	63	10.4943%	752	-0.2893	-0.2035	0.1460	0.7724
	Plant quality response	0	0	NA	NA	NA	NA	NA	NA
	Herbivore abundance	1170	94	-16.1336%	1168	-0.9604	-0.2089	0.0718	0.3370
	Herbivore damage	420	17	-50.3426%	418	-4.5364	-0.7900	-0.3082	7.4790×10^{-6}
	Herbivore diversity	89	4	27.0446%	87	2.1901	0.0612	0.9392	0.0311
	Predator abundance	836	75	21.4329%	834	3.5063	0.1323	0.4763	0.0005
	Predation	37	12	-12.6239%	35	-0.2468	-1.1350	0.8654	0.8064
	Predator diversity	111	7	18.0103%	109	0.9958	-0.2110	0.5941	0.3215
	Parasitoid abundance	336	23	13.5285%	334	0.4860	-0.2256	0.3663	0.6273
	Parasitoid diversity	50	2	38.7495%	48	2.8234	0.2616	1.4890	0.0068
	Parasitism	298	11	128.2919%	296	8.3843	0.7540	1.2270	2.0560×10^{-15}
Woody plants	Total relative response of plant performance	723	51	-1.4578%	721	-2.5568	-0.4728	-0.0609	0.0108
	Total relative response of invertebrate herbivore performance	535	51	-24.5764%	533	-1.7843	-0.4765	0.0257	0.0749
	Total relative response of natural enemy performance	695	51	40.3181%	693	6.8279	0.3862	0.7008	1.8810×10^{-11}
	Total relative response of predator performance	445	48	40.288%	443	7.3812	0.5429	0.9328	7.7700×10^{-13}
	Total relative response of parasitoid performance	250	20	45.9610%	248	2.9057	0.1194	0.6083	0.0040
	Plant growth response	399	29	-3.2977%	397	-3.0901	-0.6223	-0.1398	0.0021
	Plant reproduction response	186	27	0.0488%	184	-0.4942	-0.4349	0.2525	0.6217
	Plant quality response	138	3	-0.7994%	136	-1.2196	-0.7217	0.1668	0.2247
	Herbivore abundance	389	42	-31.1463%	387	-2.8910	-0.7709	-0.1447	0.0041
	Herbivore damage	53	13	-10.6481%	51	0.3297	-0.6019	0.7914	0.7429
	Herbivore diversity	93	8	15.901%	91	1.7517	-0.0400	0.8908	0.0831
	Predator abundance	353	45	44.9789%	351	6.2918	0.4995	0.9542	9.2970×10^{-10}
	Predation	12	2	59.3625%	10	0.8226	-0.4561	1.5360	0.4268
	Predator diversity	80	10	4.7751%	78	0.7226	-0.2785	0.5975	0.4720
	Parasitoid abundance	174	19	64.5464%	172	3.5027	0.2314	0.8305	0.0006
	Parasitoid diversity	35	3	18.2988%	33	0.9211	-0.4141	1.0850	0.3633
	Parasitism	41	2	-19.9054%	39	-1.3667	-1.7010	0.2922	0.1792

Table S4. The effects of binary plant diversity on the five trophic groups of plants, invertebrate herbivores, invertebrate natural enemies, invertebrate predators and invertebrate parasitoids in managed or observational studies. Plant performance includes the growth, reproduction and quality of plants, herbivore performance includes the abundance, damage and diversity of herbivores, and natural enemy performance includes the predator abundance, predation, predator diversity, parasitoid abundance, parasitism and parasitoid diversity. Predator performance includes predator abundance, predation and predator diversity. Parasitoid performance includes parasitoid abundance, parasitism and parasitoid diversity. Zero-or-one inflated beta regression was used to test whether the sets of values in two groups are significantly different ($P<0.05$). Each test is two-sided and the original P value is reported with no multiple comparisons. Increased percentage of relative performance = (data of relative performance in treatment－data of relative performance in control) / data of relative performance in control×100%. Degree of freedom, 95% confidence interval and the number of studies and observations available for each predictor category are also presented.

Study type	Category	Number of observations	Number of studies	Increased percentage of relative performance	df	t-value	CI_{lb}	CI_{ub}	P-value
Managed studies	Total relative response of plant performance	1773	98	8.098%	1771	-0.7753	-0.1618	0.0713	0.4383
	Total relative response of invertebrate herbivore performance	1916	98	-24.1304%	1914	-3.1900	-0.3061	-0.0711	0.0014
	Total relative response of natural enemy performance	2027	98	38.7257%	2025	9.4462	0.4215	0.6465	9.3200×10^{-21}
	Total relative response of predator performance	1215	91	28.1523%	1213	7.0013	0.3469	0.6182	4.1850×10^{-12}
	Total relative response of parasitoid performance	812	33	58.4004%	810	7.6518	0.4784	0.8115	5.6190×10^{-14}
	Plant growth response	788	47	8.9865%	786	0.5708	-0.1209	0.2080	0.5683
	Plant reproduction response	847	63	8.078%	845	-0.7178	-0.2308	0.1094	0.4731
	Plant quality response	138	3	-0.7994%	136	-1.2673	-0.7076	0.1532	0.2072
	Herbivore abundance	1354	94	-20.6489%	1352	-1.9785	-0.2769	-0.0057	0.0481
	Herbivore damage	434	19	-47.7975%	432	-4.1686	-0.7452	-0.2706	3.7010×10^{-5}
	Herbivore diversity	128	8	28.0231%	126	2.9551	0.1782	0.8624	0.0037
	Predator abundance	1041	87	29.9091%	1039	6.6114	0.3549	0.6492	6.0710×10^{-11}
	Predation	26	4	21.7097%	24	-0.2632	-1.3890	1.1480	0.7945
	Predator diversity	148	14	16.9560%	146	2.2373	0.0359	0.6745	0.0268
	Parasitoid abundance	454	28	27.8265%	452	1.8944	-0.0096	0.4520	0.0588
	Parasitoid diversity	74	4	34.8035%	72	3.2684	0.3086	1.2690	0.0016
	Parasitism	284	7	127.1565%	282	8.0379	0.7216	1.1930	2.4760×10^{-14}
Observational studies	Total relative response of plant performance	254	51	-2.9197%	252	-0.3129	-0.3955	0.2767	0.7546
	Total relative response of invertebrate herbivore performance	298	51	-17.0168%	296	-0.8572	-0.4614	0.1780	0.3920
	Total relative response of natural enemy performance	336	51	8.9548%	334	0.2394	-0.2160	0.2675	0.8109
	Total relative response of predator performance	214	44	9.3474%	212	0.8320	-0.1759	0.4166	0.4063
	Total relative response of parasitoid performance	122	16	14.826%	120	-0.2621	-0.4793	0.3658	0.7937
	Plant growth response	161	24	-5.6244%	159	-0.6779	-0.5926	0.2880	0.4988
	Plant reproduction response	93	27	8.9132%	91	1.5028	-0.1318	0.9197	0.1363
	Plant quality response	0	0	NA	NA	NA	NA	NA	NA
	Herbivore abundance	205	42	-17.138%	203	-1.2397	-0.6539	0.1317	0.2165
	Herbivore damage	39	11	-25.1202%	37	-0.3698	-1.0370	0.7088	0.7135
	Herbivore diversity	54	4	2.3895%	52	0.8265	-0.6077	1.3730	0.4122
	Predator abundance	148	33	15.0365%	146	0.0088	-0.3477	0.3490	0.9930
	Predation	23	10	-21.338%	21	-0.1935	-1.6780	1.3350	0.8483
	Predator diversity	43	3	29.4858%	41	1.1685	-0.3815	1.4580	0.2490
	Parasitoid abundance	56	14	31.5187%	54	0.6877	-0.3893	0.7963	0.4945
	Parasitoid diversity	11	1	24.2857%	9	0.2910	-1.7760	2.5420	0.7764
	Parasitism	55	6	0.5743%	53	-0.7127	-1.0310	0.4672	0.4790

Table S5. Statistic values for the relationship between number of added species richness in the plant species richness treatment over the control and the relative effect sizes of plants, invertebrate herbivores, invertebrate natural enemies, invertebrate predators or invertebrate parasitois along with fitted regression lines. The table gives the predictor variables included, the number of observations and studies, the degree of freedom (d.f.), the corresponding beta regression equation, the 95% confidence interval, standard error (Std.Error), test-statistics (t-value), the corresponding P-value and pseudo R^2. The pseudo R^2 measures the proportion of variance of response explained by the corresponding predictor in each beta regression model. In the beta regression equation, $g^{-1}(u)=e^u/(1+e^u)$ is the inverse of logit function, among that u represents the linear equation measures the association of plant diversity with performance. Note that a value of zero on the X axis indicates that only one plant species richness was added (log scale). Each test is two-sided and the original P value is reported with no multiple comparisons.

Estimate of trophic response category (Y)	Number of added species richness over the control (X)	Number of observations	Number of studies	d.f.	Regression equation	95%CL	95%CU	Std.Error	t-value	P-value	R^2
Relative plant performance	Plant species richness in temperate regions	1759	124	1757	$Y = g^{-1}(0.102X +1.229)$	0.0465	0.1573	0.0283	3.6040	0.0003	0.0142
Relative herbivore performance	Plant species richness in temperate regions	1979	124	1977	$Y = g^{-1}(-0.003X +1.21)$	-0.0587	0.0533	0.0286	-0.0948	0.9245	0.0039
Relative natural enemy performance	Plant species richness in temperate regions	2079	124	2077	$Y = g^{-1}(0.176X +0.586)$	0.1331	0.2185	0.0218	8.0700	7.0230×10^{-16}	0.0480
Relative predator performance	Plant species richness in temperate regions	1274	115	1272	$Y = g^{-1}(0.125X +0.735)$	0.0699	0.1799	0.0281	4.4510	8.5630×10^{-6}	0.0222
Relative parasitoid performance	Plant species richness in temperate regions	805	38	803	$Y = g^{-1}(0.27X +0.322)$	0.2014	0.3382	0.0349	7.7310	1.0650×10^{-14}	0.1109
Relative plant performance	Plant species richness in tropical regions	268	25	266	$Y = g^{-1}(0.097X +1.868)$	-0.0716	0.2647	0.0858	1.1250	0.2605	0.0237
Relative herbivore performance	Plant species richness in tropical regions	235	25	233	$Y = g^{-1}(-0.202X +1.563)$	-0.3479	-0.0568	0.0743	-2.7240	0.0064	0.2325
Relative natural enemy performance	Plant species richness in tropical regions	284	25	282	$Y = g^{-1}(0.039X +1.335)$	-0.0774	0.1550	0.0593	0.6543	0.5129	0.0002
Relative predator performance	Plant species richness in tropical regions	155	20	153	$Y = g^{-1}(0.301X +1.068)$	0.0329	0.5701	0.1370	2.2000	0.0278	0.0715
Relative parasitoid performance	Plant species richness in tropical regions	129	11	127	$Y = g^{-1}(-0.069X +1.548)$	-0.2130	0.0755	0.0736	-0.9342	0.3502	0.0311
Relative plant performance	Plant species richness in herbaceous plant	1304	98	1302	$Y = g^{-1}(0.187X +1.433)$	0.1136	0.2605	0.0375	4.9890	6.0710×10^{-7}	0.0225
Relative herbivore performance	Plant species richness in herbaceous plant	1679	98	1677	$Y = g^{-1}(0.044X +1.138)$	-0.0275	0.1154	0.0365	1.2050	0.2282	0.0044
Relative natural enemy performance	Plant species richness in herbaceous plant	1668	98	1666	$Y = g^{-1}(0.156X +0.598)$	0.1070	0.2045	0.0249	6.2660	3.7130×10^{-10}	0.0378
Relative predator performance	Plant species richness in herbaceous plant	984	87	982	$Y = g^{-1}(0.098X +0.725)$	0.0350	0.1618	0.0324	3.0410	0.0024	0.0133
Relative parasitoid performance	Plant species richness in herbaceous plant	684	29	682	$Y = g^{-1}(0.26X +0.398)$	0.1822	0.3377	0.0397	6.5550	5.5790×10^{-11}	0.0955
Relative plant performance	Plant species richness in woody plant	723	51	721	$Y = g^{-1}(0.027X +1.098)$	-0.0502	0.1045	0.0395	0.6874	0.4918	0.0036
Relative herbivore performance	Plant species richness in woody plant	535	51	533	$Y = g^{-1}(-0.162X +1.606)$	-0.2458	-0.0786	0.0427	-3.8020	0.0001	0.0597
Relative natural enemy performance	Plant species richness in woody plant	695	51	693	$Y = g^{-1}(0.17X +0.971)$	0.0992	0.2416	0.0363	4.6930	2.6960×10^{-6}	0.0343
Relative predator performance	Plant species richness in woody plant	445	48	443	$Y = g^{-1}(0.243X +1.033)$	0.1415	0.3453	0.0520	4.6830	2.8340×10^{-6}	0.0519
Relative parasitoid performance	Plant species richness in woody plant	250	20	248	$Y = g^{-1}(0.154X +0.723)$	0.0501	0.2577	0.0529	2.9070	0.0036	0.0294
Relative plant performance	Plant species richness in managed study	1773	98	1771	$Y = g^{-1}(0.166X +1.253)$	0.0976	0.2337	0.0347	4.7710	1.8370×10^{-6}	0.0363
Relative herbivore performance	Plant species richness in managed study	1916	98	1914	$Y = g^{-1}(-0.037X +1.269)$	-0.1061	0.0322	0.0353	-1.0460	0.2954	0.0078
Relative natural enemy performance	Plant species richness in managed study	2027	98	2025	$Y = g^{-1}(0.162X +0.653)$	0.1157	0.2092	0.0239	6.8070	9.9550×10^{-12}	0.0573
Relative predator performance	Plant species richness in managed study	1215	91	1213	$Y = g^{-1}(0.105X +0.789)$	0.0454	0.1644	0.0304	3.4540	0.0006	0.0337
Relative parasitoid performance	Plant species richness in managed study	812	33	810	$Y = g^{-1}(0.267X +0.431)$	0.1911	0.3438	0.0390	6.8660	6.6070×10^{-12}	0.1068
Relative plant performance	Plant species richness in observational study	254	51	252	$Y = g^{-1}(-0.185X +1.956)$	-0.3306	-0.0385	0.0745	-2.4770	0.0133	0.0592

Relative herbivore performance	Plant species richness in observational study	298	51	296	$Y = g^{-1}(-0.042X +1.282)$	-0.1774	0.0940	0.0692	-0.6025	0.5469	0.0604
Relative natural enemy performance	Plant species richness in observational study	336	51	334	$Y = g^{-1}(0.095X +0.911)$	-0.0199	0.2106	0.0588	1.6210	0.1049	0.0030
Relative predator performance	Plant species richness in observational study	214	44	212	$Y = g^{-1}(0.17X +0.821)$	0.0201	0.3203	0.0766	2.2220	0.0263	0.0034
Relative parasitoid performance	Plant species richness in observational study	122	16	120	$Y = g^{-1}(0.036X +0.967)$	-0.1632	0.2354	0.1017	0.3554	0.7223	0.0034
Relative plant performance	Plant species richness in across all studies	2027	149	2025	$Y = g^{-1}(0.096X +1.307)$	0.0433	0.1484	0.0268	3.5740	0.0004	0.0140
Relative herbivore performance	Plant species richness in across all studies	2214	149	2212	$Y = g^{-1}(-0.03X +1.261)$	-0.0833	0.0237	0.0273	-1.0900	0.2755	0.0121
Relative natural enemy performance	Plant species richness in across all studies	2363	149	2361	$Y = g^{-1}(0.162X +0.667)$	0.1217	0.2021	0.0205	7.8980	2.8240×10^{-15}	0.0363
Relative predator performance	Plant species richness in across all studies	1429	135	1427	$Y = g^{-1}(0.119X +0.79)$	0.0662	0.1726	0.0272	4.3960	1.1050×10^{-5}	0.0211
Relative parasitoid performance	Plant species richness in across all studies	934	49	932	$Y = g^{-1}(0.234X +0.453)$	0.1726	0.2945	0.0311	7.5090	5.9790×10^{-14}	0.0711
Relative plant performance	Plant species richness in organic croplands	760	55	758	$Y = g^{-1}(0.452X +1.257)$	0.2876	0.6161	0.0838	5.3930	6.93×10^{-8}	0.1480
Relative herbivore performance	Plant species richness in organic croplands	926	55	924	$Y = g^{-1}(-0.624X +1.71)$	-0.7666	-0.4819	0.0726	-8.5950	8.34×10^{-18}	0.0842
Relative natural enemy performance	Plant species richness in organic croplands	775	55	773	$Y = g^{-1}(0.564X +0.657)$	0.4243	0.7041	0.0714	7.9040	2.70×10^{-15}	0.1662
Relative predator performance	Plant species richness in organic croplands	376	47	374	$Y = g^{-1}(0.423X +1.209)$	0.2165	0.6285	0.1051	4.0200	5.83×10^{-5}	0.1490
Relative parasitoid performance	Plant species richness in organic croplands	399	23	397	$Y = g^{-1}(0.713X +0.175)$	0.5201	0.9061	0.0985	7.2420	4.41×10^{-13}	0.1820
Relative plant performance	Plant species richness in non-organic croplands	571	49	569	$Y = g^{-1}(0.202X +1.452)$	0.0180	0.3864	0.0940	2.1520	0.0314	0.0410
Relative herbivore performance	Plant species richness in non-organic croplands	419	49	417	$Y = g^{-1}(-0.497X +1.864)$	-0.6947	-0.2991	0.1009	-4.9250	8.46×10^{-7}	0.1762
Relative natural enemy performance	Plant species richness in non-organic croplands	533	49	531	$Y = g^{-1}(0.487X +0.563)$	0.3210	0.6522	0.0845	5.7600	8.39×10^{-9}	0.1919
Relative predator performance	Plant species richness in non-organic croplands	373	48	371	$Y = g^{-1}(0.467X +0.825)$	0.2569	0.6780	0.1074	4.3510	1.35×10^{-5}	0.1698
Relative parasitoid performance	Plant species richness in non-organic croplands	160	11	158	$Y = g^{-1}(0.545X +0.117)$	0.2658	0.8244	0.1425	3.8250	0.0001	0.2465
Relative plant performance	Plant species richness in grasslands	454	26	452	$Y = g^{-1}(0.249X +0.993)$	0.1543	0.3440	0.0484	5.1470	2.6410×10^{-7}	0.0500
Relative herbivore performance	Plant species richness in grasslands	603	26	601	$Y = g^{-1}(0.312X +0.415)$	0.2356	0.3889	0.0391	7.9870	1.3860×10^{-15}	0.1149
Relative natural enemy performance	Plant species richness in grasslands	752	26	750	$Y = g^{-1}(0.219X +0.196)$	0.1568	0.2818	0.0319	6.8780	6.0750×10^{-12}	0.0572
Relative predator performance	Plant species richness in grasslands	498	24	496	$Y = g^{-1}(0.239X +0.054)$	0.1615	0.3158	0.0394	6.0640	1.3240×10^{-9}	0.0462
Relative parasitoid performance	Plant species richness in grasslands	254	10	252	$Y = g^{-1}(0.11X +0.796)$	0.0001	0.2201	0.0561	1.9620	0.0497	0.0904
Relative plant performance	Plant species richness in forests	242	19	240	$Y = g^{-1}(0.039X +0.655)$	-0.0655	0.1432	0.0532	0.7296	0.4656	0.0000
Relative herbivore performance	Plant species richness in forests	266	19	264	$Y = g^{-1}(-0.009X +1.163)$	-0.1169	0.0989	0.0551	-0.1641	0.8697	0.0002
Relative natural enemy performance	Plant species richness in forests	303	19	301	$Y = g^{-1}(0.009X +1.264)$	-0.0920	0.1101	0.0515	0.1754	0.8607	0.0000
Relative predator performance	Plant species richness in forests	182	16	180	$Y = g^{-1}(0.054X +1.499)$	-0.0835	0.1906	0.0699	0.7652	0.4441	0.0009
Relative parasitoid performance	Plant species richness in forests	121	5	119	$Y = g^{-1}(0.137X +0.402)$	-0.0131	0.2864	0.0764	1.7890	0.0737	0.0171

Table S6. Results of the path analyses for the bottom-up and top-down effects of binary plant species richness on the tri-trophic interactions of plant performance (i.e., plant growth, plant reproduction and plant quality), invertebrate herbivore performance (i.e., herbivore abundance, herbivore damage and herbivore diverisity) and their invertebrate natural enemy performance (i.e., predator abundance, predation, predator diversity, parasitoid abundance, parasitism and parasitoid diversity) in global terrestrial ecosystems, organic and non-organic croplands, grasslands and forests (as presented also in Fig. 4) as well as in Creek grassland biodiversity experiments in USA, Jena grassland biodiversity experiments in Germany, and in BEF forest biodiversity experiments in China. The predictor and response columns specify the trophic group pairs and the moderator category. The estimate represents the strength of the relationship. The std. err. of estimate denotes the standardized error of the estimate coefficients for the fitted path-analytic models. The number of studies and observations for the predictor-response pair are also presented. Each test is two-sided and the original P value is reported with no multiple comparisons. In addition, test statistic (t value), and 95% confidence interval are reported. R^2 represents the proportion of variance explained for each endogenous variable, the reported R^2 is marginal, which represents variance explained by fixed effects only. Fisher's C statistic assesses the goodness-of-fit of the model through Shipley's test of directed separation. AICc is the adjusted Akaike´s information criterion, the strength of top-down and bottom-up pathways can be assessed using the differences in the AICc values.

Ecosystem type	Tri-trophic interaction (effect classification)	Predictor	Response	Number of observations	Number of studies	Estimate	Std.Err. of Estimate	t-value	P-value	CI_{lb}	CI_{ub}	R^2	Fisher´s C	AICc
Global terrestrial ecosystems	Binary plant species richness on tri-trophic interactions of plants, herbivores and natural enemies (bottom-up effects)	Plant species richness	→Plant performance	3241	149	0.3866	0.0314	12.3121	5.3652×10^{-34}	0.3250	0.4482	0.04	22.499	24683.145
		Plant performance	→Herbivore performance	3241	149	-0.0167	0.0165	-1.0121	0.3116	-0.0491	0.0157	0.09	22.499	24683.145
		Herbivore performance	→Natural enemy performance	3241	149	-0.0108	0.0173	-17.4809	1.7179×10^{-59}	-0.6105	-0.4873	0.13	22.499	24683.145
		Plant species richness	→Herbivore performance	3241	149	-0.5489	0.0314	-0.6243	0.5325	-0.0447	0.0231	0.09	22.499	24683.145
		Plant species richness	→Natural enemy performance	3241	149	0.6892	0.0325	21.2062	1.0588×10^{-81}	0.6254	0.7530	0.13	22.499	24683.145
	Binary plant species richness on tri-trophic interactions of plants, herbivores and natural enemies (top-down effects)	Plant species richness	→Natural enemy performance	3241	149	0.6986	0.0309	22.6084	5.64169×10^{-6}	0.6380	0.7592	0.13	23.911	24670.829
		Natural enemy performance	→Herbivore performance	3241	149	-0.0183	0.0175	-1.0457	0.2958	-0.0526	0.0160	0.09	23.911	24670.829
		Herbivore performance	→Plant performance	3241	149	-0.0200	0.0171	-16.4169	1.6377×10^{-53}	-0.6084	-0.4784	0.04	23.911	24670.829
		Plant species richness	→Herbivore performance	3241	149	-0.5434	0.0331	-1.1696	0.2423	-0.0535	0.0135	0.09	23.911	24670.829
		Plant species richness	→Plant performance	3241	149	0.3757	0.0332	11.3163	4.6835×10^{-29}	0.3106	0.4408	0.04	23.911	24670.829
Organic croplands	Binary plant species richness on tri-trophic interactions of plants, herbivores and natural enemies (bottom-up effects)	Plant species richness	→Plant performance	1189	55	0.3770	0.0433	8.7067	1.08331×10^{-17}	0.2920	0.4620	0.03	22.499	8409.178
		Plant performance	→Herbivore performance	1189	55	-0.1176	0.0243	-4.8395	1.4790×10^{-6}	-0.1653	-0.0699	0.24	22.499	8409.178
		Herbivore performance	→Natural enemy performance	1189	55	-0.0818	0.0295	-19.3296	2.6217×10^{-72}	-0.9560	-0.7798	0.21	22.499	8409.178
		Plant species richness	→Herbivore performance	1189	55	-0.8679	0.0449	-2.7729	5.6453×10^{-3}	-0.1397	-0.0239	0.24	22.499	8409.178
		Plant species richness	→Natural enemy performance	1189	55	0.7738	0.0545	14.1982	2.8338×10^{-42}	0.6669	0.8807	0.21	22.499	8409.178
	Binary plant species richness on tri-trophic interactions of plants, herbivores and natural enemies (top-down effects)	Plant species richness	→Natural enemy performance	1189	55	0.8525	0.0471	18.0998	1.1926×10^{-64}	0.7601	0.9449	0.21	23.911	8395.72
		Natural enemy performance	→Herbivore performance	1189	55	-0.0822	0.0271	-3.0332	2.4739×10^{-3}	-0.1354	-0.0290	0.23	23.911	8395.72
		Herbivore performance	→Plant performance	1189	55	-0.1468	0.0269	-16.8120	7.5216×10^{-57}	-0.9387	-0.7425	0.05	23.911	8395.72
		Plant species richness	→Herbivore performance	1189	55	-0.8406	0.0500	-5.4572	5.9237×10^{-8}	-0.1996	-0.0940	0.23	23.911	8395.72
		Plant species richness	→Plant performance	1189	55	0.2423	0.0496	4.8851	1.1845×10^{-6}	0.1450	0.3396	0.05	23.911	8395.72
Non-organic	Binary plant species richness on tri-trophic interactions of plants, herbivores and natural enemies (bottom-up effects)	Plant species richness	→Plant performance	887	49	0.1804	0.0550	3.2800	1.0798×10^{-3}	0.0725	0.2883	0.01	22.499	6242.761
		Plant performance	→Herbivore performance	887	49	-0.0797	0.0269	-2.9628	3.1314×10^{-3}	-0.1325	-0.0269	0.36	22.499	6242.761
		Herbivore performance	→Natural enemy performance	887	49	-0.0979	0.0366	-22.6605	2.1027×10^{-89}	-1.1893	-0.9997	0.23	22.499	6242.761
		Plant species richness	→Herbivore performance	887	49	-1.0945	0.0483	-2.6749	7.6167×10^{-3}	-0.1697	-0.0261	0.36	22.499	6242.761
		Plant species richness	→Natural enemy performance	887	49	0.7897	0.0664	11.8931	2.6121×10^{-30}	0.6594	0.9200	0.23	22.499	6242.761

croplands	Binary plant species richness on tri-trophic interactions of plants, herbivores and natural enemies (top-down effects)	Plant species richness	→Natural enemy performance	887	49	0.9051	0.0523	17.3059	8.7805×10^{-58}	0.8024	1.0078	0.23	23.911	6219.8
		Natural enemy performance	→Herbivore performance	887	49	-0.0974	0.0298	-3.2685	1.1246×10^{-3}	-0.1559	-0.0389	0.36	23.911	6219.8
		Herbivore performance	→Plant performance	887	49	-0.1451	0.0382	-18.6673	1.81526×10^{-5}	-1.1347	-0.9187	0.02	23.911	6219.8
		Plant species richness	→Herbivore performance	887	49	-1.0267	0.0550	-3.7984	1.5601×10^{-4}	-0.2201	-0.0701	0.36	23.911	6219.8
		Plant species richness	→Plant performance	887	49	0.0201	0.0693	0.2900	0.7719	-0.1159	0.1561	0.02	23.911	6219.8
Grasslands	Binary plant species richness on tri-trophic interactions of plants, herbivores and natural enemies (bottom-up effects)	Plant species richness	→Plant performance	832	26	0.5415	0.0719	7.5313	1.3819×10^{-13}	0.4004	0.6826	0.06	22.538	6455.606
		Plant performance	→Herbivore performance	832	26	0.0590	0.0303	1.9472	0.0519	-0.0005	0.1185	0.02	22.538	6455.606
		Herbivore performance	→Natural enemy performance	832	26	0.2742	0.0346	3.4325	0.0006	0.0958	0.3518	0.09	22.538	6455.606
		Plant species richness	→Herbivore performance	832	26	0.2238	0.0652	7.9249	5.7810×10^{-14}	0.2061	0.3423	0.02	22.538	6455.606
		Plant species richness	→Natural enemy performance	832	26	0.1707	0.0709	2.4076	0.0163	0.0315	0.3099	0.09	22.538	6455.606
	Binary plant species richness on tri-trophic interactions of plants, herbivores and natural enemies (top-down effects)	Plant species richness	→Natural enemy performance	832	26	0.2393	0.0722	3.3144	0.0010	0.0976	0.3810	0.01	23.949	6455.793
		Natural enemy performance	→Herbivore performance	832	26	0.2153	0.0295	7.2983	7.4199×10^{-13}	0.1574	0.2732	0.06	23.949	6455.793
		Herbivore performance	→Plant performance	832	26	0.0530	0.0338	3.3177	0.0010	0.0836	0.3258	0.07	23.949	6455.793
		Plant species richness	→Herbivore performance	832	26	0.2047	0.0617	1.5680	0.1173	-0.0133	0.1193	0.06	23.949	6455.793
		Plant species richness	→Plant performance	832	26	0.5265	0.0725	7.2621	9.2130×10^{-13}	0.3842	0.6688	0.07	23.949	6455.793
Forests	Binary plant species richness on tri-trophic interactions of plants, herbivores and natural enemies (bottom-up effects)	Plant species richness	→Plant performance	333	19	0.9642	0.1169	8.2481	4.5500×10^{-15}	0.7342	1.1942	0.17	22.538	2613.338
		Plant performance	→Herbivore performance	333	19	0.1052	0.0539	1.9518	0.0518	-0.0008	0.2112	0.02	22.538	2613.338
		Herbivore performance	→Natural enemy performance	333	19	0.1292	0.0547	1.3935	0.1644	-0.0741	0.4339	0.06	22.538	2613.338
		Plant species richness	→Herbivore performance	333	19	0.1799	0.1291	2.3620	0.0188	0.0215	0.2369	0.02	22.538	2613.338
		Plant species richness	→Natural enemy performance	333	19	0.4415	0.1203	3.6700	0.0003	0.2048	0.6782	0.06	22.538	2613.338
	Binary plant species richness on tri-trophic interactions of plants, herbivores and natural enemies (top-down effects)	Plant species richness	→Natural enemy performance	333	19	0.4855	0.1197	4.0560	6.2572×10^{-5}	0.2500	0.7210	0.04	23.949	2614.129
		Natural enemy performance	→Herbivore performance	333	19	0.1277	0.0518	2.4653	0.0142	0.0258	0.2296	0.03	23.949	2614.129
		Herbivore performance	→Plant performance	333	19	0.0839	0.0504	1.8884	0.0599	-0.0094	0.4596	0.18	23.949	2614.129
		Plant species richness	→Herbivore performance	333	19	0.2251	0.1192	1.6647	0.0970	-0.0153	0.1831	0.03	23.949	2614.129
		Plant species richness	→Plant performance	333	19	0.9383	0.1176	7.9787	2.88181×10^{-4}	0.7069	1.1697	0.18	23.949	2614.129
Creek grassland biodiversity experiments in USA	Binary plant species richness on tri-trophic interactions of plants, herbivores and natural enemies (bottom-up effects)	Plant species richness	→Plant performance	268	6	0.3713	0.1223	3.036	0.0027	0.1304	0.6122	0.03	11.638	2205.329
		Plant performance	→Herbivore performance	268	6	0.0107	0.0623	0.1717	0.8638	-0.112	0.1334	0.01	11.638	2205.329
		Herbivore performance	→Natural enemy performance	268	6	0.0328	0.0614	-1.2658	0.2068	-0.409	0.089	0.01	11.638	2205.329
		Plant species richness	→Herbivore performance	268	6	-0.1600	0.1264	0.5342	0.5937	-0.0881	0.1537	0.01	11.638	2205.329
		Plant species richness	→Natural enemy performance	268	6	-0.1405	0.1246	-1.1276	0.2606	-0.3859	0.1049	0.01	11.638	2205.329
		Plant species richness	→Natural enemy performance	268	6	-0.1456	0.124	-1.1742	0.2415	-0.3898	0.0986	0.01	11.664	2205.387

	Binary plant species richness on tri-trophic interactions of plants, herbivores and natural enemies (top-down effects)	Natural enemy performance	→Herbivore performance	268	6	0.0328	0.0614	0.5342	0.5937	-0.0881	0.1537	0.01	11.664	2205.387
		Herbivore performance	→Plant performance	268	6	0.0104	0.0606	-1.2145	0.2257	-0.3964	0.094	0.03	11.664	2205.387
		Plant species richness	→Herbivore performance	268	6	-0.1512	0.1245	0.1716	0.8639	-0.1089	0.1297	0.01	11.664	2205.387
		Plant species richness	→Plant performance	268	6	0.3729	0.1228	3.0366	0.0027	0.131	0.6148	0.03	11.664	2205.387
Jena grassland biodiversity experiments in Germany	Binary plant species richness on tri-trophic interactions of plants, herbivores and natural enemies (bottom-up effects)	Plant species richness	→Plant performance	146	3	1.2473	0.1678	7.4333	9.7769×10^{-12}	0.9155	1.5791	0.28	13.416	1091.698
		Plant performance	→Herbivore performance	146	3	0.2466	0.0802	3.0748	0.0025	0.088	0.4052	0.08	13.416	1091.698
		Herbivore performance	→Natural enemy performance	146	3	0.5731	0.0684	1.3426	0.1816	-0.1208	0.6318	0.37	13.416	1091.698
		Plant species richness	→Herbivore performance	146	3	0.2555	0.1903	8.3787	6.5607×10^{-6}	0.4213	0.7249	0.08	13.416	1091.698
		Plant species richness	→Natural enemy performance	146	3	0.2646	0.1621	1.6323	0.1049	-0.0559	0.5851	0.37	13.416	1091.698
	Binary plant species richness on tri-trophic interactions of plants, herbivores and natural enemies (top-down effects)	Plant species richness	→Natural enemy performance	146	3	0.5915	0.1853	3.1921	0.0017	0.2251	0.9579	0.06	14.254	1099.41
		Natural enemy performance	→Herbivore performance	146	3	0.4792	0.0639	7.4992	1.5948E-11	0.3526	0.6058	0.25	14.254	1099.41
		Herbivore performance	→Plant performance	146	3	0.1815	0.0719	1.9201	0.0569	-0.0084	0.5706	0.31	14.254	1099.41
		Plant species richness	→Herbivore performance	146	3	0.2811	0.1464	2.5243	0.0127	0.0393	0.3237	0.25	14.254	1099.41
		Plant species richness	→Plant performance	146	3	1.1383	0.1704	6.6802	5.3663×10^{-10}	0.8014	1.4752	0.31	14.254	1099.41
BEF forest biodiversity experiments in China	Binary plant species richness on tri-trophic interactions of plants, herbivores and natural enemies (bottom-up effects)	Plant species richness	→Plant performance	156	5	0.9663	0.1864	5.184	6.9334×10^{-7}	0.598	1.3346	0.14	1.677	1171.777
		Plant performance	→Herbivore performance	156	5	-0.0578	0.0604	-0.957	0.3402	-0.1772	0.0616	0.06	1.677	1171.777
		Herbivore performance	→Natural enemy performance	156	5	0.3074	0.0846	4.3351	2.6841×10^{-5}	0.3597	0.9625	0.12	1.677	1171.777
		Plant species richness	→Herbivore performance	156	5	0.6611	0.1525	3.6336	0.0004	0.1401	0.4747	0.06	1.677	1171.777
		Plant species richness	→Natural enemy performance	156	5	0.2558	0.1712	1.4942	0.1373	-0.0825	0.5941	0.12	1.677	1171.777
	Binary plant species richness on tri-trophic interactions of plants, herbivores and natural enemies (top-down effects)	Plant species richness	→Natural enemy performance	156	5	0.4464	0.1693	2.6367	0.0093	0.1119	0.7809	0.03	2.119	1175.936
		Natural enemy performance	→Herbivore performance	156	5	0.1989	0.0653	3.0459	0.0027	0.0699	0.3279	0.1	2.119	1175.936
		Herbivore performance	→Plant performance	156	5	-0.0529	0.0762	3.704	0.0003	0.2422	0.7964	0.14	2.119	1175.936
		Plant species richness	→Herbivore performance	156	5	0.5193	0.1402	-0.6942	0.4886	-0.2035	0.0977	0.1	2.119	1175.936
		Plant species richness	→Plant performance	156	5	0.9982	0.1923	5.1908	6.7667×10^{-7}	0.6182	1.3782	0.14	2.119	1175.936

Table S7. Results of the path analyses for the bottom-up and top-down effects of binary plant species richness on the tri-trophic interactions of plant performance (i.e., plant growth, plant reproduction and plant quality), invertebrate herbivore performance (i.e., herbivore abundance, herbivore damage and herbivore diveristy) and their invertebrate natural enemy performance (i.e., predator abundance, predation, predator diversity, parasitoid abundance, parasitism and parasitoid diversity) in croplands across different climatic regions, different plant types, and different study types (as presented also in figs. S10−12). The predictor and response columns specify the trophic group pairs and the moderator category. The estimate represents the strength of the relationship. The std. err. of estimate denotes the standardized error of the estimate coefficients for the fitted path-analytic models. The number of studies and observations for the predictor-response pair are also presented. Each test is two-sided and the original P value is reported with no multiple comparisons. In addition, test statistic (t value), and 95% confidence interval are reported. R^2 represents the proportion of variance explained for each endogenous variable, the reported R^2 is marginal, which represents variance explained by fixed effects only. Fisher's C statistic assesses the goodness-of-fit of the model through Shipley's test of directed separation. AICc is the adjusted Akaike´s information criterion, the strength of top-down and bottom-up pathways can be assessed using the differences in the AICc values.

Ecosystem type (other item)	Tri-trophic interaction (effect classification)	Predictor	Response	Number of observations	Number of studies	Estimate	Std.Err. of Estimate	t-value	P-value	CI_{lb}	CI_{ub}	R^2	Fisher´s C	AICc
Croplands (temperate regions)	Binary plant species richness on tri-trophic interactions of plants, herbivores and natural enemies (bottom-up effects)	Plant species richness	→Plant performance	1498	82	0.2639	0.0437	6.0389	1.9671×10^{-9}	0.1782	0.3496	0.02	9.042	10721.293
		Plant performance	→Herbivore performance	1498	82	-0.1352	0.0214	-6.3178	3.5066×10^{-10}	-0.1772	-0.0932	0.33	9.042	10721.293
		Herbivore performance	→Natural enemy performance	1498	82	-0.1163	0.0273	-25.7617	7.0345×10^{-121}	-1.0701	-0.9187	0.24	9.042	10721.293
		Plant species richness	→Herbivore performance	1498	82	-0.9944	0.0386	-4.2601	2.1737×10^{-5}	-0.1699	-0.0627	0.33	9.042	10721.293
		Plant species richness	→Natural enemy performance	1498	82	0.7712	0.0496	15.5484	1.4586×10^{-50}	0.6739	0.8685	0.24	9.042	10721.293
	Binary plant species richness on tri-trophic interactions of plants, herbivores and natural enemies (top-down effects)	Plant species richness	→Natural enemy performance	1498	82	0.8957	0.0409	21.8998	4.0151×10^{-92}	0.8155	0.9759	0.23	9.962	10687.331
		Natural enemy performance	→Herbivore performance	1498	82	-0.1076	0.0240	-4.4833	7.9254×10^{-6}	-0.1547	-0.0605	0.32	9.962	10687.331
		Herbivore performance	→Plant performance	1498	82	-0.2137	0.0286	-21.3417	4.1596×10^{-88}	-1.0230	-0.8508	0.05	9.962	10687.331
		Plant species richness	→Herbivore performance	1498	82	-0.9369	0.0439	-7.4720	1.3906×10^{-13}	-0.2698	-0.1576	0.32	9.962	10687.331
		Plant species richness	→Plant performance	1498	82	0.0434	0.0521	0.8330	0.4050	-0.0588	0.1456	0.05	9.962	10687.331
Croplands (tropical regions)	Binary plant species richness on tri-trophic interactions of plants, herbivores and natural enemies (bottom-up effects)	Plant species richness	→Plant performance	578	22	0.3782	0.0549	6.8889	1.5587×10^{-11}	0.2704	0.4860	0.04	9.042	3943.857
		Plant performance	→Herbivore performance	578	22	-0.0155	0.0318	-0.4874	0.6262	-0.0780	0.0470	0.25	9.042	3943.857
		Herbivore performance	→Natural enemy performance	578	22	-0.0475	0.0428	-14.9934	7.5591×10^{-43}	-1.0225	-0.7857	0.18	9.042	3943.857
		Plant species richness	→Herbivore performance	578	22	-0.9041	0.0603	-1.1098	0.2676	-0.1316	0.0366	0.25	9.042	3943.857
		Plant species richness	→Natural enemy performance	578	22	0.7852	0.0805	9.7540	7.2330×10^{-21}	0.6271	0.9433	0.18	9.042	3943.857
	Binary plant species richness on tri-trophic interactions of plants, herbivores and natural enemies (top-down effects)	Plant species richness	→Natural enemy performance	578	22	0.8333	0.0688	12.1119	3.7184×10^{-30}	0.6982	0.9684	0.19	9.962	3939.963
		Natural enemy performance	→Herbivore performance	578	22	-0.0600	0.0350	-1.7143	0.0870	-0.1287	0.0087	0.22	9.962	3939.963
		Herbivore performance	→Plant performance	578	22	-0.0066	0.0338	-13.0715	3.7619×10^{-34}	-0.9879	-0.7297	0.04	9.962	3939.963
		Plant species richness	→Herbivore performance	578	22	-0.8588	0.0657	-0.1953	0.8453	-0.0730	0.0598	0.22	9.962	3939.963
		Plant species richness	→Plant performance	578	22	0.3782	0.0636	5.9465	5.0136×10^{-9}	0.2533	0.5031	0.04	9.962	3939.963
Croplands (herbaceous plants)	Binary plant species richness on tri-trophic interactions of plants, herbivores and natural enemies (bottom-up effects)	Plant species richness	→Plant performance	1350	72	0.3345	0.0433	7.7252	2.2138×10^{-14}	0.2496	0.4194	0.03	9.042	9762.58
		Plant performance	→Herbivore performance	1350	72	-0.1296	0.0241	-5.3776	8.9062×10^{-8}	-0.1769	-0.0823	0.22	9.042	9762.58
		Herbivore performance	→Natural enemy performance	1350	72	-0.1121	0.0277	-18.2500	1.7548×10^{-66}	-0.8731	-0.7037	0.17	9.042	9762.58
		Plant species richness	→Herbivore performance	1350	72	-0.7884	0.0432	-4.0469	5.4896×10^{-5}	-0.1664	-0.0578	0.22	9.042	9762.58
		Plant species richness	→Natural enemy performance	1350	72	0.6540	0.0499	13.1062	5.6235×10^{-37}	0.5561	0.7519	0.17	9.042	9762.58
	Binary plant species richness	Plant species richness	→Natural enemy performance	1350	72	0.7508	0.0443	16.9481	2.0213×10^{-58}	0.6639	0.8377	0.17	9.962	9755.249
		Natural enemy performance	→Herbivore performance	1350	72	-0.1002	0.0258	-3.8837	0.0001	-0.1508	-0.0496	0.21	9.962	9755.249

	on tri-trophic interactions of plants, herbivores and natural enemies (top-down effects)	Herbivore performance	→Plant performance	1350	72	-0.1578	0.0268	-16.0533	4.5281×10^{-53}	-0.8449	-0.6609	0.05	9.962	9755.249
		Plant species richness	→Herbivore performance	1350	72	-0.7529	0.0469	-5.8881	4.9592×10^{-9}	-0.2104	-0.1052	0.21	9.962	9755.249
		Plant species richness	→Plant performance	1350	72	0.2021	0.0485	4.1670	3.2908×10^{-5}	0.1070	0.2972	0.05	9.962	9755.249
Croplands (woody plants)	Binary plant species richness on tri-trophic interactions of plants, herbivores and natural enemies (bottom-up effects)	Plant species richness	→Plant performance	726	32	0.2195	0.0608	3.6102	0.0003	0.1001	0.3389	0.01	9.042	4978.956
		Plant performance	→Herbivore performance	726	32	-0.0491	0.0257	-1.9105	0.0565	-0.0996	0.0014	0.47	9.042	4978.956
		Herbivore performance	→Natural enemy performance	726	32	-0.0284	0.0425	-26.7766	2.4324×10^{-109}	-1.4025	-1.2109	0.32	9.042	4978.956
		Plant species richness	→Herbivore performance	726	32	-1.3067	0.0488	-0.6682	0.5042	-0.1118	0.0550	0.47	9.042	4978.956
		Plant species richness	→Natural enemy performance	726	32	1.0614	0.0805	13.1851	1.2423×10^{-35}	0.9034	1.2194	0.32	9.042	4978.956
	Binary plant species richness on tri-trophic interactions of plants, herbivores and natural enemies (top-down effects)	Plant species richness	→Natural enemy performance	726	32	1.1051	0.0571	19.3538	2.9738×10^{-67}	0.9930	1.2172	0.32	9.962	4963.036
		Natural enemy performance	→Herbivore performance	726	32	-0.0207	0.0315	-0.6571	0.5113	-0.0825	0.0411	0.47	9.962	4963.036
		Herbivore performance	→Plant performance	726	32	-0.0941	0.0450	-21.8507	4.9988×10^{-81}	-1.4193	-1.1853	0.02	9.962	4963.036
		Plant species richness	→Herbivore performance	726	32	-1.3023	0.0596	-2.0911	0.0369	-0.1825	-0.0057	0.47	9.962	4963.036
		Plant species richness	→Plant performance	726	32	0.0980	0.0850	1.1529	0.2493	-0.0689	0.2649	0.02	9.962	4963.036
Croplands (managed experiment)	Binary plant species richness on tri-trophic interactions of plants, herbivores and natural enemies (bottom-up effects)	Plant species richness	→Plant performance	1923	74	0.2729	0.0373	7.3164	3.7576×10^{-13}	0.1997	0.3461	0.02	9.042	13653.548
		Plant performance	→Herbivore performance	1923	74	-0.0862	0.0185	-4.6595	3.3910×10^{-6}	-0.1225	-0.0499	0.33	9.042	13653.548
		Herbivore performance	→Natural enemy performance	1923	74	-0.0899	0.0242	-30.7640	1.2235×10^{-168}	-1.1094	-0.9764	0.25	9.042	13653.548
		Plant species richness	→Herbivore performance	1923	74	-1.0429	0.0339	-3.7149	0.0002	-0.1374	-0.0424	0.33	9.042	13653.548
		Plant species richness	→Natural enemy performance	1923	74	0.8238	0.0446	18.4709	3.7364×10^{-70}	0.7363	0.9113	0.25	9.042	13653.548
	Binary plant species richness on tri-trophic interactions of plants, herbivores and natural enemies (top-down effects)	Plant species richness	→Natural enemy performance	1923	74	0.9231	0.0364	25.3599	2.6512×10^{-122}	0.8517	0.9945	0.24	9.962	13627.091
		Natural enemy performance	→Herbivore performance	1923	74	-0.0755	0.0209	-3.6124	0.0003	-0.1165	-0.0345	0.32	9.962	13627.091
		Herbivore performance	→Plant performance	1923	74	-0.1489	0.0245	-25.8269	4.3472×10^{-126}	-1.0754	-0.9236	0.03	9.962	13627.091
		Plant species richness	→Herbivore performance	1923	74	-0.9995	0.0387	-6.0776	1.4736×10^{-9}	-0.1970	-0.1008	0.32	9.962	13627.091
		Plant species richness	→Plant performance	1923	74	0.1141	0.0453	2.5188	0.0119	0.0253	0.2029	0.03	9.962	13627.091
Croplands (observational experiment)	Binary plant species richness on tri-trophic interactions of plants, herbivores and natural enemies (bottom-up effects)	Plant species richness	→Plant performance	153	30	0.4990	0.1351	3.6936	0.0003	0.2319	0.7661	0.08	9.042	1141.034
		Plant performance	→Herbivore performance	153	30	-0.2253	0.0744	-3.0282	0.0030	-0.3725	-0.0781	0.06	9.042	1141.034
		Herbivore performance	→Natural enemy performance	153	30	-0.0164	0.0709	-0.5691	0.5702	-0.3334	0.1844	0.03	9.042	1141.034
		Plant species richness	→Herbivore performance	153	30	-0.0745	0.1309	-0.2313	0.8175	-0.1567	0.1239	0.06	9.042	1141.034
		Plant species richness	→Natural enemy performance	153	30	0.2856	0.1248	2.2885	0.0237	0.0387	0.5325	0.03	9.042	1141.034
	Binary plant species richness on tri-trophic interactions of plants, herbivores and natural enemies (top-down effects)	Plant species richness	→Natural enemy performance	153	30	0.3058	0.1220	2.5066	0.0134	0.0645	0.5471	0.03	9.962	1141.272
		Natural enemy performance	→Herbivore performance	153	30	-0.0186	0.0744	-0.2500	0.8030	-0.1658	0.1286	0.01	9.962	1141.272
		Herbivore performance	→Plant performance	153	30	-0.1997	0.0774	-1.4469	0.1503	-0.4483	0.0695	0.13	9.962	1141.272
		Plant species richness	→Herbivore performance	153	30	-0.1894	0.1309	-2.5801	0.0110	-0.3528	-0.0466	0.01	9.962	1141.272
		Plant species richness	→Plant performance	153	30	0.4773	0.1346	3.5461	0.0005	0.2111	0.7435	0.13	9.962	1141.272
Organic croplands (temperate regions)	Binary plant species richness on tri-trophic interactions of plants, herbivores and natural enemies (bottom-up effects)	Plant species richness	→Plant performance	635	34	0.3782	0.0636	5.9465	4.6599×10^{-9}	0.2533	0.5031	0.04	15.004	4570.675
		Plant performance	→Herbivore performance	635	34	-0.2095	0.0337	-6.2166	9.3959×10^{-10}	-0.2757	-0.1433	0.32	15.004	4570.675
		Herbivore performance	→Natural enemy performance	635	34	-0.1114	0.0407	-14.2721	4.0995×10^{-40}	-0.9904	-0.7508	0.23	15.004	4570.675
		Plant species richness	→Herbivore performance	635	34	-0.8706	0.0610	-2.7371	0.0064	-0.1913	-0.0315	0.32	15.004	4570.675
		Plant species richness	→Natural enemy performance	635	34	0.7587	0.0737	10.2944	5.2223×10^{-23}	0.6140	0.9034	0.23	15.004	4570.675
		Plant species richness	→Natural enemy performance	635	34	0.8660	0.0630	13.7460	1.2709×10^{-37}	0.7423	0.9897	0.23	20.068	4558.253

	Binary plant species richness on tri-trophic interactions of plants, herbivores and natural enemies (top-down effects)	Natural enemy performance	→Herbivore performance	635	34	-0.1046	0.0385	-2.7169	0.0068	-0.1802	-0.0290	0.28	20.068	4558.253
		Herbivore performance	→Plant performance	635	34	-0.2765	0.0398	-12.2999	3.1744×10^{-31}	-0.9942	-0.7204	0.08	20.068	4558.253
		Plant species richness	→Herbivore performance	635	34	-0.8573	0.0697	-6.9472	9.8191×10^{-12}	-0.3547	-0.1983	0.28	20.068	4558.253
		Plant species richness	→Plant performance	635	34	0.1124	0.0722	1.5568	0.1201	-0.0294	0.2542	0.08	20.068	4558.253
Organic croplands (tropical regions)	Binary plant species richness on tri-trophic interactions of plants, herbivores and natural enemies (bottom-up effects)	Plant species richness	→Plant performance	554	21	0.3907	0.0548	7.1296	3.4338×10^{-12}	0.2830	0.4984	0.04	15.004	3746.616
		Plant performance	→Herbivore performance	554	21	-0.0197	0.0321	-0.6137	0.5397	-0.0828	0.0434	0.19	15.004	3746.616
		Herbivore performance	→Natural enemy performance	554	21	-0.0524	0.0429	-14.1149	1.4740×10^{-38}	-0.9792	-0.7400	0.19	15.004	3746.616
		Plant species richness	→Herbivore performance	554	21	-0.8596	0.0609	-1.2214	0.2225	-0.1367	0.0319	0.19	15.004	3746.616
		Plant species richness	→Natural enemy performance	554	21	0.7940	0.0807	9.8389	4.1118×10^{-21}	0.6355	0.9525	0.19	15.004	3746.616
	Binary plant species richness on tri-trophic interactions of plants, herbivores and natural enemies (top-down effects)	Plant species richness	→Natural enemy performance	554	21	0.8447	0.0700	12.0671	7.8436×10^{-30}	0.7072	0.9822	0.19	20.068	3742.171
		Natural enemy performance	→Herbivore performance	554	21	-0.0678	0.0356	-1.9045	0.0574	-0.1377	0.0021	0.2	20.068	3742.171
		Herbivore performance	→Plant performance	554	21	-0.0112	0.0332	-12.0912	8.3391×10^{-30}	-0.9403	-0.6775	0.05	20.068	3742.171
		Plant species richness	→Herbivore performance	554	21	-0.8089	0.0669	-0.3373	0.7360	-0.0764	0.0540	0.2	20.068	3742.171
		Plant species richness	→Plant performance	554	21	0.3871	0.0625	6.1936	1.2442×10^{-9}	0.2643	0.5099	0.05	20.068	3742.171
Organic croplands (herbaceous plants)	Binary plant species richness on tri-trophic interactions of plants, herbivores and natural enemies (bottom-up effects)	Plant species richness	→Plant performance	945	36	0.4140	0.0474	8.7342	1.2321×10^{-17}	0.3210	0.5070	0.04	15.004	6661.991
		Plant performance	→Herbivore performance	945	36	-0.1065	0.0280	-3.8036	0.0002	-0.1615	-0.0515	0.25	15.004	6661.991
		Herbivore performance	→Natural enemy performance	945	36	-0.0985	0.0326	-16.6725	1.2113×10^{-54}	-0.9616	-0.7590	0.21	15.004	6661.991
		Plant species richness	→Herbivore performance	945	36	-0.8603	0.0516	-3.0215	0.0026	-0.1625	-0.0345	0.25	15.004	6661.991
		Plant species richness	→Natural enemy performance	945	36	0.7647	0.0602	12.7027	3.9919×10^{-34}	0.6466	0.8828	0.21	15.004	6661.991
	Binary plant species richness on tri-trophic interactions of plants, herbivores and natural enemies (top-down effects)	Plant species richness	→Natural enemy performance	945	36	0.8583	0.0521	16.4741	1.4790×10^{-53}	0.7560	0.9606	0.21	20.068	6651.475
		Natural enemy performance	→Herbivore performance	945	36	-0.0932	0.0311	-2.9968	0.0028	-0.1542	-0.0322	0.25	20.068	6651.475
		Herbivore performance	→Plant performance	945	36	-0.1292	0.0295	-14.3118	5.3045×10^{-42}	-0.9342	-0.7088	0.05	20.068	6651.475
		Plant species richness	→Herbivore performance	945	36	-0.8215	0.0574	-4.3797	1.3277×10^{-5}	-0.1871	-0.0713	0.25	20.068	6651.475
		Plant species richness	→Plant performance	945	36	0.2967	0.0543	5.4641	6.1321×10^{-8}	0.1901	0.4033	0.05	20.068	6651.475
Organic croplands (woody plants)	Binary plant species richness on tri-trophic interactions of plants, herbivores and natural enemies (bottom-up effects)	Plant species richness	→Plant performance	244	19	0.2420	0.1042	2.3225	0.0211	0.0367	0.4473	0.01	15.004	1788.278
		Plant performance	→Herbivore performance	244	19	-0.1594	0.0457	-3.4880	0.0006	-0.2495	-0.0693	0.28	15.004	1788.278
		Herbivore performance	→Natural enemy performance	244	19	-0.0258	0.0675	-10.9859	1.0122×10^{-22}	-1.1013	-0.7663	0.19	15.004	1788.278
		Plant species richness	→Herbivore performance	244	19	-0.9338	0.0850	-0.3822	0.7027	-0.1588	0.1072	0.28	15.004	1788.278
		Plant species richness	→Natural enemy performance	244	19	0.7809	0.1244	6.2773	1.6436×10^{-9}	0.5358	1.0260	0.19	15.004	1788.278
	Binary plant species richness on tri-trophic interactions of plants, herbivores and natural enemies (top-down effects)	Plant species richness	→Natural enemy performance	244	19	0.8046	0.1065	7.5549	9.0856×10^{-13}	0.5948	1.0144	0.19	20.068	1788.316
		Natural enemy performance	→Herbivore performance	244	19	-0.0481	0.0524	-0.9179	0.3597	-0.1514	0.0552	0.26	20.068	1788.316
		Herbivore performance	→Plant performance	244	19	-0.2152	0.0644	-9.7058	8.5330×10^{-19}	-1.1233	-0.7441	0.04	20.068	1788.316
		Plant species richness	→Herbivore performance	244	19	-0.9337	0.0962	-3.3416	0.0010	-0.3421	-0.0883	0.26	20.068	1788.316
		Plant species richness	→Plant performance	244	19	0.0364	0.1192	0.3054	0.7604	-0.1985	0.2713	0.04	20.068	1788.316
Organic croplands (managed	Binary plant species richness on tri-trophic interactions of plants, herbivores and natural enemies (bottom-up effects)	Plant species richness	→Plant performance	1079	39	0.3309	0.0446	7.4193	2.4313×10^{-13}	0.2434	0.4184	0.02	15.004	7536.062
		Plant performance	→Herbivore performance	1079	39	-0.1038	0.0243	-4.2716	2.1176×10^{-5}	-0.1515	-0.0561	0.27	15.004	7536.062
		Herbivore performance	→Natural enemy performance	1079	39	-0.0812	0.0317	-21.3118	5.3946×10^{-84}	-1.0450	-0.8688	0.23	15.004	7536.062
		Plant species richness	→Herbivore performance	1079	39	-0.9569	0.0449	-2.5615	0.0106	-0.1434	-0.0190	0.27	15.004	7536.062
		Plant species richness	→Natural enemy performance	1079	39	0.7936	0.0585	13.5658	9.2605×10^{-39}	0.6788	0.9084	0.23	15.004	7536.062

experiment)	Binary plant species richness on tri-trophic interactions of plants, herbivores and natural enemies (top-down effects)	Plant species richness	→Natural enemy performance	1079	39	0.8747	0.0495	17.6707	2.0436×10^{-61}	0.7776	0.9718	0.22	20.068	7525.63
		Natural enemy performance	→Herbivore performance	1079	39	-0.0711	0.0274	-2.5949	0.0096	-0.1249	-0.0173	0.26	20.068	7525.63
		Herbivore performance	→Plant performance	1079	39	-0.1416	0.0283	-18.3419	2.2343×10^{-65}	-1.0274	-0.8288	0.04	20.068	7525.63
		Plant species richness	→Herbivore performance	1079	39	-0.9281	0.0506	-5.0035	6.6040×10^{-7}	-0.1971	-0.0861	0.26	20.068	7525.63
		Plant species richness	→Plant performance	1079	39	0.1891	0.0523	3.6157	0.0003	0.0865	0.2917	0.04	20.068	7525.63
Organic croplands (observational experiment)	Binary plant species richness on tri-trophic interactions of plants, herbivores and natural enemies (bottom-up effects)	Plant species richness	→Plant performance	110	16	0.8245	0.1592	5.1790	1.1496×10^{-6}	0.5087	1.1403	0.2	15.004	782.598
		Plant performance	→Herbivore performance	110	16	-0.4230	0.0846	-5.0000	2.7515×10^{-6}	-0.5910	-0.2550	0.16	15.004	782.598
		Herbivore performance	→Natural enemy performance	110	16	-0.0287	0.0653	0.7508	0.4546	-0.1957	0.4337	0.17	15.004	782.598
		Plant species richness	→Herbivore performance	110	16	0.1190	0.1585	-0.4395	0.6614	-0.1585	0.1011	0.16	15.004	782.598
		Plant species richness	→Natural enemy performance	110	16	0.7220	0.1235	5.8462	7.9780×10^{-8}	0.4766	0.9674	0.17	15.004	782.598
	Binary plant species richness on tri-trophic interactions of plants, herbivores and natural enemies (top-down effects)	Plant species richness	→Natural enemy performance	110	16	0.7460	0.1191	6.2636	1.1610×10^{-8}	0.5095	0.9825	0.17	20.068	785.69
		Natural enemy performance	→Herbivore performance	110	16	-0.0528	0.0900	-0.5867	0.5589	-0.2316	0.1260	0.02	20.068	785.69
		Herbivore performance	→Plant performance	110	16	-0.3192	0.0819	-1.2900	0.2002	-0.5522	0.1172	0.31	20.068	785.69
		Plant species richness	→Herbivore performance	110	16	-0.2175	0.1686	-3.8974	0.0002	-0.4818	-0.1566	0.02	20.068	785.69
		Plant species richness	→Plant performance	110	16	0.7652	0.1521	5.0309	2.2218×10^{-6}	0.4633	1.0671	0.31	20.068	785.69
Non-organic croplands (temperate regions)	Binary plant species richness on tri-trophic interactions of plants, herbivores and natural enemies (bottom-up effects)	Plant species richness	→Plant performance	863	48	0.1829	0.0553	3.3074	0.0010	0.0744	0.2914	0.01	0.63	6067.247
		Plant performance	→Herbivore performance	863	48	-0.0826	0.0276	-2.9928	0.0028	-0.1368	-0.0284	0.35	0.63	6067.247
		Herbivore performance	→Natural enemy performance	863	48	-0.1020	0.0366	-21.6646	7.0964×10^{-83}	-1.1696	-0.9752	0.24	0.63	6067.247
		Plant species richness	→Herbivore performance	863	48	-1.0724	0.0495	-2.7869	0.0054	-0.1738	-0.0302	0.35	0.63	6067.247
		Plant species richness	→Natural enemy performance	863	48	0.7965	0.0662	12.0317	7.1824×10^{-31}	0.6666	0.9264	0.24	0.63	6067.247
	Binary plant species richness on tri-trophic interactions of plants, herbivores and natural enemies (top-down effects)	Plant species richness	→Natural enemy performance	863	48	0.9143	0.0526	17.3821	5.41705×10^{-8}	0.8111	1.0175	0.23	0.431	6043.337
		Natural enemy performance	→Herbivore performance	863	48	-0.1036	0.0306	-3.3856	0.0007	-0.1637	-0.0435	0.35	0.431	6043.337
		Herbivore performance	→Plant performance	863	48	-0.1497	0.0381	-17.6779	1.3550×10^{-59}	-1.1097	-0.8879	0.02	0.431	6043.337
		Plant species richness	→Herbivore performance	863	48	-0.9988	0.0565	-3.9291	9.2449×10^{-5}	-0.2245	-0.0749	0.35	0.431	6043.337
		Plant species richness	→Plant performance	863	48	0.0208	0.0690	0.3014	0.7632	-0.1146	0.1562	0.02	0.431	6043.337
Non-organic croplands (tropical regions)	Binary plant species richness on tri-trophic interactions of plants, herbivores and natural enemies (bottom-up effects)	Plant species richness	→Plant performance	24	1	NA	NA	NA	NA	NA	NA	NA	NA	NA
		Plant performance	→Herbivore performance	24	1	NA	NA	NA	NA	NA	NA	NA	NA	NA
		Herbivore performance	→Natural enemy performance	24	1	NA	NA	NA	NA	NA	NA	NA	NA	NA
		Plant species richness	→Herbivore performance	24	1	NA	NA	NA	NA	NA	NA	NA	NA	NA
		Plant species richness	→Natural enemy performance	24	1	NA	NA	NA	NA	NA	NA	NA	NA	NA
	Binary plant species richness on tri-trophic interactions of plants, herbivores and natural enemies (top-down effects)	Plant species richness	→Natural enemy performance	24	1	NA	NA	NA	NA	NA	NA	NA	NA	NA
		Natural enemy performance	→Herbivore performance	24	1	NA	NA	NA	NA	NA	NA	NA	NA	NA
		Herbivore performance	→Plant performance	24	1	NA	NA	NA	NA	NA	NA	NA	NA	NA
		Plant species richness	→Herbivore performance	24	1	NA	NA	NA	NA	NA	NA	NA	NA	NA
		Plant species richness	→Plant performance	24	1	NA	NA	NA	NA	NA	NA	NA	NA	NA
Non-organic	Binary plant species richness on tri-trophic interactions of plants, herbivores and natural enemies (bottom-up effects)	Plant species richness	→Plant performance	405	36	0.1462	0.0815	1.7939	0.0736	-0.0140	0.3064	0.01	0.63	2967.615
		Plant performance	→Herbivore performance	405	36	-0.1930	0.0456	-4.2325	2.9282×10^{-5}	-0.2827	-0.1033	0.18	0.63	2967.615
		Herbivore performance	→Natural enemy performance	405	36	-0.0690	0.0502	-8.4428	6.7179×10^{-16}	-0.7734	-0.4812	0.08	0.63	2967.615
		Plant species richness	→Herbivore performance	405	36	-0.6273	0.0743	-1.3745	0.1701	-0.1677	0.0297	0.18	0.63	2967.615
		Plant species richness	→Natural enemy performance	405	36	0.4535	0.0844	5.3732	1.3391×10^{-7}	0.2876	0.6194	0.08	0.63	2967.615

croplands (herbaceous plants)	Binary plant species richness on tri-trophic interactions of plants, herbivores and natural enemies (top-down effects)	Plant species richness	→Natural enemy performance	405	36	0.4989	0.0777	6.4208	4.0690×10^{-10}	0.3461	0.6517	0.08	0.431	2974.056
		Natural enemy performance	→Herbivore performance	405	36	-0.0999	0.0446	-2.2399	0.0257	-0.1876	-0.0122	0.15	0.431	2974.056
		Herbivore performance	→Plant performance	405	36	-0.1794	0.0531	-7.7647	7.6871×10^{-14}	-0.7610	-0.4534	0.03	0.431	2974.056
		Plant species richness	→Herbivore performance	405	36	-0.6072	0.0782	-3.3785	0.0008	-0.2838	-0.0750	0.15	0.431	2974.056
		Plant species richness	→Plant performance	405	36	0.0269	0.0881	0.3053	0.7603	-0.1463	0.2001	0.03	0.431	2974.056
Non-organic croplands (woody plants)	Binary plant species richness on tri-trophic interactions of plants, herbivores and natural enemies (bottom-up effects)	Plant species richness	→Plant performance	482	13	0.2090	0.0642	3.2555	0.0012	0.0828	0.3352	0.01	0.63	3048.396
		Plant performance	→Herbivore performance	482	13	0.0014	0.0289	0.0484	0.9614	-0.0554	0.0582	0.61	0.63	3048.396
		Herbivore performance	→Natural enemy performance	482	13	0.0446	0.0568	-26.9639	3.5356×10^{-97}	-1.6027	-1.3849	0.42	0.63	3048.396
		Plant species richness	→Herbivore performance	482	13	-1.4938	0.0554	0.7852	0.4327	-0.0670	0.1562	0.61	0.63	3048.396
		Plant species richness	→Natural enemy performance	482	13	1.3089	0.1089	12.0193	3.8216×10^{-29}	1.0949	1.5229	0.42	0.63	3048.396
	Binary plant species richness on tri-trophic interactions of plants, herbivores and natural enemies (top-down effects)	Plant species richness	→Natural enemy performance	482	13	1.2491	0.0666	18.7553	5.7540×10^{-59}	1.1182	1.3800	0.42	0.431	3031.52
		Natural enemy performance	→Herbivore performance	482	13	0.0294	0.0375	0.7840	0.4334	-0.0443	0.1031	0.62	0.431	3031.52
		Herbivore performance	→Plant performance	482	13	0.0035	0.0542	-21.4548	2.3131×10^{-71}	-1.6839	-1.4013	0.01	0.431	3031.52
		Plant species richness	→Herbivore performance	482	13	-1.5426	0.0719	0.0646	0.9485	-0.1030	0.1100	0.62	0.431	3031.52
		Plant species richness	→Plant performance	482	13	0.2170	0.1038	2.0906	0.0371	0.0130	0.4210	0.01	0.431	3031.52
Non-organic croplands (managed experiment)	Binary plant species richness on tri-trophic interactions of plants, herbivores and natural enemies (bottom-up effects)	Plant species richness	→Plant performance	844	35	0.1988	0.0560	3.5500	0.0004	0.0889	0.3087	0.01	0.63	5855.697
		Plant performance	→Herbivore performance	844	35	-0.0854	0.0269	-3.1747	0.0016	-0.1382	-0.0326	0.39	0.63	5855.697
		Herbivore performance	→Natural enemy performance	844	35	-0.0609	0.0372	-23.6070	2.9188×10^{-94}	-1.2427	-1.0519	0.25	0.63	5855.697
		Plant species richness	→Herbivore performance	844	35	-1.1473	0.0486	-1.6371	0.1020	-0.1339	0.0121	0.39	0.63	5855.697
		Plant species richness	→Natural enemy performance	844	35	0.9055	0.0679	13.3358	7.4566×10^{-37}	0.7722	1.0388	0.25	0.63	5855.697
	Binary plant species richness on tri-trophic interactions of plants, herbivores and natural enemies (top-down effects)	Plant species richness	→Natural enemy performance	844	35	0.9829	0.0519	18.9383	1.4056×10^{-66}	0.8810	1.0848	0.25	0.431	5832.13
		Natural enemy performance	→Herbivore performance	844	35	-0.0787	0.0305	-2.5803	0.0100	-0.1386	-0.0188	0.38	0.431	5832.13
		Herbivore performance	→Plant performance	844	35	-0.1518	0.0399	-19.2910	1.5355×10^{-68}	-1.2051	-0.9825	0.03	0.431	5832.13
		Plant species richness	→Herbivore performance	844	35	-1.0938	0.0567	-3.8045	0.0002	-0.2301	-0.0735	0.38	0.431	5832.13
		Plant species richness	→Plant performance	844	35	0.0235	0.0726	0.3237	0.7463	-0.1190	0.1660	0.03	0.431	5832.13
Non-organic croplands (observational experiment)	Binary plant species richness on tri-trophic interactions of plants, herbivores and natural enemies (bottom-up effects)	Plant species richness	→Plant performance	43	14	-0.2493	0.2257	-1.1046	0.2788	-0.7116	0.2130	0.03	0.63	344.447
		Plant performance	→Herbivore performance	43	14	0.3374	0.1512	2.2315	0.0345	0.0265	0.6483	0.11	0.63	344.447
		Herbivore performance	→Natural enemy performance	43	14	0.1652	0.1444	0.1538	0.8789	-0.4208	0.4890	0.16	0.63	344.447
		Plant species richness	→Herbivore performance	43	14	0.0341	0.2217	1.1440	0.2631	-0.1317	0.4621	0.11	0.63	344.447
		Plant species richness	→Natural enemy performance	43	14	-0.5406	0.2117	-2.5536	0.0166	-0.9750	-0.1062	0.16	0.63	344.447
	Binary plant species richness on tri-trophic interactions of plants, herbivores and natural enemies (top-down effects)	Plant species richness	→Natural enemy performance	43	14	-0.5489	0.2123	-2.5855	0.0152	-0.9838	-0.1140	0.14	0.431	344.204
		Natural enemy performance	→Herbivore performance	43	14	0.1919	0.1677	1.1443	0.2629	-0.1528	0.5366	0.03	0.431	344.204
		Herbivore performance	→Plant performance	43	14	0.3280	0.1470	0.2249	0.8238	-0.4491	0.5597	0.13	0.431	344.204
		Plant species richness	→Herbivore performance	43	14	0.0553	0.2459	2.2313	0.0346	0.0257	0.6303	0.03	0.431	344.204
		Plant species richness	→Plant performance	43	14	-0.2329	0.2156	-1.0802	0.2896	-0.6753	0.2095	0.13	0.431	344.204

Table S8. Results of the path analyses for the bottom-up and top-down effects of binary plant species richness on the tri-trophic interactions of plant performance (i.e., plant growth, plant reproduction and plant quality), invertebrate herbivore performance (i.e., herbivore abundance, herbivore damage and herbivore diveristy) and their invertebrate natural enemy performance (i.e., predator abundance, predation, predator diversity, parasitoid abundance, parasitism and parasitoid diversity) in grasslands across different climatic regions, different plant types, and different study types (as presented also in figs. S13−15). The predictor and response columns specify the trophic group pairs and the moderator category. The estimate represents the strength of the relationship. The std. err. of estimate denotes the standardized error of the estimate coefficients for the fitted path-analytic models. The number of studies and observations for the predictor-response pair are also presented. Each test is two-sided and the original P value is reported with no multiple comparisons. In addition, test statistic (t value), and 95% confidence interval are reported. R^2 represents the proportion of variance explained for each endogenous variable, the reported R^2 is marginal, which represents variance explained by fixed effects only. Fisher's C statistic assesses the goodness-of-fit of the model through Shipley's test of directed separation. AICc is the adjusted Akaike´s information criterion, the strength of top-down and bottom-up pathways can be assessed using the differences in the AICc values.

Ecosystem type (other item)	Tri-trophic interaction (effect classification)	Predictor	Response	Number of observations	Number of studies	Estimate	Std.Err. of Estimate	t-value	P-value	CI_{lb}	CI_{ub}	R^2	Fisher´s C	AICc
Grasslands (temperate regions)	Binary plant species richness on tri-trophic interactions of plants, herbivores and natural enemies (bottom-up effects)	Plant species richness	→Plant performance	832	26	0.5415	0.0719	7.5313	1.3819×10^{-13}	0.4004	0.6826	0.06	16.298	6455.606
		Plant performance	→Herbivore performance	832	26	0.0590	0.0303	1.9472	0.0519	-0.0005	0.1185	0.02	16.298	6455.606
		Herbivore performance	→Natural enemy performance	832	26	0.2742	0.0346	3.4325	0.0006	0.0958	0.3518	0.09	16.298	6455.606
		Plant species richness	→Herbivore performance	832	26	0.2238	0.0652	7.9249	5.7810×10^{-14}	0.2061	0.3423	0.02	16.298	6455.606
		Plant species richness	→Natural enemy performance	832	26	0.1707	0.0709	2.4076	0.0163	0.0315	0.3099	0.09	16.298	6455.606
	Binary plant species richness on tri-trophic interactions of plants, herbivores and natural enemies (top-down effects)	Plant species richness	→Natural enemy performance	832	26	0.2393	0.0722	3.3144	0.0010	0.0976	0.3810	0.01	15.551	6455.793
		Natural enemy performance	→Herbivore performance	832	26	0.2153	0.0295	7.2983	7.4199×10^{-13}	0.1574	0.2732	0.06	15.551	6455.793
		Herbivore performance	→Plant performance	832	26	0.0530	0.0338	3.3177	0.0010	0.0836	0.3258	0.07	15.551	6455.793
		Plant species richness	→Herbivore performance	832	26	0.2047	0.0617	1.5680	0.1173	-0.0133	0.1193	0.06	15.551	6455.793
		Plant species richness	→Plant performance	832	26	0.5265	0.0725	7.2621	9.2130×10^{-13}	0.3842	0.6688	0.07	15.551	6455.793
Grasslands (tropical regions)	Binary plant species richness on tri-trophic interactions of plants, herbivores and natural enemies (bottom-up effects)	Plant species richness	→Plant performance	N/A	N/A	N/A	N/A	N/A	N/A	N/A	N/A	N/A	N/A	N/A
		Plant performance	→Herbivore performance	N/A	N/A	N/A	N/A	N/A	N/A	N/A	N/A	N/A	N/A	N/A
		Herbivore performance	→Natural enemy performance	N/A	N/A	N/A	N/A	N/A	N/A	N/A	N/A	N/A	N/A	N/A
		Plant species richness	→Herbivore performance	N/A	N/A	N/A	N/A	N/A	N/A	N/A	N/A	N/A	N/A	N/A
		Plant species richness	→Natural enemy performance	N/A	N/A	N/A	N/A	N/A	N/A	N/A	N/A	N/A	N/A	N/A
	Binary plant species richness on tri-trophic interactions of plants, herbivores and natural enemies (top-down effects)	Plant species richness	→Natural enemy performance	N/A	N/A	N/A	N/A	N/A	N/A	N/A	N/A	N/A	N/A	N/A
		Natural enemy performance	→Herbivore performance	N/A	N/A	N/A	N/A	N/A	N/A	N/A	N/A	N/A	N/A	N/A
		Herbivore performance	→Plant performance	N/A	N/A	N/A	N/A	N/A	N/A	N/A	N/A	N/A	N/A	N/A
		Plant species richness	→Herbivore performance	N/A	N/A	N/A	N/A	N/A	N/A	N/A	N/A	N/A	N/A	N/A

		Plant species richness	→Plant performance	N/A	N/A	N/A	N/A	N/A	N/A	N/A	N/A	N/A	N/A	N/A
Grasslands (herbaceous plants)	Binary plant species richness on tri-trophic interactions of plants, herbivores and natural enemies (bottom-up effects)	Plant species richness	→Plant performance	832	26	0.5415	0.0719	7.5313	1.3819×10^{-13}	0.4004	0.6826	0.06	16.298	6455.606
		Plant performance	→Herbivore performance	832	26	0.0590	0.0303	1.9472	0.0519	-0.0005	0.1185	0.02	16.298	6455.606
		Herbivore performance	→Natural enemy performance	832	26	0.2742	0.0346	3.4325	0.0006	0.0958	0.3518	0.09	16.298	6455.606
		Plant species richness	→Herbivore performance	832	26	0.2238	0.0652	7.9249	5.7810×10^{-14}	0.2061	0.3423	0.02	16.298	6455.606
		Plant species richness	→Natural enemy performance	832	26	0.1707	0.0709	2.4076	0.0163	0.0315	0.3099	0.09	16.298	6455.606
	Binary plant species richness on tri-trophic interactions of plants, herbivores and natural enemies (top-down effects)	Plant species richness	→Natural enemy performance	832	26	0.2393	0.0722	3.3144	0.0010	0.0976	0.3810	0.01	15.551	6455.793
		Natural enemy performance	→Herbivore performance	832	26	0.2153	0.0295	7.2983	7.4199×10^{-13}	0.1574	0.2732	0.06	15.551	6455.793
		Herbivore performance	→Plant performance	832	26	0.0530	0.0338	3.3177	0.0010	0.0836	0.3258	0.07	15.551	6455.793
		Plant species richness	→Herbivore performance	832	26	0.2047	0.0617	1.5680	0.1173	-0.0133	0.1193	0.06	15.551	6455.793
		Plant species richness	→Plant performance	832	26	0.5265	0.0725	7.2621	9.2130×10^{-13}	0.3842	0.6688	0.07	15.551	6455.793
Grasslands (woody plants)	Binary plant species richness on tri-trophic interactions of plants, herbivores and natural enemies (bottom-up effects)	Plant species richness	→Plant performance	N/A	N/A	N/A	N/A	N/A	N/A	N/A	N/A	N/A	N/A	N/A
		Plant performance	→Herbivore performance	N/A	N/A	N/A	N/A	N/A	N/A	N/A	N/A	N/A	N/A	N/A
		Herbivore performance	→Natural enemy performance	N/A	N/A	N/A	N/A	N/A	N/A	N/A	N/A	N/A	N/A	N/A
		Plant species richness	→Herbivore performance	N/A	N/A	N/A	N/A	N/A	N/A	N/A	N/A	N/A	N/A	N/A
		Plant species richness	→Natural enemy performance	N/A	N/A	N/A	N/A	N/A	N/A	N/A	N/A	N/A	N/A	N/A
	Binary plant species richness on tri-trophic interactions of plants, herbivores and natural enemies (top-down effects)	Plant species richness	→Natural enemy performance	N/A	N/A	N/A	N/A	N/A	N/A	N/A	N/A	N/A	N/A	N/A
		Natural enemy performance	→Herbivore performance	N/A	N/A	N/A	N/A	N/A	N/A	N/A	N/A	N/A	N/A	N/A
		Herbivore performance	→Plant performance	N/A	N/A	N/A	N/A	N/A	N/A	N/A	N/A	N/A	N/A	N/A
		Plant species richness	→Herbivore performance	N/A	N/A	N/A	N/A	N/A	N/A	N/A	N/A	N/A	N/A	N/A
		Plant species richness	→Plant performance	N/A	N/A	N/A	N/A	N/A	N/A	N/A	N/A	N/A	N/A	N/A
Grasslands (managed experiment)	Binary plant species richness on tri-trophic interactions of plants, herbivores and natural enemies (bottom-up effects)	Plant species richness	→Plant performance	738	13	0.5242	0.0758	6.9156	1.0498×10^{-11}	0.3754	0.6730	0.06	16.298	5742.332
		Plant performance	→Herbivore performance	738	13	0.0528	0.0322	1.6398	0.1015	-0.0104	0.1160	0.01	16.298	5742.332
		Herbivore performance	→Natural enemy performance	738	13	0.2637	0.0372	2.0729	0.0385	0.0075	0.2769	0.08	16.298	5742.332
		Plant species richness	→Herbivore performance	738	13	0.1422	0.0686	7.0887	4.8150×10^{-12}	0.1906	0.3368	0.01	16.298	5742.332
		Plant species richness	→Natural enemy performance	738	13	0.1652	0.0742	2.2264	0.0263	0.0195	0.3109	0.08	16.298	5742.332

	Binary plant species richness on tri-trophic interactions of plants, herbivores and natural enemies (top-down effects)	Plant species richness	→Natural enemy performance	738	13	0.2095	0.0761	2.7530	0.0061	0.0601	0.3589	0.01	15.551	5740.605
		Natural enemy performance	→Herbivore performance	738	13	0.2228	0.0313	7.1182	2.9148×10^{-12}	0.1613	0.2843	0.06	15.551	5740.605
		Herbivore performance	→Plant performance	738	13	0.0455	0.0359	1.9134	0.0561	-0.0032	0.2508	0.06	15.551	5740.605
		Plant species richness	→Herbivore performance	738	13	0.1238	0.0647	1.2674	0.2054	-0.0250	0.1160	0.06	15.551	5740.605
		Plant species richness	→Plant performance	738	13	0.5147	0.0762	6.7546	3.0129×10^{-11}	0.3651	0.6643	0.06	15.551	5740.605
Grasslands (observational experiment)	Binary plant species richness on tri-trophic interactions of plants, herbivores and natural enemies (bottom-up effects)	Plant species richness	→Plant performance	94	13	0.7318	0.2338	3.1300	0.0024	0.2671	1.1965	0.1	16.300	764.776
		Plant performance	→Herbivore performance	94	13	0.0752	0.0836	0.8995	0.3711	-0.0912	0.2416	0.23	16.300	764.776
		Herbivore performance	→Natural enemy performance	94	13	0.2699	0.1101	5.2868	9.9778×10^{-7}	0.6612	1.4588	0.1	16.300	764.776
		Plant species richness	→Herbivore performance	94	13	1.0600	0.2005	2.4514	0.0316	0.0285	0.5113	0.23	16.300	764.776
		Plant species richness	→Natural enemy performance	94	13	0.2125	0.2597	0.8183	0.4159	-0.3053	0.7303	0.1	16.300	764.776
	Binary plant species richness on tri-trophic interactions of plants, herbivores and natural enemies (top-down effects)	Plant species richness	→Natural enemy performance	94	13	0.4986	0.2392	2.0844	0.0401	0.0230	0.9742	0.04	15.552	768.122
		Natural enemy performance	→Herbivore performance	94	13	0.1187	0.0830	1.4301	0.1565	-0.0464	0.2838	0.24	15.552	768.122
		Herbivore performance	→Plant performance	94	13	0.0995	0.1099	5.4361	5.2717×10^{-7}	0.6688	1.4404	0.1	15.552	768.122
		Plant species richness	→Herbivore performance	94	13	1.0546	0.1940	0.9054	0.3678	-0.1189	0.3179	0.24	15.552	768.122
		Plant species richness	→Plant performance	94	13	0.6307	0.2593	2.4323	0.0172	0.1150	1.1464	0.1	15.552	768.122

Table S9. Results of the path analyses for the bottom-up and top-down effects of binary plant species richness on the tri-trophic interactions of plant performance (i.e., plant growth, plant reproduction and plant quality), invertebrate herbivore performance (i.e., herbivore abundance, herbivore damage and herbivore diveristy) and their invertebrate natural enemy performance (i.e., predator abundance, predation, predator diversity, parasitoid abundance, parasitism and parasitoid diversity) in forests across different climatic regions, different plant types, and different study types (as presented also in figs. S16−18). The predictor and response columns specify the trophic group pairs and the moderator category. The estimate represents the strength of the relationship. The std. err. of estimate denotes the standardized error of the estimate coefficients for the fitted path-analytic models. The number of studies and observations for the predictor-response pair are also presented. Each test is two-sided and the original P value is reported with no multiple comparisons. In addition, test statistic (t value), and 95% confidence interval are reported. R^2 represents the proportion of variance explained for each endogenous variable, the reported R^2 is marginal, which represents variance explained by fixed effects only. Fisher's C statistic assesses the goodness-of-fit of the model through Shipley's test of directed separation. AICc is the adjusted Akaike´s information criterion, the strength of top-down and bottom-up pathways can be assessed using the differences in the AICc values.

Ecosystem type (other item)	Tri-trophic interaction (effect classification)	Predictor	Response	Number of observations	Number of studies	Estimate	Std.Err. of Estimate	t-value	P-value	CI_{lb}	CI_{ub}	R^2	Fisher´s C	AICc
Forests (temperate regions)	Binary plant species richness on tri-trophic interactions of plants, herbivores and natural enemies (bottom-up effects)	Plant species richness	→Plant performance	291	16	0.9893	0.1207	8.1964	1.0813×10^{-14}	0.7516	1.2270	0.19	2.362	2289.266
		Plant performance	→Herbivore performance	291	16	0.0816	0.0594	1.3737	0.1706	-0.0353	0.1985	0.02	2.362	2289.266
		Herbivore performance	→Natural enemy performance	291	16	0.1537	0.0577	1.3220	0.1872	-0.0895	0.4557	0.07	2.362	2289.266
		Plant species richness	→Herbivore performance	291	16	0.1831	0.1385	2.6638	0.0084	0.0398	0.2676	0.02	2.362	2289.266
		Plant species richness	→Natural enemy performance	291	16	0.4706	0.1252	3.7588	0.0002	0.2241	0.7171	0.07	2.362	2289.266
	Binary plant species richness on tri-trophic interactions of plants, herbivores and natural enemies (top-down effects)	Plant species richness	→Natural enemy performance	291	16	0.5184	0.1252	4.1406	4.5775×10^{-5}	0.2720	0.7648	0.05	1.922	2290.673
		Natural enemy performance	→Herbivore performance	291	16	0.1453	0.0575	2.5270	0.0121	0.0321	0.2585	0.03	1.922	2290.673
		Herbivore performance	→Plant performance	291	16	0.0629	0.0534	1.5650	0.1187	-0.0509	0.4459	0.19	1.922	2290.673
		Plant species richness	→Herbivore performance	291	16	0.1975	0.1262	1.1779	0.2399	-0.0422	0.1680	0.03	1.922	2290.673
		Plant species richness	→Plant performance	291	16	0.9713	0.1215	7.9942	4.1714×10^{-14}	0.7321	1.2105	0.19	1.922	2290.673
Forests (tropical regions)	Binary plant species richness on tri-trophic interactions of plants, herbivores and natural enemies (bottom-up effects)	Plant species richness	→Plant performance	42	3	0.7592	0.4549	1.6689	0.1030	-0.1602	1.6786	0.06	2.365	364.541
		Plant performance	→Herbivore performance	42	3	0.2327	0.1147	2.0288	0.0499	0.0001	0.4653	0.07	2.365	364.541
		Herbivore performance	→Natural enemy performance	42	3	0.3150	0.1472	1.3102	0.1982	-0.2481	1.1561	0.09	2.365	364.541
		Plant species richness	→Herbivore performance	42	3	0.4540	0.3465	2.1399	0.0388	0.0170	0.6130	0.07	2.365	364.541
		Plant species richness	→Natural enemy performance	42	3	0.3510	0.3356	1.0459	0.3027	-0.3298	1.0318	0.09	2.365	364.541
	Binary plant species richness on tri-trophic interactions of plants, herbivores and natural enemies (top-down effects)	Plant species richness	→Natural enemy performance	42	3	0.5461	0.3416	1.5987	0.1184	-0.1460	1.2382	0.03	1.922	369.127
		Natural enemy performance	→Herbivore performance	42	3	0.1255	0.1171	1.0717	0.2909	-0.1119	0.3629	0.04	1.922	369.127
		Herbivore performance	→Plant performance	42	3	0.2305	0.1535	1.6356	0.1104	-0.1374	1.2872	0.11	1.922	369.127

		Plant species richness	→Herbivore performance	42	3	0.5749	0.3515	1.5016	0.1416	-0.0804	0.5414	0.04	1.922	369.127
		Plant species richness	→Plant performance	42	3	0.6250	0.4568	1.3682	0.1791	-0.2990	1.5490	0.11	1.922	369.127
Forests (herbaceous plants)	Binary plant species richness on tri-trophic interactions of plants, herbivores and natural enemies (bottom-up effects)	Plant species richness	→Plant performance	N/A	N/A	N/A	N/A	N/A	N/A	N/A	N/A	N/A	N/A	N/A
		Plant performance	→Herbivore performance	N/A	N/A	N/A	N/A	N/A	N/A	N/A	N/A	N/A	N/A	N/A
		Herbivore performance	→Natural enemy performance	N/A	N/A	N/A	N/A	N/A	N/A	N/A	N/A	N/A	N/A	N/A
		Plant species richness	→Herbivore performance	N/A	N/A	N/A	N/A	N/A	N/A	N/A	N/A	N/A	N/A	N/A
		Plant species richness	→Natural enemy performance	N/A	N/A	N/A	N/A	N/A	N/A	N/A	N/A	N/A	N/A	N/A
	Binary plant species richness on tri-trophic interactions of plants, herbivores and natural enemies (top-down effects)	Plant species richness	→Natural enemy performance	N/A	N/A	N/A	N/A	N/A	N/A	N/A	N/A	N/A	N/A	N/A
		Natural enemy performance	→Herbivore performance	N/A	N/A	N/A	N/A	N/A	N/A	N/A	N/A	N/A	N/A	N/A
		Herbivore performance	→Plant performance	N/A	N/A	N/A	N/A	N/A	N/A	N/A	N/A	N/A	N/A	N/A
		Plant species richness	→Herbivore performance	N/A	N/A	N/A	N/A	N/A	N/A	N/A	N/A	N/A	N/A	N/A
		Plant species richness	→Plant performance	N/A	N/A	N/A	N/A	N/A	N/A	N/A	N/A	N/A	N/A	N/A
Forests (woody plants)	Binary plant species richness on tri-trophic interactions of plants, herbivores and natural enemies (bottom-up effects)	Plant species richness	→Plant performance	333	19	0.9642	0.1169	8.2481	4.5500×10^{-15}	0.7342	1.1942	0.17	2.362	2613.338
		Plant performance	→Herbivore performance	333	19	0.1052	0.0539	1.9518	0.0518	-0.0008	0.2112	0.02	2.362	2613.338
		Herbivore performance	→Natural enemy performance	333	19	0.1292	0.0547	1.3935	0.1644	-0.0741	0.4339	0.06	2.362	2613.338
		Plant species richness	→Herbivore performance	333	19	0.1799	0.1291	2.3620	0.0188	0.0215	0.2369	0.02	2.362	2613.338
		Plant species richness	→Natural enemy performance	333	19	0.4415	0.1203	3.6700	0.0003	0.2048	0.6782	0.06	2.362	2613.338
	Binary plant species richness on tri-trophic interactions of plants, herbivores and natural enemies (top-down effects)	Plant species richness	→Natural enemy performance	333	19	0.4855	0.1197	4.0560	$6.25725\times10^{-}$	0.2500	0.7210	0.04	1.922	2614.129
		Natural enemy performance	→Herbivore performance	333	19	0.1277	0.0518	2.4653	0.0142	0.0258	0.2296	0.03	1.922	2614.129
		Herbivore performance	→Plant performance	333	19	0.0839	0.0504	1.8884	0.0599	-0.0094	0.4596	0.18	1.922	2614.129
		Plant species richness	→Herbivore performance	333	19	0.2251	0.1192	1.6647	0.0970	-0.0153	0.1831	0.03	1.922	2614.129
		Plant species richness	→Plant performance	333	19	0.9383	0.1176	7.9787	2.8818×10^{-14}	0.7069	1.1697	0.18	1.922	2614.129
Forests	Binary plant species richness on tri-trophic interactions of plants, herbivores and natural enemies (bottom-up effects)	Plant species richness	→Plant performance	220	11	1.2092	0.1266	9.5513	5.1980×10^{-18}	0.9595	1.4589	0.29	2.362	1646.983
		Plant performance	→Herbivore performance	220	11	-0.0233	0.0650	-0.3585	0.7204	-0.1515	0.1049	0.04	2.362	1646.983
		Herbivore performance	→Natural enemy performance	220	11	0.1511	0.0665	3.3795	0.0009	0.2053	0.7815	0.07	2.362	1646.983
		Plant species richness	→Herbivore performance	220	11	0.4934	0.1460	2.2722	0.0256	0.0189	0.2833	0.04	2.362	1646.983

(managed experiment)		Plant species richness	→Natural enemy performance	220	11	0.4227	0.1308	3.2317	0.0015	0.1641	0.6813	0.07	2.362	1646.983
	Binary plant species richness on tri-trophic interactions of plants, herbivores and natural enemies (top-down effects)	Plant species richness	→Natural enemy performance	220	11	0.4943	0.1282	3.8557	0.0002	0.2414	0.7472	0.05	1.922	1649.312
		Natural enemy performance	→Herbivore performance	220	11	0.1080	0.0644	1.6770	0.0950	-0.0190	0.2350	0.05	1.922	1649.312
		Herbivore performance	→Plant performance	220	11	-0.0363	0.0584	3.2667	0.0013	0.1631	0.6601	0.29	1.922	1649.312
		Plant species richness	→Herbivore performance	220	11	0.4116	0.1260	-0.6216	0.5349	-0.1514	0.0788	0.05	1.922	1649.312
		Plant species richness	→Plant performance	220	11	1.2287	0.1306	9.4081	1.4748×10^{-17}	0.9711	1.4863	0.29	1.922	1649.312
Forests (observational experiment)	Binary plant species richness on tri-trophic interactions of plants, herbivores and natural enemies (bottom-up effects)	Plant species richness	→Plant performance	113	8	0.3926	0.2547	1.5414	0.1266	-0.1132	0.8984	0.02	2.362	965.899
		Plant performance	→Herbivore performance	113	8	0.2366	0.0897	2.6377	0.0096	0.0587	0.4145	0.06	2.362	965.899
		Herbivore performance	→Natural enemy performance	113	8	0.1245	0.0888	-1.4901	0.1392	-0.8758	0.1242	0.03	2.362	965.899
		Plant species richness	→Herbivore performance	113	8	-0.3758	0.2522	1.4020	0.1640	-0.0517	0.3007	0.06	2.362	965.899
		Plant species richness	→Natural enemy performance	113	8	0.4827	0.2425	1.9905	0.0491	0.0020	0.9634	0.03	2.362	965.899
	Binary plant species richness on tri-trophic interactions of plants, herbivores and natural enemies (top-down effects)	Plant species richness	→Natural enemy performance	113	8	0.4502	0.2426	1.8557	0.0662	-0.0307	0.9311	0.02	1.922	967.159
		Natural enemy performance	→Herbivore performance	113	8	0.0900	0.0928	0.9698	0.3344	-0.0940	0.2740	0.02	1.922	967.159
		Herbivore performance	→Plant performance	113	8	0.2355	0.0922	-1.2160	0.2267	-0.8291	0.1987	0.07	1.922	967.159
		Plant species richness	→Herbivore performance	113	8	-0.3152	0.2592	2.5542	0.0121	0.0527	0.4183	0.02	1.922	967.159
		Plant species richness	→Plant performance	113	8	0.4632	0.2501	1.8521	0.0673	-0.0336	0.9600	0.07	1.922	967.159

Table S10. Results of the path analyses for the bottom-up and top-down effects of number of added plant species richness on the tri-trophic interactions of plant performance (i.e., plant growth, plant reproduction and plant quality), invertebrate herbivore performance (i.e., herbivore abundance, herbivore damage and herbivore diveristy) and their invertebrate natural enemy performance (i.e., predator abundance, predation, predator diversity, parasitoid abundance, parasitism and parasitoid diversity) in global terrestrial ecosystems, organic and non-organic croplands, grasslands and forests. The predictor and response columns specify the trophic group pairs and the moderator category. The estimate represents the strength of the relationship. The std. err. of estimate denotes the standardized error of the estimate coefficients for the fitted path-analytic models. The number of studies and observations for the predictor-response pair are also presented. Each test is two-sided and the original P value is reported with no multiple comparisons. In addition, test statistic (t value), and 95% confidence interval are reported. R^2 represents the proportion of variance explained for each endogenous variable, the reported R^2 is marginal, which represents variance explained by fixed effects only. Fisher's C statistic assesses the goodness-of-fit of the model through Shipley's test of directed separation. AICc is the adjusted Akaike´s information criterion, the strength of top-down and bottom-up pathways can be assessed using the differences in the AICc values.

Ecosystem type (other item)	Tri-trophic interaction (effect classification)	Predictor	Response	Number of observations	Number of studies	Estimate	Std.Err. of Estimate	t-value	P-value	CI_{lb}	CI_{ub}	R^2	Fisher´s C	AICc
Global terrestrial ecosystems	Number of added plant species richness on tri-trophic interactions of plants, herbivores and natural enemies (bottom-up effects)	Plant species richness	→Plant performance	3241	149	0.0029	0.0012	2.4167	0.0172	0.0005	0.0053	0.00	60.496	25456.729
		Plant performance	→Herbivore performance	3241	149	-0.0749	0.0169	-4.4320	9.6531×10^{-6}	-0.1080	-0.0418	0.01	60.496	25456.729
		Herbivore performance	→Natural enemy performance	3241	149	-0.1348	0.0175	4.5000	1.4295×10^{-5}	0.0030	0.0078	0.04	60.496	25456.729
		Plant species richness	→Herbivore performance	3241	149	0.0054	0.0012	-7.7029	1.7640×10^{-14}	-0.1691	-0.1005	0.01	60.496	25456.729
		Plant species richness	→Natural enemy performance	3241	149	0.0114	0.0012	9.5000	6.70821×10^{-7}	0.0090	0.0138	0.04	60.496	25456.729
	Number of added plant species richness on tri-trophic interactions of plants, herbivores and natural enemies (top-down effects)	Plant species richness	→Natural enemy performance	3241	149	0.0106	0.0013	8.1538	1.7422×10^{-13}	0.0080	0.0132	0.02	64.305	25441.52
		Natural enemy performance	→Herbivore performance	3241	149	-0.1362	0.0170	-8.0118	1.5701×10^{-15}	-0.1695	-0.1029	0.02	64.305	25441.52
		Herbivore performance	→Plant performance	3241	149	-0.0834	0.0166	5.5833	1.2192×10^{-7}	0.0043	0.0091	0.01	64.305	25441.52
		Plant species richness	→Herbivore performance	3241	149	0.0067	0.0012	-5.0241	5.3377×10^{-7}	-0.1159	-0.0509	0.02	64.305	25441.52
		Plant species richness	→Plant performance	3241	149	0.0032	0.0012	2.6667	0.0087	0.0008	0.0056	0.01	64.305	25441.52
Organic croplands	Number of added plant species richness on tri-trophic interactions of plants, herbivores and natural enemies (bottom-up effects)	Plant species richness	→Plant performance	1189	55	0.0005	0.0075	0.0667	0.9469	-0.0142	0.0152	0.00	60.496	8958.88
		Plant performance	→Herbivore performance	1189	55	-0.2093	0.0270	-7.7519	1.9853×10^{-14}	-0.2623	-0.1563	0.06	60.496	8958.88
		Herbivore performance	→Natural enemy performance	1189	55	-0.2686	0.0276	-4.6267	7.1289×10^{-6}	-0.0495	-0.0199	0.10	60.496	8958.88
		Plant species richness	→Herbivore performance	1189	55	-0.0347	0.0075	-9.7319	1.4742×10^{-21}	-0.3228	-0.2144	0.06	60.496	8958.88
		Plant species richness	→Natural enemy performance	1189	55	0.0423	0.0079	5.3544	1.7308×10^{-7}	0.0268	0.0578	0.10	60.496	8958.88
	Number of added plant species richness on tri-trophic interactions of plants, herbivores and natural enemies (top-down effects)	Plant species richness	→Natural enemy performance	1189	55	0.0573	0.0081	7.0741	8.7876×10^{-12}	0.0414	0.0732	0.04	64.305	8918.75
		Natural enemy performance	→Herbivore performance	1189	55	-0.2801	0.0270	-10.3741	3.5724×10^{-24}	-0.3331	-0.2271	0.09	64.305	8918.75
		Herbivore performance	→Plant performance	1189	55	-0.2215	0.0239	-3.0811	0.0024	-0.0374	-0.0082	0.04	64.305	8918.75
		Plant species richness	→Herbivore performance	1189	55	-0.0228	0.0074	-9.2678	9.0952×10^{-20}	-0.2684	-0.1746	0.09	64.305	8918.75
		Plant species richness	→Plant performance	1189	55	-0.0132	0.0074	-1.7838	0.0752	-0.0277	0.0013	0.04	64.305	8918.75
Non-organic	Number of added plant species richness on tri-trophic interactions of plants,	Plant species richness	→Plant performance	887	49	0.0272	0.0164	1.6585	0.0987	-0.0051	0.0595	0.00	60.496	6685.753
		Plant performance	→Herbivore performance	887	49	-0.1372	0.0325	-4.2215	2.6861×10^{-5}	-0.2010	-0.0734	0.09	60.496	6685.753
		Herbivore performance	→Natural enemy performance	887	49	-0.3077	0.0317	-8.2331	2.3425×10^{-12}	-0.1666	-0.1018	0.16	60.496	6685.753

croplands	herbivores and natural enemies (bottom-up effects)	Plant species richness	→Herbivore performance	887	49	-0.1342	0.0163	-9.7066	3.8839×10^{-21}	-0.3699	-0.2455	0.09	60.496	6685.753
		Plant species richness	→Natural enemy performance	887	49	0.1052	0.0161	6.5342	7.2349×10^{-9}	0.0731	0.1373	0.16	60.496	6685.753
	Number of added plant species richness on tri-trophic interactions of plants, herbivores and natural enemies (top-down effects)	Plant species richness	→Natural enemy performance	887	49	0.1448	0.0155	9.3419	3.5437×10^{-14}	0.1139	0.1757	0.08	64.305	6666.709
		Natural enemy performance	→Herbivore performance	887	49	-0.3201	0.0317	-10.0978	1.1187×10^{-22}	-0.3823	-0.2579	0.16	64.305	6666.709
		Herbivore performance	→Plant performance	887	49	-0.1514	0.0317	-5.4049	8.3726×10^{-7}	-0.1206	-0.0556	0.02	64.305	6666.709
		Plant species richness	→Herbivore performance	887	49	-0.0881	0.0163	-4.7760	2.1213×10^{-6}	-0.2136	-0.0892	0.16	64.305	6666.709
		Plant species richness	→Plant performance	887	49	0.0010	0.0178	0.0562	0.9553	-0.0342	0.0362	0.02	64.305	6666.709
Grasslands	Number of added plant species richness on tri-trophic interactions of plants, herbivores and natural enemies (bottom-up effects)	Plant species richness	→Plant performance	832	26	0.0017	0.0014	1.2143	0.2303	-0.0011	0.0045	0.00	60.507	6491.328
		Plant performance	→Herbivore performance	832	26	0.0781	0.0290	2.6931	0.0072	0.0212	0.1350	0.04	60.507	6491.328
		Herbivore performance	→Natural enemy performance	832	26	0.2288	0.0354	5.5000	7.6680×10^{-7}	0.0042	0.0090	0.10	60.507	6491.328
		Plant species richness	→Herbivore performance	832	26	0.0066	0.0012	6.4633	2.0721×10^{-10}	0.1593	0.2983	0.04	60.507	6491.328
		Plant species richness	→Natural enemy performance	832	26	0.0075	0.0013	5.7692	2.3190×10^{-7}	0.0049	0.0101	0.1	60.507	6491.328
	Number of added plant species richness on tri-trophic interactions of plants, herbivores and natural enemies (top-down effects)	Plant species richness	→Natural enemy performance	832	26	0.0090	0.0013	6.9231	3.9788×10^{-9}	0.0064	0.0116	0.05	64.43	6491.277
		Natural enemy performance	→Herbivore performance	832	26	0.1979	0.0300	6.5967	7.6317×10^{-11}	0.1390	0.2568	0.06	64.43	6491.277
		Herbivore performance	→Plant performance	832	26	0.0816	0.0348	4.0833	0.0001	0.0025	0.0073	0.01	64.43	6491.277
		Plant species richness	→Herbivore performance	832	26	0.0049	0.0012	2.3448	0.0193	0.0133	0.1499	0.06	64.43	6491.277
		Plant species richness	→Plant performance	832	26	0.0014	0.0014	1.0000	0.3222	-0.0014	0.0042	0.01	64.43	6491.277
Forests	Number of added plant species richness on tri-trophic interactions of plants, herbivores and natural enemies (bottom-up effects)	Plant species richness	→Plant performance	333	19	0.0011	0.0071	0.1549	0.8772	-0.0130	0.0152	0.00	60.507	2658.257
		Plant performance	→Herbivore performance	333	19	0.1342	0.0457	2.9365	0.0036	0.0443	0.2241	0.14	60.507	2658.257
		Herbivore performance	→Natural enemy performance	333	19	0.1273	0.0577	7.0312	3.0353×10^{-11}	0.0324	0.0576	0.03	60.507	2658.257
		Plant species richness	→Herbivore performance	333	19	0.0450	0.0064	2.2062	0.0283	0.0137	0.2409	0.14	60.507	2658.257
		Plant species richness	→Natural enemy performance	333	19	0.0134	0.0073	1.8356	0.0689	-0.0011	0.0279	0.03	60.507	2658.257
	Number of added plant species richness on tri-trophic interactions of plants, herbivores and natural enemies (top-down effects)	Plant species richness	→Natural enemy performance	333	19	0.0185	0.0070	2.6429	0.0091	0.0047	0.0323	0.02	64.43	2661.872
		Natural enemy performance	→Herbivore performance	333	19	0.0877	0.0488	1.7971	0.0733	-0.0083	0.1837	0.12	64.43	2661.872
		Herbivore performance	→Plant performance	333	19	0.1435	0.0562	6.5152	4.9885×10^{-10}	0.0300	0.0560	0.02	64.43	2661.872
		Plant species richness	→Herbivore performance	333	19	0.0430	0.0066	2.5534	0.0111	0.0329	0.2541	0.12	64.43	2661.872
		Plant species richness	→Plant performance	333	19	-0.0034	0.0073	-0.4658	0.6426	-0.0179	0.0111	0.02	64.43	2661.872

Table S11. Results of the path analyses for the bottom-up and top-down effects of number of added plant species richness on the tri-trophic interactions of plant performance (i.e., plant growth, plant reproduction and plant quality), invertebrate herbivore performance (i.e., herbivore abundance, herbivore damage and herbivore diveristy) and their invertebrate natural enemy performance (i.e., predator abundance, predation, predator diversity, parasitoid abundance, parasitism and parasitoid diversity) in croplands across different climatic regions, different plant types, and different study types. The predictor and response columns specify the trophic group pairs and the moderator category. The estimate represents the strength of the relationship. The std. err. of estimate denotes the standardized error of the estimate coefficients for the fitted path-analytic models. The number of studies and observations for the predictor-response pair are also presented. Each test is two-sided and the original P value is reported with no multiple comparisons. In addition, test statistic (t value), and 95% confidence interval are reported. R^2 represents the proportion of variance explained for each endogenous variable, the reported R^2 is marginal, which represents variance explained by fixed effects only. Fisher's C statistic assesses the goodness-of-fit of the model through Shipley's test of directed separation. AICc is the adjusted Akaike´s information criterion, the strength of top-down and bottom-up pathways can be assessed using the differences in the AICc values.

Ecosystem type (other item)	Tri-trophic interaction (effect classification)	Predictor	Response	Number of observations	Number of studies	Estimate	Std.Err. of Estimate	t-value	P-value	CI_{lb}	CI_{ub}	R^2	Fisher´s C	AICc
Croplands (temperate regions)	Number of added plant species richness on tri-trophic interactions of plants, herbivores and natural enemies (bottom-up effects)	Plant species richness	→Plant performance	1498	82	0.0047	0.0054	0.8704	0.3841	-0.0059	0.0153	0.00	12.492	12597.783
		Plant performance	→Herbivore performance	1498	82	-0.1975	0.0235	-8.4043	5.0488×10^{-17}	-0.2436	-0.1514	0.02	12.492	12597.783
		Herbivore performance	→Natural enemy performance	1498	82	-0.2597	0.0250	0.3750	0.7077	-0.0051	0.0075	0.05	12.492	12597.783
		Plant species richness	→Herbivore performance	1498	82	0.0012	0.0032	-10.3880	4.6301×10^{-25}	-0.3087	-0.2107	0.02	12.492	12597.783
		Plant species richness	→Natural enemy performance	1498	82	0.0361	0.0032	11.2812	2.7721×10^{-29}	0.0298	0.0424	0.05	12.492	12597.783
	Number of added plant species richness on tri-trophic interactions of plants, herbivores and natural enemies (top-down effects)	Plant species richness	→Natural enemy performance	1498	82	0.0336	0.0032	10.5000	1.2838×10^{-25}	0.0273	0.0399	0.01	18.944	12574.75
		Natural enemy performance	→Herbivore performance	1498	82	-0.2525	0.0242	-10.4339	2.7009×10^{-25}	-0.2999	-0.2051	0.03	18.944	12574.75
		Herbivore performance	→Plant performance	1498	82	-0.2186	0.0244	3.1515	0.0016	0.0039	0.0169	0.02	18.944	12574.75
		Plant species richness	→Herbivore performance	1498	82	0.0104	0.0033	-8.9590	4.2457×10^{-19}	-0.2664	-0.1708	0.03	18.944	12574.75
		Plant species richness	→Plant performance	1498	82	0.0043	0.0053	0.8113	0.4172	-0.0061	0.0147	0.02	18.944	12574.75
Croplands (tropical regions)	Number of added plant species richness on tri-trophic interactions of plants, herbivores and natural enemies (bottom-up effects)	Plant species richness	→Plant performance	578	22	0.1162	0.0192	6.0521	1.6514×10^{-9}	0.0785	0.1539	0.02	12.492	4407.56
		Plant performance	→Herbivore performance	578	22	0.0053	0.0315	0.1683	0.8664	-0.0565	0.0671	0.11	12.492	4407.56
		Herbivore performance	→Natural enemy performance	578	22	-0.1089	0.0439	-13.2683	1.5111×10^{-34}	-0.3123	-0.2317	0.05	12.492	4407.56
		Plant species richness	→Herbivore performance	578	22	-0.2720	0.0205	-2.4806	0.0132	-0.1950	-0.0228	0.11	12.492	4407.56
		Plant species richness	→Natural enemy performance	578	22	0.1608	0.0268	6.0000	4.7260×10^{-9}	0.1081	0.2135	0.05	12.492	4407.56
	Number of added plant species richness on tri-trophic interactions of plants, herbivores and natural enemies (top-down effects)	Plant species richness	→Natural enemy performance	578	22	0.1887	0.0245	7.7020	9.5588×10^{-14}	0.1405	0.2369	0.04	18.944	4404.38
		Natural enemy performance	→Herbivore performance	578	22	-0.0978	0.0319	-3.0658	0.0022	-0.1603	-0.0353	0.11	18.944	4404.38
		Herbivore performance	→Plant performance	578	22	-0.0155	0.0337	-12.0813	6.3485×10^{-29}	-0.2936	-0.2114	0.02	18.944	4404.38
		Plant species richness	→Herbivore performance	578	22	-0.2525	0.0209	-0.4599	0.6456	-0.0816	0.0506	0.11	18.944	4404.38
		Plant species richness	→Plant performance	578	22	0.1123	0.0213	5.2723	1.4633×10^{-7}	0.0705	0.1541	0.02	18.944	4404.38
	Number of added plant species richness on tri-trophic interactions of plants, herbivores and natural enemies (bottom-up effects)	Plant species richness	→Plant performance	1350	72	-0.0054	0.0069	-0.7826	0.4339	-0.0189	0.0081	0.00	12.492	11085.407
		Plant performance	→Herbivore performance	1350	72	-0.1134	0.0240	-4.7250	2.3386×10^{-6}	-0.1604	-0.0664	0.01	12.492	11085.407
		Herbivore performance	→Natural enemy performance	1350	72	-0.2435	0.0274	-5.3250	1.1364×10^{-7}	-0.0583	-0.0269	0.03	12.492	11085.407
		Plant species richness	→Herbivore performance	1350	72	-0.0426	0.0080	-8.8869	8.2226×10^{-19}	-0.2972	-0.1898	0.01	12.492	11085.407
		Plant species richness	→Natural enemy performance	1350	72	0.0354	0.0062	5.7097	1.1687×10^{-8}	0.0232	0.0476	0.03	12.492	11085.407

Croplands (herbaceous plants)	Number of added plant species richness on tri-trophic interactions of plants, herbivores and natural enemies (top-down effects)	Plant species richness	→Natural enemy performance	1350	72	0.0384	0.0064	6.0000	2.0512×10^{-9}	0.0259	0.0509	0.00	18.944	11067.501
		Natural enemy performance	→Herbivore performance	1350	72	-0.1964	0.0238	-8.2521	1.7967×10^{-16}	-0.2431	-0.1497	0.02	18.944	11067.501
		Herbivore performance	→Plant performance	1350	72	-0.1792	0.0248	-3.9500	8.1309×10^{-5}	-0.0473	-0.0159	0.01	18.944	11067.501
		Plant species richness	→Herbivore performance	1350	72	-0.0316	0.0080	-7.2258	5.5783×10^{-13}	-0.2278	-0.1306	0.02	18.944	11067.501
		Plant species richness	→Plant performance	1350	72	-0.0083	0.0068	-1.2206	0.2223	-0.0216	0.0050	0.01	18.944	11067.501
Croplands (woody plants)	Number of added plant species richness on tri-trophic interactions of plants, herbivores and natural enemies (bottom-up effects)	Plant species richness	→Plant performance	726	32	-0.0101	0.0077	-1.3117	0.1898	-0.0252	0.0050	0.00	12.492	6289.576
		Plant performance	→Herbivore performance	726	32	-0.2137	0.0344	-6.2122	5.8088×10^{-10}	-0.2811	-0.1463	0.02	12.492	6289.576
		Herbivore performance	→Natural enemy performance	726	32	-0.2677	0.0356	0.6047	0.5455	-0.0058	0.0110	0.04	12.492	6289.576
		Plant species richness	→Herbivore performance	726	32	0.0026	0.0043	-7.5197	7.1573×10^{-14}	-0.3375	-0.1979	0.02	12.492	6289.576
		Plant species richness	→Natural enemy performance	726	32	0.0179	0.0055	3.2545	0.0013	0.0071	0.0287	0.04	12.492	6289.576
	Number of added plant species richness on tri-trophic interactions of plants, herbivores and natural enemies (top-down effects)	Plant species richness	→Natural enemy performance	726	32	0.0223	0.0056	3.9821	9.1541×10^{-5}	0.0113	0.0333	0.00	18.944	6282.388
		Natural enemy performance	→Herbivore performance	726	32	-0.2788	0.0365	-7.6384	2.9495×10^{-14}	-0.3504	-0.2072	0.03	18.944	6282.388
		Herbivore performance	→Plant performance	726	32	-0.2028	0.0328	2.8182	0.0049	0.0038	0.0210	0.02	18.944	6282.388
		Plant species richness	→Herbivore performance	726	32	0.0124	0.0044	-6.1829	7.0109×10^{-10}	-0.2671	-0.1385	0.03	18.944	6282.388
		Plant species richness	→Plant performance	726	32	-0.0132	0.0076	-1.7368	0.0825	-0.0281	0.0017	0.02	18.944	6282.388
Croplands (managed experiment)	Number of added plant species richness on tri-trophic interactions of plants, herbivores and natural enemies (bottom-up effects)	Plant species richness	→Plant performance	1923	74	-0.0095	0.0070	-1.3571	0.1748	-0.0232	0.0042	0.00	12.492	15868.677
		Plant performance	→Herbivore performance	1923	74	-0.1356	0.0206	-6.5825	4.8682×10^{-11}	-0.1760	-0.0952	0.01	12.492	15868.677
		Herbivore performance	→Natural enemy performance	1923	74	-0.2659	0.0224	4.6000	4.2631×10^{-6}	0.0079	0.0197	0.05	12.492	15868.677
		Plant species richness	→Herbivore performance	1923	74	0.0138	0.0030	-11.8705	3.4879×10^{-32}	-0.3098	-0.2220	0.01	12.492	15868.677
		Plant species richness	→Natural enemy performance	1923	74	0.0411	0.0035	11.7429	1.7792×10^{-31}	0.0342	0.0480	0.05	12.492	15868.677
	Number of added plant species richness on tri-trophic interactions of plants, herbivores and natural enemies (top-down effects)	Plant species richness	→Natural enemy performance	1923	74	0.0409	0.0036	11.3611	1.3726×10^{-29}	0.0338	0.0480	0.01	18.944	15826.267
		Natural enemy performance	→Herbivore performance	1923	74	-0.2638	0.0207	-12.7440	7.4150×10^{-37}	-0.3044	-0.2232	0.03	18.944	15826.267
		Herbivore performance	→Plant performance	1923	74	-0.1625	0.0208	7.7667	8.4771×10^{-15}	0.0174	0.0292	0.01	18.944	15826.267
		Plant species richness	→Herbivore performance	1923	74	0.0233	0.0030	-7.8125	6.3890×10^{-15}	-0.2033	-0.1217	0.03	18.944	15826.267
		Plant species richness	→Plant performance	1923	74	-0.0211	0.0071	-2.9718	0.0030	-0.0350	-0.0072	0.01	18.944	15826.267
Croplands (observational experiment)	Number of added plant species richness on tri-trophic interactions of plants, herbivores and natural enemies (bottom-up effects)	Plant species richness	→Plant performance	153	30	0.0248	0.0096	2.5833	0.0100	0.0059	0.0437	0.00	12.492	1257.823
		Plant performance	→Herbivore performance	153	30	-0.3264	0.0692	-4.7168	2.5013×10^{-6}	-0.4621	-0.1907	0.03	12.492	1257.823
		Herbivore performance	→Natural enemy performance	153	30	-0.0585	0.0664	-0.0885	0.9295	-0.0232	0.0212	0.01	12.492	1257.823
		Plant species richness	→Herbivore performance	153	30	-0.0010	0.0113	-0.8810	0.3784	-0.1887	0.0717	0.03	12.492	1257.823
		Plant species richness	→Natural enemy performance	153	30	0.0487	0.0091	5.3516	8.7976×10^{-8}	0.0309	0.0665	0.01	12.492	1257.823
	Number of added plant species richness on tri-trophic interactions of plants, herbivores and natural enemies (top-down effects)	Plant species richness	→Natural enemy performance	153	30	0.0478	0.0090	5.3111	1.0985×10^{-7}	0.0302	0.0654	0.01	18.944	1255.674
		Natural enemy performance	→Herbivore performance	153	30	-0.0458	0.0695	-0.6590	0.5099	-0.1821	0.0905	0.00	18.944	1255.674
		Herbivore performance	→Plant performance	153	30	-0.3729	0.0747	-1.0164	0.3095	-0.0363	0.0115	0.04	18.944	1255.674
		Plant species richness	→Herbivore performance	153	30	-0.0124	0.0122	-4.9920	6.2784×10^{-7}	-0.5194	-0.2264	0.00	18.944	1255.674
		Plant species richness	→Plant performance	153	30	0.0299	0.0090	3.3222	0.0009	0.0122	0.0476	0.04	18.944	1255.674
	Number of added plant species richness on tri-trophic	Plant species richness	→Plant performance	635	34	0.0033	0.0068	0.4853	0.6275	-0.01	0.0166	0.00	14.227	5522.724
		Plant performance	→Herbivore performance	635	34	-0.2696	0.0352	-7.6591	2.2748×10^{-14}	-0.3386	-0.2006	0.03	14.227	5522.724

Organic croplands (temperate regions)	interactions of plants, herbivores and natural enemies (bottom-up effects)	Herbivore performance	→Natural enemy performance	635	34	-0.2477	0.0357	1.1579	0.2478	-0.0031	0.0119	0.05	14.227	5522.724
		Plant species richness	→Herbivore performance	635	34	0.0044	0.0038	-6.9384	4.7908×10^{-12}	-0.3177	-0.1777	0.03	14.227	5522.724
		Plant species richness	→Natural enemy performance	635	34	0.0416	0.0039	10.6667	6.56892×10^{-6}	0.034	0.0492	0.05	14.227	5522.724
	Number of added plant species richness on tri-trophic interactions of plants, herbivores and natural enemies (top-down effects)	Plant species richness	→Natural enemy performance	635	34	0.037	0.004	9.25	5.2993×10^{-20}	0.0292	0.0448	0.02	28.577	5510.791
		Natural enemy performance	→Herbivore performance	635	34	-0.2491	0.0385	-6.4701	1.1164×10^{-10}	-0.3246	-0.1736	0.03	28.577	5510.791
		Herbivore performance	→Plant performance	635	34	-0.3005	0.0351	2.7949	0.0055	0.0032	0.0186	0.04	28.577	5510.791
		Plant species richness	→Herbivore performance	635	34	0.0109	0.0039	-8.5613	1.6329×10^{-17}	-0.3693	-0.2317	0.03	28.577	5510.791
		Plant species richness	→Plant performance	635	34	0.0027	0.0065	0.4154	0.6779	-0.01	0.0154	0.04	28.577	5510.791
Organic croplands (tropical regions)	Number of added plant species richness on tri-trophic interactions of plants, herbivores and natural enemies (bottom-up effects)	Plant species richness	→Plant performance	554	21	0.1162	0.0192	6.0521	1.6601×10^{-9}	0.0785	0.1539	0.02	14.227	4197.695
		Plant performance	→Herbivore performance	554	21	0.0054	0.0316	0.1709	0.8643	-0.0566	0.0674	0.11	14.227	4197.695
		Herbivore performance	→Natural enemy performance	554	21	-0.107	0.0446	-13.1765	3.2073×10^{-34}	-0.3089	-0.2287	0.05	14.227	4197.695
		Plant species richness	→Herbivore performance	554	21	-0.2688	0.0204	-2.3991	0.0165	-0.1944	-0.0196	0.11	14.227	4197.695
		Plant species richness	→Natural enemy performance	554	21	0.1606	0.027	5.9481	6.2898×10^{-9}	0.1075	0.2137	0.05	14.227	4197.695
	Number of added plant species richness on tri-trophic interactions of plants, herbivores and natural enemies (top-down effects)	Plant species richness	→Natural enemy performance	554	21	0.1876	0.0246	7.626	1.5967×10^{-13}	0.1392	0.236	0.04	28.577	4194.027
		Natural enemy performance	→Herbivore performance	554	21	-0.0987	0.032	-3.0844	0.0021	-0.1614	-0.036	0.11	28.577	4194.027
		Herbivore performance	→Plant performance	554	21	-0.017	0.0339	-12.0386	8.7251×10^{-29}	-0.2899	-0.2085	0.02	28.577	4194.027
		Plant species richness	→Herbivore performance	554	21	-0.2492	0.0207	-0.5015	0.6161	-0.0835	0.0495	0.11	28.577	4194.027
		Plant species richness	→Plant performance	554	21	0.1121	0.0212	5.2877	1.3505×10^{-7}	0.0705	0.1537	0.02	28.577	4194.027
Organic croplands (herbaceous plants)	Number of added plant species richness on tri-trophic interactions of plants, herbivores and natural enemies (bottom-up effects)	Plant species richness	→Plant performance	945	36	-0.0122	0.0068	-1.7941	0.0728	-0.0255	0.0011	0.00	14.227	7714.802
		Plant performance	→Herbivore performance	945	36	-0.1184	0.0287	-4.1254	3.7480×10^{-5}	-0.1747	-0.0621	0.01	14.227	7714.802
		Herbivore performance	→Natural enemy performance	945	36	-0.2629	0.0309	-3.3696	0.0008	-0.049	-0.013	0.04	14.227	7714.802
		Plant species richness	→Herbivore performance	945	36	-0.031	0.0092	-8.5081	2.35461×10^{-7}	-0.3235	-0.2023	0.01	14.227	7714.802
		Plant species richness	→Natural enemy performance	945	36	0.0467	0.0067	6.9701	3.3424×10^{-12}	0.0336	0.0598	0.04	14.227	7714.802
	Number of added plant species richness on tri-trophic interactions of plants, herbivores and natural enemies (top-down effects)	Plant species richness	→Natural enemy performance	945	36	0.0488	0.0069	7.0725	1.6248×10^{-12}	0.0353	0.0623	0.01	28.577	7692.721
		Natural enemy performance	→Herbivore performance	945	36	-0.2285	0.0285	-8.0175	1.2695×10^{-15}	-0.2844	-0.1726	0.03	28.577	7692.721
		Herbivore performance	→Plant performance	945	36	-0.1739	0.0265	-2.3297	0.0199	-0.039	-0.0034	0.01	28.577	7692.721
		Plant species richness	→Herbivore performance	945	36	-0.0212	0.0091	-6.5623	5.8715×10^{-11}	-0.2259	-0.1219	0.03	28.577	7692.721
		Plant species richness	→Plant performance	945	36	-0.0136	0.0067	-2.0299	0.0424	-0.0267	-0.0005	0.01	28.577	7692.721
Organic croplands (woody plants)	Number of added plant species richness on tri-trophic interactions of plants, herbivores and natural enemies (bottom-up effects)	Plant species richness	→Plant performance	244	19	-0.0088	0.0101	-0.8713	0.3839	-0.0286	0.011	0.00	14.227	2257.253
		Plant performance	→Herbivore performance	244	19	-0.3425	0.0526	-6.5114	9.2590×10^{-11}	-0.4457	-0.2393	0.04	14.227	2257.253
		Herbivore performance	→Natural enemy performance	244	19	-0.1764	0.0582	1.2041	0.2286	-0.0037	0.0155	0.01	14.227	2257.253
		Plant species richness	→Herbivore performance	244	19	0.0059	0.0049	-3.0309	0.0025	-0.2906	-0.0622	0.04	14.227	2257.253
		Plant species richness	→Natural enemy performance	244	19	0.0059	0.008	0.7375	0.4627	-0.01	0.0218	0.01	14.227	2257.253
	Number of added plant species richness on tri-trophic interactions of plants, herbivores and natural enemies (top-down effects)	Plant species richness	→Natural enemy performance	244	19	0.0072	0.0081	0.8889	0.3765	-0.0089	0.0233	0.00	28.577	2261.84
		Natural enemy performance	→Herbivore performance	244	19	-0.1978	0.0662	-2.9879	0.0029	-0.3277	-0.0679	0.02	28.577	2261.84
		Herbivore performance	→Plant performance	244	19	-0.3433	0.0565	2.5818	0.0099	0.0034	0.025	0.04	28.577	2261.84
		Plant species richness	→Herbivore performance	244	19	0.0142	0.0055	-6.0761	1.4506×10^{-9}	-0.4541	-0.2325	0.02	28.577	2261.84
		Plant species richness	→Plant performance	244	19	-0.0116	0.0095	-1.2211	0.2225	-0.0303	0.0071	0.04	28.577	2261.84

Organic croplands (managed experiment)	Number of added plant species richness on tri-trophic interactions of plants, herbivores and natural enemies (bottom-up effects)	Plant species richness	→Plant performance	1079	39	-0.0181	0.008	-2.2625	0.0237	-0.0338	-0.0024	0.00	14.227	8744.429
		Plant performance	→Herbivore performance	1079	39	-0.1419	0.0259	-5.4788	4.4500×10^{-8}	-0.1927	-0.0911	0.01	14.227	8744.429
		Herbivore performance	→Natural enemy performance	1079	39	-0.2443	0.029	7.4242	1.1830×10^{-13}	0.018	0.031	0.05	14.227	8744.429
		Plant species richness	→Herbivore performance	1079	39	0.0245	0.0033	-8.4241	4.8701×10^{-17}	-0.3012	-0.1874	0.01	14.227	8744.429
		Plant species richness	→Natural enemy performance	1079	39	0.0529	0.0046	11.5	4.4178×10^{-30}	0.0439	0.0619	0.05	14.227	8744.429
	Number of added plant species richness on tri-trophic interactions of plants, herbivores and natural enemies (top-down effects)	Plant species richness	→Natural enemy performance	1079	39	0.0513	0.0047	10.9149	2.8260×10^{-27}	0.0421	0.0605	0.02	28.577	8703.951
		Natural enemy performance	→Herbivore performance	1079	39	-0.2605	0.0269	-9.684	5.4321×10^{-22}	-0.3132	-0.2078	0.03	28.577	8703.951
		Herbivore performance	→Plant performance	1079	39	-0.1656	0.0243	9.9687	2.3581×10^{-23}	0.0256	0.0382	0.02	28.577	8703.951
		Plant species richness	→Herbivore performance	1079	39	0.0319	0.0032	-6.8148	1.0623×10^{-11}	-0.2132	-0.118	0.03	28.577	8703.951
		Plant species richness	→Plant performance	1079	39	-0.0294	0.008	-3.675	0.0002	-0.0451	-0.0137	0.02	28.577	8703.951
Organic croplands (observational experiment)	Number of added plant species richness on tri-trophic interactions of plants, herbivores and natural enemies (bottom-up effects)	Plant species richness	→Plant performance	110	16	0.0248	0.011	2.2545	0.0244	0.0032	0.0464	0.00	14.227	909.883
		Plant performance	→Herbivore performance	110	16	-0.4293	0.0777	-5.5251	3.5886×10^{-8}	-0.5817	-0.2769	0.05	14.227	909.883
		Herbivore performance	→Natural enemy performance	110	16	-0.0971	0.0729	0.7387	0.4602	-0.0136	0.03	0.02	14.227	909.883
		Plant species richness	→Herbivore performance	110	16	0.0082	0.0111	-1.332	0.1830	-0.24	0.0458	0.05	14.227	909.883
		Plant species richness	→Natural enemy performance	110	16	0.0563	0.0096	5.8646	4.6700×10^{-9}	0.0375	0.0751	0.02	14.227	909.883
	Number of added plant species richness on tri-trophic interactions of plants, herbivores and natural enemies (top-down effects)	Plant species richness	→Natural enemy performance	110	16	0.0555	0.0094	5.9043	3.6507×10^{-9}	0.0371	0.0739	0.02	28.577	908.572
		Natural enemy performance	→Herbivore performance	110	16	-0.0817	0.0823	-0.9927	0.3209	-0.2431	0.0797	0.00	28.577	908.572
		Herbivore performance	→Plant performance	110	16	-0.4692	0.0841	-0.2581	0.7964	-0.0275	0.0211	0.05	28.577	908.572
		Plant species richness	→Herbivore performance	110	16	-0.0032	0.0124	-5.5791	2.6300×10^{-8}	-0.6341	-0.3043	0.00	28.577	908.572
		Plant species richness	→Plant performance	110	16	0.0317	0.0099	3.202	0.0014	0.0123	0.0511	0.05	28.577	908.572
Non-organic croplands (temperate regions)	Number of added plant species richness on tri-trophic interactions of plants, herbivores and natural enemies (bottom-up effects)	Plant species richness	→Plant performance	863	48	0.0508	0.0138	3.6812	0.0002	0.0237	0.0779	0.01	0.708	7001.508
		Plant performance	→Herbivore performance	863	48	-0.1258	0.0315	-3.9937	6.6445×10^{-5}	-0.1876	-0.064	0.01	0.708	7001.508
		Herbivore performance	→Natural enemy performance	863	48	-0.2556	0.0345	-2.5055	0.0123	-0.0406	-0.005	0.05	0.708	7001.508
		Plant species richness	→Herbivore performance	863	48	-0.0228	0.0091	-7.4087	1.6901×10^{-13}	-0.3232	-0.188	0.01	0.708	7001.508
		Plant species richness	→Natural enemy performance	863	48	0.0647	0.0096	6.7396	1.9274×10^{-11}	0.0459	0.0835	0.05	0.708	7001.508
	Number of added plant species richness on tri-trophic interactions of plants, herbivores and natural enemies (top-down effects)	Plant species richness	→Natural enemy performance	863	48	0.0728	0.0093	7.828	7.2420×10^{-15}	0.0546	0.091	0.01	0.632	6996.689
		Natural enemy performance	→Herbivore performance	863	48	-0.2332	0.031	-7.5226	6.8202×10^{-14}	-0.294	-0.1724	0.03	0.632	6996.689
		Herbivore performance	→Plant performance	863	48	-0.0918	0.0334	-0.4348	0.6638	-0.022	0.014	0.01	0.632	6996.689
		Plant species richness	→Herbivore performance	863	48	-0.004	0.0092	-2.7485	0.0060	-0.1573	-0.0263	0.03	0.632	6996.689
		Plant species richness	→Plant performance	863	48	0.0396	0.0146	2.7123	0.0068	0.011	0.0682	0.01	0.632	6996.689
Non-organic croplands (tropical regions)	Number of added plant species richness on tri-trophic interactions of plants, herbivores and natural enemies (bottom-up effects)	Plant species richness	→Plant performance	24	1	NA	NA	NA	NA	NA	NA	NA	NA	NA
		Plant performance	→Herbivore performance	24	1	NA	NA	NA	NA	NA	NA	NA	NA	NA
		Herbivore performance	→Natural enemy performance	24	1	NA	NA	NA	NA	NA	NA	NA	NA	NA
		Plant species richness	→Herbivore performance	24	1	NA	NA	NA	NA	NA	NA	NA	NA	NA
		Plant species richness	→Natural enemy performance	24	1	NA	NA	NA	NA	NA	NA	NA	NA	NA
	Number of added plant species richness on tri-trophic interactions of plants,	Plant species richness	→Natural enemy performance	24	1	NA	NA	NA	NA	NA	NA	NA	NA	NA
		Natural enemy performance	→Herbivore performance	24	1	NA	NA	NA	NA	NA	NA	NA	NA	NA
		Herbivore performance	→Plant performance	24	1	NA	NA	NA	NA	NA	NA	NA	NA	NA

	herbivores and natural enemies (top-down effects)	Plant species richness	→Herbivore performance	24	1	NA	NA	NA	NA	NA	NA	NA	NA	NA
		Plant species richness	→Plant performance	24	1	NA	NA	NA	NA	NA	NA	NA	NA	NA
Non-organic croplands (herbaceous plants)	Number of added plant species richness on tri-trophic interactions of plants, herbivores and natural enemies (bottom-up effects)	Plant species richness	→Plant performance	405	36	-0.0824	0.0266	-3.0977	0.0021	-0.1347	-0.0301	0.01	0.708	3206.431
		Plant performance	→Herbivore performance	405	36	-0.1722	0.0434	-3.9677	7.5889×10^{-5}	-0.2573	-0.0871	0.02	0.708	3206.431
		Herbivore performance	→Natural enemy performance	405	36	-0.1147	0.0551	-2.6056	0.0095	-0.0974	-0.0136	0.03	0.708	3206.431
		Plant species richness	→Herbivore performance	405	36	-0.0555	0.0213	-2.0817	0.0376	-0.2228	-0.0066	0.02	0.708	3206.431
		Plant species richness	→Natural enemy performance	405	36	0.1111	0.0292	3.8048	0.0002	0.0538	0.1684	0.03	0.708	3206.431
	Number of added plant species richness on tri-trophic interactions of plants, herbivores and natural enemies (top-down effects)	Plant species richness	→Natural enemy performance	405	36	0.124	0.0287	4.3206	1.7393×10^{-5}	0.0677	0.1803	0.02	0.632	3209.614
		Natural enemy performance	→Herbivore performance	405	36	-0.1111	0.0412	-2.6966	0.0071	-0.1919	-0.0303	0.01	0.632	3209.614
		Herbivore performance	→Plant performance	405	36	-0.204	0.0531	-1.0837	0.2792	-0.0656	0.019	0.03	0.632	3209.614
		Plant species richness	→Herbivore performance	405	36	-0.0233	0.0215	-3.8418	0.0001	-0.3082	-0.0998	0.01	0.632	3209.614
		Plant species richness	→Plant performance	405	36	-0.1024	0.0267	-3.8352	0.0001	-0.1549	-0.0499	0.03	0.632	3209.614
Non-organic croplands (woody plants)	Number of added plant species richness on tri-trophic interactions of plants, herbivores and natural enemies (bottom-up effects)	Plant species richness	→Plant performance	482	13	0.0521	0.0176	2.9602	0.0031	0.0176	0.0866	0.01	0.708	3878.959
		Plant performance	→Herbivore performance	482	13	-0.1106	0.0443	-2.4966	0.0126	-0.1975	-0.0237	0.01	0.708	3878.959
		Herbivore performance	→Natural enemy performance	482	13	-0.3322	0.0437	-1.5327	0.1594	-0.0406	0.0078	0.09	0.708	3878.959
		Plant species richness	→Herbivore performance	482	13	-0.0164	0.0107	-7.6018	2.1264×10^{-11}	-0.419	-0.2454	0.01	0.708	3878.959
		Plant species richness	→Natural enemy performance	482	13	0.0607	0.0102	5.951	0.0002	0.0376	0.0838	0.09	0.708	3878.959
	Number of added plant species richness on tri-trophic interactions of plants, herbivores and natural enemies (top-down effects)	Plant species richness	→Natural enemy performance	482	13	0.0683	0.01	6.83	0.0002	0.0451	0.0915	0.01	0.632	3875.11
		Natural enemy performance	→Herbivore performance	482	13	-0.3297	0.0434	-7.5968	2.1779×10^{-13}	-0.415	-0.2444	0.06	0.632	3875.11
		Herbivore performance	→Plant performance	482	13	-0.0357	0.0355	0.4623	0.6507	-0.0178	0.0276	0.01	0.632	3875.11
		Plant species richness	→Herbivore performance	482	13	0.0049	0.0106	-1.0056	0.3148	-0.1053	0.0339	0.06	0.632	3875.11
		Plant species richness	→Plant performance	482	13	0.0535	0.0197	2.7157	0.0067	0.0149	0.0921	0.01	0.632	3875.11
Non-organic croplands (managed experiment)	Number of added plant species richness on tri-trophic interactions of plants, herbivores and natural enemies (bottom-up effects)	Plant species richness	→Plant performance	844	35	0.0512	0.0138	3.7101	0.0002	0.0241	0.0783	0.01	0.708	6851.731
		Plant performance	→Herbivore performance	844	35	-0.14	0.0315	-4.4444	9.1078×10^{-6}	-0.2018	-0.0782	0.01	0.708	6851.731
		Herbivore performance	→Natural enemy performance	844	35	-0.2717	0.0346	-2.6556	0.0080	-0.0415	-0.0063	0.06	0.708	6851.731
		Plant species richness	→Herbivore performance	844	35	-0.0239	0.009	-7.8526	5.9977×10^{-15}	-0.3395	-0.2039	0.01	0.708	6851.731
		Plant species richness	→Natural enemy performance	844	35	0.0659	0.0095	6.9368	5.0409×10^{-12}	0.0473	0.0845	0.06	0.708	6851.731
	Number of added plant species richness on tri-trophic interactions of plants, herbivores and natural enemies (top-down effects)	Plant species richness	→Natural enemy performance	844	35	0.0746	0.0092	8.1087	8.1713×10^{-16}	0.0566	0.0926	0.01	0.632	6844.395
		Natural enemy performance	→Herbivore performance	844	35	-0.2512	0.031	-8.1032	7.5333×10^{-16}	-0.312	-0.1904	0.04	0.632	6844.395
		Herbivore performance	→Plant performance	844	35	-0.1163	0.0339	-0.3626	0.7169	-0.0211	0.0145	0.01	0.632	6844.395
		Plant species richness	→Herbivore performance	844	35	-0.0033	0.0091	-3.4307	0.0006	-0.1828	-0.0498	0.04	0.632	6844.395
		Plant species richness	→Plant performance	844	35	0.0364	0.0147	2.4762	0.0135	0.0076	0.0652	0.01	0.632	6844.395
Non-organic croplands	Number of added plant species richness on tri-trophic interactions of plants, herbivores and natural enemies (bottom-up effects)	Plant species richness	→Plant performance	43	14	-0.0569	0.1177	-0.4834	0.6293	-0.2889	0.1751	0	0.708	365.317
		Plant performance	→Herbivore performance	43	14	0.3119	0.1518	2.0547	0.0410	0.0129	0.6109	0.03	0.708	365.317
		Herbivore performance	→Natural enemy performance	43	14	0.2161	0.1492	0.7454	0.4568	-0.1405	0.3115	0.04	0.708	365.317
		Plant species richness	→Herbivore performance	43	14	0.0855	0.1147	1.4484	0.1488	-0.0778	0.51	0.03	0.708	365.317
		Plant species richness	→Natural enemy performance	43	14	-0.2287	0.1139	-2.0079	0.0459	-0.4532	-0.0042	0.04	0.708	365.317

(observational experiment)	Number of added plant species richness on tri-trophic interactions of plants, herbivores and natural enemies (top-down effects)	Plant species richness	→Natural enemy performance	43	14	-0.214	0.115	-1.8609	0.0641	-0.4406	0.0126	0.03	0.634	365.223
		Natural enemy performance	→Herbivore performance	43	14	0.2307	0.1593	1.4482	0.1488	-0.083	0.5444	0.02	0.634	365.223
		Herbivore performance	→Plant performance	43	14	0.3062	0.149	0.959	0.3386	-0.1235	0.3577	0.03	0.634	365.223
		Plant species richness	→Herbivore performance	43	14	0.1171	0.1221	2.055	0.0409	0.0127	0.5997	0.02	0.634	365.223
		Plant species richness	→Plant performance	43	14	-0.0777	0.1138	-0.6828	0.4955	-0.302	0.1466	0.03	0.634	365.223

Table S12. Results of the path analyses for the bottom-up and top-down effects of number of added plant species richness on the tri-trophic interactions of plant performance (i.e., plant growth, plant reproduction and plant quality), invertebrate herbivore performance (i.e., herbivore abundance, herbivore damage and herbivore diverisity) and their invertebrate natural enemy performance (i.e., predator abundance, predation, predator diversity, parasitoid abundance, parasitism and parasitoid diversity) in grasslands across different climatic regions, different plant types, and different study types. The predictor and response columns specify the trophic group pairs and the moderator category. The estimate represents the strength of the relationship. The std. err. of estimate denotes the standardized error of the estimate coefficients for the fitted path-analytic models. The number of studies and observations for the predictor-response pair are also presented. Each test is two-sided and the original P value is reported with no multiple comparisons. In addition, test statistic (t value), and 95% confidence interval are reported. R^2 represents the proportion of variance explained for each endogenous variable, the reported R^2 is marginal, which represents variance explained by fixed effects only. Fisher's C statistic assesses the goodness-of-fit of the model through Shipley's test of directed separation. AICc is the adjusted Akaike´s information criterion, the strength of top-down and bottom-up pathways can be assessed using the differences in the AICc values.

Ecosystem type (other item)	Tri-trophic interaction (effect classification)	Predictor	Response	Number of observations	Number of studies	Estimate	Std.Err. of Estimate	t-value	P-value	CI_{lb}	CI_{ub}	R^2	Fisher´s C	AICc
Grasslands (temperate regions)	Number of added plant species richness on tri-trophic interactions of plants, herbivores and natural enemies (bottom-up effects)	Plant species richness	→Plant performance	832	26	0.0017	0.0014	1.2143	0.2303	-0.0011	0.0045	000	19.825	6491.328
		Plant performance	→Herbivore performance	832	26	0.0781	0.0290	2.6931	0.0072	0.0212	0.1350	0.04	19.825	6491.328
		Herbivore performance	→Natural enemy performance	832	26	0.2288	0.0354	5.5000	7.6680×10^{-7}	0.0042	0.0090	0.10	19.825	6491.328
		Plant species richness	→Herbivore performance	832	26	0.0066	0.0012	6.4633	2.0721×10^{-10}	0.1593	0.2983	0.04	19.825	6491.328
		Plant species richness	→Natural enemy performance	832	26	0.0075	0.0013	5.7692	2.3190×10^{-7}	0.0049	0.0101	0.10	19.825	6491.328
	Number of added plant species richness on tri-trophic interactions of plants, herbivores and natural enemies (top-down effects)	Plant species richness	→Natural enemy performance	832	26	0.0090	0.0013	6.9231	3.9788×10^{-9}	0.0064	0.0116	0.05	18.186	6491.277
		Natural enemy performance	→Herbivore performance	832	26	0.1979	0.0300	6.5967	7.6317×10^{-11}	0.1390	0.2568	0.06	18.186	6491.277
		Herbivore performance	→Plant performance	832	26	0.0816	0.0348	4.0833	0.0001	0.0025	0.0073	0.01	18.186	6491.277
		Plant species richness	→Herbivore performance	832	26	0.0049	0.0012	2.3448	0.0193	0.0133	0.1499	0.06	18.186	6491.277
		Plant species richness	→Plant performance	832	26	0.0014	0.0014	1.0000	0.3222	-0.0014	0.0042	0.01	18.186	6491.277
Grasslands (tropical regions)	Number of added plant species richness on tri-trophic interactions of plants, herbivores and natural enemies (bottom-up effects)	Plant species richness	→Plant performance	N/A	N/A	N/A	N/A	N/A	N/A	N/A	N/A	N/A	N/A	N/A
		Plant performance	→Herbivore performance	N/A	N/A	N/A	N/A	N/A	N/A	N/A	N/A	N/A	N/A	N/A
		Herbivore performance	→Natural enemy performance	N/A	N/A	N/A	N/A	N/A	N/A	N/A	N/A	N/A	N/A	N/A
		Plant species richness	→Herbivore performance	N/A	N/A	N/A	N/A	N/A	N/A	N/A	N/A	N/A	N/A	N/A
		Plant species richness	→Natural enemy performance	N/A	N/A	N/A	N/A	N/A	N/A	N/A	N/A	N/A	N/A	N/A
	Number of added plant species richness on tri-trophic interactions of plants, herbivores and natural enemies (top-down effects)	Plant species richness	→Natural enemy performance	N/A	N/A	N/A	N/A	N/A	N/A	N/A	N/A	N/A	N/A	N/A
		Natural enemy performance	→Herbivore performance	N/A	N/A	N/A	N/A	N/A	N/A	N/A	N/A	N/A	N/A	N/A
		Herbivore performance	→Plant performance	N/A	N/A	N/A	N/A	N/A	N/A	N/A	N/A	N/A	N/A	N/A
		Plant species richness	→Herbivore performance	N/A	N/A	N/A	N/A	N/A	N/A	N/A	N/A	N/A	N/A	N/A
		Plant species richness	→Plant performance	N/A	N/A	N/A	N/A	N/A	N/A	N/A	N/A	N/A	N/A	N/A

Grasslands (herbaceous plants)	Number of added plant species richness on tri-trophic interactions of plants, herbivores and natural enemies (bottom-up effects)	Plant species richness	→Plant performance	832	26	0.0017	0.0014	1.2143	0.2303	-0.0011	0.0045	0.00	19.825	6491.328
		Plant performance	→Herbivore performance	832	26	0.0781	0.0290	2.6931	0.0072	0.0212	0.1350	0.04	19.825	6491.328
		Herbivore performance	→Natural enemy performance	832	26	0.2288	0.0354	5.5000	7.6680×10^{-7}	0.0042	0.0090	0.10	19.825	6491.328
		Plant species richness	→Herbivore performance	832	26	0.0066	0.0012	6.4633	2.0721×10^{-10}	0.1593	0.2983	0.04	19.825	6491.328
		Plant species richness	→Natural enemy performance	832	26	0.0075	0.0013	5.7692	2.3190×10^{-7}	0.0049	0.0101	0.10	19.825	6491.328
	Number of added plant species richness on tri-trophic interactions of plants, herbivores and natural enemies (top-down effects)	Plant species richness	→Natural enemy performance	832	26	0.0090	0.0013	6.9231	3.9788×10^{-9}	0.0064	0.0116	0.05	18.186	6491.277
		Natural enemy performance	→Herbivore performance	832	26	0.1979	0.0300	6.5967	7.6317×10^{-11}	0.1390	0.2568	0.06	18.186	6491.277
		Herbivore performance	→Plant performance	832	26	0.0816	0.0348	4.0833	0.0001	0.0025	0.0073	0.01	18.186	6491.277
		Plant species richness	→Herbivore performance	832	26	0.0049	0.0012	2.3448	0.0193	0.0133	0.1499	0.06	18.186	6491.277
		Plant species richness	→Plant performance	832	26	0.0014	0.0014	1.0000	0.3222	-0.0014	0.0042	0.01	18.186	6491.277
Grasslands (woody plants)	Number of added plant species richness on tri-trophic interactions of plants, herbivores and natural enemies (bottom-up effects)	Plant species richness	→Plant performance	N/A	N/A	N/A	N/A	N/A	N/A	N/A	N/A	N/A	N/A	N/A
		Plant performance	→Herbivore performance	N/A	N/A	N/A	N/A	N/A	N/A	N/A	N/A	N/A	N/A	N/A
		Herbivore performance	→Natural enemy performance	N/A	N/A	N/A	N/A	N/A	N/A	N/A	N/A	N/A	N/A	N/A
		Plant species richness	→Herbivore performance	N/A	N/A	N/A	N/A	N/A	N/A	N/A	N/A	N/A	N/A	N/A
		Plant species richness	→Natural enemy performance	N/A	N/A	N/A	N/A	N/A	N/A	N/A	N/A	N/A	N/A	N/A
	Number of added plant species richness on tri-trophic interactions of plants, herbivores and natural enemies (top-down effects)	Plant species richness	→Natural enemy performance	N/A	N/A	N/A	N/A	N/A	N/A	N/A	N/A	N/A	N/A	N/A
		Natural enemy performance	→Herbivore performance	N/A	N/A	N/A	N/A	N/A	N/A	N/A	N/A	N/A	N/A	N/A
		Herbivore performance	→Plant performance	N/A	N/A	N/A	N/A	N/A	N/A	N/A	N/A	N/A	N/A	N/A
		Plant species richness	→Herbivore performance	N/A	N/A	N/A	N/A	N/A	N/A	N/A	N/A	N/A	N/A	N/A
		Plant species richness	→Plant performance	N/A	N/A	N/A	N/A	N/A	N/A	N/A	N/A	N/A	N/A	N/A
Grasslands (managed experiment)	Number of added plant species richness on tri-trophic interactions of plants, herbivores and natural enemies (bottom-up effects)	Plant species richness	→Plant performance	738	13	0.0017	0.0014	1.2143	0.2307	-0.0011	0.0045	0.00	19.825	5758.609
		Plant performance	→Herbivore performance	738	13	0.0611	0.0306	1.9967	0.0462	0.0010	0.1212	0.03	19.825	5758.609
		Herbivore performance	→Natural enemy performance	738	13	0.2240	0.0374	5.6667	2.8260×10^{-7}	0.0044	0.0092	0.10	19.825	5758.609
		Plant species richness	→Herbivore performance	738	13	0.0068	0.0012	5.9893	3.8778×10^{-9}	0.1505	0.2975	0.03	19.825	5758.609
		Plant species richness	→Natural enemy performance	738	13	0.0077	0.0013	5.9231	8.0785×10^{-8}	0.0051	0.0103	0.10	19.825	5758.609
		Plant species richness	→Natural enemy performance	738	13	0.0094	0.0014	6.7143	4.4373×10^{-9}	0.0066	0.0122	0.06	18.186	5756.68

	Number of added plant species richness on tri-trophic interactions of plants, herbivores and natural enemies (top-down effects)	Natural enemy performance	→Herbivore performance	738	13	0.1959	0.0318	6.1604	1.2148×10^{-9}	0.1335	0.2583	0.07	18.186	5756.68
		Herbivore performance	→Plant performance	738	13	0.0651	0.0370	4.1667	7.8354×10^{-5}	0.0026	0.0074	0.01	18.186	5756.68
		Plant species richness	→Herbivore performance	738	13	0.0050	0.0012	1.7595	0.0789	-0.0075	0.1377	0.07	18.186	5756.68
		Plant species richness	→Plant performance	738	13	0.0014	0.0014	1.0000	0.3226	-0.0014	0.0042	0.01	18.186	5756.68
Grasslands (observational experiment)	Number of added plant species richness on tri-trophic interactions of plants, herbivores and natural enemies (bottom-up effects)	Plant species richness	→Plant performance	94	13	0.0452	0.0477	0.9476	0.3464	-0.0498	0.1402	0.01	19.83	788.06
		Plant performance	→Herbivore performance	94	13	0.1759	0.0815	2.1583	0.0339	0.0137	0.3381	0.17	19.83	788.06
		Herbivore performance	→Natural enemy performance	94	13	0.2511	0.1049	4.5567	1.6845×10^{-5}	0.1020	0.2598	0.12	19.83	788.06
		Plant species richness	→Herbivore performance	94	13	0.1809	0.0397	2.3937	0.0290	0.0291	0.4731	0.17	19.83	788.06
		Plant species richness	→Natural enemy performance	94	13	0.0761	0.0483	1.5756	0.1198	-0.0203	0.1725	0.12	19.83	788.06
	Number of added plant species richness on tri-trophic interactions of plants, herbivores and natural enemies (top-down effects)	Plant species richness	→Natural enemy performance	94	13	0.1194	0.0466	2.5622	0.0121	0.0268	0.2120	0.07	18.199	792.176
		Natural enemy performance	→Herbivore performance	94	13	0.1192	0.0893	1.3348	0.1856	-0.0584	0.2968	0.17	18.199	792.176
		Herbivore performance	→Plant performance	94	13	0.2052	0.1092	4.0285	0.0001	0.0859	0.2533	0.05	18.199	792.176
		Plant species richness	→Herbivore performance	94	13	0.1696	0.0421	1.8791	0.0636	-0.0119	0.4223	0.17	18.199	792.176
		Plant species richness	→Plant performance	94	13	0.0124	0.0502	0.2470	0.8056	-0.0878	0.1126	0.05	18.199	792.176

Table S13. Results of the path analyses for the bottom-up and top-down effects of number of added plant species richness on the tri-trophic interactions of plant performance (i.e., plant growth, plant reproduction and plant quality), invertebrate herbivore performance (i.e., herbivore abundance, herbivore damage and herbivore diverisity) and their invertebrate natural enemy performance (i.e., predator abundance, predation, predator diversity, parasitoid abundance, parasitism and parasitoid diversity) in forests across different climatic regions, different plant types, and different study types. The predictor and response columns specify the trophic group pairs and the moderator category. The estimate represents the strength of the relationship. The std. err. of estimate denotes the standardized error of the estimate coefficients for the fitted path-analytic models. The number of studies and observations for the predictor-response pair are also presented. Each test is two-sided and the original P value is reported with no multiple comparisons. In addition, test statistic (t value), and 95% confidence interval are reported. R^2 represents the proportion of variance explained for each endogenous variable, the reported R^2 is marginal, which represents variance explained by fixed effects only. Fisher's C statistic assesses the goodness-of-fit of the model through Shipley's test of directed separation. AICc is the adjusted Akaike´s information criterion, the strength of top-down and bottom-up pathways can be assessed using the differences in the AICc values.

Ecosystem type (other item)	Tri-trophic interaction (effect classification)	Predictor	Response	Number of observations	Number of studies	Estimate	Std.Err. of Estimate	t-value	P-value	CI_{lb}	CI_{ub}	R^2	Fisher´s C	AICc
Forests (temperate regions)	Number of added plant species richness on tri-trophic interactions of plants, herbivores and natural enemies (bottom-up effects)	Plant species richness	→Plant performance	291	16	0.0015	0.0073	0.2055	0.8378	-0.0131	0.0161	0.00	8.64	2338.368
		Plant performance	→Herbivore performance	291	16	0.1123	0.0498	2.2550	0.0249	0.0143	0.2103	0.14	8.64	2338.368
		Herbivore performance	→Natural enemy performance	291	16	0.1454	0.0609	6.5075	1.0262×10^{-9}	0.0304	0.0568	0.05	8.64	2338.368
		Plant species richness	→Herbivore performance	291	16	0.0436	0.0067	2.3875	0.0183	0.0250	0.2658	0.14	8.64	2338.368
		Plant species richness	→Natural enemy performance	291	16	0.0160	0.0075	2.1333	0.0364	0.0010	0.0310	0.05	8.64	2338.368
	Number of added plant species richness on tri-trophic interactions of plants, herbivores and natural enemies (top-down effects)	Plant species richness	→Natural enemy performance	291	16	0.0214	0.0072	2.9722	0.0038	0.0071	0.0357	0.03	6.517	2341.745
		Natural enemy performance	→Herbivore performance	291	16	0.0970	0.0543	1.7864	0.0751	-0.0099	0.2039	0.13	6.517	2341.745
		Herbivore performance	→Plant performance	291	16	0.1211	0.0604	6.0588	8.4785×10^{-9}	0.0278	0.0546	0.01	6.517	2341.745
		Plant species richness	→Herbivore performance	291	16	0.0412	0.0068	2.0050	0.0459	0.0022	0.2400	0.13	6.517	2341.745
		Plant species richness	→Plant performance	291	16	-0.0023	0.0075	-0.3067	0.7601	-0.0173	0.0127	0.01	6.517	2341.745
Forests (tropical regions)	Number of added plant species richness on tri-trophic interactions of plants, herbivores and natural enemies (bottom-up effects)	Plant species richness	→Plant performance	42	3	-0.0079	0.0353	-0.2238	0.8242	-0.0795	0.0637	0.00	8.641	380.142
		Plant performance	→Herbivore performance	42	3	0.2804	0.1066	2.6304	0.0125	0.0642	0.4966	0.10	8.641	380.142
		Herbivore performance	→Natural enemy performance	42	3	0.3235	0.1491	2.2379	0.0312	0.0053	0.1057	0.09	8.641	380.142
		Plant species richness	→Herbivore performance	42	3	0.0555	0.0248	2.1697	0.0363	0.0218	0.6252	0.10	8.641	380.142
		Plant species richness	→Natural enemy performance	42	3	0.0201	0.0262	0.7672	0.4479	-0.0330	0.0732	0.09	8.641	380.142
	Number of added plant species richness on tri-trophic interactions of plants, herbivores and natural enemies (top-down effects)	Plant species richness	→Natural enemy performance	42	3	0.0364	0.0265	1.3736	0.1776	-0.0172	0.0900	0.02	6.519	385.97
		Natural enemy performance	→Herbivore performance	42	3	0.1320	0.1152	1.1458	0.2594	-0.1016	0.3656	0.05	6.519	385.97
		Herbivore performance	→Plant performance	42	3	0.2894	0.1564	1.8609	0.0706	-0.0044	0.1034	0.08	6.519	385.97

		Plant species richness	→Herbivore performance	42	3	0.0495	0.0266	1.8504	0.0721	-0.0273	0.6061	0.05	6.519	385.97
		Plant species richness	→Plant performance	42	3	-0.0205	0.0349	-0.5874	0.5608	-0.0914	0.0504	0.08	6.519	385.97
Forests (herbaceous plants)	Number of added plant species richness on tri-trophic interactions of plants, herbivores and natural enemies (bottom-up effects)	Plant species richness	→Plant performance	N/A	N/A	N/A	N/A	N/A	N/A	N/A	N/A	N/A	N/A	N/A
		Plant performance	→Herbivore performance	N/A	N/A	N/A	N/A	N/A	N/A	N/A	N/A	N/A	N/A	N/A
		Herbivore performance	→Natural enemy performance	N/A	N/A	N/A	N/A	N/A	N/A	N/A	N/A	N/A	N/A	N/A
		Plant species richness	→Herbivore performance	N/A	N/A	N/A	N/A	N/A	N/A	N/A	N/A	N/A	N/A	N/A
		Plant species richness	→Natural enemy performance	N/A	N/A	N/A	N/A	N/A	N/A	N/A	N/A	N/A	N/A	N/A
	Number of added plant species richness on tri-trophic interactions of plants, herbivores and natural enemies (top-down effects)	Plant species richness	→Natural enemy performance	N/A	N/A	N/A	N/A	N/A	N/A	N/A	N/A	N/A	N/A	N/A
		Natural enemy performance	→Herbivore performance	N/A	N/A	N/A	N/A	N/A	N/A	N/A	N/A	N/A	N/A	N/A
		Herbivore performance	→Plant performance	N/A	N/A	N/A	N/A	N/A	N/A	N/A	N/A	N/A	N/A	N/A
		Plant species richness	→Herbivore performance	N/A	N/A	N/A	N/A	N/A	N/A	N/A	N/A	N/A	N/A	N/A
		Plant species richness	→Plant performance	N/A	N/A	N/A	N/A	N/A	N/A	N/A	N/A	N/A	N/A	N/A
Forests (woody plants)	Number of added plant species richness on tri-trophic interactions of plants, herbivores and natural enemies (bottom-up effects)	Plant species richness	→Plant performance	333	19	0.0011	0.0071	0.1549	0.8772	-0.0130	0.0152	0.00	8.64	2658.257
		Plant performance	→Herbivore performance	333	19	0.1342	0.0457	2.9365	0.0036	0.0443	0.2241	0.14	8.64	2658.257
		Herbivore performance	→Natural enemy performance	333	19	0.1273	0.0577	7.0312	3.0353×10^{-11}	0.0324	0.0576	0.03	8.64	2658.257
		Plant species richness	→Herbivore performance	333	19	0.0450	0.0064	2.2062	0.0283	0.0137	0.2409	0.14	8.64	2658.257
		Plant species richness	→Natural enemy performance	333	19	0.0134	0.0073	1.8356	0.0689	-0.0011	0.0279	0.03	8.64	2658.257
	Number of added plant species richness on tri-trophic interactions of plants, herbivores and natural enemies (top-down effects)	Plant species richness	→Natural enemy performance	333	19	0.0185	0.0070	2.6429	0.0091	0.0047	0.0323	0.02	6.517	2661.872
		Natural enemy performance	→Herbivore performance	333	19	0.0877	0.0488	1.7971	0.0733	-0.0083	0.1837	0.12	6.517	2661.872
		Herbivore performance	→Plant performance	333	19	0.1435	0.0562	6.5152	4.9885×10^{-10}	0.0300	0.0560	0.02	6.517	2661.872
		Plant species richness	→Herbivore performance	333	19	0.0430	0.0066	2.5534	0.0111	0.0329	0.2541	0.12	6.517	2661.872
		Plant species richness	→Plant performance	333	19	-0.0034	0.0073	-0.4658	0.6426	-0.0179	0.0111	0.02	6.517	2661.872
Forests (managed experiment)	Number of added plant species richness on tri-trophic interactions of plants, herbivores and natural enemies (bottom-up effects)	Plant species richness	→Plant performance	220	11	0.0066	0.0089	0.7416	0.4631	-0.0114	0.0246	0.00	8.64	1727.27
		Plant performance	→Herbivore performance	220	11	0.0796	0.0524	1.5191	0.1303	-0.0237	0.1829	0.11	8.64	1727.27
		Herbivore performance	→Natural enemy performance	220	11	0.1676	0.0707	5.6892	5.6874×10^{-8}	0.0275	0.0567	0.05	8.64	1727.27
		Plant species richness	→Herbivore performance	220	11	0.0421	0.0074	2.3706	0.0219	0.0253	0.3099	0.11	8.64	1727.27

		Plant species richness	→Natural enemy performance	220	11	0.0120	0.0088	1.3636	0.1783	-0.0056	0.0296	0.05	8.64	1727.27
	Number of added plant species richness on tri-trophic interactions of plants, herbivores and natural enemies (top-down effects)	Plant species richness	→Natural enemy performance	220	11	0.0196	0.0083	2.3614	0.0194	0.0032	0.0360	0.02	6.517	1729.721
		Natural enemy performance	→Herbivore performance	220	11	0.1145	0.0604	1.8957	0.0594	-0.0046	0.2336	0.12	6.517	1729.721
		Herbivore performance	→Plant performance	220	11	0.0887	0.0713	5.4267	2.0120×10^{-7}	0.0259	0.0555	0.01	6.517	1729.721
		Plant species richness	→Herbivore performance	220	11	0.0407	0.0075	1.2440	0.2149	-0.0519	0.2293	0.12	6.517	1729.721
		Plant species richness	→Plant performance	220	11	0.0029	0.0094	0.3085	0.7599	-0.0163	0.0221	0.01	6.517	1729.721
Forests (observational experiment)	Number of added plant species richness on tri-trophic interactions of plants, herbivores and natural enemies (bottom-up effects)	Plant species richness	→Plant performance	113	8	-0.0091	0.0122	-0.7459	0.4964	-0.0427	0.0245	0.00	8.64	978.461
		Plant performance	→Herbivore performance	113	8	0.2426	0.0841	2.8847	0.0048	0.0758	0.4094	0.15	8.64	978.461
		Herbivore performance	→Natural enemy performance	113	8	0.0847	0.0908	3.6822	0.0006	0.0216	0.0734	0.02	8.64	978.461
		Plant species richness	→Herbivore performance	113	8	0.0475	0.0129	0.9328	0.3535	-0.0958	0.2652	0.15	8.64	978.461
		Plant species richness	→Natural enemy performance	113	8	0.0162	0.0118	1.3729	0.2405	-0.0163	0.0487	0.02	8.64	978.461
	Number of added plant species richness on tri-trophic interactions of plants, herbivores and natural enemies (top-down effects)	Plant species richness	→Natural enemy performance	113	8	0.0182	0.0116	1.5690	0.1814	-0.0122	0.0486	0.02	6.517	981.157
		Natural enemy performance	→Herbivore performance	113	8	0.0487	0.0882	0.5522	0.5820	-0.1262	0.2236	0.10	6.517	981.157
		Herbivore performance	→Plant performance	113	8	0.2261	0.0931	3.2463	0.0022	0.0165	0.0705	0.05	6.517	981.157
		Plant species richness	→Herbivore performance	113	8	0.0435	0.0134	2.4286	0.0168	0.0415	0.4107	0.10	6.517	981.157
		Plant species richness	→Plant performance	113	8	-0.0121	0.0120	-1.0083	0.3706	-0.0455	0.0213	0.05	6.517	981.157

Table S14. Results of the path analyses for the bottom-up and top-down effects of binary plant species richness on the tri-trophic interactions of plant performance (i.e., plant growth, plant reproduction and plant quality), invertebrate herbivore performance (i.e., herbivore abundance, herbivore damage and herbivore diveirsity) and their invertebrate natural enemy performance (i.e., predator abundance, predation, predator diversity, parasitoid abundance, parasitism and parasitoid diversity) across different climatic region types (i.e., temperate zone and tropical zone), plant types (i.e., herbaceous plant and woody plant), and study types (managed experiment and observational experiment). The predictor and response columns specify the trophic group pairs and the moderator category. The estimate represents the strength of the relationship. The std. err. of estimate denotes the standardized error of the estimate coefficients for the fitted path-analytic models. The number of studies and observations for the predictor-response pair are also presented. Each test is two-sided and the original P value is reported with no multiple comparisons. In addition, test statistic (t value) and 95% confidence interval are reported. R^2 represents the proportion of variance explained for each endogenous variable, the reported R^2 is marginal, which represents variance explained by fixed effects only. Fisher's C statistic assesses the goodness-of-fit of the model through Shipley's test of directed separation. AICc is the adjusted Akaike´s information criterion, the strength of top-down and bottom-up pathways can be assessed using the differences in the AICc values.

Ecosystem type (other item)	Tri-trophic interaction (effect classification)	Predictor	Response	Number of observations	Number of studies	Estimate	Std.Err. of Estimate	t-value	P-value	CI_{lb}	CI_{ub}	R^2	Fisher´s C	AICc
Temperate zone	Binary plant species richness on tri-trophic interactions of plants, herbivores and natural enemies (bottom-up effects)	Plant species richness	→Plant performance	2621	124	0.3915	0.0360	10.8750	7.4928×10^{-27}	0.3209	0.4621	0.04	22.499	20148.613
		Plant performance	→Herbivore performance	2621	124	-0.0247	0.0187	-1.3209	0.1867	-0.0614	0.0120	0.07	22.499	20148.613
		Herbivore performance	→Natural enemy performance	2621	124	0.0037	0.0192	-13.6610	1.4410×10^{-37}	-0.5531	-0.4141	0.12	22.499	20148.613
		Plant species richness	→Herbivore performance	2621	124	-0.4836	0.0354	0.1927	0.8472	-0.0340	0.0414	0.07	22.499	20148.613
		Plant species richness	→Natural enemy performance	2621	124	0.6720	0.0360	18.6667	3.66016×10^{-3}	0.6013	0.7427	0.12	22.499	20148.613
	Binary plant species richness on tri-trophic interactions of plants, herbivores and natural enemies (top-down effects)	Plant species richness	→Natural enemy performance	2621	124	0.6736	0.0346	19.4682	1.2137×10^{-70}	0.6057	0.7415	0.13	23.911	20134.238
		Natural enemy performance	→Herbivore performance	2621	124	-0.0026	0.0196	-0.1327	0.8945	-0.0410	0.0358	0.07	23.911	20134.238
		Herbivore performance	→Plant performance	2621	124	-0.0301	0.0194	-13.2642	6.4786×10^{-36}	-0.5649	-0.4193	0.04	23.911	20134.238
		Plant species richness	→Herbivore performance	2621	124	-0.4921	0.0371	-1.5515	0.1209	-0.0681	0.0079	0.07	23.911	20134.238
		Plant species richness	→Plant performance	2621	124	0.3755	0.0375	10.0133	4.68382×10^{-23}	0.3020	0.4490	0.04	23.911	20134.238
Tropical zone	Binary plant species richness on tri-trophic interactions of plants, herbivores and natural enemies (bottom-up effects)	Plant species richness	→Plant performance	620	25	0.3896	0.0561	6.9447	1.0130×10^{-11}	0.2794	0.4998	0.04	22.499	4296.296
		Plant performance	→Herbivore performance	620	25	0.0046	0.0312	0.1474	0.8828	-0.0567	0.0659	0.21	22.499	4296.296
		Herbivore performance	→Natural enemy performance	620	25	-0.0349	0.0406	-14.2256	6.1130×10^{-39}	-0.9617	-0.7283	0.18	22.499	4296.296
		Plant species richness	→Herbivore performance	620	25	-0.8450	0.0594	-0.8596	0.3904	-0.1146	0.0448	0.21	22.499	4296.296
		Plant species richness	→Natural enemy performance	620	25	0.7869	0.0764	10.2997	6.5825×10^{-23}	0.6368	0.9370	0.18	22.499	4296.296
	Binary plant species richness on tri-trophic interactions of plants, herbivores and natural enemies (top-down effects)	Plant species richness	→Natural enemy performance	620	25	0.8200	0.0671	12.2206	8.6803×10^{-31}	0.6882	0.9518	0.18	23.911	4291.815
		Natural enemy performance	→Herbivore performance	620	25	-0.0536	0.0341	-1.5718	0.1165	-0.1206	0.0134	0.21	23.911	4291.815
		Herbivore performance	→Plant performance	620	25	0.0202	0.0330	-12.4417	5.1539×10^{-31}	-0.9263	-0.6737	0.05	23.911	4291.815
		Plant species richness	→Herbivore performance	620	25	-0.8000	0.0643	0.6121	0.5407	-0.0446	0.0850	0.21	23.911	4291.815

		Plant species richness	→Plant performance	620	25	0.4138	0.0635	6.5165	1.5947×10^{-10}	0.2891	0.5385	0.05	23.911	4291.815
Herbaceous plant	Binary plant species richness on tri-trophic interactions of plants, herbivores and natural enemies (bottom-up effects)	Plant species richness	→Plant performance	2182	98	0.3766	0.0379	9.9367	9.8007×10^{-23}	0.3023	0.4509	0.04	22.499	16709.583
		Plant performance	→Herbivore performance	2182	98	-0.0434	0.0205	-2.1171	0.0344	-0.0836	-0.0032	0.05	22.499	16709.583
		Herbivore performance	→Natural enemy performance	2182	98	0.0048	0.0213	-10.8000	1.1697×10^{-24}	-0.4914	-0.3402	0.09	22.499	16709.583
		Plant species richness	→Herbivore performance	2182	98	-0.4158	0.0385	0.2254	0.8217	-0.0370	0.0466	0.05	22.499	16709.583
		Plant species richness	→Natural enemy performance	2182	98	0.5811	0.0392	14.8240	9.2005×10^{-43}	0.5041	0.6581	0.09	22.499	16709.583
	Binary plant species richness on tri-trophic interactions of plants, herbivores and natural enemies (top-down effects)	Plant species richness	→Natural enemy performance	2182	98	0.5806	0.0379	15.3193	1.6326×10^{-46}	0.5062	0.6550	0.09	23.911	16706.07
		Natural enemy performance	→Herbivore performance	2182	98	0.0052	0.0211	0.2464	0.8054	-0.0362	0.0466	0.05	23.911	16706.07
		Herbivore performance	→Plant performance	2182	98	-0.0505	0.0204	-10.9874	1.8810×10^{-25}	-0.5129	-0.3573	0.04	23.911	16706.07
		Plant species richness	→Herbivore performance	2182	98	-0.4351	0.0396	-2.4755	0.0134	-0.0905	-0.0105	0.05	23.911	16706.07
		Plant species richness	→Plant performance	2182	98	0.3540	0.0390	9.0769	2.7778×10^{-19}	0.2775	0.4305	0.04	23.911	16706.07
Woody plant	Binary plant species richness on tri-trophic interactions of plants, herbivores and natural enemies (bottom-up effects)	Plant species richness	→Plant performance	1059	51	0.4001	0.0550	7.2745	7.0581×10^{-13}	0.2922	0.5080	0.04	22.499	7940.356
		Plant performance	→Herbivore performance	1059	51	0.0360	0.0274	1.3139	0.1892	-0.0178	0.0898	0.18	22.499	7940.356
		Herbivore performance	→Natural enemy performance	1059	51	-0.0049	0.0301	-15.6404	2.7196×10^{-43}	-0.9402	-0.7302	0.23	22.499	7940.356
		Plant species richness	→Herbivore performance	1059	51	-0.8352	0.0534	-0.1628	0.8707	-0.0640	0.0542	0.18	22.499	7940.356
		Plant species richness	→Natural enemy performance	1059	51	0.9532	0.0587	16.2385	2.3846×10^{-44}	0.8377	1.0687	0.23	22.499	7940.356
	Binary plant species richness on tri-trophic interactions of plants, herbivores and natural enemies (top-down effects)	Plant species richness	→Natural enemy performance	1059	51	0.9627	0.0524	18.3721	7.4749×10^{-61}	0.8598	1.0656	0.24	23.911	7924.862
		Natural enemy performance	→Herbivore performance	1059	51	-0.0375	0.0309	-1.2136	0.2252	-0.0981	0.0231	0.18	23.911	7924.862
		Herbivore performance	→Plant performance	1059	51	0.0611	0.0313	-13.1970	3.4278×10^{-33}	-0.9083	-0.6727	0.05	23.911	7924.862
		Plant species richness	→Herbivore performance	1059	51	-0.7905	0.0599	1.9521	0.0512	-0.0003	0.1225	0.18	23.911	7924.862
		Plant species richness	→Plant performance	1059	51	0.4591	0.0623	7.3692	3.72741×10^{-3}	0.3368	0.5814	0.05	23.911	7924.862
Managed experiment	Binary plant species richness on tri-trophic interactions of plants, herbivores and natural enemies (bottom-up effects)	Plant species richness	→Plant performance	2881	98	0.3741	0.0328	11.4055	1.9177×10^{-29}	0.3098	0.4384	0.03	22.499	21820.191
		Plant performance	→Herbivore performance	2881	98	-0.0227	0.0173	-1.3121	0.1896	-0.0566	0.0112	0.11	22.499	21820.191
		Herbivore performance	→Natural enemy performance	2881	98	-0.0126	0.0185	-18.9480	5.9964×10^{-69}	-0.6838	-0.5554	0.15	22.499	21820.191
		Plant species richness	→Herbivore performance	2881	98	-0.6196	0.0327	-0.6811	0.4959	-0.0489	0.0237	0.11	22.499	21820.191
		Plant species richness	→Natural enemy performance	2881	98	0.7233	0.0344	21.0262	6.0623×10^{-81}	0.6558	0.7908	0.15	22.499	21820.191
		Plant species richness	→Natural enemy performance	2881	98	0.7344	0.0322	22.8075	3.5154×10^{-98}	0.6712	0.7976	0.15	23.911	21808.572

	Binary plant species richness on tri-trophic interactions of plants, herbivores and natural enemies (top-down effects)	Natural enemy performance	→Herbivore performance	2881	98	-0.0209	0.0185	-1.1297	0.2587	-0.0572	0.0154	0.11	23.911	21808.572
		Herbivore performance	→Plant performance	2881	98	-0.0313	0.0183	-17.6351	3.4954×10^{-61}	-0.6820	-0.5454	0.04	23.911	21808.572
		Plant species richness	→Herbivore performance	2881	98	-0.6137	0.0348	-1.7104	0.0873	-0.0672	0.0046	0.11	23.911	21808.572
		Plant species richness	→Plant performance	2881	98	0.3530	0.0352	10.0284	3.1498×10^{-23}	0.2840	0.4220	0.04	23.911	21808.572
Observational experiment	Binary plant species richness on tri-trophic interactions of plants, herbivores and natural enemies (bottom-up effects)	Plant species richness	→Plant performance	360	51	0.4948	0.1054	4.6945	4.8433×10^{-6}	0.2870	0.7026	0.06	22.499	2811.721
		Plant performance	→Herbivore performance	360	51	0.0391	0.0507	0.7712	0.4412	-0.0606	0.1388	0.0	22.499	2811.721
		Herbivore performance	→Natural enemy performance	360	51	0.0736	0.0501	0.5743	0.5662	-0.1528	0.2788	0.03	22.499	2811.721
		Plant species richness	→Herbivore performance	360	51	0.0630	0.1097	1.4691	0.1428	-0.0250	0.1722	0.00	22.499	2811.721
		Plant species richness	→Natural enemy performance	360	51	0.3418	0.1045	3.2708	0.0012	0.1362	0.5474	0.03	22.499	2811.721
	Binary plant species richness on tri-trophic interactions of plants, herbivores and natural enemies (top-down effects)	Plant species richness	→Natural enemy performance	360	51	0.3559	0.1040	3.4221	0.0007	0.1513	0.5605	0.03	23.911	2809.649
		Natural enemy performance	→Herbivore performance	360	51	0.0774	0.0498	1.5542	0.1211	-0.0206	0.1754	0.01	23.911	2809.649
		Herbivore performance	→Plant performance	360	51	0.0432	0.0514	0.5102	0.6102	-0.1565	0.2661	0.06	23.911	2809.649
		Plant species richness	→Herbivore performance	360	51	0.0548	0.1074	0.8405	0.4013	-0.0579	0.1443	0.01	23.911	2809.649
		Plant species richness	→Plant performance	360	51	0.5084	0.1058	4.8053	2.9779×10^{-6}	0.2998	0.7170	0.06	23.911	2809.649

Table S15. Results of the path analyses for the bottom-up and top-down effects of number of added plant species on the tri-trophic interactions of plant performance (i.e., plant growth, plant reproduction and plant quality), invertebrate herbivore performance (i.e., herbivore abundance, herbivore damage and herbivore diverisity) and their invertebrate natural enemy performance (i.e., predator abundance, predation, predator diversity, parasitoid abundance, parasitism and parasitoid diversity) across different climatic region types (i.e., temperate zone and tropical zone), plant types (i.e., herbaceous plant and woody plant), and study types (managed experiment and observational experiment). The predictor and response columns specify the trophic group pairs and the moderator category. The estimate represents the strength of the relationship. The std. err. of estimate denotes the standardized error of the estimate coefficients for the fitted path-analytic models. The number of studies and observations for the predictor-response pair are also presented. Each test is two-sided and the original P value is reported with no multiple comparisons. In addition, test statistic (t value) and 95% confidence interval are reported. R^2 represents the proportion of variance explained for each endogenous variable, the reported R^2 is marginal, which represents variance explained by fixed effects only. Fisher's C statistic assesses the goodness-of-fit of the model through Shipley's test of directed separation. AICc is the adjusted Akaike´s information criterion, the strength of top-down and bottom-up pathways can be assessed using the differences in the AICc values.

Items	Tri-trophic interaction (effect classification)	Predictor	Response	Number of observations	Number of studies	Estimate	Std.Err. of Estimate	t-value	P-value	CI_{lb}	CI_{ub}	R^2	Fisher´s C	AICc
Temperate zone	Number of added plant species on tri-trophic interactions of plants, herbivores and natural enemies (bottom-up effects)	Plant species richness	→Plant performance	2621	124	0.0033	0.0013	2.5385	0.0128	0.0007	0.0059	0.00	60.496	20689.447
		Plant performance	→Herbivore performance	2621	124	-0.0763	0.0189	-4.0370	5.5708×10^{-5}	-0.1134	-0.0392	0.01	60.496	20689.447
		Herbivore performance	→Natural enemy performance	2621	124	-0.1078	0.0195	5.0000	2.2139×10^{-6}	0.0036	0.0084	0.04	60.496	20689.447
		Plant species richness	→Herbivore performance	2621	124	0.0060	0.0012	-5.5282	3.5595×10^{-8}	-0.1460	-0.0696	0.01	60.496	20689.447
		Plant species richness	→Natural enemy performance	2621	124	0.0106	0.0012	8.8333	4.0760×10^{-14}	0.0082	0.0130	0.04	60.496	20689.447
	Number of added plant species on tri-trophic interactions of plants, herbivores and natural enemies (top-down effects)	Plant species richness	→Natural enemy performance	2621	124	0.0101	0.0013	7.7692	8.3740×10^{-12}	0.0075	0.0127	0.02	64.305	20672.887
		Natural enemy performance	→Herbivore performance	2621	124	-0.1102	0.0191	-5.7696	8.8916×10^{-9}	-0.1477	-0.0727	0.02	64.305	20672.887
		Herbivore performance	→Plant performance	2621	124	-0.0876	0.0190	5.8333	5.7157×10^{-8}	0.0046	0.0094	0.01	64.305	20672.887
		Plant species richness	→Herbivore performance	2621	124	0.0070	0.0012	-4.6105	4.2129×10^{-6}	-0.1249	-0.0503	0.02	64.305	20672.887
		Plant species richness	→Plant performance	2621	124	0.0037	0.0013	2.8462	0.0054	0.0011	0.0063	0.01	64.305	20672.887
Tropical zone	Number of added plant species on tri-trophic interactions of plants, herbivores and natural enemies (bottom-up effects)	Plant species richness	→Plant performance	620	25	0.0628	0.0153	4.1046	4.6275×10^{-5}	0.0328	0.0928	0.02	60.496	4511.807
		Plant performance	→Herbivore performance	620	25	-0.0538	0.0337	-1.5964	0.1109	-0.1200	0.0124	0.09	60.496	4511.807
		Herbivore performance	→Natural enemy performance	620	25	-0.1676	0.0393	-8.1458	3.8373×10^{-10}	-0.1464	-0.0882	0.1	60.496	4511.807
		Plant species richness	→Herbivore performance	620	25	-0.1173	0.0144	-4.2646	2.3443×10^{-5}	-0.2448	-0.0904	0.09	60.496	4511.807
		Plant species richness	→Natural enemy performance	620	25	0.0994	0.0171	5.8129	2.7456×10^{-7}	0.0652	0.1336	0.1	60.496	4511.807
	Number of added plant species on tri-trophic interactions of plants, herbivores and natural enemies (top-down effects)	Plant species richness	→Natural enemy performance	620	25	0.1203	0.0166	7.2470	4.9224×10^{-10}	0.0872	0.1534	0.08	64.305	4505.324
		Natural enemy performance	→Herbivore performance	620	25	-0.1736	0.0343	-5.0612	5.7838×10^{-7}	-0.2410	-0.1062	0.11	64.305	4505.324
		Herbivore performance	→Plant performance	620	25	-0.0440	0.0318	-6.9379	5.0846×10^{-8}	-0.1301	-0.0711	0.02	64.305	4505.324

		Plant species richness	→Herbivore performance	620	25	-0.1006	0.0145	-1.3836	0.1670	-0.1065	0.0185	0.11	64.305	4505.324
		Plant species richness	→Plant performance	620	25	0.0555	0.0163	3.4049	0.0007	0.0235	0.0875	0.02	64.305	4505.324
Herbaceous plant	Number of added plant species on tri-trophic interactions of plants, herbivores and natural enemies (bottom-up effects)	Plant species richness	→Plant performance	2182	98	0.0006	0.0013	0.4615	0.6447	-0.0020	0.0032	000	60.496	17076.568
		Plant performance	→Herbivore performance	2182	98	-0.0894	0.0206	-4.3398	1.4922×10^{-5}	-0.1298	-0.0490	0.01	60.496	17076.568
		Herbivore performance	→Natural enemy performance	2182	98	-0.0867	0.0214	3.5000	0.0007	0.0018	0.0066	0.03	60.496	17076.568
		Plant species richness	→Herbivore performance	2182	98	0.0042	0.0012	-4.0514	5.2734×10^{-5}	-0.1287	-0.0447	0.01	60.496	17076.568
		Plant species richness	→Natural enemy performance	2182	98	0.0102	0.0013	7.8462	7.7361×10^{-12}	0.0076	0.0128	0.03	60.496	17076.568
	Number of added plant species on tri-trophic interactions of plants, herbivores and natural enemies (top-down effects)	Plant species richness	→Natural enemy performance	2182	98	0.0097	0.0013	7.4615	5.6337×10^{-11}	0.0071	0.0123	0.03	64.305	17075.131
		Natural enemy performance	→Herbivore performance	2182	98	-0.0780	0.0208	-3.7500	0.0002	-0.1188	-0.0372	0.01	64.305	17075.131
		Herbivore performance	→Plant performance	2182	98	-0.0965	0.0202	3.6923	0.0004	0.0022	0.0074	0.01	64.305	17075.131
		Plant species richness	→Herbivore performance	2182	98	0.0048	0.0013	-4.7772	1.8978×10^{-6}	-0.1361	-0.0569	0.01	64.305	17075.131
		Plant species richness	→Plant performance	2182	98	0.0010	0.0013	0.7692	0.4422	-0.0016	0.0036	0.01	64.305	17075.131
Woody plant	Number of added plant species on tri-trophic interactions of plants, herbivores and natural enemies (bottom-up effects)	Plant species richness	→Plant performance	1059	51	0.0038	0.0051	0.7451	0.4571	-0.0063	0.0139	0.00	60.496	8397.522
		Plant performance	→Herbivore performance	1059	51	-0.0457	0.0295	-1.5492	0.1217	-0.1036	0.0122	0.02	60.496	8397.522
		Herbivore performance	→Natural enemy performance	1059	51	-0.2411	0.0297	4.2115	5.2768×10^{-5}	0.0116	0.0322	0.10	60.496	8397.522
		Plant species richness	→Herbivore performance	1059	51	0.0219	0.0052	-8.1178	1.3473×10^{-15}	-0.2994	-0.1828	0.02	60.496	8397.522
		Plant species richness	→Natural enemy performance	1059	51	0.0365	0.0053	6.8868	1.13581×10^{-0}	0.0260	0.0470	0.1	60.496	8397.522
	Number of added plant species on tri-trophic interactions of plants, herbivores and natural enemies (top-down effects)	Plant species richness	→Natural enemy performance	1059	51	0.0320	0.0053	6.0377	1.1837×10^{-8}	0.0215	0.0425	0.04	64.305	8375.054
		Natural enemy performance	→Herbivore performance	1059	51	-0.2657	0.0292	-9.0993	4.5692×10^{-19}	-0.3230	-0.2084	0.09	64.305	8375.054
		Herbivore performance	→Plant performance	1059	51	-0.0468	0.0284	5.9412	3.2692×10^{-8}	0.0202	0.0404	0.00	64.305	8375.054
		Plant species richness	→Herbivore performance	1059	51	0.0303	0.0051	-1.6479	0.0997	-0.1025	0.0089	0.09	64.305	8375.054
		Plant species richness	→Plant performance	1059	51	0.0041	0.0052	0.7885	0.4314	-0.0062	0.0144	0.00	64.305	8375.054
	Number of added plant species on tri-trophic interactions of plants, herbivores and natural enemies (bottom-up effects)	Plant species richness	→Plant performance	2881	98	0.0030	0.0012	2.5000	0.0141	0.0006	0.0054	0.00	60.496	22609.336
		Plant performance	→Herbivore performance	2881	98	-0.0870	0.0180	-4.8333	1.4139×10^{-6}	-0.1223	-0.0517	0.01	60.496	22609.336
		Herbivore performance	→Natural enemy performance	2881	98	-0.1606	0.0184	4.0833	8.2268×10^{-5}	0.0025	0.0073	0.05	60.496	22609.336
		Plant species richness	→Herbivore performance	2881	98	0.0049	0.0012	-8.7283	4.3362×10^{-18}	-0.1967	-0.1245	0.01	60.496	22609.336

Managed experiment		Plant species richness	→Natural enemy performance	2881	98	0.0116	0.0013	8.9231	3.9860×10^{-15}	0.0090	0.0142	0.05	60.496	22609.336
	Number of added plant species on tri-trophic interactions of plants, herbivores and natural enemies (top-down effects)	Plant species richness	→Natural enemy performance	2881	98	0.0107	0.0013	8.2308	2.1011×10^{-13}	0.0081	0.0133	0.02	64.305	22593.373
		Natural enemy performance	→Herbivore performance	2881	98	-0.1610	0.0181	-8.8950	1.0176×10^{-18}	-0.1965	-0.1255	0.03	64.305	22593.373
		Herbivore performance	→Plant performance	2881	98	-0.0981	0.0174	5.5000	2.3641×10^{-7}	0.0042	0.0090	0.01	64.305	22593.373
		Plant species richness	→Herbivore performance	2881	98	0.0066	0.0012	-5.6379	1.8910×10^{-8}	-0.1322	-0.0640	0.03	64.305	22593.373
		Plant species richness	→Plant performance	2881	98	0.0033	0.0012	2.7500	0.0071	0.0009	0.0057	0.01	64.305	22593.373
Observational experiment	Number of added plant species on tri-trophic interactions of plants, herbivores and natural enemies (bottom-up effects)	Plant species richness	→Plant performance	360	51	0.0034	0.0087	0.3908	0.6996	-0.0146	0.0214	0.00	60.496	2837.219
		Plant performance	→Herbivore performance	360	51	0.0423	0.0481	0.8794	0.3798	-0.0523	0.1369	0.04	60.496	2837.219
		Herbivore performance	→Natural enemy performance	360	51	0.0546	0.0509	3.6413	0.0007	0.0150	0.0520	0.03	60.496	2837.219
		Plant species richness	→Herbivore performance	360	51	0.0335	0.0092	1.0727	0.2842	-0.0455	0.1547	0.04	60.496	2837.219
		Plant species richness	→Natural enemy performance	360	51	0.0263	0.0090	2.9222	0.0066	0.0079	0.0447	0.03	60.496	2837.219
	Number of added plant species on tri-trophic interactions of plants, herbivores and natural enemies (top-down effects)	Plant species richness	→Natural enemy performance	360	51	0.0280	0.0089	3.1461	0.0037	0.0098	0.0462	0.03	64.305	2836.987
		Natural enemy performance	→Herbivore performance	360	51	0.0555	0.0488	1.1373	0.2562	-0.0405	0.1515	0.04	64.305	2836.987
		Herbivore performance	→Plant performance	360	51	0.0452	0.0532	3.4516	0.0013	0.0133	0.0509	0.00	64.305	2836.987
		Plant species richness	→Herbivore performance	360	51	0.0321	0.0093	0.8496	0.3961	-0.0594	0.1498	0.04	64.305	2836.987
		Plant species richness	→Plant performance	360	51	0.0029	0.0087	0.3333	0.7420	-0.0151	0.0209	0.00	64.305	2836.987

Table S16. Results of the path analyses for the bottom-up and top-down effects of binary plant species richness on the tri-trophic interactions of plant performance (i.e., plant growth, plant reproduction and plant quality), invertebrate herbivore performance (i.e., herbivore abundance, herbivore damage and herbivore diverisity) and their invertebrate predator performance (i.e., predator abundance, predation and predator diversity) in global terrestrial ecosystems, organic and non-organic croplands, grasslands and forests (as presented also in fig. S8). The predictor and response columns specify the trophic group pairs and the moderator category. The estimate represents the strength of the relationship. The std. err. of estimate denotes the standardized error of the estimate coefficients for the fitted path-analytic models. The number of studies and observations for the predictor-response pair are also presented. Each test is two-sided and the original P value is reported with no multiple comparisons. In addition, test statistic (t value), and 95% confidence interval are reported. R^2 represents the proportion of variance explained for each endogenous variable, the reported R^2 is marginal, which represents variance explained by fixed effects only. Fisher's C statistic assesses the goodness-of-fit of the model through Shipley's test of directed separation. AICc is the adjusted Akaike´s information criterion, the strength of top-down and bottom-up pathways can be assessed using the differences in the AICc values.

Ecosystem type	Tri-trophic interaction (effect classification)	Predictor	Response	Number of observations	Number of studies	Estimate	Std.Err. of Estimate	t-value	P-value	CI_{lb}	CI_{ub}	R^2	Fisher´s C	AICc
Global terrestrial ecosystems	Binary plant species richness on tri-trophic interactions of plants, herbivores and predators (bottom-up effects)	Plant species richness	→Plant performance	1997	135	0.4224	0.0393	10.7481	3.5001×10^{-26}	0.3453	0.4995	0.05	3.163	14797.103
		Plant performance	→Herbivore performance	1997	135	0.0316	0.0215	1.4698	0.1418	-0.0106	0.0738	0.07	3.163	14797.103
		Herbivore performance	→Predator performance	1997	135	0.0288	0.0198	-12.3109	6.6992×10^{-32}	-0.5738	-0.4160	0.10	3.163	14797.103
		Plant species richness	→Herbivore performance	1997	135	-0.4949	0.0402	1.4545	0.1460	-0.0100	0.0676	0.07	3.163	14797.103
		Plant species richness	→Predator performance	1997	135	0.7248	0.0370	19.5892	7.9831×10^{-78}	0.6522	0.7974	0.10	3.163	14797.103
	Binary plant species richness on tri-trophic interactions of plants, herbivores and predators (top-down effects)	Plant species richness	→Predator performance	1997	135	0.7142	0.0351	20.3476	2.9620×10^{-83}	0.6454	0.7830	0.09	3.715	14779.18
		Predator performance	→Herbivore performance	1997	135	0.0644	0.0234	2.7521	0.0060	0.0185	0.1103	0.07	3.715	14779.18
		Herbivore performance	→Plant performance	1997	135	0.0347	0.0215	-12.3958	1.2750×10^{-32}	-0.6131	-0.4455	0.05	3.715	14779.18
		Plant species richness	→Herbivore performance	1997	135	-0.5293	0.0427	1.6140	0.1067	-0.0075	0.0769	0.07	3.715	14779.18
		Plant species richness	→Plant performance	1997	135	0.4433	0.0410	10.8122	1.8963×10^{-26}	0.3629	0.5237	0.05	3.715	14779.18
Organic croplands	Binary plant species richness on tri-trophic interactions of plants, herbivores and predators (bottom-up effects)	Plant species richness	→Plant performance	596	47	0.3627	0.0545	6.6550	6.7653×10^{-11}	0.2557	0.4697	0.03	3.163	3874.783
		Plant performance	→Herbivore performance	596	47	-0.0383	0.0338	-1.1331	0.2577	-0.1047	0.0281	0.26	3.163	3874.783
		Herbivore performance	→Predator performance	596	47	-0.0809	0.0344	-15.2322	4.4511×10^{-44}	-1.0369	-0.8001	0.14	3.163	3874.783
		Plant species richness	→Herbivore performance	596	47	-0.9185	0.0603	-2.3517	0.0190	-0.1485	-0.0133	0.26	3.163	3874.783
		Plant species richness	→Predator performance	596	47	0.8164	0.0612	13.3399	2.6450×10^{-35}	0.6962	0.9366	0.14	3.163	3874.783
	Binary plant species richness on tri-trophic interactions of plants, herbivores and predators (top-down effects)	Plant species richness	→Predator performance	596	47	0.8958	0.0519	17.2601	1.3875×10^{-53}	0.7939	0.9977	0.14	3.715	3870.038
		Predator performance	→Herbivore performance	596	47	-0.0598	0.0397	-1.5063	0.1346	-0.1384	0.0188	0.26	3.715	3870.038
		Herbivore performance	→Plant performance	596	47	-0.0508	0.0360	-12.6946	4.2911×10^{-32}	-1.0130	-0.7414	0.03	3.715	3870.038
		Plant species richness	→Herbivore performance	596	47	-0.8772	0.0691	-1.4111	0.1588	-0.1215	0.0199	0.26	3.715	3870.038
		Plant species richness	→Plant performance	596	47	0.3179	0.0640	4.9672	9.0481×10^{-7}	0.1922	0.4436	0.03	3.715	3870.038
	Binary plant species richness on tri-trophic interactions of plants, herbivores and predators (bottom-up effects)	Plant species richness	→Plant performance	621	48	0.2229	0.0670	3.3269	9.32694×10^{-4}	0.0913	0.3545	0.01	3.163	4282.339
		Plant performance	→Herbivore performance	621	48	-0.0525	0.0328	-1.6006	0.1100	-0.1169	0.0119	0.33	3.163	4282.339
		Herbivore performance	→Predator performance	621	48	-0.0036	0.0377	-17.7791	6.6223×10^{-57}	-1.1530	-0.9236	0.16	3.163	4282.339
		Plant species richness	→Herbivore performance	621	48	-1.0383	0.0584	-0.0955	0.9240	-0.0776	0.0704	0.33	3.163	4282.339

		Plant species richness	→Predator performance	621	48	0.8247	0.0670	12.3090	5.1230×10^{-31}	0.6931	0.9563	0.16	3.163	4282.339
Non-organic croplands	Binary plant species richness on tri-trophic interactions of plants, herbivores and predators (top-down effects)	Plant species richness	→Predator performance	621	48	0.8383	0.0533	15.7280	1.5033×10^{-46}	0.7336	0.9430	0.17	3.715	4266.962
		Predator performance	→Herbivore performance	621	48	0.0153	0.0402	0.3806	0.7037	-0.0637	0.0943	0.33	3.715	4266.962
		Herbivore performance	→Plant performance	621	48	-0.0985	0.0462	-16.0284	2.54054×10^{-8}	-1.2019	-0.9395	0.02	3.715	4266.962
		Plant species richness	→Herbivore performance	621	48	-1.0707	0.0668	-2.1320	0.0334	-0.1892	-0.0078	0.33	3.715	4266.962
		Plant species richness	→Plant performance	621	48	0.1212	0.0831	1.4585	0.1452	-0.0420	0.2844	0.02	3.715	4266.962
Grasslands	Binary plant species richness on tri-trophic interactions of plants, herbivores and predators (bottom-up effects)	Plant species richness	→Plant performance	578	24	0.6003	0.0870	6.9000	1.4091×10^{-11}	0.4294	0.7712	0.08	3.124	4407.153
		Plant performance	→Herbivore performance	578	24	0.0787	0.0362	2.1740	0.0301	0.0076	0.1498	0.03	3.124	4407.153
		Herbivore performance	→Predator performance	578	24	0.2323	0.0393	3.6625	0.0003	0.1343	0.4451	0.07	3.124	4407.153
		Plant species richness	→Herbivore performance	578	24	0.2897	0.0791	5.9109	6.2413×10^{-9}	0.1551	0.3095	0.03	3.124	4407.153
		Plant species richness	→Predator performance	578	24	0.2189	0.0789	2.7744	0.0057	0.0639	0.3739	0.07	3.124	4407.153
	Binary plant species richness on tri-trophic interactions of plants, herbivores and predators (top-down effects)	Plant species richness	→Predator performance	578	24	0.3040	0.0794	3.8287	0.0001	0.1480	0.4600	0.02	3.728	4383.29
		Predator performance	→Herbivore performance	578	24	0.2727	0.0349	7.8138	4.5347×10^{-13}	0.2038	0.3416	0.1	3.728	4383.29
		Herbivore performance	→Plant performance	578	24	0.0727	0.0405	3.5423	0.0004	0.1155	0.4031	0.08	3.728	4383.29
		Plant species richness	→Herbivore performance	578	24	0.2593	0.0732	1.7951	0.0732	-0.0068	0.1522	0.1	3.728	4383.29
		Plant species richness	→Plant performance	578	24	0.5747	0.0880	6.5307	1.4724×10^{-10}	0.4018	0.7476	0.08	3.728	4383.29
Forests	Binary plant species richness on tri-trophic interactions of plants, herbivores and predators (bottom-up effects)	Plant species richness	→Plant performance	202	16	1.0466	0.1318	7.9408	2.3313×10^{-13}	0.7865	1.3067	0.24	3.124	1566.462
		Plant performance	→Herbivore performance	202	16	0.0521	0.0752	0.6928	0.4893	-0.0962	0.2004	0.04	3.124	1566.462
		Herbivore performance	→Predator performance	202	16	0.1314	0.0653	2.3309	0.0208	0.0589	0.7075	0.1	3.124	1566.462
		Plant species richness	→Herbivore performance	202	16	0.3832	0.1644	2.0123	0.0456	0.0026	0.2602	0.04	3.124	1566.462
		Plant species richness	→Predator performance	202	16	0.5710	0.1336	4.2740	3.0437×10^{-5}	0.3075	0.8345	0.1	3.124	1566.462
	Binary plant species richness on tri-trophic interactions of plants, herbivores and predators (top-down effects)	Plant species richness	→Predator performance	202	16	0.6303	0.1304	4.8336	2.7761×10^{-6}	0.3731	0.8875	0.08	3.728	1562.247
		Predator performance	→Herbivore performance	202	16	0.2081	0.0719	2.8943	0.0044	0.0659	0.3503	0.09	3.728	1562.247
		Herbivore performance	→Plant performance	202	16	0.0427	0.0630	2.0995	0.0371	0.0188	0.6018	0.24	3.728	1562.247
		Plant species richness	→Herbivore performance	202	16	0.3103	0.1478	0.6778	0.4987	-0.0816	0.1670	0.09	3.728	1562.247
		Plant species richness	→Plant performance	202	16	1.0281	0.1348	7.6269	1.5729×10^{-12}	0.7620	1.2942	0.24	3.728	1562.247

Table S17. Results of the path analyses for the bottom-up and top-down effects of binary plant species richness on the tri-trophic interactions of plant performance (i.e., plant growth, plant reproduction and plant quality), invertebrate herbivore performance (i.e., herbivore abundance, herbivore damage and herbivore diverisity) and their invertebrate parasitoid performance (i.e., parasitoid abundance, parasitism and parasitoid diversity) in global terrestrial ecosystems, organic and non-organic croplands, grasslands and forests (as presented also in fig. S9). The predictor and response columns specify the trophic group pairs and the moderator category. The estimate represents the strength of the relationship. The std. err. of estimate denotes the standardized error of the estimate coefficients for the fitted path-analytic models. The number of studies and observations for the predictor-response pair are also presented. Each test is two-sided and the original P value is reported with no multiple comparisons. In addition, test statistic (t value), and 95% confidence interval are reported. R^2 represents the proportion of variance explained for each endogenous variable, the reported R^2 is marginal, which represents variance explained by fixed effects only. Fisher's C statistic assesses the goodness-of-fit of the model through Shipley's test of directed separation. AICc is the adjusted Akaike´s information criterion, the strength of top-down and bottom-up pathways can be assessed using the differences in the AICc values.

Ecosystem type	Tri-trophic interaction (effect classification)	Predictor	Response	Number of observations	Number of studies	Estimate	Std.Err. of Estimate	t-value	P-value	CI_{lb}	CI_{ub}	R^2	Fisher´s C	AICc
Global terrestrial ecosystems	Binary plant species richness on tri-trophic interactions of plants, herbivores and parasitoids (bottom-up effects)	Plant species richness	→Plant performance	1244	49	0.3411	0.0516	6.6105	5.8535×10^{-11}	0.2399	0.4423	0.03	31.832	9316.965
		Plant performance	→Herbivore performance	1244	49	-0.0906	0.0257	-3.5253	0.0004	-0.1410	-0.0402	0.13	31.832	9316.965
		Herbivore performance	→Parasitoid performance	1244	49	-0.1293	0.0251	-13.0040	1.0207×10^{-33}	-0.7454	-0.5498	0.07	31.832	9316.965
		Plant species richness	→Herbivore performance	1244	49	-0.6476	0.0498	-5.1514	3.0182×10^{-7}	-0.1785	-0.0801	0.13	31.832	9316.965
		Plant species richness	→Parasitoid performance	1244	49	0.6393	0.0469	13.6311	2.0915×10^{-39}	0.5473	0.7313	0.07	31.832	9316.965
	Binary plant species richness on tri-trophic interactions of plants, herbivores and parasitoids (top-down effects)	Plant species richness	→Parasitoid performance	1244	49	0.7376	0.0433	17.0346	2.5064×10^{-58}	0.6526	0.8226	0.06	16.841	9318.718
		Parasitoid performance	→Herbivore performance	1244	49	-0.1317	0.0276	-4.7717	2.3314×10^{-6}	-0.1859	-0.0775	0.14	16.841	9318.718
		Herbivore performance	→Plant performance	1244	49	-0.1046	0.0279	-11.2989	1.0378×10^{-25}	-0.6924	-0.4872	0.04	16.841	9318.718
		Plant species richness	→Herbivore performance	1244	49	-0.5898	0.0522	-3.7491	0.0002	-0.1593	-0.0499	0.14	16.841	9318.718
		Plant species richness	→Plant performance	1244	49	0.2640	0.0553	4.7740	2.0511×10^{-6}	0.1555	0.3725	0.04	16.841	9318.718
Organic croplands	Binary plant species richness on tri-trophic interactions of plants, herbivores and parasitoids (bottom-up effects)	Plant species richness	→Plant performance	593	23	0.3879	0.0669	5.7982	1.1607×10^{-8}	0.2565	0.5193	0.03	31.832	4142.847
		Plant performance	→Herbivore performance	593	23	-0.1863	0.0342	-5.4474	7.6326×10^{-8}	-0.2535	-0.1191	0.22	31.832	4142.847
		Herbivore performance	→Parasitoid performance	593	23	-0.1283	0.0311	-12.5832	3.5634×10^{-32}	-0.9529	-0.6955	0.09	31.832	4142.847
		Plant species richness	→Herbivore performance	593	23	-0.8242	0.0655	-4.1254	4.2605×10^{-5}	-0.1894	-0.0672	0.22	31.832	4142.847
		Plant species richness	→Parasitoid performance	593	23	0.7199	0.0589	12.2224	1.2719×10^{-30}	0.6042	0.8356	0.09	31.832	4142.847
	Binary plant species richness on tri-trophic interactions of plants, herbivores and parasitoids (top-down effects)	Plant species richness	→Parasitoid performance	593	23	0.8404	0.0519	16.1927	1.1514×10^{-48}	0.7385	0.9423	0.09	16.841	4143.295
		Parasitoid performance	→Herbivore performance	593	23	-0.1420	0.0421	-3.3729	9.6745×10^{-4}	-0.2253	-0.0587	0.21	16.841	4143.295
		Herbivore performance	→Plant performance	593	23	-0.2277	0.0391	-10.5807	1.6671×10^{-23}	-0.9246	-0.6350	0.06	16.841	4143.295
		Plant species richness	→Herbivore performance	593	23	-0.7798	0.0737	-5.8235	9.6728×10^{-9}	-0.3045	-0.1509	0.21	16.841	4143.295
		Plant species richness	→Plant performance	593	23	0.1776	0.0745	2.3839	0.0175	0.0312	0.3240	0.06	16.841	4143.295
	Binary plant species richness	Plant species richness	→Plant performance	266	11	0.0797	0.0935	0.8524	0.3948	-0.1044	0.2638	0.00	31.832	1849.908
		Plant performance	→Herbivore performance	266	11	-0.1422	0.0462	-3.0779	2.3220×10^{-3}	-0.2332	-0.0512	0.43	31.832	1849.908

Non-organic croplands	on tri-trophic interactions of plants, herbivores and parasitoids (bottom-up effects)	Herbivore performance	→Parasitoid performance	266	11	-0.3232	0.0610	-14.5478	3.4754×10^{-35}	-1.3990	-1.0654	0.20	31.832	1849.908
		Plant species richness	→Herbivore performance	266	11	-1.2322	0.0847	-5.2984	2.6383×10^{-7}	-0.4434	-0.2030	0.43	31.832	1849.908
		Plant species richness	→Parasitoid performance	266	11	0.6705	0.1131	5.9284	9.9901×10^{-9}	0.4478	0.8932	0.20	31.832	1849.908
	Binary plant species richness on tri-trophic interactions of plants, herbivores and parasitoids (top-down effects)	Plant species richness	→Parasitoid performance	266	11	1.0724	0.0879	12.2002	3.75182×10^{-7}	0.8993	1.2455	0.15	16.841	1829.707
		Parasitoid performance	→Herbivore performance	266	11	-0.3137	0.0494	-6.3502	1.0956×10^{-9}	-0.4110	-0.2164	0.49	16.841	1829.707
		Herbivore performance	→Plant performance	266	11	-0.2857	0.0662	-9.4490	2.1167×10^{-18}	-1.0961	-0.7181	0.05	16.841	1829.707
		Plant species richness	→Herbivore performance	266	11	-0.9071	0.0960	-4.3157	2.4435×10^{-5}	-0.4162	-0.1552	0.49	16.841	1829.707
		Plant species richness	→Plant performance	266	11	-0.2756	0.1223	-2.2535	0.0251	-0.5165	-0.0347	0.05	16.841	1829.707
Grasslands	Binary plant species richness on tri-trophic interactions of plants, herbivores and parasitoids (bottom-up effects)	Plant species richness	→Plant performance	254	10	0.4204	0.1284	3.2741	0.0012	0.1675	0.6733	0.04	31.843	2017.589
		Plant performance	→Herbivore performance	254	10	0.0072	0.0551	0.1307	0.8962	-0.1014	0.1158	0.00	31.843	2017.589
		Herbivore performance	→Parasitoid performance	254	10	0.2026	0.0647	0.8341	0.4051	-0.1308	0.3228	0.04	31.843	2017.589
		Plant species richness	→Herbivore performance	254	10	0.0960	0.1151	3.1314	0.0025	0.0736	0.3316	0.00	31.843	2017.589
		Plant species richness	→Parasitoid performance	254	10	0.1416	0.1151	1.2302	0.2199	-0.0852	0.3684	0.04	31.843	2017.589
	Binary plant species richness on tri-trophic interactions of plants, herbivores and parasitoids (top-down effects)	Plant species richness	→Parasitoid performance	254	10	0.1691	0.1162	1.4552	0.1470	-0.0599	0.3981	0.01	17.132	2024.224
		Parasitoid performance	→Herbivore performance	254	10	0.0758	0.0568	1.3345	0.1848	-0.0368	0.1884	0.01	17.132	2024.224
		Herbivore performance	→Plant performance	254	10	0.0033	0.0620	0.7675	0.4435	-0.1355	0.3085	0.04	17.132	2024.224
		Plant species richness	→Herbivore performance	254	10	0.0865	0.1127	0.0532	0.9576	-0.1188	0.1254	0.01	17.132	2024.224
		Plant species richness	→Plant performance	254	10	0.4199	0.1289	3.2576	0.0013	0.1660	0.6738	0.04	17.132	2024.224
Forests	Binary plant species richness on tri-trophic interactions of plants, herbivores and parasitoids (bottom-up effects)	Plant species richness	→Plant performance	131	5	0.8247	0.2369	3.4812	0.0007	0.3558	1.2936	0.09	31.843	1029.274
		Plant performance	→Herbivore performance	131	5	0.1493	0.0737	2.0258	0.0450	0.0034	0.2952	0.02	31.843	1029.274
		Herbivore performance	→Parasitoid performance	131	5	0.0526	0.0771	-0.7477	0.4561	-0.5664	0.2558	0.00	31.843	1029.274
		Plant species richness	→Herbivore performance	131	5	-0.1553	0.2077	0.6822	0.4964	-0.1001	0.2053	0.02	31.843	1029.274
		Plant species richness	→Parasitoid performance	131	5	0.2188	0.1802	1.2142	0.2270	-0.1380	0.5756	0.00	31.843	1029.274
	Binary plant species richness on tri-trophic interactions of plants, herbivores and parasitoids (top-down effects)	Plant species richness	→Parasitoid performance	131	5	0.2201	0.1801	1.2221	0.2240	-0.1364	0.5766	0.00	17.132	1030.904
		Parasitoid performance	→Herbivore performance	131	5	0.0250	0.0817	0.3060	0.7602	-0.1368	0.1868	0.00	17.132	1030.904
		Herbivore performance	→Plant performance	131	5	0.1300	0.0837	-0.1885	0.8508	-0.4394	0.3630	0.10	17.132	1030.904
		Plant species richness	→Herbivore performance	131	5	-0.0382	0.2027	1.5532	0.1230	-0.0357	0.2957	0.00	17.132	1030.904
		Plant species richness	→Plant performance	131	5	0.8207	0.2357	3.4820	0.0007	0.3542	1.2872	0.10	17.132	1030.904

Table S 18. Results of the path analyses for the bottom-up and top-down effects of number of added plant species richness on the tri-trophic interactions of plant performance (i.e., plant growth, plant reproduction and plant quality), invertebrate herbivore performance (i.e., herbivore abundance, herbivore damage and herbivore diveristy) and their invertebrate predator performance (i.e., predator abundance, predation and predator diversity) in global terrestrial ecosystems, organic and non-organic croplands, grasslands and forests. The predictor and response columns specify the trophic group pairs and the moderator category. The estimate represents the strength of the relationship. The std. err. of estimate denotes the standardized error of the estimate coefficients for the fitted path-analytic models. The number of studies and observations for the predictor-response pair are also presented. Each test is two-sided and the original P value is reported with no multiple comparisons. In addition, test statistic (t value), and 95% confidence interval are reported. R^2 represents the proportion of variance explained for each endogenous variable, the reported R^2 is marginal, which represents variance explained by fixed effects only. Fisher's C statistic assesses the goodness-of-fit of the model through Shipley's test of directed separation. AICc is the adjusted Akaike´s information criterion, the strength of top-down and bottom-up pathways can be assessed using the differences in the AICc values.

Ecosystem type	Tri-trophic interaction (effect classification)	Predictor	Response	Number of observations	Number of studies	Estimate	Std.Err. of Estimate	t-value	P-value	CI_{lb}	CI_{ub}	R^2	Fisher´s C	AICc
Global terrestrial ecosystems	Number of added plant species richness on tri-trophic interactions of plants, herbivores and predators (bottom-up effects)	Number of added plant species richness	→Plant performance	1997	135	0.0049	0.0016	3.0625	0.0027	0.0017	0.0081	0.00	26.939	15349.657
		Plant performance	→Herbivore performance	1997	135	-0.0293	0.0217	-1.3502	0.1771	-0.0719	0.0133	0.01	26.939	15349.657
		Herbivore performance	→Predator performance	1997	135	-0.0942	0.0205	3.8750	0.0002	0.0030	0.0094	0.02	26.939	15349.657
		Number of added plant species richness	→Herbivore performance	1997	135	0.0062	0.0016	-4.5951	4.6094×10^{-6}	-0.1344	-0.0540	0.01	26.939	15349.657
		Number of added plant species richness	→Predator performance	1997	135	0.0123	0.0017	7.2353	6.6979×10^{-13}	0.0090	0.0156	0.02	26.939	15349.657
	Number of added plant species richness on tri-trophic interactions of plants, herbivores and predators (top-down effects)	Number of added plant species richness	→Predator performance	1997	135	0.0119	0.0017	7.0000	3.5189×10^{-12}	0.0086	0.0152	0.02	26.966	15356.907
		Predator performance	→Herbivore performance	1997	135	-0.0640	0.0226	-2.8319	0.0047	-0.1083	-0.0197	0.01	26.966	15356.907
		Herbivore performance	→Plant performance	1997	135	-0.0343	0.0212	4.2500	3.4380×10^{-5}	0.0036	0.0100	0.01	26.966	15356.907
		Number of added plant species richness	→Herbivore performance	1997	135	0.0068	0.0016	-1.6179	0.1058	-0.0759	0.0073	0.01	26.966	15356.907
		Number of added plant species richness	→Plant performance	1997	135	0.0051	0.0016	3.1875	0.0018	0.0019	0.0083	0.01	26.966	15356.907
Organic croplands	Number of added plant species richness on tri-trophic interactions of plants, herbivores and predators (bottom-up effects)	Number of added plant species richness	→Plant performance	596	47	-0.0041	0.0077	-0.5325	0.5946	-0.0192	0.0110	0.0	26.939	4252.404
		Plant performance	→Herbivore performance	596	47	-0.1455	0.0389	-3.7404	0.0002	-0.2219	-0.0691	0.04	26.939	4252.404
		Herbivore performance	→Predator performance	596	47	-0.2928	0.0340	-3.2785	0.0014	-0.0416	-0.0102	0.08	26.939	4252.404
		Number of added plant species richness	→Herbivore performance	596	47	-0.0259	0.0079	-8.6118	7.6968×10^{-17}	-0.3596	-0.2260	0.04	26.939	4252.404
		Number of added plant species richness	→Predator performance	596	47	0.0332	0.0082	4.0488	5.9265×10^{-5}	0.0171	0.0493	0.08	26.939	4252.404
	Number of added plant species richness on tri-trophic interactions of plants, herbivores and predators (top-down effects)	Number of added plant species richness	→Predator performance	596	47	0.0481	0.0084	5.7262	1.7706×10^{-8}	0.0316	0.0646	0.04	26.966	4250.408
		Predator performance	→Herbivore performance	596	47	-0.3070	0.0391	-7.8517	1.4354×10^{-13}	-0.3840	-0.2300	0.10	26.966	4250.408
		Herbivore performance	→Plant performance	596	47	-0.1548	0.0319	-1.6076	0.1104	-0.0283	0.0029	0.02	26.966	4250.408
		Number of added plant species richness	→Herbivore performance	596	47	-0.0127	0.0079	-4.8527	1.5902×10^{-6}	-0.2175	-0.0921	0.10	26.966	4250.408
		Number of added plant species richness	→Plant performance	596	47	-0.0123	0.0077	-1.5974	0.1108	-0.0274	0.0028	0.02	26.966	4250.408

Non-organic croplands	Number of added plant species richness on tri-trophic interactions of plants, herbivores and predators (bottom-up effects)	Number of added plant species richness →Plant performance	621	48	0.0208	0.0191	1.0890	0.2772	-0.0168	0.0584	0.00	26.939	4575.083
		Plant performance →Herbivore performance	621	48	-0.1247	0.0390	-3.1974	0.0015	-0.2013	-0.0481	0.07	26.939	4575.083
		Herbivore performance →Predator performance	621	48	-0.1913	0.0330	-5.8541	1.2090×10^{-7}	-0.1452	-0.0714	0.14	26.939	4575.083
		Number of added plant species richness →Herbivore performance	621	48	-0.1083	0.0185	-5.7970	1.1178×10^{-8}	-0.2561	-0.1265	0.07	26.939	4575.083
		Number of added plant species richness →Predator performance	621	48	0.1762	0.0189	9.3228	2.5776×10^{-19}	0.1391	0.2133	0.14	26.939	4575.083
	Number of added plant species richness on tri-trophic interactions of plants, herbivores and predators (top-down effects)	Number of added plant species richness →Predator performance	621	48	0.2034	0.0174	11.6897	1.7031×10^{-28}	0.1692	0.2376	0.14	26.966	4562.125
		Predator performance →Herbivore performance	621	48	-0.2505	0.0435	-5.7586	1.9891×10^{-8}	-0.3361	-0.1649	0.09	26.966	4562.125
		Herbivore performance →Plant performance	621	48	-0.1372	0.0388	-3.8021	0.0004	-0.1115	-0.0345	0.02	26.966	4562.125
		Number of added plant species richness →Herbivore performance	621	48	-0.0730	0.0192	-3.5361	4.3761×10^{-4}	-0.2134	-0.0610	0.09	26.966	4562.125
		Number of added plant species richness →Plant performance	621	48	0.0019	0.0207	0.0918	0.9270	-0.0389	0.0427	0.02	26.966	4562.125
Grasslands	Number of added plant species richness on tri-trophic interactions of plants, herbivores and predators (bottom-up effects)	Number of added plant species richness →Plant performance	578	24	0.0042	0.0018	2.3333	0.0223	0.0006	0.0078	0.01	26.777	4456.263
		Plant performance →Herbivore performance	578	24	0.0995	0.0347	2.8674	0.0043	0.0313	0.1677	0.04	26.777	4456.263
		Herbivore performance →Predator performance	578	24	0.2275	0.0390	4.6875	1.0411×10^{-5}	0.0043	0.0107	0.08	26.777	4456.263
		Number of added plant species richness →Herbivore performance	578	24	0.0075	0.0016	5.8333	9.6432×10^{-9}	0.1509	0.3041	0.04	26.777	4456.263
		Number of added plant species richness →Predator performance	578	24	0.0067	0.0018	3.7222	0.0002	0.0032	0.0102	0.08	26.777	4456.263
	Number of added plant species richness on tri-trophic interactions of plants, herbivores and predators (top-down effects)	Number of added plant species richness →Predator performance	578	24	0.0085	0.0018	4.7222	2.9632×10^{-6}	0.0050	0.0120	0.03	27.048	4435.51
		Predator performance →Herbivore performance	578	24	0.2654	0.0352	7.5398	2.4383×10^{-12}	0.1959	0.3349	0.10	27.048	4435.51
		Herbivore performance →Plant performance	578	24	0.1052	0.0416	3.6250	0.0005	0.0026	0.0090	0.02	27.048	4435.51
		Number of added plant species richness →Herbivore performance	578	24	0.0058	0.0016	2.5288	0.0117	0.0235	0.1869	0.10	27.048	4435.51
		Number of added plant species richness →Plant performance	578	24	0.0036	0.0018	2.0000	0.0493	0.0000	0.0072	0.02	27.048	4435.51
Forests	Number of added plant species richness on tri-trophic interactions of plants, herbivores and predators (bottom-up effects)	Number of added plant species richness →Plant performance	202	16	0.0014	0.0083	0.1687	0.8673	-0.0156	0.0184	0.00	26.777	1633.332
		Plant performance →Herbivore performance	202	16	0.1358	0.0624	2.1763	0.0308	0.0127	0.2589	0.13	26.777	1633.332
		Herbivore performance →Predator performance	202	16	0.1443	0.0697	4.8072	1.2976×10^{-5}	0.0233	0.0565	0.07	26.777	1633.332
		Number of added plant species richness →Herbivore performance	202	16	0.0399	0.0083	2.0703	0.0398	0.0068	0.2818	0.13	26.777	1633.332
		Number of added plant species richness →Predator performance	202	16	0.0227	0.0094	2.4149	0.0171	0.0041	0.0413	0.07	26.777	1633.332
	Number of added plant species richness on tri-trophic interactions of plants, herbivores and predators (top-down effects)	Number of added plant species richness →Predator performance	202	16	0.0289	0.0090	3.2111	0.0016	0.0111	0.0467	0.05	27.048	1632.279
		Predator performance →Herbivore performance	202	16	0.1636	0.0698	2.3438	0.0202	0.0259	0.3013	0.13	27.048	1632.279
		Herbivore performance →Plant performance	202	16	0.1453	0.0723	4.1529	0.0001	0.0183	0.0523	0.02	27.048	1632.279
		Number of added plant species richness →Herbivore performance	202	16	0.0353	0.0085	2.0097	0.0458	0.0027	0.2879	0.13	27.048	1632.279
		Number of added plant species richness →Plant performance	202	16	-0.0027	0.0085	-0.3176	0.7534	-0.0202	0.0148	0.02	27.048	1632.279

Table S19. Results of the path analyses for the bottom-up and top-down effects of number of added plant species richness on the tri-trophic interactions of plant performance (i.e., plant growth, plant reproduction and plant quality), invertebrate herbivore performance (i.e., herbivore abundance, herbivore damage and herbivore diveirsity) and their invertebrate parasitoid performance (i.e., parasitoid abundance, parasitism and parasitoid diversity) in global terrestrial ecosystems, organic and non-organic croplands, grasslands and forests. The predictor and response columns specify the trophic group pairs and the moderator category. The estimate represents the strength of the relationship. The std. err. of estimate denotes the standardized error of the estimate coefficients for the fitted path-analytic models. The number of studies and observations for the predictor-response pair are also presented. Each test is two-sided and the original P value is reported with no multiple comparisons. In addition, test statistic (t value), and 95% confidence interval are reported. R^2 represents the proportion of variance explained for each endogenous variable, the reported R^2 is marginal, which represents variance explained by fixed effects only. Fisher's C statistic assesses the goodness-of-fit of the model through Shipley's test of directed separation. AICc is the adjusted Akaike´s information criterion, the strength of top-down and bottom-up pathways can be assessed using the differences in the AICc values.

Ecosystem type	Tri-trophic interaction (effect classification)	Predictor	Response	Number of observations	Number of studies	Estimate	Std.Err. of Estimate	t-value	P-value	CI_{lb}	CI_{ub}	R^2	Fisher´s C	AICc
Global terrestrial ecosystems	Number of added plant species richness on tri-trophic interactions of plants, herbivores and parasitoids (bottom-up effects)	Number of added plant species richness	→Plant performance	1244	49	0.0003	0.0019	0.1579	0.8749	-0.0035	0.0041	0.00	48.737	9649.088
		Plant performance	→Herbivore performance	1244	49	-0.1451	0.0270	-5.3741	9.2217×10^{-8}	-0.1981	-0.0921	0.02	48.737	9649.088
		Herbivore performance	→Parasitoid performance	1244	49	-0.2732	0.0241	2.2222	0.0285	0.0004	0.0076	0.05	48.737	9649.088
		Number of added plant species richness	→Herbivore performance	1244	49	0.0040	0.0018	-11.3361	2.2646×10^{-28}	-0.3205	-0.2259	0.02	48.737	9649.088
		Number of added plant species richness	→Parasitoid performance	1244	49	0.0139	0.0018	7.7222	2.4135×10^{-14}	0.0104	0.0174	0.05	48.737	9649.088
	Number of added plant species richness on tri-trophic interactions of plants, herbivores and parasitoids (top-down effects)	Number of added plant species richness	→Parasitoid performance	1244	49	0.0132	0.0019	6.9474	6.0987×10^{-12}	0.0095	0.0169	0.02	27.758	9681.153
		Parasitoid performance	→Herbivore performance	1244	49	-0.2560	0.0274	-9.3431	1.3041×10^{-19}	-0.3098	-0.2022	0.07	27.758	9681.153
		Herbivore performance	→Plant performance	1244	49	-0.1544	0.0261	3.6667	0.0004	0.0030	0.0102	0.02	27.758	9681.153
		Number of added plant species richness	→Herbivore performance	1244	49	0.0066	0.0018	-5.9157	4.2986×10^{-9}	-0.2056	-0.1032	0.07	27.758	9681.153
		Number of added plant species richness	→Plant performance	1244	49	0.0007	0.0018	0.3889	0.6983	-0.0029	0.0043	0.02	27.758	9681.153
Organic croplands	Number of added plant species richness on tri-trophic interactions of plants, herbivores and parasitoids (bottom-up effects)	Number of added plant species richness	→Plant performance	593	23	0.0213	0.0171	1.2456	0.2181	-0.0130	0.0556	0.00	48.737	4392.619
		Plant performance	→Herbivore performance	593	23	-0.2544	0.0376	-6.7660	3.3042×10^{-11}	-0.3283	-0.1805	0.09	48.737	4392.619
		Herbivore performance	→Parasitoid performance	593	23	-0.2629	0.0302	-4.1622	5.7952×10^{-5}	-0.1136	-0.0404	0.06	48.737	4392.619
		Number of added plant species richness	→Herbivore performance	593	23	-0.0770	0.0185	-8.7053	3.4574×10^{-17}	-0.3222	-0.2036	0.09	48.737	4392.619
		Number of added plant species richness	→Parasitoid performance	593	23	0.1140	0.0166	6.8675	2.1706×10^{-11}	0.0814	0.1466	0.06	48.737	4392.619
	Number of added plant species richness on tri-trophic interactions of plants, herbivores and parasitoids (top-down effects)	Number of added plant species richness	→Parasitoid performance	593	23	0.1505	0.0170	8.8529	1.8513×10^{-17}	0.1171	0.1839	0.04	27.758	4391.313
		Parasitoid performance	→Herbivore performance	593	23	-0.3208	0.0413	-7.7676	5.4650×10^{-13}	-0.4023	-0.2393	0.11	27.758	4391.313
		Herbivore performance	→Plant performance	593	23	-0.2764	0.0352	-3.2513	0.0015	-0.0979	-0.0237	0.05	27.758	4391.313
		Number of added plant species richness	→Herbivore performance	593	23	-0.0608	0.0187	-7.8523	2.0823×10^{-14}	-0.3455	-0.2073	0.11	27.758	4391.313
		Number of added plant species richness	→Plant performance	593	23	-0.0075	0.0167	-0.4491	0.6553	-0.0410	0.0260	0.05	27.758	4391.313
	Number of added plant species	Number of added plant species richness	→Plant performance	266	11	0.0187	0.0329	0.5684	0.5722	-0.0473	0.0847	0.00	48.737	1997.994
		Plant performance	→Herbivore performance	266	11	-0.1675	0.0566	-2.9594	3.4091×10^{-3}	-0.2790	-0.0560	0.17	48.737	1997.994

Non-organic croplands	richness on tri-trophic interactions of plants, herbivores and parasitoids (bottom-up effects)	Herbivore performance	→Parasitoid performance	266	11	-0.5150	0.0543	-6.6844	2.0657×10^{-4}	-0.3057	-0.1475	0.19	48.737	1997.994
		Number of added plant species richness	→Herbivore performance	266	11	-0.2266	0.0339	-9.4843	1.9236×10^{-18}	-0.6219	-0.4081	0.17	48.737	1997.994
		Number of added plant species richness	→Parasitoid performance	266	11	0.0778	0.0376	2.0691	0.0395	0.0038	0.1518	0.19	48.737	1997.994
	Number of added plant species richness on tri-trophic interactions of plants, herbivores and parasitoids (top-down effects)	Number of added plant species richness	→Parasitoid performance	266	11	0.2442	0.0386	6.3264	1.1126×10^{-9}	0.1682	0.3202	0.07	27.758	1973.601
		Parasitoid performance	→Herbivore performance	266	11	-0.5076	0.0474	-10.7089	9.2560×10^{-21}	-0.6012	-0.4140	0.40	27.758	1973.601
		Herbivore performance	→Plant performance	266	11	-0.2123	0.0546	-4.8754	3.0656×10^{-3}	-0.2181	-0.0715	0.04	27.758	1973.601
		Number of added plant species richness	→Herbivore performance	266	11	-0.1448	0.0297	-3.8883	1.5363×10^{-4}	-0.3202	-0.1044	0.40	27.758	1973.601
		Number of added plant species richness	→Plant performance	266	11	-0.0406	0.0355	-1.1437	0.2613	-0.1129	0.0317	0.04	27.758	1973.601
Grasslands	Number of added plant species richness on tri-trophic interactions of plants, herbivores and parasitoids (bottom-up effects)	Number of added plant species richness	→Plant performance	254	10	-0.0013	0.0020	-0.6500	0.5203	-0.0054	0.0028	0.00	48.753	2018.053
		Plant performance	→Herbivore performance	254	10	0.0227	0.0533	0.4259	0.6706	-0.0823	0.1277	0.02	48.753	2018.053
		Herbivore performance	→Parasitoid performance	254	10	0.1646	0.0621	2.7778	0.0086	0.0013	0.0087	0.13	48.753	2018.053
		Number of added plant species richness	→Herbivore performance	254	10	0.0050	0.0018	2.6506	0.0092	0.0415	0.2877	0.02	48.753	2018.053
		Number of added plant species richness	→Parasitoid performance	254	10	0.0106	0.0019	5.5789	6.7418×10^{-8}	0.0069	0.0143	0.13	48.753	2018.053
	Number of added plant species richness on tri-trophic interactions of plants, herbivores and parasitoids (top-down effects)	Number of added plant species richness	→Parasitoid performance	254	10	0.0111	0.0019	5.8421	1.7545×10^{-8}	0.0074	0.0148	0.10	28.365	2023.719
		Parasitoid performance	→Herbivore performance	254	10	0.0341	0.0590	0.5780	0.5648	-0.0832	0.1514	0.02	28.365	2023.719
		Herbivore performance	→Plant performance	254	10	0.0199	0.0633	2.4211	0.0218	0.0007	0.0085	0.00	28.365	2023.719
		Number of added plant species richness	→Herbivore performance	254	10	0.0046	0.0019	0.3144	0.7535	-0.1048	0.1446	0.02	28.365	2023.719
		Number of added plant species richness	→Plant performance	254	10	-0.0014	0.0021	-0.6667	0.5098	-0.0057	0.0029	0.00	28.365	2023.719
Forests	Number of added plant species richness on tri-trophic interactions of plants, herbivores and parasitoids (bottom-up effects)	Number of added plant species richness	→Plant performance	131	5	0.0002	0.0136	0.0147	0.9883	-0.0267	0.0271	0.00	48.753	1037.502
		Plant performance	→Herbivore performance	131	5	0.1327	0.0651	2.0384	0.0437	0.0038	0.2616	0.07	48.753	1037.502
		Herbivore performance	→Parasitoid performance	131	5	0.0121	0.0818	4.6931	7.0651×10^{-6}	0.0274	0.0674	0.01	48.753	1037.502
		Number of added plant species richness	→Herbivore performance	131	5	0.0474	0.0101	0.1479	0.8827	-0.1498	0.1740	0.07	48.753	1037.502
		Number of added plant species richness	→Parasitoid performance	131	5	0.0165	0.0107	1.5421	0.1256	-0.0047	0.0377	0.01	48.753	1037.502
	Number of added plant species richness on tri-trophic interactions of plants, herbivores and parasitoids (top-down effects)	Number of added plant species richness	→Parasitoid performance	131	5	0.0170	0.0100	1.7000	0.0916	-0.0028	0.0368	0.01	28.365	1038.682
		Parasitoid performance	→Herbivore performance	131	5	-0.0047	0.0754	-0.0623	0.9504	-0.1541	0.1447	0.06	28.365	1038.682
		Herbivore performance	→Plant performance	131	5	0.1421	0.0906	4.6117	1.0041×10^{-5}	0.0271	0.0679	0.02	28.365	1038.682
		Number of added plant species richness	→Herbivore performance	131	5	0.0475	0.0103	1.5684	0.1193	-0.0372	0.3214	0.06	28.365	1038.682
		Number of added plant species richness	→Plant performance	131	5	-0.0054	0.0140	-0.3857	0.7004	-0.0331	0.0223	0.02	28.365	1038.682

Captions for the supplementary data

Data S1 Data collection for tri-trophic interactions to address raw data for each replicate in each year.

Data S2 Lists of global experiments of plant species diversity effects on tri-trophic groups in our analyses. If invertebrate herbivore management technologies were used in a certain experiment, the amount and species of pesticides and the use of non-pesticide technologies should be the same in all plots in each experiment.

Data S3. Results of the path analyses to test the bottom-up and top-down effects of each gradient of number of added plant species richness on the tri-trophic interactions of plant performance, invertebrate herbivore performance and their invertebrate natural enemy performance in croplands (as presented also in fig. S19). Number of added plant species is a continuous variable describing the increase in plant species richness between the control (monocultures of the lowest experimental species richness of plants) and the treatment containing an increased number of plant species richness relative to the control. The predictor and response columns specify the trophic group pairs and the moderator category. The estimate represents the strength of the relationship. The std. err. of estimate denotes the standardized error of the estimate coefficients for the fitted path-analytic models. The number of studies and observations for the predictor-response pair are also presented. Each test is two-sided and the original P value is reported with no multiple comparisons. In addition, test statistic (t value), and 95% confidence interval are reported. R^2 represents the proportion of variance explained for each endogenous variable, the reported R^2 is marginal which represents variance explained by fixed effects only. Fisher's C statistic assesses the goodness-of-fit of the model through Shipley's test of directed separation. AICc is the adjusted akaike information criterion, the strength of top-down and bottom-up pathway can be assessed using the difference of the AICc.

Data S4. Results of the path analyses to test the bottom-up and top-down effects of each gradient of number of added plant species richness on the tri-trophic interactions of plant performance, invertebrate herbivore performance and their invertebrate natural enemy performance in grasslands (as presented also in fig. S20). Number of added plant species is a continuous variable describing the increase in plant species richness between the control (monocultures of the lowest experimental species richness of plants) and the treatment containing an increased number of plant species richness relative to the control. The predictor and response columns specify the trophic group pairs and the moderator category. The estimate represents the strength of the relationship. The std. err. of estimate denotes the standardized error of the estimate coefficients for the fitted path-analytic models. The number of studies and observations for the predictor-response pair are also presented. Each test is two-sided and the original P value is reported with no multiple comparisons. In addition, test statistic (t value), and 95% confidence interval are reported. R^2 represents the proportion of variance explained for each endogenous variable, the reported R^2 is marginal which represents variance explained by fixed effects only. Fisher's C statistic assesses the goodness-of-fit of the model through Shipley's test of directed separation. AICc is the adjusted akaike information criterion, the strength of top-down and bottom-up pathway can be assessed using the difference of the AICc.

Data S5. Results of the path analyses to test the bottom-up and top-down effects of each gradient of number of added plant species richness on the tri-trophic interactions of plant performance, invertebrate herbivore performance and their invertebrate natural enemy performance in forests (as presented also in fig. S21). Number of added plant species is a continuous variable describing the increase in plant species richness between the control (monocultures of the lowest experimental species richness of plants) and the treatment containing an increased number of plant species richness relative to the control. The predictor and response columns specify the trophic group pairs and the moderator category. The estimate represents the strength of the relationship. The std. err. of estimate denotes the standardized error of the estimate coefficients for the fitted path-analytic models. The number of studies and observations for the predictor-response pair are also presented. Each test is two-sided and the original P value is reported with no multiple comparisons. In addition, test statistic (t value), and 95% confidence

interval are reported. R^2 represents the proportion of variance explained for each endogenous variable, the reported R^2 is marginal which represents variance explained by fixed effects only. Fisher's C statistic assesses the goodness-of-fit of the model through Shipley's test of directed separation. AICc is the adjusted akaike information criterion, the strength of top-down and bottom-up pathway can be assessed using the difference of the AICc.

Data S6 Relationship between standardized herbivore performance and standardized plant performance. The whole data is divided different subgroups according to experiment type and ecosystem type ,and then was used to conduct simple linear regression to investigate the relationship between standized herbivore performance and standized plant performance. In addition, the ecosystem type was re-divided into four categories (i.e., organic cropland v.s. non-organic cropland & grassland & forest, non-organic cropland v.s. organic cropland & grassland & forest, grassland v.s. organic cropland & non-organic cropland & forest, and forest v.s. organic cropland & non-organic cropland & grassland) to investigate the interaction effect of ecosystem using mixed linear regression. The table gives the predictor variables included, the number of observations and studies, the degree of freedom (d.f.), the corresponding regression equation, the 95% confidence interval, the slope difference, standard error (Std.Error), test-statistics (t-value), the corresponding P-value and R^2.

Data S7 Relationship between standardized herbivore performance and standardized natural enemy performance. The whole data is divided different subgroups according to experiment type and ecosystem type ,and then was used to conduct simple linear regression to investigate the relationship between standized herbivore performance and standized natural enemy performance. In addition, the ecosystem type was re-divided into four categories (i.e., organic cropland v.s. non-organic cropland & grassland & forest, non-organic cropland v.s. organic cropland & grassland & forest, grassland v.s. organic cropland & non-organic cropland & forest, and forest v.s. organic cropland & non-organic cropland & grassland) to investigate the interaction effect of ecosystem using mixed linear regression. The table gives the predictor variables included, the number of observations and studies, the degree of freedom (d.f.), the corresponding regression equation, the 95% confidence interval, the slope difference, standard error (Std.Error), test-statistics (t-value), the corresponding P-value and R^2.
